# Studies of Health Insurance Claims Data in Japan: A Scoping Review

**DOI:** 10.31662/jmaj.2022-0184

**Published:** 2023-06-19

**Authors:** Maiko Suto, Takehiro Sugiyama, Kenjiro Imai, Takashi Furuno, Mariko Hosozawa, Yuichi Ichinose, Noriko Ihana-Sugiyama, Tomoko Kodama, Ryuji Koizumi, Yuko Shimizu-Motohashi, Shunsuke Murata, Yayoi Nakamura, Mariko Niino, Misuzu Sato, Reina Taguchi, Misa Takegami, Motoko Tanaka, Kota Tsutsumimoto, Kentaro Usuda, Kenji Takehara, Hiroyasu Iso

**Affiliations:** 1 Department of Health Policy, National Center for Child Health and Development, Tokyo, Japan; 2 Institute for Global Health Policy Research, Bureau of International Health Cooperation, National Center for Global Health and Medicine, Tokyo, Japan; 3 Department of Health Services Research, Institute of Medicine, University of Tsukuba, Ibaraki, Japan; 4 Diabetes and Metabolism Information Center, Research Institute, National Center for Global Health and Medicine, Tokyo, Japan; 5 Division of Health Services Research, Institute for Cancer Control, National Cancer Center, Tokyo, Japan; 6 Department of Public Health Policy, National Institute of Public Health, Saitama, Japan; 7 AMR Clinical Reference Center, Disease Control and Prevention Center, National Center for Global Health and Medicine, Tokyo, Japan; 8 Department of Child Neurology, National Center Hospital, National Center of Neurology and Psychiatry, Tokyo, Japan; 9 Department of Preventive Medicine and Epidemiology, National Cerebral and Cardiovascular Center, Osaka, Japan; 10 AIDS Clinical Center, National Center for Global Health and Medicine, Tokyo, Japan; 11 Department of Clinical Epidemiology and Health Economics, School of Public Health, The University of Tokyo, Tokyo, Japan; 12 Institute for Health Economics and Policy, Tokyo, Japan; 13 Department of Health Care Policy and Management, Doctoral Program in Public Health, Degree Programs in Comprehensive Human Sciences, Graduate School of Comprehensive Human Sciences, University of Tsukuba, Ibaraki, Japan; 14 Department of Preventive Gerontology, Center for Gerontology and Social Science, National Center for Geriatrics and Gerontology, Aichi, Japan; 15 Department of Public Mental Health Research, National Institute of Mental Health, National Center of Neurology and Psychiatry, Tokyo, Japan

**Keywords:** health insurance claims, validation studies, healthcare policy, Japan, scoping review

## Abstract

**Background:**

Health insurance claims data are used in various research fields; however, an overview on how they are used in healthcare research is scarce in Japan. Therefore, we conducted a scoping review to systematically map the relevant studies using Japanese claims data.

**Methods:**

MEDLINE, EMBASE, and Ichushi-Web were searched up to April 2021 for studies using Japanese healthcare claims data. We abstracted the data on study characteristics and summarized target diseases and research themes by the types of claims database. Moreover, we described the results of studies that aimed to compare health insurance claims data with other data sources narratively.

**Results:**

A total of 1,493 studies were included. Overall, the most common disease classifications were “Diseases of the circulatory system” (18.8%, n = 281), “Endocrine, nutritional, and metabolic diseases” (11.5%, n = 171; mostly diabetes), and “Neoplasms” (10.9%, n = 162), and the most common research themes were “medical treatment status” (30.0%, n = 448), “intervention effect” (29.9%, n = 447), and “clinical epidemiology, course of diseases” (27.9%, n = 417). Frequent diseases and themes varied by type of claims databases. A total of 19 studies aimed to assess the validity of the claims-based definition, and 21 aimed to compare the results of claims data with other data sources. Most studies that assessed the validity of claims data compared to medical records were hospital-based, with a small number of institutions.

**Conclusions:**

Claims data are used in various research areas and will increasingly provide important evidence for healthcare policy in Japan. It is important to use previous claims database studies and share information on methodology among researchers, including validation studies, while informing policymakers about the applicability of claims data for healthcare planning and management.

## Introduction

Understanding disease and treatment patterns is important for developing appropriate health policies at the national and regional levels. For healthcare research, the use of healthcare databases that represent routine clinical practice has several advantages: an adequate population to study rare events, the reflection of real-world effectiveness and practice patterns, and a relatively low cost and short time ^(1)^.

One of the most used healthcare databases involves health insurance claims data for healthcare services, procedures, and pharmaceuticals ^(1)^. These claims data are used in various research areas, such as health service utilization, cost analysis, intervention and evaluation studies, drug risk assessment, health policy research, and guideline adherence ^(2), (3), (4)^. These data have the potential to provide important evidence for Japanese healthcare policies. To conduct future claims database studies, a systematic summary of previous studies would be a useful resource from several perspectives. First, a research overview of claims database studies will reveal the well-addressed research areas (e.g., targeted diseases and research themes) and warrant further research. Second, the list of relevant previous studies will provide methodological guidance for future research, including how to define diseases using diagnostic and procedure codes. In addition, findings regarding what data sources have been used for comparison are important in claims database studies ^(1), (5)^.

Some review studies and data profiles have described the research areas and validation studies for Japanese claims database studies. However, these reviews included only certain claims databases, such as the National Database of Health Insurance Claims (NDB) ^(6)^ and JMDC Claims Database (JMDC) ^(7)^; the overall distribution of research areas, as well as differences by type of claims database, has not yet been revealed. Regarding validation studies, one review study searched PubMed and reported the studies published in English ^(5)^. However, a comprehensive review, including Japanese electronic sources, remains lacking. A thorough literature review of previous claims database studies, using multiple electronic sources, is required to facilitate claims database study in Japan. Therefore, this scoping review, which systematically mapped the studies using claims data in Japan, aimed to investigate (1) the distribution of target diseases in each study, (2) the details and distribution of research themes, and (3) the types of studies that aimed to assess the validity of claims data or compare their results with other data sources, such as medical records, registries, and surveillance data.

## Materials and Methods

### Eligibility criteria

Studies using Japanese health insurance claims data published after 2010 in Japanese or English were included.

Study papers using only health checkup data or long-term care insurance claims data, publicly available data that were freely available on the Internet (e.g., NDB Open Data, which are summary tables of NDB data compiled by the government), data combined with primary research (including controlled trials, cohort studies, and surveys), and not original data (e.g., editorials, commentaries, reviews, and conference abstracts) or theses were excluded. Hospital-based studies involving ten or fewer institutions were also excluded.

Studies that compared results of claims data with other data sources were included regardless of the number of institutions that participated in each study. This was because most studies that assessed the validity of claims data were hospital-based, with fewer institutions, and did not meet the abovementioned inclusion criteria.

### Search and selection of sources of evidence

To identify potentially relevant documents, electronic sources such as MEDLINE, EMBASE, and Ichushi-Web were searched up to April 2021. Two experienced information specialists assessed the search strategies (Supplementary File 1 shows the complete electronic search strategies).

A total of 14 reviewers working in pairs independently assessed the titles and abstracts retrieved from the electronic searches for review inclusion, using the Rayyan software. We sourced and assessed full papers when their eligibility for this review was unclear from the title and abstract alone. One of the reviewers conducted full-text screening and extracted data from the studies that potentially met our inclusion criteria, using the data-extracting form and manual developed for this review through discussion. Another reviewer (MS) confirmed the decisions regarding inclusion/exclusion and results of data extraction throughout the entire study to ensure consistency of categories.

### Data items and synthesis of results

We abstracted data on publication year, type of claims database (classified as NDB; JMDC; Medical Data Vision EBM Provider (MDV); Diagnosis Procedure Combination Database (DPC); National Health Insurance (NHI), including Kokuho Database (KDB)/Latter-Stage Elderly Healthcare System (LSEHS); Japan Health Insurance Association (JHIA); and Other/Multiple), study setting (national, regional, and others), age of study sample (children, older persons, and others), targeted disease (International Classification of Diseases-10 (ICD-10) chapter classification), and research theme (classified as 12 categories; these categories concerned related studies ^(2), (3), (4)^. For targeted disease and research themes, we selected up to two categories for each study (studies that targeted more than two diseases were classified as “others”).

We summarized the study characteristics (setting and age of study sample) (Table 1), target diseases (Table 2), and research themes (Table 3) by type of claims database. We picked up studies that aimed to assess the validity of claims data or compare their results with other data sources, describing the results narratively. We conducted this review according to the PRISMA-ScR reporting guideline (Supplementary File 2) ^(8)^.

**Table 1.** Characteristics of Included Studies.

		All		NDB		JMDC		MDV		DPC		NHI/LSEHS		JHIA		Other/Multiple	
	Total	1493	%	102	%	340	%	187	%	584	%	160	%	12	%	108	%
**Setting**	National	1267	84.9	97	95.1	339	99.7	187	100	567	97.1	2	1.3	2	16.7	73	67.6
	Regional	156	10.4	3	2.9	0	0.0	0	0.0	5	0.9	129	80.6	10	83.3	9	8.3
	Others	70	4.7	2	2.0	1	0.3	0	0.0	12	2.1	29	18.1	0	0.0	26	24.1
**Age**	Children	94	6.3	7	6.9	33	9.7	7	3.7	39	6.7	3	1.9	0	0.0	5	4.6
	Older persons	134	9	10	9.8	9	2.6	4	2.1	29	5.0	80	50.0	0	0.0	2	1.9
	Others	1265	84.7	85	83.3	298	87.6	176	94.1	516	88.4	77	48.1	12	100.0	101	93.5

*Note.* *NDB, National Database of Health Insurance Claims; JMDC, JMDC Claims Database; MDV, Medical Data Vision EBM Provider; DPC, Diagnosis Procedure Combination Database; NHI, National Health Insurance, including Kokuho Database (KDB)/Latter-Stage Elderly Healthcare System (LSEHS); JHIA, Japan Health Insurance Association*

**Table 2.** Disease Characteristics by Type of Claims Databases.

			Database														Age					
	All		NDB		JMDC		MDV		DPC		NHI/LSEHS		JHIA		Other/Multiple		Children		Older persons		Others	
**Total**	1493	%	102	%	340	%	187	%	584	%	160	%	12	%	108	%	94	%	134	%	1265	%
**1) Certain infectious and parasitic diseases**	117	7.8	15	14.7	29	8.5	16	8.6	41	7.0	4	2.5	0	0.0	12	11.1	14	14.9	0	0.0	103	8.1
**2) Neoplasms**	162	10.9	6	5.9	22	6.5	31	16.6	92	15.8	4	2.5	3	25.0	4	3.7	2	2.1	2	1.5	158	12.5
**3) Diseases of the blood and blood-forming organs and certain disorders involving the immune mechanism**	19	1.3	0	0.0	0	0.0	3	1.6	15	2.6	0	0.0	0	0.0	1	0.9	4	4.3	0	0.0	15	1.2
**4) Endocrine, nutritional, and metabolic diseases**	171	11.5	11	10.8	62	18.2	40	21.4	9	1.5	21	13.1	3	25.0	25	23.1	5	5.3	4	3.0	162	12.8
**5) Mental, behavioral, and neurodevelopmental disorders**	110	7.4	13	12.7	47	13.8	2	1.1	22	3.8	13	8.1	3	25.0	10	9.3	6	6.4	13	9.7	91	7.2
**6) Diseases of the nervous system**	69	4.6	4	3.9	18	5.3	10	5.3	24	4.1	7	4.4	0	0.0	6	5.6	3	3.2	9	6.7	57	4.5
**7) Diseases of the eye and adnexa**	22	1.5	1	1.0	13	3.8	1	0.5	3	0.5	0	0.0	0	0.0	4	3.7	0	0.0	0	0.0	22	1.7
**8) Diseases of the ear and mastoid process**	4	0.3	0	0.0	3	0.9	0	0.0	1	0.2	0	0.0	0	0.0	0	0.0	3	3.2	0	0.0	1	0.1
**9) Diseases of the circulatory system**	281	18.8	11	10.8	49	14.4	41	21.9	138	23.6	21	13.1	3	25.0	18	16.7	3	3.2	11	8.2	267	21.1
**10) Diseases of the respiratory system**	135	9.0	11	10.8	38	11.2	10	5.3	56	9.6	13	8.1	0	0.0	7	6.5	24	25.5	22	16.4	89	7.0
**11) Diseases of the digestive system**	115	7.7	6	5.9	24	7.1	8	4.3	61	10.4	11	6.9	1	8.3	4	3.7	6	6.4	8	6.0	101	8.0
**12) Diseases of the skin and subcutaneous tissue**	17	1.1	2	2.0	9	2.6	1	0.5	3	0.5	1	0.6	0	0.0	1	0.9	3	3.2	1	0.7	13	1.0
**13) Diseases of the musculoskeletal system and connective tissue**	93	6.2	7	6.9	25	7.4	16	8.6	36	6.2	5	3.1	0	0.0	4	3.7	5	5.3	7	5.2	81	6.4
**14) Diseases of the genitourinary system**	75	5.0	4	3.9	15	4.4	9	4.8	34	5.8	7	4.4	1	8.3	5	4.6	1	1.1	5	3.7	69	5.5
**15) Pregnancy, childbirth, and the puerperium**	25	1.7	4	3.9	9	2.6	1	0.5	11	1.9	0	0.0	0	0.0	0	0.0	0	0.0	0	0.0	25	2.0
**16) Certain conditions originating in the perinatal period**	8	0.5	0	0.0	3	0.9	1	0.5	3	0.5	0	0.0	0	0.0	1	0.9	4	4.3	0	0.0	4	0.3
**17) Congenital malformations, deformations, and chromosomal abnormalities**	13	0.9	0	0.0	3	0.9	1	0.5	8	1.4	0	0.0	0	0.0	1	0.9	11	11.7	0	0.0	2	0.2
**18) Injury, poisoning, and certain other consequences of external causes**	110	7.4	11	10.8	9	2.6	3	1.6	77	13.2	9	5.6	0	0.0	1	0.9	7	7.4	22	16.4	81	6.4
**19) Others (multidisease, not focused on specific diseases)**	238	15.9	21	20.6	41	12.1	21	11.2	69	11.8	68	42.5	0	0.0	18	16.7	16	17.0	50	37.3	172	13.6

*Up to two diseases could be chosen.

**Table 3.** Research Theme by Type of Claims Databases.

	All		NDB		JMDC		MDV		DPC		NHI/LSEHS		JHIA		Other/Multiple	
**Total**	1493	%	102	%	340	%	187	%	584	%	160	%	12	%	108	%
**Medical treatment status**	448	30.0	40	39.2	149	43.8	73	39.0	106	18.2	43	26.9	2	16.7	35	32.4
**Intervention effect**	447	29.9	17	16.7	85	25.0	60	32.1	259	44.3	15	9.4	1	8.3	10	9.3
**Clinical epidemiology, course of diseases**	417	27.9	33	32.4	92	27.1	50	26.7	187	32.0	36	22.5	4	33.3	15	13.9
**Health economics**	211	14.1	10	9.8	47	13.8	43	23.0	42	7.2	44	27.5	3	25.0	22	20.4
**Health policy evaluation and utilization**	186	12.5	9	8.8	23	6.8	8	4.3	78	13.4	49	30.6	4	33.3	15	13.9
**Quality of care**	91	6.1	11	10.8	30	8.8	9	4.8	29	5.0	8	5.0	0	0.0	4	3.7
**Research methodology**	77	5.2	15	14.7	12	3.5	6	3.2	22	3.8	6	3.8	0	0.0	16	14.8
**Patient health service utilization**	76	5.1	1	1.0	31	9.1	6	3.2	3	0.5	16	10.0	2	16.7	17	15.7
**Socioeconomic comparison**	56	3.8	8	7.8	7	2.1	2	1.1	14	2.4	17	10.6	2	16.7	6	5.6
**Prediction model**	36	2.4	1	1.0	4	1.2	2	1.1	22	3.8	3	1.9	0	0.0	4	3.7
**COVID-19**	11	0.7	0	0.0	1	0.3	5	2.7	4	0.7	1	0.6	0	0.0	0	0.0
**Others**	8	0.5	0	0.0	4	1.2	0	0.0	2	0.3	1	0.6	0	0.0	1	0.9

*Up to two themes could be chosen.

## Results

**Figure 1.**
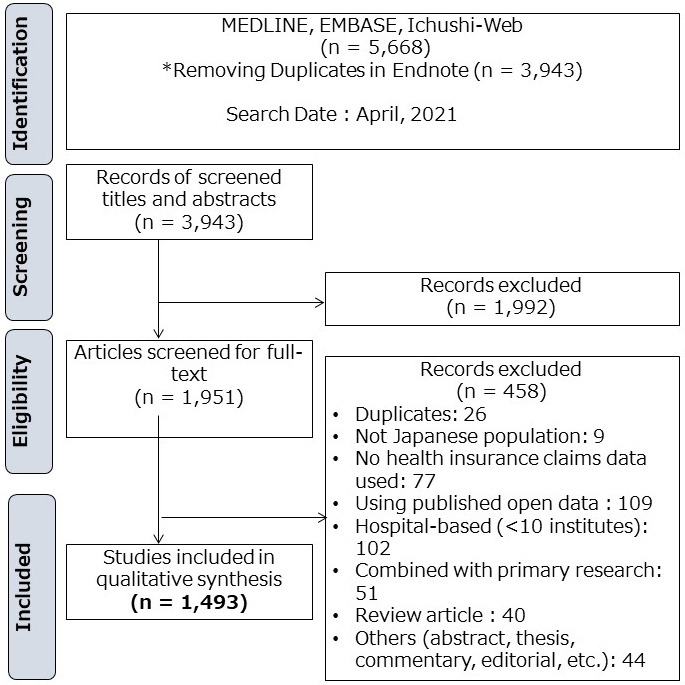
Study selection flowchart.

After duplicates were removed, 3,943 citations were identified from electronic searches. Based on the title and abstract, 1,992 were excluded. A total of 1,951 sources for eligibility in the full-text screening were assessed and 458 citations were excluded. The remaining 1,493 studies were considered eligible for this review. Figure 1 shows the study selection flowchart; Supplementary File 3 shows the excluded studies, with reasons for exclusion in the full-text screening.

### Characteristics of included studies

**Figure 2.**
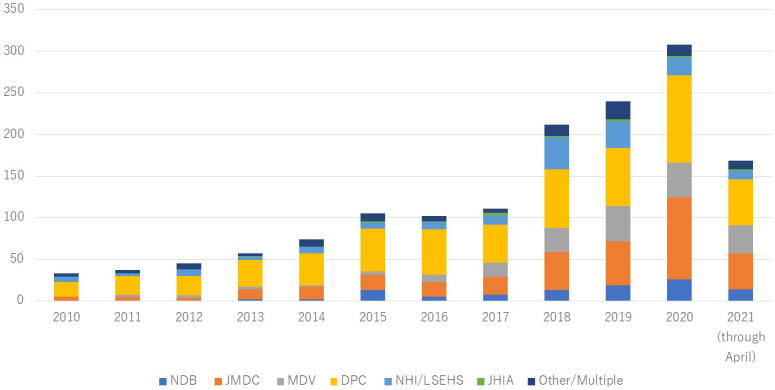
Annual number of publications between 2010 and 2021 National Database of Health Insurance Claims (NDB); JMDC Claims Database (JMDC); Medical Data Vision EBM Provider (MDV); Diagnosis Procedure Combination Database (DPC); National Health Insurance (NHI), including Kokuho Database (KDB)/Latter-Stage Elderly Healthcare System (LSEHS); Japan Health Insurance Association (JHIA).

The number of published studies using health insurance claims data increased since 2010 (Figure 2). We grouped these studies by the type of claims database and described the study setting and age of study sample for each group (Table 1). Supplementary File 4 shows characteristics of the individual included studies. The largest number of studies used DPC (n = 584), followed by JMDC (n = 340). We found 102 NDB studies, including 21 studies using NDB sampling data and 12 using accumulated NDB data. In the DPC studies, several types of databases were found: (1) data collected by DPC study groups and institutions, such as DPC Research Institute, Quality Indicator/Improvement Project (QIP) database, and National Hospital Organization and (2) data being combined with specific disease registries, such as Hospital-based Cancer Registries, J-ASPECT Study (nationwide stroke registry), and JROAD-DPC (Japanese Registry Of All cardiac and vascular Diseases). The “Other” category database included (1) health insurance societies-based claims database, such as JammNet claims database, MinaCare, and other corporate health insurance societies; (2) pharmacy claims data, such as IQVIA NPA data, Medi-Trend (Kyowa Kikaku), and Nihon-Chouzai pharmacy claims database; (3) databases sourced from medical institutions; and (4) studies using multiple claims databases together (e.g., JMDC and MDV).

NDB, JMDC, MDV, and DPC were mostly analyzed at the nationwide level, while NHI/LSEHS and JHIA were examined at the regional level. Regarding the age of the study sample, half of the studies (80/160 studies) using NHI and LSEHS, which are municipality-based claims databases and do not include the employee health insurance claims data, targeted the older population (several studies combined with long-term care insurance data), and only three studies targeted children. On the other hand, in JMDC studies, which collected data from corporate health insurance societies, 33 studies targeted children and only 9 targeted the older population.

### Distribution of target diseases

We summarized target diseases for each type of claims database (Table 2). Overall, the most common disease classifications were “Diseases of the circulatory system” (18.8%, n = 281/1493), “Endocrine, nutritional, and metabolic diseases” (11.5%, n = 171/1493; the vast majority dealt with diabetes), and “Neoplasms” (10.9%, n = 162/1493). On the other hand, the number of studies regarding blood and immune system diseases, as well as eye, ear, skin, and perinatal diseases, was small.

The frequency of diseases varied depending on the type of claims database. There were only few studies on “Endocrine, nutritional, and metabolic diseases,” including diabetes, in DPC (1.5%, n = 9/584), while there were many such studies in JMDC and MDV. For “Neoplasms,” there were few studies in NDB (5.9%, n = 6/102), JMDC (6.5%, n = 22/340), and NHI/LSEHS (2.5%, n = 4/160).

Of the included studies, 238 were classified as “Others.” This category included topics such as antimicrobial use, healthcare costs, hospitalizations/intensive care unit, and dealing with multiple diseases. Many studies did not focus on a specific disease in NHI/LSEHS, especially those targeting the older population. Nevertheless, they dealt with healthcare costs or delivery systems, such as hospitalization and home healthcare.

In studies of children, respiratory and infectious diseases, such as asthma, upper respiratory tract infection, and influenza, were more common. At the same time, pneumonia and fractures were more common in studies of the older population. Supplementary File 5 shows a list of frequently occurring diseases by ICD-10 chapter classification.

### Distribution of research themes

We summarized research themes for each type of claims database (Table 3). Overall, the most common research objectives were “medical treatment status” (describe the patterns of providing medical care, such as diagnosis, treatment, tests, and prescriptions; 30.0%, n = 448/1493), “intervention effect” (examine the effects and risks of treatment, such as surgery, prescriptions, rehabilitation; 29.9%, n = 447/1493), and “clinical epidemiology, course of diseases” (examine the prevalence, risk factors, prognosis, etc.; 27.9%, n = 417/1493). These three research themes were common to all disease categories (Supplementary File 6). Table 4 shows more specific research themes.

**Table 4.** List of Research Theme.

**Medical treatment status**	Diagnosis pattern
	Treatment pattern
	Screening, test, monitoring (frequency, rate)
	Prescription pattern
	Hospitalization, readmission, NICU
	Emergency medicine
	Home-based care
**Intervention effect**	Effectiveness (treatment, prevention): surgery, procedures, drug, vaccine, periodontal management, follow-up, rehabilitation, health guidance, etc.
	Drug repositioning
	Safety
	Adverse outcomes
**Clinical epidemiology, course of diseases**	Prevalence, incidence, number of patients
	Association, risk factor, predictor, prognostic factor
	Clinical characteristics (age, sex)
	Comorbidity, underlying conditions
	Mortality, survival, prognosis
	Clinical course
	Surveillance, seasonality, yearly change
**Health economics**	Cost-effectiveness
	Cost analysis
	Factors associated with medical expenditure
	Economic impact
	Medical billing issues
**Health policy evaluation and utilization**	Health policy impact: medical subsidy, payment system, drug approval, regulatory action on drug, labeling change on prescriptions OTC switching, clinical guideline, information campaign, etc.
	Hospital performance
	Volume-outcome relationship (other healthcare factors)
	Supply and demand for healthcare
	Post-marketing surveillance
	Analysis of healthcare region
	Use for healthcare planning
	Evaluation of resource consumption
	Analysis of home care service utilization
**Quality of care**	Adherence to clinical guideline
	Quality indicator
	Safety indicator
	Healthcare benchmarking
**Research methodology**	Validation study
	Comparison with other data sources
	Patient traceability
	Definition of death
	Correction methods for medical fee revisions
	Development of database
	Development of indicator/algorithm
**Patient health service utilization**	Adherence, compliance, persistence
	Treatment initiation
	Follow-up visits
	Healthcare utilization
	Healthcare-seeking behavior
	Patient choice, selection
**Socioeconomic comparison**	Regional variations
	Age and sex distribution
	Income-related inequality
	Residence (home, care facilities)
	Type of health insurance
	International comparison
**Prediction model**	Predict survival/mortality
	Severe adverse events
	Hospitalization
	Comorbidity scores
	High-need high-cost patients
	Prediction model of infectious disease
**COVID-19**	Trends in hospitalizations (NICU) during the COVID-19 outbreak
	Changes in intervention/care practice
	Adverse events of COVID-19 vaccines
	School closure and social distancing for COVID-19
	Economic impact of COVID-19 pandemic
**Others**	Effect of earthquakes
	Others

By type of claims database, DPC was characterized by a large number of studies on “intervention effect” (44.3%, n = 259/584) and a few on “medical treatment status” (18.2%, n = 106/584). Studies using NHI/LSEHS, a municipality-based claims database, often aimed at “health economics” and “health policy evaluation and utilization.” On the other hand, the number of studies varied by prefecture. The most common region was “Fukuoka,” with 32 studies; although some studies had anonymous municipality names, there was no report for several prefectures. The claims databases used in several studies on COVID-19 were MDV and DPC (QIP database) (literature search conducted in April 2021).

### Studies comparing claims data with other data sources

A total of 19 studies aimed to assess the validity of claims-based definition ^(9), (10), (11), (12), (13), (14), (15), (16), (17), (18), (19), (20), (21), (22), (23), (24), (25), (26), (27)^ and 21 aimed to compare results of claims data with other data sources, to evaluate the usefulness of claims databases as statistics or survey data ^(28), (29), (30), (31), (32), (33), (34), (35), (36), (37), (38), (39), (40), (41), (42), (43), (44), (45), (46), (47), (48)^. Depending on the purpose of the study, the following data sources were used as comparative data for claims data: to assess the accuracy of diagnoses and procedure records in claims data, 13 studies compared the data to medical records and laboratory data (chart review) ^(10), (11), (12), (13), (17), (19), (20), (21), (22), (23), (24), (26), (27)^, and 2 compared them to disease registries, linking claims data with individual-based information ^(9), (25)^. Two studies assessed claims-based definitions of death using enrollment data ^(14), (16)^. In addition, a study conducted a validity assessment of self-reported medication use that was collected in an annual health checkup, by comparing claims data to pharmacy insurance claims ^(18)^; one study examined the association between prognostic burn index (from DPC) and mortality ^(15)^. On the other hand, to understand the utility of claims data as statistical and survey data, such as the number of patients, incidence of events, and medication use, 4 studies made comparisons with electronic medical records data ^(29), (35), (47), (48)^, 4 with government statistics ^(28), (32), (43), (46)^, and 13 with disease registries, epidemiological studies, surveillance, post-marketing surveillance, and sales data ^(30), (31), (33), (34), (36), (37), (38), (39), (40), (41), (42), (44), (45)^.

The target diseases were mostly related to diabetes and/or cardiovascular diseases (13 studies). Studies that linked and compared results from claims data with individual data from medical records and disease registries were hospital-based, involving a single or few centers (five or fewer), with the exception of one study on cardiovascular disease in diabetic patients ^(20)^.

## Discussion

### Distribution of target diseases

Disease classifications with the highest number of studies were “Diseases of the circulatory system,” “Endocrine, nutritional, and metabolic diseases” (the vast majority of studies dealt with diabetes), and “Neoplasms.” These three diseases are included in the five major diseases in Japan (cancer, stroke, acute myocardial infarction, diabetes, and psychiatric diseases), for which local governments are required to monitor healthcare indicators and have a large number of patients.

This study showed that there were still some disease classifications for which few previous studies existed. Among the five diseases, the number of studies on psychiatric diseases was relatively low. In addition, the number of studies on more specific diseases, such as blood and immune system diseases, as well as eye, ear, skin, and perinatal diseases, was low. For these disease classifications, it would be desirable to promote studies using claims data.

While some disease areas have specific reasons and technical barriers for the small number of studies, in recent years, with the increase in number of DPC studies, studies on diseases with fewer hospitalizations should account for a smaller percentage of total claims database studies. Moreover, in practice areas where there are nationwide databases, such as the National Clinical Database (with support from the Japan Surgical Society ^(49)^, there would be less demand for the use of claims data. There may also be technical barriers to claims database studies in specific disease areas, such as those with ambiguous diagnostic criteria. Large hospital-related variations make it difficult to determine the case definitions in claims data and describe the disease and treatment pattern. In addition, normal pregnancies and vaginal deliveries not covered by public health insurance make it difficult to obtain the whole picture of the perinatal disease. As described above, it should be noted that studies using a claims database may not be appropriate for some diseases.

The frequency of diseases also varied depending on the type of claims database. In DPC, the number of studies on “Endocrine, nutritional, and metabolic diseases,” including diabetes, was low. In JMDC claims databases sourced from health insurance societies, health checkup data were included to ascertain the health status of insured persons, as well as special health checkups to enable patient-based tracking if insured by the same health insurance society ^(7)^. These strengths make it easy to use in studies for chronic diseases.

### Distribution of research themes

Claims data were used for various research themes. Overall, the most common research objectives were “medical treatment status,” “intervention effect,” and “clinical epidemiology, course of diseases.” These three themes were considered to take advantage of claims data: (1) the studies involved a large population to understand real-world effectiveness and practice patterns for rare diseases ^(50), (51), (52)^, (2) these studies aimed to examine the external validity of clinical trials in a real-world setting with claims data ^(53)^, and (3) they provided a more detailed picture of the actual state of disease, combining the results of epidemiological studies or other data sources ^(42)^.

Studies that used NHI/LSEHS, a municipality-based claims database, often aimed at “health economics” and “health policy evaluation and utilization.” This review found various highly practical studies for municipal administrative planning and management. For instance, a study aimed to identify the number of vulnerable people in neighborhood units by using the NHI database to create an evacuation support plan ^(54)^. Some studies proposed to use claims database research as fundamental data for monitoring healthcare indicators in regional healthcare or cost moderation plans ^(55), (56), (57)^. While the number of reported papers varies among municipalities, it is important to provide information to policymakers and promote collaboration with researchers to use a claims database in each region.

The distribution of research themes by claims database was affected by the information contained in each database. For instance, DPC data (acute inpatient database) was characterized by many studies on the “intervention effect.” DPC databases include detailed information about patients and hospitalization, making it easier to determine the disease severity and clinical presentation; DPC data comprise basic and clinical information, including the day-to-day status of patients on Form 1 and the H-file ^(58)^. Likewise, MDV includes results of blood tests and other laboratory tests, in addition to DPC data information ^(31)^; JMDC claims databases sourced from medical institutions also include DPC assessment forms and clinical laboratory test values ^(59)^. Although NDB contains limited outcome data or clinical information, such as laboratory test results, compared to the above databases, it includes almost all health insurance claims in Japan and can be used to describe diseases and treatment patterns at the national level. JMDC claims databases sourced from health insurance societies have the ability to link household members, examining the impact of medication during pregnancy on infant outcomes ^(60), (61)^ and enabling analysis on patient/spouse pairs ^(62), (63)^. NHI and LSEMCS, sourced from municipal health insurance societies, also include health checkup results and enable individual-level linked data on long-term care insurance, thereby expanding research possibilities ^(64)^. As of April 2021, MDV and DPC (QIP database) have been used in several studies on COVID-19 ^(65), (66), (67), (68), (69), (70), (71), (72), (73)^; these databases are capable of rapid analysis in accordance with social conditions. It is important to consider the characteristics, strengths, and information in claims databases when a researcher plans to conduct a study using these databases.

### Studies comparing claims data with other data sources

Research using claims data is expected to significantly contribute to healthcare research. Nevertheless, a major challenge in using claims data for research purposes involves ensuring the data’s validity. Claims data are not collected for the primary purpose of research, so their quality may not be as robust as primary data collection ^(74)^. Therefore, validation studies are important to ensure the credibility of results.

We picked up two types of studies that compared claims data with other data sources: (1) studies that aimed to assess the validity of claims data and (2) those that aimed to evaluate the utility of claims data as statistical or survey data. For studies that aimed to assess the validity of data, the comparisons were conducted either at the aggregate level, such as using surveillance and statistical data, or at the individual data level, such as using medical records. Most of the studies that conducted chart reviews with medical records were hospital-based, with a small number of institutions. In these hospital-based studies, the authors mentioned a limitation in that it was unclear whether these results could be generalized to other hospitals. To ensure the credibility of results from claims data, more extensive support is needed ^(5)^.

### Limitations and strengths

Our scoping review has several limitations. First, although the search strategies were determined by experienced information specialists, our review failed to include some claims database studies. The search strategies in this review contained typical claims database names, such as NDB, DPC, JMDC, MDV, and KDB, and some related terms with “health insurance claims.” However, there are many ways to describe the names of each database and claims data. It was not possible to use all terms in our search strategies (e.g., we could not detect the studies described as “administrative data” in the title and abstract). This causes relevant records to be missed while acting as barrier to finding previous claims data studies. It may be necessary to unify the description method, such as including the database name in the title and abstract. In addition, our review did not include relevant studies not listed in the three electronic sources (MEDLINE, EMBASE, and Ichushi-Web). Second, two reviewers did not independently conduct full-text screening and data extraction due to the large number of retrieved studies. Among several reviewers, one conducted full-text screening and data extraction. A second reviewer confirmed the results of screening and data extraction throughout the study to minimize misclassification, considering whether the criteria differed among the reviewers.

Despite these limitations, we believe that this review can contribute to grasping the research overview of claims database studies in Japan, considering multiple electronic sources for literature search and any types of claims databases. Our findings showed that it is important to consider the strengths and limitations of each claims database when a researcher plans to conduct a study with them. In addition, when planning a new claims data study, the list of included studies in this review will provide the index information to previous claims data studies. It will allow researchers to refer to methodological issues, such as claims-based case definition. To facilitate healthcare research and evidence-based policy development, it is important to use previous studies using claims data and share information on methodology among researchers in each disease area and across diseases, including validation studies, while informing policymakers across the country about the applicability of claims data for healthcare planning and management.

## Article Information

### Conflicts of Interest

None

### Sources of Funding

This work was supported by Research Project for the Establishment of an NDB Research System for Health Policy and Other Purposes through 6NC Collaboration (2019-(1)-3)

### Acknowledgement

The authors thank Mr. Masahiko Watanabe and Ms. Chiemi Kataoka for developing and executing the search strategy. We express our gratitude to Dr. Sho Nakakubo, Dr. Satoshi Kurita, Dr. Yuto Kiuchi, and Dr. Kazuhei Nishimoto for their supports in data extraction.

### Author Contributions

MaS, TS, KI, TF, MH, YI, NI, TK, RK, YM, SM, YN, MN, MiT, KoT, KU, KeT, and HI designed the study. MaS, TS, KI, TF, MH, NI, RK, SM, YN, MN, MiT, MoT, KoT, and KeT conducted title and abstract screening. MaS, TS, KI, TF, MH, YI, NI, RK, YM, SM, YN, MN, MiS, RT, MiT, MoT, KoT, KU, and KeT conducted full-text screening and data extraction for included studies. MaS and KeT drafted the initial manuscript. All authors reviewed and approved the final manuscript.

### Approval by Institutional Review Board (IRB)

Not applicable

## Supplement

Supplementary Files
