# Supplementary material for: Studies of Health Insurance Claims Data in Japan: A Scoping Review
Source: JMA J. 2023 Jun 19;6(3):233–45. doi: 10.31662/jmaj.2022-0184 (PMC10407298; doi:10.31662/jmaj.2022-0184)
Supplement: Supplementary Files [file 2433-3298-6-3-0233-s001.pdf]

Supplement file 1 Search strategy

	Ichushi-Web	Results
#1	NDB/AL and (data/TA or information/TA or (database/TH or database/AL))	451
#2	(nation/TA or Japan/TA) and (health/TA or insurance/TA or administrative/TA) and claim/TA	62
#3	(nation/TA or Japan/TA) and claim/TA and database/TA	86
#4	national database/AL or national claims database/AL	112
#5	(claims data/TA or specific health checkups/TA or specific health guidance/TA) and (database/TH or database/AL)	325
#6	#1 or #2 or #3 or #4 or #5	806
#7	JMDC/AL	51
#8	Japan Medical Data Center/AL or (Japan/TA and medical/TA and data/TA and center/TA)	70
#9	employ/TA and health/TA and insurance/TA	5
#10	#7 or #8 or #9	116
#11	MDV/AL and (data/TA or information/TA or (database/TH or database/AL))	18
#12	Medical Data Vision/AL or Medical・Data・Vision/AL	74
#13	#11 or #12	86
#14	KDB/AL or kokuho database/AL or (Kokuho/TA and database/TA)	73
#15	((public health insurance/TH or public health insurance/AL) or health insurance/TA or medical insurance/TA or community insurance/TA) and (database/TH or database/AL)	358
#16	(Kokuho/TA or Shaho/TA or Kempo/TA or Kenpo/TA) and (data/TA orinformation/TA or (database/TH or database/AL))	318
#17	#14 or #15 or #16	675
#18	((insurance claim/TH or claim/AL) or health insurance claim/AL or insurance claim/AL or claim/AL or (health insurance/TH or health insurance/AL)) and (data/TA or information/TA or (database/TH or database/AL))	2,262
#19	health/TA and (insurance/TA or administrative/TA) and claim/TA	46
#20	#18 or #19	2,276
#21	(DPC/TA or PDPS/TA or DRG/TA) and (data/TA or information/TA or (database/TH or database/AL))	2,432
#22	(Diagnosis Procedure Combination／Per-Diem Payment System/TH or Per-Diem Payment System/AL or Diagnosis Procedure Combination/AL or Diagnosis Group/AL or Disease by Diagnosis/AL) and (data/TA or information/TA or (database/TH or database/AL))	3,094
#23	(diagnos/TA and procedure/TA and combination/TA) or (diagnos/TA and related/TA and groups/TA)	432
#24	#21 or #22 or #23	3,576
#25	medical/TA and electronic/TA and claim/TA	10
#26	#6 or #10 or #13 or #17 or #20 or #24 or #25	6,403
#27	(#26) and (PT=Article)	1,609
#28	(#26) and (PT=Review)	83
#29	(#26) and (PT=Commentary)	1,598
#30	(#26) and (PT=Conference Abstract)	3,013
	※Search was conducted in Japanese (translated into English for submission).	

	MEDLINE	Results
1	NDB.mp. and (Japan$.mp. or Japan.in.)	53
2	(nation$ adj5 insurance adj2 claim?).mp. and (Japan$.mp. or Japan.in.)	106
3	1 or 2	131
4	(JMDC or (employ$ adj2 health adj1 insurance)).mp. and (Japan$.mp. or Japan.in.)	100
5	(Japan adj1 medical adj1 data adj1 center).mp. or (Japan adj1 medical adj1 data adj1 center).in.	109
6	4 or 5	181
7	(MDV or (medical adj1 data adj1 vision)).mp. and (Japan$.mp. or Japan.in.)	258
8	(medical adj1 data adj1 vision).in.	12
9	7 or 8	269
10	(KDB.mp. and (Japan$.mp. or Japan.in.)) or (Kokuho adj1 database?).mp.	10
11	((DPC and database?) or PDPS).mp. and (Japan$.mp. or Japan.in.)	114
12	(diagnos$ adj1 procedure$ adj1 combination).mp. and (Japan$.mp. or Japan.in.)	575
13	(diagno$ adj1 related adj1 groups).mp. and (Japan$.mp. or Japan.in.)	122
14	(per-diem adj1 payment adj1 system).mp. and (Japan$.mp. or Japan.in.)	21
15	diagnosis-related groups.sh. and (Japan$.mp. or Japan.in.)	116
16	11 or 12 or 13 or 14 or 15	686
17	((medical or health or healthcare or administrative or insurance) adj5 claim?).mp. and (Japan$.mp. or Japan.in.)	822
18	(claim? adj1 (data or database)).mp. and (Japan$.mp. or Japan.in.)	696
19	(administrative claims, healthcare or insurance claim reporting or insurance claim review).sh. and (Japan$.mp. or Japan.in.)	160
20	(nationwide adj1 database adj5 Japan$).mp.	84
21	17 or 18 or 19 or 20	1085
22	3 or 6 or 9 or 10 or 16 or 21	1987

	EMBASE	Results
#1	(ndb OR (nation* NEXT/5 insurance NEXT/2 claim*)) AND japan*	231
#2	(jmdc OR (japan NEXT/1 medical NEXT/1 data NEXT/1 center)) AND japan*	304

#3	(employ* NEXT/2 health NEXT/1 insurance) AND japan*	53
#4	#2 OR #3	350
#5	(mdv:ti,ab,kw OR (medical NEXT/1 data NEXT/1 vision)) AND japan*	340
#6	kdb AND japan* OR (kokuho NEXT/1 database*)	11
#7	(dpc AND database* OR pdps) AND japan*	277
#8	(diagnos* NEXT/1 procedure* NEXT/1 combination) AND japan*	807
#9	(diagnos* NEXT/1 related NEXT/1 group*) AND japan*	284
#10	'diagnosis related group'/exp AND japan*	255
#11	('per diem' NEXT/1 payment NEXT/1 system) AND japan*	30
#12	#7 OR #8 OR #9 OR #10 OR #11	1154
#13	((medical OR health OR healthcare OR administrative OR insurance) NEXT/5 claim*) AND japan*	1169
#14	(claim* NEAR/1 (data OR database*)) AND japan*	1258
#15	nationwide NEXT/1 database* NEAR/5 japan*	105
#16	'health insurance'/exp AND claim* AND japan*	661
#17	'billing and claims'/exp AND japan*	32
#18	#13 OR #14 OR #15 OR #16 OR #17	1860
#19	#1 OR #4 OR #5 OR #6 OR #12 OR #18	3238
#20	#19 AND ('Article'/it OR 'Article in Press'/it)	2069
#21	#19 AND 'review'/it	113
#22	#19 AND ('letter'/it OR 'note'/it OR 'short survey'/it)	32

Supplement file 2 PRISMA-ScR CHECKLIST

SECTION	ITEM	PRISMA-ScR CHECKLIST ITEM	REPORTED ON PAGE #
**TITLE**			
Title	1	Identify the report as a scoping review.	Title
**ABSTRACT**			
Structured summary	2	Provide a structured summary that includes (as applicable): background, objectives, eligibility criteria, sources of evidence, charting methods, results, and conclusions that relate to the review questions and objectives.	Abstract
**INTRODUCTION**			
Rationale	3	Describe the rationale for the review in the context of what is already known. Explain why the review questions/objectives lend themselves to a scoping review approach.	p. 4
Objectives	4	Provide an explicit statement of the questions and objectives being addressed with reference to their key elements (e.g., population or participants, concepts, and context) or other relevant key elements used to conceptualize the review questions and/or objectives.	p. 4
**METHODS**			
Protocol and registration	5	Indicate whether a review protocol exists; state if and where it can be accessed (e.g., a Web address); and if available, provide registration information, including the registration number.	NA
Eligibility criteria	6	Specify characteristics of the sources of evidence used as eligibility criteria (e.g., years considered, language, and publication status), and provide a rationale.	p. 4
Information sources*	7	Describe all information sources in the search (e.g., databases with dates of coverage and contact with authors to identify additional sources), as well as the date the most recent search was executed.	p. 5
Search	8	Present the full electronic search strategy for at least 1 database, including any limits used, such that it could be repeated.	Supplementary file 1
Selection of sources of evidence†	9	State the process for selecting sources of evidence (i.e., screening and eligibility) included in the scoping review.	p. 5
Data charting process‡	10	Describe the methods of charting data from the included sources of evidence (e.g., calibrated forms or forms that have been tested by the team before their use, and whether data charting was done independently or in duplicate) and any processes for obtaining and confirming data from investigators.	p. 6
Data items	11	List and define all variables for which data were sought and any assumptions and simplifications made.	p. 6
Critical appraisal of individual sources of evidence§	12	If done, provide a rationale for conducting a critical appraisal of included sources of evidence; describe the methods used and how this information was used in any data synthesis (if appropriate).	NA
Synthesis of results	13	Describe the methods of handling and summarizing the data that were charted.	p. 6
**RESULTS**			
Selection of sources of evidence	14	Give numbers of sources of evidence screened, assessed for eligibility, and included in the review, with reasons for exclusions at each stage, ideally using a flow diagram.	p. 6, Figure 1
Characteristics of sources of evidence	15	For each source of evidence, present characteristics for which data were charted and provide the citations.	p. 7, Table 1
Critical appraisal within sources of evidence	16	If done, present data on critical appraisal of included sources of evidence (see item 12).	NA
Results of individual sources of evidence	17	For each included source of evidence, present the relevant data that were charted that relate to the review questions and objectives.	Supplementary file 4
Synthesis of results	18	Summarize and/or present the charting results as they relate to the review questions and objectives.	pp. 8-, Table 2 and 3
**DISCUSSION**			
Summary of evidence	19	Summarize the main results (including an overview of concepts, themes, and types of evidence available), link to the review questions and objectives, and consider the relevance to key groups.	p. 12
Limitations	20	Discuss the limitations of the scoping review process.	p. 16
Conclusions	21	Provide a general interpretation of the results with respect to the review questions and objectives, as well as potential implications and/or next steps.	p. 17
**FUNDING**			
Funding	22	Describe sources of funding for the included sources of evidence, as well as sources of funding for the scoping review. Describe the role of the funders of the scoping review.	Title page

Supplement file 3 List of excluded studies with reasons

ID (Author Year)	Title (*titles translated by author)	Reason	Note
Abe 2011	Assessment of psychiatric outcomes in Japan based on diagnostic procedure combination information	Hospital-based (<10 institutes)	single-center
Akira 2019	Corrigendum to Treatment procedures and associated medical costs of methicillin-resistant Staphylococcus aureus infection in Japan: A retrospective analysis using a database of Japanese employment-based health insurance	Others (duplicate/ no original data used)	Correction
Arakaki 2012	Editorial comment to management trends, angioembolization performance and multiorgan injury indicators of renal trauma from Japanese administrative claims database	Others (duplicate/ no original data used)	Editorial Comment
Arakawa 2013	Economic evaluation for the prevention of cervical cancer by vaccination - From perspective of health insurance society and industry	Combined with primary research	(addition to data from JMDC's receipt database)
Asai 2019	Current Difficult Airway Management-Not Good Enough!	No health insurance claims data used	closed claims
Assimos 2012	Re: Renal haemorrhage risk after extracorporeal shockwave lithotripsy: results from the Japanese Diagnosis Procedure Combination database	Others (duplicate/ no original data used)	Editorial Comment
Babazono 2011	Do interventions to prevent lifestyle-related diseases reduce healthcare expenditures? A randomized controlled clinical trial	Combined with primary research	RCT
Besstremyannaya 2016	Differential Effects of Declining Rates in a Per Diem Payment System	Using published open data	DPC data (MHLW)
Chen 2016	The predictability of eye diseases for Alzheimer's disease	Not Japanese population	Taiwan
Chen 2020	Letter to the Editor Regarding "Treatment Risk for Elderly Patients with Unruptured Cerebral Aneurysm from a Nationwide Database in Japan"	Others (duplicate/ no original data used)	Letter to the Editor
Chen 2021	Characteristics of interstitial lung disease in patients from post-marketing data on metastatic breast cancer patients who received abemaciclib in Japan	Combined with primary research	questionnaires and/or interviews
Chida 2014	Implementation and evaluation of an efficient secure computation system using 'R' for healthcare statistics	Others (duplicate/ no original data used)	dummy insurance claim data (propose a secure statistical analysis system)
Crawford 2013	Real-world characterization of hypertension patients in japan: A 1-year overview	Others (duplicate/ no original data used)	
Disease 2018	Global, regional, and national incidence, prevalence, and years lived with disability for 354 diseases and injuries for 195 countries and territories, 1990-2017: a systematic analysis for the Global Burden of Disease Study 2017	Using published open data	(Global Burden of Disease Study)
Ebihara 2012	Impact of blunted perception of dyspnea on medical care use and expenditure, and mortality in elderly people	Combined with primary research	Tsurugaya project, a population-based longitudinal study
Enger 2010	Comorbidities in patients with persistent or chronic immune thrombocytopenia	Not Japanese population	
Fujieda 2019	The effects of the medical economics of prescription inquiries made by pharmacy pharmacists	No health insurance claims data used	prescription inquiries
Fujii 2016	Infectious disease during hospitalization is the major causative factor for prolonged hospitalization: multivariate analysis of diagnosis procedure combination (DPC) data of 20,876 cases in Japan	Hospital-based (<10 institutes)	single-center
Fujii 2019	Impact of Body Mass Index of Japanese Gallbladder Cancer Patients on their Postoperative Outcomes	Hospital-based (<10 institutes)	National Saiseikai Hospital DPC survey
Fujita 2019	Secondhand Smoke and Streptococcal Infection in Young Children Under Japan's Voluntary Tobacco-Free Policy	Others (duplicate/ no original data used)	Not available
Fukai 2020	Validation of self-reported medication use for hypertension, diabetes, and dyslipidemia among employees of large-sized companies in Japan	Combined with primary research	web- or paper-based questionnaires
Fukuda 2014	Medical Expenditures Associated With Type 2 Diabetes Mellitus in Japan: A Large Claims Database Study	Others (duplicate/ no original data used)	
Fukuhara 2015	Geographical analysis of aneurysmal subarachnoid hemorrhage in Japan utilizing publically-accessible DPC database	Using published open data	DPC data (MHLW)
Fukuhara 2016	Prefectural difference in spontaneous intracerebral hemorrhage incidence in Japan analyzed with publically accessible diagnosis procedure combination data: possibilities and limitations	Using published open data	DPC data (MHLW)
Graham 2020	Artificial intelligence approaches to predicting and detecting cognitive decline in older adults: A conceptual review	Review article	A Conceptual Review
Grosse 2021	Identification of congenital CMV cases in administrative databases and implications for monitoring prevalence, healthcare utilization, and costs	Review article	review study
Hamada 2012	Effects of the per diem prospective payment system with DRG-like grouping system (DPC/PDPS) on resource usage and healthcare quality in Japan	Others (duplicate/ no original data used)	Thesis
Hamamoto 2019	Editorial Comment from Dr Hamamoto to Endurological treatment trend of upper urinary urolithiasis in Japan from the Japanese Diagnosis Procedure Combination Database	Others (duplicate/ no original data used)	Editorial Comment
Hamaya 2021	Effects of an mHealth App (Kencom) With Integrated Functions for Healthy Lifestyles on Physical Activity Levels and Cardiovascular Risk Biomarkers: Observational Study of 12,602 Users	Combined with primary research	Mobile health (mHealth) apps
Han 2019	Increased risk of psoriasis in subjects with abdominal obesity: A nationwide population-based study	Not Japanese population	Korean National Health Insurance Service (NHIS) database
Hashimoto 2012	Gains in Disability-Free Life Expectancy From Elimination of Diseases and Injuries in Japan	No health insurance claims data used	Comprehensive Survey of Living Conditions of the People on Health and Welfare
Hayashi 2017	[Expectations of Certified Psychologist in the Clinical Medicine System in Germany for Future Reference]	No health insurance claims data used	
Hayashi 2020	Associations between the injustice experience questionnaire and treatment term in	No health insurance claims data	
Hayashida 2013	Situation Analysis of Emergency Department of the Japanese Acute Care Hospitals	Combined with primary research	additional research for emergency care
Hayashida 2021	History and Profile of Diagnosis Procedure Combination (DPC): Development of a Real Data Collection System for Acute Inpatient Care in Japan	Review article	Review
Hern 2015	US hospital payment adjustments for innovative technology lag behind those in Germany, France, and Japan	No health insurance claims data used	payment systems in US, Germany, France, and Japan
Higaonna 2019	Effect of the Partnership Nursing Model on In-hospital Mortality in Japan	Using published open data	DPC data (MHLW)

Higaonna 2020	Association between nursing care delivery models and patients' health outcomes in a university hospital: A retrospective cohort study based on the Diagnostic Procedure Combination database	Hospital-based (<10 institutes)	an academic teaching hospital
Higashi 2015	Measuring the quality of care using a large clinical database: Lessons learned in oncology	Review article	Review
Hiraoka 2019	Prediction of Prognosis of Intermediate-Stage HCC Patients: Validation of the Tumor Marker Score in a Nationwide Database in Japan	No health insurance claims data used	a nationwide survey system
Hirose 2020	A review of studies using the Japanese National Database of Health Insurance Claims and Specific Health Checkups	Review article	review study
Hishida 2016	Long-term outcome and prognostic factors of surgically treated thymic carcinoma: results of 306 cases from a Japanese Nationwide Database Study	No health insurance claims data used	a nationwide database: The Japanese Association for Research of the Thymus(JART)
Hung 2018	Diagnostic consistency and interchangeability of schizophrenic disorders and bipolar disorders: A 7-year follow-up study	Not Japanese population	Taiwan
Ie 2017	Potentially Harmful Medication Use and Decline in Health-Related Quality of Life among Community-Dwelling Older Adults	Not Japanese population	
Igarashi 2019	Cost-minimisation model of magnetic resonance-guided focussed ultrasound therapy compared to unilateral deep brain stimulation for essential tremor treatment in Japan	Using published open data	data from published sources and expert clinical opinion
Iihara 2013	Issues in acute stroke care systems in Japan with a special emphasis on implementation of comprehensive stroke centers	Review article	Review
Ikawa 2020	In Reply to Letter to the Editor Regarding "Treatment Risk for Elderly Patients with Unruptured Cerebral Aneurysm from a Nationwide Database in Japan"	Others (duplicate/ no original data used)	Letter
Inagaki 2014	[Metabolic and lithium monitoring in Japanese psychiatric outpatient clinics]	Duplicates	Duplicate (稲垣 2014)
Inoue 2016	[Psychotropic Prescribing Practices for Children and Adolescents with Intellectual Disabilities: A Cohort Study Using a Large-scale Health Insurance Database]	Duplicates	Duplicate (井上 2016)
Inoue 2017	Regional Variation in the Use of Percutaneous Coronary Intervention in Japan	Using published open data	DPC summary tables (MHLW)
Inoue 2019	[Factors related to life expectancy in prefectures: An ecological study using the National Database]	No health insurance claims data used	Health checkup data
Inoue 2019	Regional variance in patterns of prescriptions for chronic kidney disease in Japan	Using published open data	NDB Open data
Inoue 2021	Regional variation in the use of catheter ablation for patients with arrhythmia in Japan	Using published open data	DPC data (MHLW)
Ishii 2012	DRG/PPS and DPC/PDPS as Prospective Payment Systems	Review article	Review
Ishikawa 2016	Medical Big Data for Research Use: Current Status and Related Issues	Review article	Conferences and Lectures
Ishimaru 2019	Domiciliary dental care among homebound older adults: A nested case-control study in Japan	No health insurance claims data used	LTC insurance claims database and the Survey of Medical Institutions in Japan
Isobe 2020	Correlation between the number of laparoscopy-qualified gynecologists and the proportion of laparoscopic surgeries for benign gynecological diseases in Japan: An ecological study	Using published open data	NDB Open data
Isobe 2020	The number of overall hysterectomies per population with the perimenopausal status is increasing in Japan: A national representative cohort study	Using published open data	NDB Open data
Isumi 2020	Assessment of Additional Medical Costs Among Older Adults in Japan With a History of Childhood Maltreatment	Combined with primary research	the Japan Gerontological Evaluation Study (JAGES)
Itoh 2019	National burden of the pharmaceutical cost of wet compresses and its cost predictors: nationwide cross-sectional study in Japan	Using published open data	NDB Open data
Iwasaki 2017	Association between tooth loss and medical costs related to stroke in healthy older adults aged over 75 years in Japan	Combined with primary research	Niigata Study (a prospective community-based study)
Jamotte 2014	Treatment Patterns and Health Care Costs in Patients with Schizophrenia Initiating with First- or Second-Generation Antipsychotic: Results from a Japanese Claims Database Analysis	Others (duplicate/ no original data used)	
Jamotte 2014	Treatment Patterns and Health Care Costs in Patients with Depression Treated with Antidepressant only or Combined with Benzodiazepine: Results From a Japanese Claims Database Analysis	Others (duplicate/ no original data used)	
Jingushi 2021	Low-intensity pulsed ultrasound is frequently used to treat fractures after osteosynthesis in elderly patients: A study using open data from the national database of health insurance claims of Japan	Using published open data	NDB Open data
Kamata 2018	Wide difference in biologics usage and expenditure for the treatment of patients with rheumatoid arthritis in each prefecture in Japan analyzed using "National Database of Health Insurance Claims and Specific Health Checkups of Japan"	Using published open data	NDB Open data
Kamata 2019	Status quo of osteoporosis treatment in Japan disclosed by the National Database of Health Insurance Claims and Specific Health Checkups: too late in treatment initiation and too few in treated patients?	Using published open data	NDB Open data
Kamijo 2014	Cost information of chemotherapy for cervical and endometrial cancer in Japan	Hospital-based (<10 institutes)	a specialized cancer hospital
Kaneko 2020	Medical and Dental Visits of Chronic Kidney Disease-Diagnosed Participants Analyzed From the Specific Health Checkups Results in Japan: TAMA MED Project-CKD	Combined with primary research	questionnaire (TAMA MED Project-CKD)
Kanzaki 2020	Improvement of diagnostic accuracy of Parkinson's disease on I-123-ioflupane single photon emission computed tomography (123I FP-CIT SPECT) using new Japanese normal database	No health insurance claims data used	Japanese normal database (NDB)
Karimian 2020	A commentary on the article: Comparison of outcomes after differentiated thyroid cancer surgery performed with and without energy devices: A population-based cohort study using a nationwide database in Japan, Int. J. Surg. 2020 Apr 9, doi: 10.1016/j.ijsu.2020.03.072, pii: S1743-9191(20)30286-7	Others (duplicate/ no original data used)	Invited Commentary
Kashiwagi 2013	Factors associated with the use of home-visit nursing services covered by the long-term care insurance in rural Japan: a cross-sectional study	No health insurance claims data used	long-term care insurance
Katano 2018	Trends in isolated meniscus repair and meniscectomy in Japan, 2011-2016	Using published open data	NDB Open data
Katano 2020	Trends in arthroplasty in Japan by a complete survey, 2014-2017	Using published open data	NDB Open data
Kataoka 2019	Scholarly Activity Support Systems in Internal Medicine Residency Programs: A National Representative Survey in Japan	Combined with primary research	web questionnaire
Kato 2016	Consequences of an Early PSA Response to Enzalutamide Treatment for Japanese Patients with Metastatic Castration-resistant Prostate Cancer	No health insurance claims data used	

Kato 2017	Comparative economic evaluation of home-based and hospital-based palliative care for terminal cancer patients	Hospital-based (<10 institutes)	Nikko Memorial Hospital and Motowanishi Family Clinic in Muroran city, Hokkaido, Japan
Kato 2017	History of the secondary use of national database of health insurance claims and specific health checkups of Japan(NDB)	Review article	Review
Kawahara 2020	A high neutrophil-to-lymphocyte ratio is a poor prognostic factor for castration-resistant prostate cancer patients who undergo abiraterone acetate or enzalutamide treatment	No health insurance claims data used	
Kimura 2011	Pharmacovigilance systems and databases in Korea, Japan, and Taiwan	Review article	Review
Kimura 2020	Cardiovascular and cerebrovascular diseases risk associated with the incidence of presenteeism and the costs of presenteeism	Combined with primary research	self-administered questionnaire about presenteeism and absenteeism
Kinosada 2016	[Utilization of Big Data in Medicine and Future Outlook]	Using published open data	DPC data (MHLW)
Kinoshita 2014	A Retrospective, Hospital-Based Study to Determine the Incidence of Rotavirus Hospitalizations among Children Less than 5 Years of Age over a 10-Year Period(2001-2011) in Akita Prefecture, Japan	Hospital-based (<10 institutes)	Yuri-Kumiai General Hospital
Kinoshita 2021	Cost-effectiveness of a hybrid emergency room system for severe trauma: a health technology assessment from the perspective of the third-party payer in Japan	Hospital-based (<10 institutes)	a tertiary care hospital
Kobayashi 2020	Sequential Use of Androgen Receptor Axis-targeted Agents in Chemotherapy-naive Castration-resistant Prostate Cancer: A Multicenter Retrospective Analysis With 3-Year Follow-up	No health insurance claims data used	
Kodama 2019	Construction of a Heart Failure Database Collating Administrative Claims Data and Electronic Medical Record Data to Evaluate Risk Factors for In-Hospital Death and Prolonged Hospitalization	Hospital-based (<10 institutes)	3 hospitals
Kohsaka 2021	Current trends in diabetes mellitus database research in Japan	Review article	Review
Kon 2018	Association between renal function and cardiovascular and all-cause mortality in the community-based elderly population: results from the Specific Health Check and Guidance Program in Japan	No health insurance claims data used	Specific Health Check and Guidance Program
Konno 2015	Moderately increased albuminuria is an independent risk factor of cardiovascular events in the general Japanese population under 75 years of age: the Watari study	Combined with primary research	Watari study (a community-based prospective cohort study)
Konta 2020	Association between serum uric acid levels and mortality: a nationwide community-based cohort study	No health insurance claims data used	Specific Health Checkup
Koram 2019	Validation studies of claims data in the Asia-Pacific region: A comprehensive review	Review article	review study
Koyama 2017	Patterns of CT use in Japan, 2014: A nationwide cross-sectional study	Using published open data	NDB Open data
Koyama 2018	Trends in the medication reviews of community pharmacies in Japan: a nationwide retrospective study	Using published open data	Survey of medical care activities in public health insurance (MHLW)
Kubo 2011	[Practical use of DPC information for occupational health consultation]	Using published open data	DPC data (MHLW)
Kumamaru 2018	Global and Japanese regional variations in radiologist potential workload for computed tomography and magnetic resonance imaging examinations	Using published open data	NDB Open data
Kuniyoshi 2021	Regional variation in the development of neonatal hyperbilirubinemia and relation with sunshine duration in Japan: an ecological study	Using published open data	NDB Open data
Kurasawa 2020	Pneumonia prevention effects of perioperative oral management in approximately 25,000 patients following cancer surgery	Hospital-based (<10 institutes)	eight regional central hospitals
Kurita 2017	A phase I/II trial of intrabone marrow cord blood transplantation and comparison of the hematological recovery with the Japanese nationwide database	No health insurance claims data used	a phase I/II prospective single-center trial
Kurita 2018	Association among(Nursery) School Absenteeism Surveillance System and Incidence of Infectious Diseases	Using published open data	used published NDB data
Kuriyama 2014	[Analysis of cancer treatment in Iwate Prefecture - an analysis of health care regions based on MHLW DPC data]	Using published open data	DPC data (MHLW)
Kuroiwa 2010	[Economical basis for neurologic services and the system for health insurance: diagnosis procedure combinations and insurance scores of neurophysiological tests]	Review article	Symposium Review
Kurosaki 2020	[Medical expenses for diabetes care in Japan: Analysis of inter-prefecture differences]	Using published open data	NDB Open data
Kusama 2020	Obstacles to antimicrobial use surveillance using claims data in elderly care facilities in Japan	Review article	Review
Kuwabara 2016	Behavior modification stages of attendance at specific health check-ups and relevant knowledge, attitudes, and behaviors in one municipal insurance representation	No health insurance claims data used	self-administered questionnaire
Lai 2015	Databases in the Asia-pacific region: The potential for a distributed network approach	Review article	characteristics of databases in Asian-pacific countries
Lin 2017	Survival analysis of increases in care needs associated with dementia and living alone among older long-term care service users in Japan	No health insurance claims data used	LTC insurance claims dataset
Liu 2010	Use of benzodiazepines, hypnotics, and anxiolytics in major depressive disorder: Association with chronic pain diseases	Not Japanese population	US
Liu 2021	Effect of Care Rehabilitation on Medical Expenses, Care Costs, and Total Costs of Elderly Individuals with Parkinson's Disease	Others (duplicate/ no original data used)	Not available
Lkhagva 2013	Does co-payment rate influence the relationship between monthly salary and health care service demand among the insured of health insurance societies in Japan?	Using published open data	National Federation of Health Insurance Societies; Annual Report
Matsuda 2016	Hospitals and Big Data: Use of Case Mix and E-claim Data in Japan	Review article	Review
Matsuda 2016	Development of Case Mix Based Evaluation System in Japan	Review article	Review
Matsuda 2019	Health Policy in Japan - Current Situation and Future Challenges	Review article	Review
Matsumoto 2013	DPC in acute-phase inpatient hospital care. Visualization of amount of nursing care provided and accessibility to nursing care	Hospital-based (<10 institutes)	a university hospital
Matsumoto 2020	Validity Assessment of Self-reported Medication Use for Hypertension, Diabetes, and Dyslipidemia in a Pharmacoepidemiologic Study by Comparison With Health Insurance Claims	Combined with primary research	Tsuruoka Metabolomics Cohort Study
Matsutani 2018	Pregabalin as an analgesic option for patients undergoing thoracotomy: cost analysis of pregabalin versus epidural analgesia for post-thoracotomy pain relief	Hospital-based (<10 institutes)	Teikyo University Hospital (RCT)
Mihara 2020	Factors correlated with drug use for constipation: perspectives from the 2016 open Japanese National Database	Using published open data	NDB Open data
Milea 2015	A review of accessibility of administrative healthcare databases in the Asia-Pacific region	Review article	review study

Mitsutake 2021	Influence of co-existing social isolation and homebound status on medical care utilization and expenditure among older adults in Japan	Combined with primary research	(+Postal surveys on social isolation and homebound status)
Miyamoto 2017	Integration of DPC and clinical microbiological data in Japan reveals importance of confirming a negative follow-up blood culture in patients with MRSA bacteremia	Hospital-based (<10 institutes)	Kurume University Hospital
Miyamoto 2021	Effect of tranexamic acid in patients with colonic diverticular bleeding: A nationwide inpatient database study	Duplicates	Duplicate (Miyamoto 2020)
Miyazaki 2021	Correction to: Real-World Prescription Pattern and Healthcare Cost Among Patients with Ulcerative Colitis in Japan: A Retrospective Claims Data Analysis	Others (duplicate/ no original data used)	Correction
Mizuno 2015	Surgical management of recurrent thymic epithelial tumors: a retrospective analysis based on the Japanese nationwide database	No health insurance claims data used	nationwide registry study of thymic epithelial tumors
Momo 2021	Development and application for drug-safety and efficacy using a large claims data	Review article	Symposium Review
Mori 2019	Correction to: Estimated expenditures for hip fractures using merged healthcare insurance data for individuals aged >= 75 years and long-term care insurance claims data in Japan	Others (duplicate/ no original data used)	Correction
Mori 2019	Influence of seasonal changes on disease activity and distribution of affected joints in rheumatoid arthritis	No health insurance claims data used	a nationwide RA database
Mori 2020	The Impact of Diabetes Status on Presenteeism in Japan	Combined with primary research	questionnaire survey
Morii 2019	Projecting future supply and demand for physical therapists in Japan using system dynamics	Using published open data	NDB Open data
Morimae 2012	Cost disparity between open repair and endovascular aneurysm repair for abdominal aortic aneurysm: a single-institute experience in Japan	Hospital-based (<10 institutes)	Single-center
Morishima 2013	Burden of household environmental tobacco smoke on medical expenditure for Japanese women: a population-based cohort study	Combined with primary research	Ohsaki Cohort Study
Morishima 2019	Impact of Comorbidities on Survival in Gastric, Colorectal, and Lung Cancer Patients	Hospital-based (<10 institutes)	at the five subject hospitals
Morita 2018	Factors Affecting Discharge to Home of Geriatric Intermediate Care Facility Residents in Japan	No health insurance claims data used	National LTC Insurance Claim Database
Morita 2019	Authors' Reply to Cerejeira: "Association of Early Systemic Corticosteroid Therapy with Mortality in Patients with Stevens-Johnson Syndrome or Toxic Epidermal Necrolysis: A Retrospective Cohort Study Using a Nationwide Claims Database"	Others (duplicate/ no original data used)	Authors' Reply
Mukai 2020	Trends Associated with Hemorrhoids in Japan: Data Mining of Medical Information Datasets and the National Database of Health Insurance Claims and Specific Health Checkups of Japan (NDB) Open Data Japan	Using published open data	NDB Open data
Murakami 2015	Ecological Analysis of Factors Associated with Mortality of Cerebral Infarction in Japan	Using published open data	opened results of first research attempt to apply the NDB for planning of RHCP
Murata 2012	[Preoperative antimicrobial therapy for patients with cholecystectomy for acute cholecystitis based on the administrative database associated with the Diagnosis Procedure Combination system]	Duplicates	Duplicate (村田2012)
Murata 2014	Claims Database Analysis Of Patients With Chronic Hepatitis C In Japan	Others (duplicate/ no original data used)	
Murata 2015	Circumstance of endoscopic and laparoscopic treatments for gastric cancer in Japan: A review of epidemiological studies using a national administrative database	Review article	A review
Mushiroda 2018	Association of HLA-A*31:01 Screening With the Incidence of Carbamazepine-Induced Cutaneous Adverse Reactions in a Japanese Population	Combined with primary research	genetic screening
Nagai 2020	Data resource profile: JMDC claims databases sourced from Medical Institutions	Review article	Data resource profile
Nagakura 2021	The Significant Association between Health Examination Results and Population Health: A Cross-Sectional Ecological Study Using a Nation-Wide Health Checkup Database in Japan	No health insurance claims data used	Health Checkup Database
Nagata 2018	Total Health-Related Costs Due to Absenteeism, Presenteeism, and Medical and Pharmaceutical Expenses in Japanese Employers	Combined with primary research	self-administered questionnaire
Nagata 2020	Work functioning impairment in the course of pharmacotherapy treatment for depression	Combined with primary research	using a questionnaire
Nakagawa 2011	A new accounting system for financial balance based on personnel cost after the introduction of a DPC/DRG system	Hospital-based (<10 institutes)	Single-center
Nakagoe 2015	Surgical risk model for acute diffuse peritonitis based on a Japanese nationwide database: an initial report on the surgical and 30-day mortality	No health insurance claims data used	National Clinical Database
Nakagoe 2015	Erratum to: Surgical risk model for acute diffuse peritonitis based on a Japanese nationwide database: an initial report on the surgical and 30-day mortality	Others (duplicate/ no original data used)	NCD, erratum
Nakai 2018	Response to flutamide, as second-line therapy after bicalutamide, predicts efficacy of abiraterone, not that of enzalutamide	No health insurance claims data used	at Nara Medical University Hospital
Nakajima 2015	[Examination of the Difference in Medical Treatment Contents According to Major Diagnostic Category of Hospital Group I and Group II Using the Diagnosis Procedure Combination Survey Data]	Using published open data	DPC data (MHLW)
Nakajima 2015	Examination of diagnosis procedure combination survey data that influence function evaluation coefficient II	Using published open data	DPC data (MHLW)
Nakajima 2016	Type Classification Based on Inpatient Ratio According to Major Diagnosis Category of Group III in the Diagnosis Procedure Combination System	Using published open data	DPC data (MHLW)
Nakajima 2017	Examination of Factors that Influence the Operation Income and Expenditure Balance Difference Rate of 20 Educational Foundation Universities	Using published open data	DPC data (MHLW)
Nakakita 2021	Standard table direct anterior approach within an early discharge protocol for cementless total hip arthroplasty: experience from a Japanese hospital	Hospital-based (<10 institutes)	single-center
Nakamura 2016	Diagnosis Procedure Combination Database Would Develop Nationwide Clinical Research in Japan	Review article	Review
Nakao 2020	Trends and outcomes of blunt renal trauma management: a nationwide cohort study in Japan	No health insurance claims data used	the Japan Trauma Data Bank (JTDB)
Nakashima 2021	Impact of Enteral Nutrition Within 24 Hours Versus Between 24 and 48 Hours in Patients With Severe Acute Pancreatitis: A Multicenter Retrospective Study	No health insurance claims data used	a multi-center cohort study
Nakayama 2012	[Approaching the "evidence-practice gap" in pharmaceutical risk management: analysis of healthcare claim data]	Review article	Review

Nakayama 2017	Analysis of the evidence-practice gap to facilitate proper medical care for the elderly: investigation, using databases, of utilization measures for National Database of Health Insurance Claims and Specific Health Checkups of Japan (NDB)	Others (duplicate/ no original data used)	study protocol
Nishi 2016	Effects of Eating Fast and Eating Before Bedtime on the Development of Nonalcoholic Fatty Liver Disease	Others (duplicate/ no original data used)	Not available
Nishi 2020	Regional variance in the use of urine dipstick test for outpatients in Japan	Using published open data	NDB Open data
Nishigori 2015	[Review of the health technology assessment on surgeries in Japan]	Review article	Review
Nishikawa 2021	Prognostic Effect of Incidental Pulmonary Embolism on Long-Term Mortality in Cancer Patients	Hospital-based (<10 institutes)	single-center
Nishimura 2015	Big data visualization of acute stroke care practices using a nationwide neurosurgeon survey	Review article	Review
Nishimura 2016	Status and Future Perspectives of Utilizing Big Data in Neurosurgical and Stroke Research	Review article	Review
Nishimura 2020	Role of comprehensive stroke centers in stroke care	Review article	Review
Niu 2012	C-reactive protein (CRP) is a predictor of high medical-care expenditures in a community-based elderly population aged 70 years and over: The Tsurugaya project	Combined with primary research	Tsurugaya project, a population-based longitudinal study
Noguchi 2020	Japan Trial in High-Risk Individuals to Enhance Their Referral to Physicians (J-HARP)-A Nurse-Led, Community-Based Prevention Program of Lifestyle-Related Disease	Combined with primary research	a clustered randomized controlled trial
Nonaka 2018	Incidence of aspiration pneumonia during hospitalization in Japanese hospitalized cases did not increase whereas concern factors were exacerbated in a time-dependent manner: analysis of Diagnosis Procedure Combination (DPC) data	Hospital-based (<10 institutes)	Saga University Hospital
Nonaka 2018	A Cost-benefit Analysis of Percutaneous Coronary Intervention and Coronary Artery Bypass Grafting using Reimbursement Data of Japan: A Single-center Pilot Study	Hospital-based (<10 institutes)	Saga University Hospital
Nordstrom 2020	Incidence of venous thromboembolism among postmenopausal women prescribed ospemifene, selective estrogen receptor modulators for noncancer indications, or untreated vulvar and vaginal atrophy	Not Japanese population	
Obara 2021	[Study on the Safety of Perinatal Medication]	Review article	Review
Obata 2020	The clinical usage of liposomal amphotericin B in patients receiving renal replacement therapy in Japan: a nationwide observational study	Duplicates	Duplicate (Obata 2021)
Okada 2021	Big data and real-world data-based medicine in the management of hypertension	Review article	Review
Okamoto 2010	Declining accuracy in disease classification on health insurance claims: should we reconsider classification by principal diagnosis?	Using published open data	National Health Insurance Medical Benefit Survey (NHIMBS)
Okamoto 2013	Qualitative evaluation of the supporting system for diagnosis procedure combination code selection	No health insurance claims data used	Qualitative Evaluation
Okamoto 2014	Linkage rate between data from health checks and health insurance claims in the Japan National Database	Using published open data	Four publicly available datasets
Okawara 2020	Association between the course of hypnotics treatment for insomnia and work functioning impairment in Japanese workers	Combined with primary research	a questionnaire survey
Okui 2021	An Age-Period-Cohort Analysis of Prevalence and Consultation Rate for Dyslipidemia in Japan	Using published open data	NDB Open data
Okumura 2014	[Trends of psychotropic medication use among children and adolescents in Japan data from the national insurance claims database between 2002 and 2010]	Duplicates	Duplicate (奥村 2014)
Okumura 2015	Trends in use of psychotropic medications among patients treated with cholinesterase inhibitors in Japan from 2002 to 2010	Using published open data	Survey of Medical Care Activities in Public Health Insurance
Ono 2019	Impact of care coordination on oral anticoagulant therapy among patients with atrial fibrillation in routine clinical practice in Japan: a prospective, observational study	Combined with primary research	a multi-center, single-arm, prospective cohort study
Onoue 2018	Trends in Polypharmacy in Japan: A Nationwide Retrospective Study	Using published open data	Survey of Medical Care Activities in Public Health Insurance
Otaki 2017	Analysis of Closed Claims in the Clinical Management of Rheumatoid Arthritis in Japan	No health insurance claims data used	Closed claims
Otsuki 2021	Characteristics and Burden of Diagnostic Error-Related Malpractice Claims in Neurosurgery	No health insurance claims data used	malpractice claims database
Ozaki 2019	Quality of primary care provided in community clinics in Japan	No health insurance claims data used	medical chart review
Ryu 2011	The benchmark analysis of gastric, colorectal and rectal cancer pathways: toward establishing standardized clinical pathway in the cancer care	Hospital-based (<10 institutes)	five participating cancer centers
Sado 2016	Socio-environmental factors associated with diabetes mellitus among patients hospitalized with schizophrenia in Japan	Hospital-based (<10 institutes)	single-center
Saito 2010	Copayment level and drug switching: Findings for type 2 diabetes	Not Japanese population	
Saito 2011	Hospice care and survival among elderly patients with lung cancer	Not Japanese population	
Sakai 2013	[DPC data based situation analysis of Regional Core Hospital: an application of the GIS Methodology for Regional Health Care Plans]	Hospital-based (<10 institutes)	Single-center
Sakai 2013	[Evaluation of the application of the clinical pathway using Diagnosis Procedure Combination (DPC) data]	Hospital-based (<10 institutes)	single-center
Sakamoto 2019	Editorial Comment from Dr Sakamoto to Endurological treatment trend of upper urinary urolithiasis in Japan from the Japanese Diagnosis Procedure Combination Database	Others (duplicate/ no original data used)	Editorial comment
Sakata 2020	A Study on the Status of Proton Pump Inhibitor Prescriptions Using Diagnosis Procedure Combination Data in Japan	Hospital-based (<10 institutes)	single-center
Saokaew 2015	Healthcare databases in Thailand and Japan: Potential sources for health technology assessment research	Review article	review study
Sasaki 2019	Does hospital information technology infrastructure promote the implementation of clinical practice guidelines? A multicentre observational study of Japanese hospitals	Combined with primary research	questionnaire survey
Sato 2015	Significance of estimated glomerular filtration rate in predicting brain or heart attacks in obese and non-obese populations	No health insurance claims data used	Specific Health Checkup database
Sato 2016	Association between periodontitis and medical expenditure in older adults: A 33-month follow-up study	Combined with primary research	Niigata Study (a prospective community-based study)
Sato 2018	Estimation of total prescription weights of active pharmaceutical ingredients in human medicines based on a public database for environmental risk assessment in Japan	Using published open data	NDB Open data

Sato 2019	Novel Analytics Framework for Universal Healthcare Insurance Database	Using published open data	based on the results of previous analyses
Sato 2019	Novel Analytics Framework for Universal Healthcare Insurance Claims Database	No health insurance claims data used	Case Study
Sato 2021	Disproportionality by sex in the prescription of drugs capable of inducing parkinsonism for the elderly: A survey using statistics of Japanese national health claims from 2014 to 2017	Using published open data	NDB Open data
Sekimoto 2010	Risk-adjusted assessment of incidence and quantity of blood use in acute-care hospitals in Japan: an analysis using administrative data	Hospital-based (<10 institutes)	two hospitals
Shibahashi 2021	Derivation and validation of a quantitative screening model for blunt cerebrovascular injury	No health insurance claims data used	Japan Trauma database
Shibata 2019	Correction to: Influenza vaccination effectiveness for people aged under 65 years in Japan, 2013/ 2014 season: application of a doubly robust method to a large-scale, real-world dataset	Others (duplicate/ no original data used)	Correction
Shida 2010	[Image examination of malignant tumors in lungs observed on the basis of cost analysis using diagnosis procedure combination data]	Hospital-based (<10 institutes)	Single-center
Shikamura 2012	[Medical economics research on awareness of community pharmacists about raising pharmaceutical questions regarding prescriptions issued by physicians]	Combined with primary research	surveying the actual condition of pharmaceutical inquiries
Shimazaki 2020	Association between dental consultation and oral health status among male Japanese employees	Others (duplicate/ no original data used)	a company
Shiratori 2021	Association between the chronic use of gastric acid suppressants and high-risk colorectal polyps	Hospital-based (<10 institutes)	nine high-volume hospitals
Shiroiwa 2014	Treatment Costs for Breast Cancer in Japan: Large Claim Database Analysis	Others (duplicate/ no original data used)	Abstract
Shiroiwa 2017	Cost-effectiveness analysis of the introduction of S-1 therapy for first-line metastatic breast cancer treatment in Japan: results from the randomized phase III SELECT BC trial	Combined with primary research	(expected cost was calculated using patient-level claim data)
Shoji 2011	Assessing a Clinical Pathway to Improve the Quality of Care in Pulmonary Resections	Hospital-based (<10 institutes)	single-center
Sugawara 2020	Association of moderately abnormal behavior and administered neuraminidase inhibitors	Using published open data	NDB Open data
Sugihara 2019	Regional clinical practice variation in urology: Usage example of the Open Data of the National Database of Health Insurance Claims and Specific Health Checkups of Japan	Using published open data	NDB Open data
Sugisaki 2018	Correction to: A case-control study of the risk of upper gastrointestinal mucosal injuries in patients prescribed concurrent NSAIDs and antithrombotic drugs based on data from the Japanese national claims database of 13 million accumulated patients	Others (duplicate/ no original data used)	correction
Suzuki 2010	Auto-selection of DRG codes from discharge summaries by text mining in several hospitals: analysis of difference of discharge summaries	Hospital-based (<10 institutes)	three Hospitals
Takaku 2017	Do benefits in kind or refunds affect health service utilization and health outcomes? A natural experiment from Japan	No health insurance claims data used	
Takatori 2019	Impaired activity of daily living is a risk factor for high medical cost in patients of non-variceal upper gastrointestinal bleeding	Hospital-based (<10 institutes)	Single-center
Takayama 2017	A Cost-Utility Analysis of Endovascular Aneurysm Repair for Abdominal Aortic Aneurysm	Hospital-based (<10 institutes)	Ibaraki Prefectural Central Hospital
Takeda 2017	The current situation and issues of the secondary use of electronic medical record data	No health insurance claims data used	electronic medical record data
Takeda 2020	Correction to: Prophylactic sivelestat for esophagectomy and in-hospital mortality: a propensity score-matched analysis of claims database	Others (duplicate/ no original data used)	Correction
Takeuchi 2014	A risk model for esophagectomy using data of 5354 patients included in a Japanese nationwide web-based database	No health insurance claims data used	National Clinical Database (NCD)
Takeuchi 2017	Comparison of Short-Term Outcomes Between Open and Minimally Invasive Esophagectomy for Esophageal Cancer Using a Nationwide Database in Japan	No health insurance claims data used	NCD
Takeuchi 2018	Association between Hemoglobin and Hemoglobin A1c: A Data-Driven Analysis of Health Checkup Data in Japan	No health insurance claims data used	Health Checkup Data
Takura 2010	[Study on medical economic evaluation methods for metastatic brain tumors therapy]	Combined with primary research	QOL assessment (EQ-5D, SF-36)
Takura 2019	Health Economics - Effect of Electronic Medical Record Systems on Cardiovascular Disease Outpatient Consultation Time	Combined with primary research	Electronic Medical Record Systems
Takusari 2021	Trends in Hip Fracture Incidence in Japan: Estimates Based on Nationwide Hip Fracture Surveys From 1992 to 2017	No health insurance claims data used	Nationwide Hip Fracture Surveys
Tang 2019	The evaluation of the economic burden caused by delay in the diagnosis and treatment for patients with diabetes mellitus based on Japanese medical big data	Others (duplicate/ no original data used)	Chinese
Tasaki 2012	Visualization Model for Medical Care Processes by Utilizing Japanese Case-mix Classification and its Application to the Variance Analysis of Clinical Pathway	Hospital-based (<10 institutes)	Saiseikai Kumamoto Hospital
Terahara 2019	Carbapenem-resistant Pseudomonas aeruginosa and carbapenem use in Japan: an ecological study	Using published open data	The AMR Clinical Reference Centre of National Centre for Global Health and Medicine
Tochitani 2020	Longitudinal association between mental health and future antibiotic prescriptions in healthy adults: Results from the LOHAS	Combined with primary research	prospective cohort study
Tokuda 2011	Cognitive error as the most frequent contributory factor in cases of medical injury: a study on verdict's judgment among closed claims in Japan	No health insurance claims data used	closed claims
Toyoda 2015	Visualization-based medical expenditure analysis support system	Others (duplicate/ no original data used)	Not available
Toyoshima 2018	Workers' sleep condition and related disorders in Japan: an analysis based on health insurance claim data and questionnaire	Others (duplicate/ no original data used)	among workers in a company
Tsuchiya 2014	[Information technology for ensuring drug safety]	Review article	Symposium Review
Tsuda 2017	Effect on Helicobacter pylori eradication therapy against gastric cancer in Japan	Using published open data	NDB Open data
Tsuji 2015	How to Measure the Long-term Effect of e-Health: Application of DID-PSM	Combined with primary research	National Health Insurance + mail survey
Tsukada 2016	Patterns of prescribing radiotherapy and bevacizumab in nationwide practice - analysis of 101 designated cancer care hospitals in Japan	Duplicates	duplicate (Tsukada 2016)

Tsukamoto 2016	Have Case Loads of Radical Surgery for Prostate Cancer Been Concentrated in Hospitals with Robotic Equipment ?--Analyses with Questionnaire Survey and Diagnostic Procedure Combination (DPC) Data	Combined with primary research	questionnaire survey
Tsutsumi 2020	Correction to: Impact of oral voriconazole during chemotherapy for acute myeloid leukemia and myelodysplastic syndrome: a Japanese nationwide retrospective cohort study	Others (duplicate/ no original data used)	Correction
Uchimoto 2019	Prognostic impact of C-reactive protein-albumin ratio for the lethality in castration-resistant prostate cancer	No health insurance claims data used	collected clinical records
Uenishi 2010	[Nutrition and bone health. Food for specified health use (FOSHU) and bone health]	Others (duplicate/ no original data used)	different theme
Usuki 2020	Retraction statement: National database study on the use of long-acting antipsychotic injections and hospital readmission proportions in patients with schizophrenia in Japan	Others (duplicate/ no original data used)	Retraction
Usuki 2020	National database study on the use of long-acting antipsychotic injections and hospital readmission proportions in patients with schizophrenia in Japan	Others (duplicate/ no original data used)	Retraction
Wada 2011	[Economical analysis in diagnosis procedure combination for disseminated intravascular coagulation]	Using published open data	DPC data (MHLW)
Wang 2010	Impact of the Japanese diagnosis procedure combination-based payment system in Japan	Using published open data	DPC hospitals (disk: MHLW)
Watanabe 2019	Japanese Case of Enzalutamide-Resistant Prostate Cancer Harboring a SPOP Mutation With Scattered Allelic Imbalance: Response to Platinum-Based Therapy	Others (duplicate/ no original data used)	Case Report
Watanabe 2020	Effect of surgical margin width after R0 resection for intrahepatic cholangiocarcinoma: A nationwide survey of the Liver Cancer Study Group of Japan	No health insurance claims data used	The Nationwide Follow-up Survey of Primary Liver Cancer
Yamada 2015	Surgical Outcomes of Patients With Stage III Thymoma in the Japanese Nationwide Database	No health insurance claims data used	a nationwide study on thymoma (JART)
Yamada 2020	Prevalence and clinical characteristics of children with medical complexity in Tottori Prefecture, Japan: A population-based longitudinal study	Hospital-based (<10 institutes)	three hospitals
Yamaguchi 2019	Flutamide as an Alternative Anti-androgen Agent and Predictor of the Efficacy of Novel Androgen Receptor-targeted Agents	No health insurance claims data used	medical records
Yamamoto 2014	[Privacy and public benefit in using large scale health databases]	Review article	Symposium Review
Yamamoto 2016	Large-scale Health Information Database and Privacy Protection	Review article	Conferences and Lectures
Yamamoto 2018	Perforation and Postoperative Bleeding Associated with Endoscopic Submucosal Dissection in Colorectal Tumors: An Analysis of 398 Lesions Treated in Saga, Japan	No health insurance claims data used	Saga Medical School and Saga-ken Medical Centre Koseikan
Yamamoto 2019	Resuscitative endovascular balloon occlusion of the aorta (REBOA) is associated with improved survival in severely injured patients: A propensity score matching analysis	No health insurance claims data used	Japan Trauma Data Bank
Yamanaka 2020	[Using Real World Data in Cancer Clinical Studies]	Review article	Review
Yamashita 2017	Comparison of the length of stay in patients hospitalized and initiated with dabigatran or warfarin for a concomitant non-valvular atrial fibrillation in real-world Japanese therapeutic practice (SHORT-J)	Duplicates	Duplicate (山下2017)
Yamauchi 2019	Evaluation of neurofibromatosis type 1 progression using a nationwide registry of patients who submitted claims for medical expense subsidies in Japan between 2008 and 2012	No health insurance claims data used	a nationwide registry of patients who submitted claims to receive medical expense subsidies
Yanai 2016	Syndrome of inappropriate antidiuretic hormone secretion in a patient with castration-resistant prostate cancer treated with enzalutamide	No health insurance claims data used	letter to the editor
Yang 2011	Impact of physical activity and performance on medical care costs among the Japanese elderly	Combined with primary research	with a self-administered questionnaire
Yano 2012	Association between prehypertension and chronic kidney disease in the Japanese general population	No health insurance claims data used	a nationwide Japanese database
Yasunaga 2010	Clinical features of bowel anisakiasis in Japan	Duplicates	Duplicate (Yasunaga 2010)
Yasunaga 2014	[Application of the diagnosis procedure combination (DPC) data to clinical studies]	Review article	Review
Yoshida 2010	Health insurance and payment systems for severe acute pancreatitis	Review article	Guidelines
Yoshimura 2018	Survey of anticonvulsant drugs and lithium prescription in women of childbearing age in Japan using a public national insurance claims database	Using published open data	NDB Open data
Yoshioka 2010	Comparison of public and private care management agencies under public long-term care insurance in Japan: a cross-sectional study	No health insurance claims data used	Long-Term Care Insurance (LTCI)
Zaitsu 2016	Risk of alcohol consumption in bladder cancer: Case-control study from a nationwide inpatient database in japan	No health insurance claims data used	a nationwide inpatient clinical database
Zeller 2018	An international investigation into AB plasma administration in hospitals: how many AB plasma units were infused? The HABSWIN study	No health insurance claims data used	
Abe 2014	[Evaluation of DPC classification by Decision tree method : Analysis and discussion about pneumonia]	Hospital-based (<10 institutes)	
Inoue 2019	[Factors related to life expectancy in prefectures: An ecological study using the National Database]	No health insurance claims data used	Health Checkup Database
Inoue 2019	[The Factors of Disparity Related to the Life Expectancy among Prefectures : an Analysis Using the National Data Base : Ecological Study]	No health insurance claims data used	Health Checkup Database
Uryu 2012	[Changes in medical treatment fees for pneumococcal pneumonia in our hospital before and after the introduction of DPC]	Hospital-based (<10 institutes)	Single-center
Endo 2012	[Economical Optimization of the Inpatient's Visit to Dermatology Department : Simulation Study]	Hospital-based (<10 institutes)	Single-center
Okumura 2014	[Trends of psychotropic medication use among children and adolescents in Japan data from the national insurance claims database between 2002 and 2010]	Using published open data	Statistics of Medical Care Activities in Public Health Insurance
Oka 2016	[Practical use of the data in a patient ward change of function]	Hospital-based (<10 institutes)	Single-center
Okamoto 2013	[Do the prices of DPC (diagnosis-procedure-combination) hospitals reflect their quality of care? : The relationship between the price levels and the quality of care as measured by case-mix-adjusted in-hospital mortality]	Using published open data	DPC data (MHLW)
Okamoto 2014	[Estimation of per-case hospitalization charges from Diagnosis-Procedure-Combination (DPC) data and an international comparison of hospital prices]	Using published open data	DPC data (MHLW)
Onda 2010	[BENCHMARK FOR THE TREATMENT OF PNEUMONIA AT DPCHOSPITALS FOCUSING ON THE USE OF ANTIMICROBIALS]	Hospital-based (<10 institutes)	4 hospitals

Kato 2020	[Efforts to achieve proper use of sleeping pills using the sleeping pill formula/NDB open database]*	Hospital-based (<10 institutes)	single-center
Kawai 2011	[Economic Benefits and Problems concerning the Introduction of Generic Drugs in a Hospital with a DPC System]	No health insurance claims data used	questionnaire survey
Kawai 2014	[Effort for regional medical analysis using DPC data and Quantum GIS (QGIS)]	Hospital-based (<10 institutes)	single-center
Kono 2018	[Hospital Mortality of Spinal Infection：Results from Japanese Nationwide Diagnosis Procedure Combination Database]	Using published open data	DPC data (MHLW)
Toyama 2014	[C2-4 Changing in Activities of Daily Living in the Hospital Stay]	Hospital-based (<10 institutes)	5 hospitals
Gamazawa 2013	[Utilization of out-of-hospital prescriptions for remote areas during disasters Examples from the 2011 Great East Japan Earthquake]	No health insurance claims data used	Survey
Iwata 2021	[Survey of Dementia Drugs and PIM and Multi-Drug Combinations Using NDB Open Data]*	Using published open data	NDB Open data
Yoshinaga 2013	[An approach for improving clinical process using Hospital Information System(HIS) data]	Hospital-based (<10 institutes)	Single-center
Yoshimi 2018	[Comparison of prefectures using items related to smoking and quitting smoking in the 2nd NDB Open Data]*	Using published open data	NDB Open data
Kubo 2018	[DPC system for treatment of facial nerve palsy]	Using published open data	DPC summary tables (MHLW)
Miyamoto 2010	[Influence on the outcome of the DPC cords by comparing Nissay Hospital with other medical institutions to which ICD10 cords and K-cords are applied]	Hospital-based (<10 institutes)	5 hospitals
Miyamoto 2013	[Strengths of our hospital by MDC analysis using DPC public data]*	Using published open data	DPC data (MHLW)
Kuchiki 2010	[Problems of DPC in stroke care]*	Using published open data	
Kuchiki 2011	[Improvement of DPC in the Stroke Medical Care]	Hospital-based (<10 institutes)	"our treatment options"
Kaneko 2018	[Factors affecting profit/loss at national university hospitals]	Using published open data	DPC data (MHLW)
Kinjo 2010	[Attempts at marketing using the impact assessment survey data of the introduction of DPC]*	Using published open data	DPC data (MHLW)
Komada 2017	[Surveillance to Determine Adverse Reactions to Carbamazepine and Lamotrigine: Analysis of the "Japanese Adverse Drug Event Report", "Information on Adverse Reaction Relief Benefits" and "Health] Insurance Claims and Specific Health Checkups of Japan" Databases	Using published open data	NDB Open data
Kuriyama 2014	[Analysis of Cancer Treatment in Iwate Prefecture – An Analysis of Health Care Regions Based on MHLW DPC Data]	Duplicates	duplicate (Kuriyama 2014)
Harada 2012	[Analysis of hospital stay in patients with cerebral apoplexy]	Hospital-based (<10 institutes)	single-center
Furuichi 2010	[Effects and Problems of DPC (Diagnosis Procedure Combination): A Study on Femoral Neck Fractures]	Hospital-based (<10 institutes)	Single-center
Furuta 2015	[Estimation of prevalence of dementia among aged inpatients in general hospital]	Combined with primary research	CGA
Igarashi 2020	[Estimating the Social and Economic Burden of Progressive Supranuclear Palsy in Japan]	Combined with primary research	patient／caregiver survey (medical／nonmedical costs and productivity loss)
Goto 2011	[Practical utility of the data of DPC as the management-tool in the hospital not adopting DPC]	Hospital-based (<10 institutes)	single-center
Mitsutake 2018	[The associations between readmission within 30 days and the medical institute factors among older patients receiving home medical care]	Duplicates	Duplicate (Mitsutake 2018)
Kudo 2018	[Comparison of caries severity between those who perform dental maintenance and those who do not and investigation of the importance of maintenance using big data]*	Others (duplicate/ no original data used)	Not available
Yasunaga 2016	[Clinical epidemiological study using integrated data of DPC data and test values]*	Others (duplicate/ no original data used)	
Tsunekawa 2020	[A Trial for Improving Coverage Rate Indices in Diagnosis Procedure Combination-Designated Medical Institutions]	Hospital-based (<10 institutes)	Single-center
Ehara 2014	[Are pediatric inpatients centralized at core hospitals/regional pediatric centers? An analysis using DPC data]*	Using published open data	DPC data (MHLW)
Ehara 2017	[Current status of hospitalization of children by prefecture as estimated from NDB open data]*	Using published open data	NDB Open data
Arai 2016	[Importance of care process management in terms of financial perspective : based on knowledge of hospital managers]	No health insurance claims data used	questionnaire survey
Arakawa 2013	[Economic evaluation for the prevention of cervical cancer by vaccination--from perspective of health insurance society and industry]	Duplicates	Duplicate (Alakawa 2013)
Takahashi 2015	[The Characteristics of Income and Expenditure in MDC04C]	Hospital-based (<10 institutes)	5 institutions
Takahashi 2018	[Survey of opening clinical indicators of hospitals with DPC/PDPS in Hokkaido area]	Using published open data	DPC data (MHLW)
Takahashi 2020	[Analysis of prolonged average length of hospital stay in colon malignant tumors]	Hospital-based (<10 institutes)	Single-center
Kurosaki 2020	[Medical expenses for diabetes care in Japan: Analysis of inter-prefecture differences]	Duplicates	Duplicate (Kurosaki 2020)
Kuroda 2020	[Survey on Off-Label Use of Antineoplastic Agents in Pediatric Patients using a Large-scale Medical Database in Japan]	Duplicates	Duplicate (Kuroda 2020)
Konomura 2016	[A Survey on Hospital Formulary Development in Japan in 2015]	No health insurance claims data used	questionnaire survey for medical institutions
Imai 2012	[Survey research group based on DPC data regarding the implementation status of standard drug treatment and clinical outcomes: A study on the implementation status survey of standard drug treatment using DPC data]*	Hospital-based (<10 institutes)	Single-center
Imai 2015	[A Multi-institutional Survey of Risk Factors for the Prescription of Brought-in Medicine]	No health insurance claims data used	questionnaire survey
Imai 2016	[Recurrence rate of ischemic stroke in the Shizuoka Stroke Network: a retrospective multicenter study]	Hospital-based (<10 institutes)	three general hospitals
Sato 2015	[Current situation and issues related to claiming of charges for the operations of nutrition support teams]	Combined with primary research	questionnaire survey
Sato 2017	[Investigation on the situation of onychomycosis treatment using a survey on medical fee receipts and a questionnaire survey to patients with onychomycosis]	Combined with primary research	a questionnaire survey
Sato 2017	[The Actual Condition of the Combined Diagnoses and the Influence of Added Modifiers for ICD-10 Codes]	Hospital-based (<10 institutes)	a hospital group
Sato 2019	[Age-related trends in blood pressure based on large-scale health checkup data using longitudinal analysis]	No health insurance claims data used	health checkup data

Sakamoto 2014	[Factor extraction of the liver cancer by the decision tree analysis using DPC data]	Others (duplicate/ no original data used)	Abstract
Sakamoto 2014	[Decision Tree Analysis of Factors Relating to Lung Cancers Using DPC Data]	Hospital-based (<10 institutes)	4 hospitals
Sakamoto 2019	[Analysis of estimate factors about the severity of cerebral infarction and patient's level of need for medical treatment and nursing care]	Hospital-based (<10 institutes)	three hospitals
Miura 2017	[Current status and measures against non-communicable diseases (NCD) in the National Health Plan]	No health insurance claims data used	WHO: MiNDbank
Yamashita 2019	[Clustering Analysis of DPC Data Using Weighted PLSA for Medical Treatments for Patients with Sepsis Including Blood Purification]	Hospital-based (<10 institutes)	single-center
Yamazaki 2018	[Big data analysis at Osaka International Cancer Center]*	Hospital-based (<10 institutes)	Osaka International Cancer Institute
Yamazaki 2020	[Examination of problems: Indications for myelography]*	Hospital-based (<10 institutes)	Single-center
Yamanaka 2016	[Development of a Framework for Evaluating Appropriateness of Antimicrobial Agent Use]	Hospital-based (<10 institutes)	single-center
Yamada 2012	[Economic Analysis of Profit Improvement Derived from the Introduction of Generics by Tokai Consortium for Municipal Hospitals (ToCoM)]	Hospital-based (<10 institutes)	9 hospitals (ToCoM)
Yamauchi 2018	[Current status of osteoporosis screening – Relationship with osteoporotic fractures and need for long-term care –]	Using published open data	NDB Open data
Yamanouchi 2018	[Considering community "recovery" from hospital discharge rate and re-hospitalization rate]	Using published open data	published data
Yamamoto 2015	[An example of management simulation using DPC data and operating room system data]*	Hospital-based (<10 institutes)	Single-center
Teraoka 2021	[A study on the COVID-19 pandemic and hospital medical fees　Assessment of impact using DPC data from QIP participating hospitals]*	Combined with primary research	QIP + questionnaire survey
Sakai 2013	[DPC Data Based Situation Analysis of Regional Core Hospital: An Application of the GIS Methodology for Regional Health Care Plans]	Duplicates	Duplicate (Sakai 2013)
Munakata 2016	[Relationship between Lipid Measures and Brain or Heart Disease in the Japanese General Population:The Watari Study]	Combined with primary research	Watari study (a community-based prospective cohort study)
Akiyama 2012	[Investigation of the Actual Health Care Area Using Diagnosis Procedure Combination (DPC) Data and Geographic Information System (GIS) in the Secondary Health Care Area]	Hospital-based (<10 institutes)	Single-center
Funahashi 2013	[Attributes of non-participants aged 40-59 years in specific health check-ups]	No health insurance claims data used	questionnaire survey
Ogata 2012	[Investigation of the hospitalization days and medical cost of the homeless and the livelihood protection patient using DPC data for four years]	Hospital-based (<10 institutes)	Single-center
Obara 2016	[Additional cost in medical care and length of stay due to aspiration pneumonia that inpatients developed : Observational study using DPC data]	Hospital-based (<10 institutes)	Single-center
Obara 2016	[A sensitivity analysis of medical expenses for aspiration pneumonia which occurred after admission]	Hospital-based (<10 institutes)	Single-center
Kobayakawa 2014	[The Analysis of Critical Path using Receipt Data and Its Application]	Hospital-based (<10 institutes)	Single-center
Koike 2019	[A fact-finding survey on the characteristics of those who became insured persons of the National Health Insurance after mandatory retirement, etc., and the support provided by National Health Insurance Organizations]*	No health insurance claims data used	questionnaires to insurers
Oda 2018	[Examination of idiopathic pulmonary fibrosis patients with rapidly progressive severe respiratory failure using DPC data]*	Others (duplicate/ no original data used)	
Obata 2013	[Efficiency Index of Diagnosis Procedure Combination after Adjustment for Disease Severity and Definitions of Advanced General Hospital for the Acute Care]	Hospital-based (<10 institutes)	6 hospitals
Matsui 2010	[An action for attaching the suitable disease name using medical record management data and DPC survey data]	Hospital-based (<10 institutes)	Single-center
Matsui 2013	[Attempt to analyze regional characteristics using GIS software and DPC data]	Hospital-based (<10 institutes)	Single-center
Matsunaga 2012	[FACTORS RELATED TO THE INTER-HOSPITALS DIFFERENCE IN THE STATES OF IMPLEMENTATION OF DRUG MANAGEMENT GUIDANCE JOBS]	Combined with primary research	questionnaire survey
Matsuhisa 2014	[Establishment of Tokushima Prefecture Diabetes Medical Care Coordination System Using ICT]*	Others (duplicate/ no original data used)	Establishment of the local medical care system
Matsuyama 2017	[Regional disparities in dental consultation for preschool children]*	Using published open data	Survey on the Trend of Medical Care Expenditures (MHLW)
Matsuta 2020	[Regional Difference Analysis in Age-Adjusted Medical Expenses Focusing on the Cohort and Medical Practice Codes]	Using published open data	Analysis of regional differences in health care costs (MHLW)
Matsuda 2018	[Big data analysis of medical and nursing care: Examination of the effects of specific health checkups and specific health guidance in regional insurance]*	No health insurance claims data used	health checkup data
Matsuda 2018	[A study on appropriate allocation of acute myocardial infarction treatment facilities using DPC and NDB]*	Using published open data	DPC data (MHLW)
Matsumoto 2018	[Relationship between the Number of Adult Obesity and Neuropsychiatric Prescription Drugs]	Using published open data	NDB Open data
Kamijo 2020	[A Study on Estimating the Extraction of Permanent Teeth by Main Reason, Using the Japanese National Database of Health Insurance Claims and Specific Health Checkups of Japan (NDB Japan) and the Second Survey of Reasons for Permanent Teeth Extraction]	Using published open data	NDB Open data
Shida 2010	[Image Examination of Malignant Tumors in Lungs Observed on the Basis of Cost Analysis Using Diagnosis Procedure Combination Data]	Duplicates	Duplicate (Shida 2010)
Moriwaki 2019	[Evaluating medical functions in hospital wards by focusing on patients' structure]	Hospital-based (<10 institutes)	Single-center
Kamigaki 2012	[On Prescription of Beta 2 Agonists for Pediatric Patients with Acute Bronchitis in Japan]	Combined with primary research	intervention study
Jingushi 2019	[The present situation of the fracture treatment in Japan according to the 2nd open data from the National Database of Health Insurance Claims and Specific Health Checkups of Japan]	Using published open data	NDB Open data
Sugita	[Developing systems for analyzing and utilizing data about health services to prevent lifestyle-related diseases in districts]	No health insurance claims data used	system building

Suga 2018	[The Current State of Non-traumatic Intracranial Hemorrhage Treatment in Japan Using the Diagnosis Procedure Combination Data from 2014]	Using published open data	DPC data (MHLW)
Shimizu 2018	[A comprehensive survey of clinical practice concerning telemedicine treatment]	Using published open data	NDB Open data
Shimizu 2021	[Counseling project for patients jointly conducted by the Japan Pharmaceutical Association, insurers, Japan Medical Association, and universities (2nd report): Analysis of health insurance claims data and questionnaire results for doctors]*	Combined with primary research	intervention study
Nishimura 2011	[Influence of staffing and administrative policy of ICU on Patient outcome]	No health insurance claims data used	questionnaire survey
Nishina 2011	[Evaluation of the impacts of DPC based on obstetric DIC]*	Hospital-based (<10 institutes)	Single-center
Ishikawa 2013	[A study on the effects of preventive interventions for specific health guidance: A causal analysis with propensity scores using a large-scale database]*	No health insurance claims data used	Health checkup data
Ishikawa 2018	[The ratio of home deaths and related factors in national municipalities, ecological study]	No health insurance claims data used	official data
Kawamura 2015	[Effectiveness of Colorectal Cancer Screening as Means of Disease Discovery in Terms of Disease Progression Status and Medical Costs]	No health insurance claims data used	a hospital group
Asaumi 2011	[Report of analysis case with medical treatment statistics that use DPC data]	Hospital-based (<10 institutes)	Single-center
Asami 2015	[The current state and the outcome of dementia patients in the acute hospital]	Hospital-based (<10 institutes)	Single-center
Maeda 2014	[Regional characteristics and future prospects in the number of patients TKA from public data DPC]	Using published open data	DPC data (MHLW)
Muramatsu 2013	[Prefectural Medical Care Delivery System from the National Database (NDB): Analysis of Lifestyle-related Medical Practices Using Publicly Available Indicators Created from the NDB]*	Using published open data	DPC Research Institute
Murakami 2012	[Comparison between medical treatment fee between DPC and service basis for spinal infection]	Hospital-based (<10 institutes)	Single-center
Murashima 2011	[HIM's function on the job of outpatients' department: a report from examination committee notification]	No health insurance claims data used	report from examination committee notification
Ota 2020	[Proposal of Method for Estimating the Situation of Regional Healthcare Using NDB Open Data and Sickbed Function Reports Data: Estimation of Implementation Status of Surgery in Inpatient Care in Okayama Prefecture]	Using published open data	NDB Open data
Osawa 2018	[Efforts to increase the proportion of generic drugs used]*	Others (duplicate/ no original data used)	
Otsubo 2012	[Readmission rate for health care delivery system assessment]	Duplicates	Duplicate (Ootsubo 2012)
Oshima 2016	[Change in the number of denture according to survey statistics of medical care activities in public health insurance]	Using published open data	statistics of medical care activities in public health insurance
Oshima 2018	[Correlations between regional distribution of dental hygienists and dentistry service : Analysis using NDB open data Japan]	Using published open data	NDB Open data
Oshima 2020	[Outpatient chemotherapy/inpatient chemotherapy: Management analysis in cost accounting: Profit-based study on individual anticancer drugs]*	Hospital-based (<10 institutes)	Single-center
Ono 2020	[Age-and gender-specific prescriptions and fracture surgeries of osteoporosis treatment in Japan using the National Health Insurance Claim Database]	Using published open data	NDB Open data
Tanihara 2012	[Trend and current situation of medical research using health insurance claims among Japan, Korea, and Taiwan]	Review article	review study
Niwa 2020	[Evaluation of the Antimicrobial Consumption Calculated Using the Japan Surveillance for Infection Prevention and Healthcare Epidemiology (J-SIPHE) System]	Hospital-based (<10 institutes)	single-center
Ikehata 2018	[Benchmarking of Clinical Pathways for endoscopic submucosal dissection using DPC data]	Hospital-based (<10 institutes)	Single-center
Ikehata 2018	[Benchmarking of clinical pathway system for endoscopic submucosal dissection according to DPC data of 9 hospitals among Iwate Prefectural Hospital group]	Hospital-based (<10 institutes)	9 hospitals
Ikehata 2020	[Hemorrhagic peptic ulcer -Current status and medical-economic evaluation-]	Hospital-based (<10 institutes)	Single-center
Nakamura 2015	[Evaluation of the Number of Varicella Patients Estimated by Prescription Surveillance]	Duplicates	Duplicate (Nakamura 2015)
Nakamura 2016	[Current status of oral anticoagulants and stroke patients at our hospital]*	Hospital-based (<10 institutes)	single-center
Nakajima 2014	[Examination of correlation between medical expenses with average life expectancy according to municipality and gender]	Using published open data	Analysis of regional differences in medical expenses (MHLW)
Nakajima 2014	[Evaluation of University Hospitals Including Diagnosis Procedure Combination (DPC) Data, Functional Evaluation Coefficient II, and Management Indicators]*	Using published open data	DPC data (MHLW)
Nakajima 2015	[Examination of the Difference in Medical Treatment Contents According to Major Diagnostic Category of Hospital Group Ⅰ and Group Ⅱ Using the Diagnosis Procedure Combination Survey Data]	Using published open data	DPC survey data (MHLW)
Nakajima 2015	[Examination of diagnosis procedure combination survey data that influence function evaluation coefficient II]	Using published open data	DPC survey data (MHLW)
Nakajima 2015	[Investigation of average life expectancy by prefecture using basic data on regional differences in medical care]*	Using published open data	(regional differences in health care)
Nakajima 2016	[Type Classification Based on Inpatient Ratio According to Major Diagnosis Category of Group III in the Diagnosis Procedure Combination System]	Using published open data	DPC data (MHLW)
Nakajima 2016	[Investigation of factors affecting functional evaluation coefficient II of DPC hospitals using multiple regression analysis]*	Using published open data	DPC data (MHLW)
Nakajima 2017	[Examination of Factors that Influence the Operation Income and Expenditure Balance Difference Rate of 20 Educational Foundation Universities]	Using published open data	DPC data (MHLW)
Nakajima 2017	[Investigation of Nagano-type and metropolitan-type prefectures in relation to average life expectancy using DPC data]*	Using published open data	DPC data (MHLW)
Nakajima 2020	[Major diagnostic group classifications and medical practices that affect the shortening of hospital stay]*	Using published open data	DPC data (MHLW)
Nakajima 2020	[Examination of Factors Affecting Life Expectancy by Prefecture Based on the Criteria of the Organisation for Economic Co-operation and Development]	Using published open data	Regional differences in healthcare delivery conditions (Cabinet Office)
Nakari 2015	[Factors that Prolong Admission to Acute Care Hospitals]	No health insurance claims data used	
Nakamoto 2016	[Can the quality of medical care be improved by providing feedback on the evaluation results of quality indicators for cancer care?]*	Hospital-based (<10 institutes)	4 hospitals

Asamoto 2016	[An economic analysis of relationship between hospital types and length of stay in a hospital]	Using published open data	DPC data (MHLW)
Asamoto 2017	[The organizational factors affecting hospitalization of patients with cerebral infarction (without surgery) -Empirical findings on DPC hospitals in Tokai district's 4 prefectures-]	Using published open data	DPC data (MHLW)
Hasegawa 2013	[The study of the promotion policy of the telemedicine]	No health insurance claims data used	
Tsukada 2011	[Profitability study of laparoscopic gastrectomy cases after introduction of DPC]*	Hospital-based (<10 institutes)	Single-center
Tsukada 2016	[Patterns of prescribing radiotherapy and bevacizumab in nationwide practice : analysis of 101 designated cancer care hospitals in Japan]	Others (duplicate/ no original data used)	Japanese version (Tsukada 2016)
Tsuji 2012	[The Long-term Effect of Telecare: Based on 10 Year Receipt Data of Nishi-aizu Town]	Combined with primary research	with questionnaire survey
Tsujioka 2018	[The MDC portfolio management with the DPC public data and the level of need for nursing]	Using published open data	DPC data (MHLW)
Tahara 2013	[A study on trends in the flow of patients centering on regional core hospitals in rural areas]*	Hospital-based (<10 institutes)	single-center
Taguchi 2012	[Efforts to utilize DPC data and future issues]*	Hospital-based (<10 institutes)	Single-center
Tagami 2018	[Elucidation of circulatory and respiratory dynamics in post-cardiac arrest syndrome: Approach by transpulmonary thermodilution]*	No health insurance claims data used	
Tamura 2017	[A study on the Prescription Status of Metformin and DPP–4 Inhibitors in Patients with Type 2 Diabetes Mellitus in Japan: An Analysis of Data from Health Insurance Administrative Claims]	Duplicates	Duplicate (Tamura 2017)
Tamura 2018	[A Study on the Prescription Status of Metformin and DPP–4 Inhibitors in Patients with Type 2 Diabetes Mellitus in Japan : An analysis of Time Trends by Use of a Health Insurance Administrative Claims Database]	Duplicates	Duplicate (Tamura 2018)
Tashiro 2014	[Assessment of the Adequacy of the DPC Score in Neonatal Care]	Hospital-based (<10 institutes)	7 hospitals
Tashiro 2015	[Verification of medical fees in neonatal intensive care units: To provide high-quality medical care efficiently and economically]*	Hospital-based (<10 institutes)	7 hospitals
Tashiro 2015	[Assessment of the adequacy of the DPC score in neonatal care : Analysis of the DPC data from children's hospitals]	Hospital-based (<10 institutes)	7 hospitals
Tanaka 2015	[Cancer treatment situation in Japan with regard to the type of medical facility using medical claim data of Health Insurance Societies]	Duplicates	duplicate (Tanaka 2015)
Tanaka 2016	[On the Size Distribution of Hospitals: Empirical Study and Stochastic Model]	Using published open data	DPC data (MHLW)
Tanaka 2016	[The effects of the functional differentiation of hospitals on the regional mean length of stay]*	Using published open data	DPC data (MHLW)
Tanaka 2017	[Actual use of direct oral anticoagulant (DOAC) in fiscal year 2014 : using the national database of health insurance claims and specific health checkups of Japan (NDB) open data]	Using published open data	NDB Open data
Tanaka 2018	[Determining the acute period in transurethral bladder tumor resection using DPC data]*	Hospital-based (<10 institutes)	single-center
Tanaka 2018	[Survey of Prescription Volume and Adverse Events of NSAIDs Patches in Fiscal Year 2015]	Using published open data	NDB Open data
Tanaka 2019	[Analysis of DPC Data by Topic Model and Consideration on Function of Hospitals]	Using published open data	DPC data (MHLW)
Tanaka 2019	[Investigation of the usefulness of antimicrobial use survey using Diagnosis Procedure Combination (DPC) data]*	Duplicates	Duplicate (Tanaka 2019)
Tanaka 2019	[Investigation of Prescription Pattern of Antiretroviral Using the National Database of Health Insurance Claims Specific Health Checkups of Japan Open Data]	Using published open data	NDB Open data
Tanaka 2020	[Survey on prescribing status of NSAIDs patch formulations: Perspectives on promotion of generic drug use and prescribing restrictions]*	Using published open data	NDB Open data
Tazawa 2013	[Investigation of the need for assistance using ADL score at admission]*	Hospital-based (<10 institutes)	Single-center
Tazawa 2016	[Investigation of the state of care required and destination of discharge using DPC data]*	Hospital-based (<10 institutes)	Single-center
Watanabe 2019	[Inpatient survey for prefectural medical plan formulation]	Combined with primary research	questionnaires to institutions
Fujimura 2010	[Evaluation of the Diagnosis Procedure Combination Payment System]	Hospital-based (<10 institutes)	single-center
Fujino 2017	[Multicenter inter-comparison tool for surgical procedures using Diagnosis Procedure Combination survey data]	Using published open data	DPC data (VHJ)
Nawata 2010	[Analysis of Length of Hospital Stay for Cataract Surgery Before and After Introduction of the Diagnosis Procedure Combination -Based Inclusive Payment System]	Hospital-based (<10 institutes)	5 hospitals
Minamizono 2019	[Gender, age, and regional distribution of equivalent conversion values for outpatient prescription psychotropic drugs: From the 2nd NDB Open Database]*	Using published open data	NDB Open data
Hatanaka 2015	[Risk factors for ischemic heart disease in males in the prime of life: An eight-year follow-up study]	Duplicates	Duplicate (Hatanaka 2015)
Ozaki 2018	[Impact of Oral Hygiene Management through a Multidisciplinary Approach on Patients with Acute Stroke: Building a Pneumonia-Prevention System by Dentistry]	Hospital-based (<10 institutes)	Single-center
Muto 2014	[Evaluation of Cost-effectiveness analysis in Hospital Management by using Diagnosis Procedure Combination (DPC) data : a pilot case of the medical equipment]	Hospital-based (<10 institutes)	Single-center
Fushimi 2010	[Health care Resource Allocation by DPC Case-mix Data]	Review article	Review
Fushimi 2016	[Toward the improvement of health and medical statistics using electronic health insurance claims: Overview of "Study on Improving Claims Statistics Using Electronic Claims" (Part 1)]*	Review article	Review
Fukuda 2012	[Estimates of Postoperative Resource Utilization Attributable to Surgical Site Infection in Gastrectomy Patients: Evidence from The JANIS/DPC Integrated Database]	Hospital-based (<10 institutes)	6 institutes
Fukuda 2014	[Possibility of analyzing claim data for specified insurance medical materials]*	No health insurance claims data used	insurance coverage documents
Fukuda 2017	[A study on building an integrated database of medical and clinical information]*	Hospital-based (<10 institutes)	7 hospitals
Fukudome 2014	[A study and report: Analysis of acute inpatient care in the Kagoshima medical area using DPC and patient survey data]*	Using published open data	DPC data (MHLW)
Hiraoka 2018	[A Study of Evaluating Risk Factors Assessment sheet for Prediction of Falls in Inpatients : Example of using Diagnosis Procedure Combination (DPC) data]	Hospital-based (<10 institutes)	Single-center

Minetaki 2010	[Empirical analysis of emergency medical care using medical microdata and the possibility of telemedicine]*	Hospital-based (<10 institutes)	Single-center
Horai 2019	[Study of Flat-Sum Payment system and Opportunity cost in DPC system : especially considering lost profits of Radiological Technologist and Nurse]	No health insurance claims data used	
Kitamura 2021	[The pulmonary rehabilitation utilization rate among older people with chronic obstructive pulmonary disease in Japan: A retrospective study using medical and long-term care insurance claims data]	Duplicates	Duplicate (Kitamura 2021)
Makino 2017	[Hybrid Research Concerning the Actual Prescription of Non–irritative Laxatives and Patient Consciousness]	Combined with primary research	questionnaire survey
Akematsu 2010	[Empirical analysis of a home health management system and medical expenses for four major lifestyle-related diseases]*	Combined with primary research	questionnaire survey
Akematsu 2012	[Effect of Telecare on Medical Expenditures Estimated by Propensity Score Matching JTTA 2012 KOBE]	Combined with primary research	questionnaire survey
Akematsu 2013	[Long-term Effect of Telecare Intervantion on Patients with Chronic Diseases]	Combined with primary research	on-site survey
Kimura 2015	[Treatment Persistence with Antidepressants in Japan: Assessment of the First 60 Days of Treatment Based on Pharmacy Claims Data from Reimbursement Database]	Duplicates	Duplicate (Kimura 2015)
Kadowaki 2020	[National trend of total hip arthroplasty evaluated by data from diagnosis procedure combination (DPC)]	Using published open data	DPC data (MHLW)
Nonoshita 2012	[Action to the improvement of clinical process in pneumonia by using Diagnosis Procedure Combination (DPC) data]	Hospital-based (<10 institutes)	single-center
Ryu 2010	[To establish the standardized clinical pathway in gastric and colorectal cancers by benchmark analysis of DPC data]	Hospital-based (<10 institutes)	five participating cancer centers
Suzuki 2011	[The number of emergency admissions in Accredited Training Institutions for Fellowship and Senior Fellowship of the Japanese Association for Acute Medicine based on the DPC information]	Using published open data	DPC data (MHLW)
Suzuki 2019	[Analysis on Real World Data（RWD）Using a Machine Learning Model: Exploration of the Factors Affecting the Utility of Oral Hypoglycemic Agents]	Duplicates	Duplicate (Suzuki 2019)
Wada 2019	[Macro analysis of dental reimbursement claims in the last 10 years]*	Using published open data	Health Insurance Claims Review & Reimbursement services (Survey of medical treatment status by medical institution)
Kijima 2012	[Analysis of Outpatient clinic, using E and F File of Outpatient]	Hospital-based (<10 institutes)	Single-center
Kunisawa 2019	[[Introduction to health economics for healthcare professionals] Association between shortened hospital stay and readmission rate]*	Others (duplicate/ no original data used)	(Contents: Kunisawa 2016)
Hamada 2011	[ASSESSMENT OF WORKLOAD IN OBSTETRICS AND GYNECOLOGY PRACTICE BY TIME STUDY COMBINED WITH ADMINISTRATIVE DATA]	Combined with primary research	time-study
Saito 2012	[Relationship between exercise habits at the recommended level of exercise and out-of-hospital medical expenses: A study in Fujisawa City]*	Combined with primary research	questionnaire survey

Supplement file 4 Characteristics of included studies

ID (Author Year)	Title (*titles translated by author)	Database	Notes on database	Setting	Notes on setting	Start data year	End data year	Research area/ theme	Research area/ theme-2	Disease	Disease-2	Notes on disease	Age	Notes on age
1) Certain infectious and parasitic diseases (A00-B99)														
Sugiyama 2013	[Anisakis food poisoning: the annual number of cases noticed by ordinance for enforcement of the food sanitation act and estimated through healthcare claim data analysis]	JMDC		Nationwide		2005	2011	Clinical epidemiology, course of diseases	Health policy evaluation and utilization	Certain infectious and parasitic diseases		Anisakis		
Kimura 2020	Clostridioides (Clostridium) difficile infection in Japanese hospitals 2008-2017: A real-world nationwide analysis of treatment pattern, incidence and testing density	MDV		Nationwide		2008	2017	Medical treatment status	Clinical epidemiology, course of diseases	Certain infectious and parasitic diseases		Clostridioides difficile infection	Others	≥18 year of age
Mikamo 2020	Incidence of and risk factors for recurrent Clostridioidesdifficile infection in Japan using a claims database: A retrospective cohort study	MDV		Nationwide		2012	2016	Clinical epidemiology, course of diseases		Certain infectious and parasitic diseases		recurrent Clostridioides difficile infection (A047)		
Fukuda 2018	Inpatient Expenditures Attributable to Hospital-Onset Clostridium difficile Infection: A Nationwide Case-Control Study in Japan	NDB		Nationwide		2010	2016	Health economics		Certain infectious and parasitic diseases		Clostridium difficile infections		
Kunishima 2018	Healthcare burden of recurrent Clostridioides difficile infection in Japan: A retrospective database study	MDV		Nationwide		2012	2016	Health economics		Certain infectious and parasitic diseases		Clostridioides difficile infection (A047)		patients of any age
Yasunaga 2012	The burden of Clostridium difficile-associated disease following digestive tract surgery in Japan	DPC		Nationwide		2007	2010	Intervention effect	Health economics	Certain infectious and parasitic diseases	Diseases of the digestive system	Clostridium difficile-associated disease following digestive tract surgery		
Ueno 2019	The clinical and economic burden of cytomegalovirus management post allogeneic hematopoietic stem cell transplantation in Japan - a retrospective database study	MDV		Nationwide		2010	2018	Health economics		Certain infectious and parasitic diseases		cytomegalovirus	Others	≥18 year of age
Kajimoto 2020	Patient and National Economic Burden of Dengue in Japan: Results from Japanese National Claims Database	NDB		Nationwide		2011	2015	Health economics		Certain infectious and parasitic diseases		Dengue		
Kajimoto 2020	Clinical Management of Patients with Dengue Infection in Japan: Results from National Database of Health Insurance Claims	NDB		Nationwide		2011	2015	Medical treatment status	Quality of care	Certain infectious and parasitic diseases		Dengue		
Iwanaga 2015	[An investigation of the clinical practice for dermatophytosis treatment using reimbursement data from health insurance societies in Japan]	JMDC	Existing epidemiological studies: JSMM, JFW, JOCD	Nationwide		2013	2014	Medical treatment status	Research methodology	Certain infectious and parasitic diseases		dermatophytosis		
Okubo 2019	Recent Prescription Patterns for Children With Acute Infectious Diarrhea	JMDC		Nationwide		2012	2015	Medical treatment status		Certain infectious and parasitic diseases		acute infectious diarrhea	Children	aged under 18 years
Morishima 2015	[Estimating the numbers of enterobiasis cases in Japan: a healthcare claims database study]	JMDC		Nationwide		2005	2009	Clinical epidemiology, course of diseases		Certain infectious and parasitic diseases		enterobius		
Takeuchi 2017	Pediatric urolithiasis associated with acute gastroenteritis: an inpatient database study in Japan	DPC		Nationwide		2010	2012	Clinical epidemiology, course of diseases		Certain infectious and parasitic diseases	Diseases of the genitourinary system	acute gastroenteritis, urolithiasis	Children	≤59 months old
Okubo 2018	Recent patterns in antibiotic use for children with group A streptococcal infections in Japan	JMDC		Nationwide		2012	2015	Medical treatment status		Certain infectious and parasitic diseases	Diseases of the respiratory system	group A streptococcus (GAS)	Children	aged <18 years
Hamada 2021	In-hospital mortality among patients with invasive non-group A beta-hemolytic Streptococcus treated with clindamycin combination therapy: a nationwide cohort study	DPC		Nationwide		2010	2018	Intervention effect		Certain infectious and parasitic diseases		invasive group A Streptococcus infection	Others	excluded patients who were under the age of 16 years
Tokunaga 2021	Cost Analysis in Helicobacter pylori Eradication Therapy Based on a Database of Health Insurance Claims in Japan	JMDC		Nationwide		2005	2018	Health economics		Certain infectious and parasitic diseases	Diseases of the digestive system	H. pylori gastritis, H. pylori infection		
Deguchi 2020	Current Status of Helicobacter pylori Diagnosis and Eradication Therapy in Japan Using a Nationwide Database	MDV		Nationwide		2008	2016	Intervention effect	Quality of care	Certain infectious and parasitic diseases		Helicobacter pylori infection		
Deguchi 2019	Association between parental history of Helicobacter pylori treatment failure and treatment failure in the offspring	JMDC		Nationwide		2005	2018	Clinical epidemiology, course of diseases	Medical treatment status	Certain infectious and parasitic diseases		Helicobacter pylori		
Hiroi 2017	Impact of health insurance coverage for Helicobacter pylori gastritis on the trends in eradication therapy in Japan: retrospective observational study and simulation study based on real-world data	Others	JMDC, MDV	Nationwide		2005	2015	Health policy evaluation and utilization		Certain infectious and parasitic diseases	Diseases of the digestive system	Helicobacter pylori gastritis		
Fujimori 2014	[[Changes in treatment of Helicobacter pylori infection after coverage of chronic gastritis insurance] Has eradication cases increased?]*	NHI/LSEHS	National Health Insurance and Later-Stage Elderly Healthcare System	Prefecture	Hokkaido	2012	2013	Medical treatment status	Health policy evaluation and utilization	Certain infectious and parasitic diseases	Diseases of the digestive system	Helicobacter pylori eradication		
Yatsuhashi 2019	[Clinical Study of Liver Disease using National Database (NDB) : Trends in the Number of Patients with Chronic Hepatitis C and Hepatitis B]	NDB		Nationwide		2012	2016	Clinical epidemiology, course of diseases		Certain infectious and parasitic diseases	Neoplasms	Chronic Hepatitis C and Hepatitis B		
Fukuda 2020	Healthcare Expenditures for the Treatment of Patients Infected with Hepatitis C Virus in Japan	NDB		Nationwide		2010	2018	Health economics		Certain infectious and parasitic diseases		Hepatitis C Virus	Others	≥20 years of age
Yamazaki 2019	Clinical and Economic Burden of Patients with Chronic Hepatitis C with Versus Without Antiviral Treatment in Japan: An Observational Cohort Study Using Hospital Claims Data	MDV		Nationwide		2008	2016	Health economics		Certain infectious and parasitic diseases		chronic hepatitis C	Others	≥18 year of age
Ruzicka 2018	Comorbidities and co-medications in populations with and without chronic hepatitis C virus infection in Japan between 2015 and 2016	MDV		Nationwide		2015	2016	Clinical epidemiology, course of diseases	Medical treatment status	Certain infectious and parasitic diseases		chronic hepatitis C virus (B18.2)	Others	aged ≥18 years
Hirata 2017	Surveillance rates for hepatocellular carcinoma among patients with cirrhosis, chronic hepatitis B, and chronic hepatitis C based on Japanese claims database	JMDC		Nationwide		2011	2011	Medical treatment status	Quality of care	Certain infectious and parasitic diseases	Diseases of the digestive system	LC, hepatitis B virus (HBV), or hepatitis C virus (HCV) infection		age group
Ohisa 2015	Estimated numbers of patients with liver disease related to hepatitis B or C virus infection based on the database reconstructed from medical claims from 2008 to 2010 in Japan	JMDC		Nationwide		2008	2010	Clinical epidemiology, course of diseases		Certain infectious and parasitic diseases		hepatitis viruses	Others	aged under 65 years
Udagawa 2015	Using Clinical Databases to Verify the Impact of Regulatory Agency Alerts in Japan: Hepatitis B Testing Behavior After an Alert Regarding Risk of Viral Reactivation	MDV		Nationwide		2010	2012	Health policy evaluation and utilization		Certain infectious and parasitic diseases		hepatitis B		
Sako 2011	Acute hepatitis B in Japan: Incidence, clinical practices and health policy	DPC		Nationwide		2007	2008	Clinical epidemiology, course of diseases	Medical treatment status	Certain infectious and parasitic diseases		acute hepatites B		
Yotsuyanagi 2021	Characteristics and healthcare costs in the aging hepatitis B population of Japan: A nationwide real-world analysis	MDV		Nationwide		2012	2016	Clinical epidemiology, course of diseases	Health economics	Certain infectious and parasitic diseases		Hepatitis B (ICD-10: B18.1)	Others	aged 18 years or older
Imai 2019	Validity of administrative database detection of previously resolved hepatitis B virus in Japan	Others	NHO, chart abstraction	Others	4 hospitals	2011	2015	Research methodology		Certain infectious and parasitic diseases	Diseases of the musculoskeletal system and connective tissue	hepatitis B virus, rheumatoid arthritis		
Fujita 2018	Hepatitis B virus reactivation in patients with rheumatoid arthritis: Analysis of the National Database of Japan	NDB		Nationwide		2013	2014	Clinical epidemiology, course of diseases	Quality of care	Certain infectious and parasitic diseases	Diseases of the musculoskeletal system and connective tissue	hepatitis B virus, rheumatoid arthritis	Others	patients aged 30- 79 years

Hagiwara 2015	The effectiveness of risk communication regarding drug safety information: a nationwide survey by the Japanese public health insurance claims data	NDB	national health insurance claims database (NHICD)	Nationwide		2010	2010	Health policy evaluation and utilization		Certain infectious and parasitic diseases	Diseases of the musculoskeletal system and connective tissue	hepatitis, rheumatoid arthritis		
Honda 2017	Treatment patterns of postherpetic neuralgia patients before and after the launch of pregabalin and its effect on medical costs: Analysis of Japanese claims data provided by Japan Medical Data Center	JMDC		Nationwide		2005	2013	Health policy evaluation and utilization	Health economics	Certain infectious and parasitic diseases		postherpetic neuralgia	Others	aged 18 years or older
Imafuku 2020	Risk of herpes zoster in the Japanese population with immunocompromising and chronic disease conditions: Results from a claims database cohort study, from 2005 to 2014	JMDC		Nationwide		2005	2014	Clinical epidemiology, course of diseases		Certain infectious and parasitic diseases	Others	herpes zoster (with immunocompromising and chronic disease conditions)	Others	≥18 year of age
Imafuku 2019	Burden of Herpes Zoster in the Japanese Population with Immunocompromised/Chronic Disease Conditions: Results from a Cohort Study Claims Database from 2005-2014	JMDC		Nationwide		2005	2014	Clinical epidemiology, course of diseases	Health economics	Certain infectious and parasitic diseases		Herpes Zoster	Others	aged 18–74 years
Ruzicka 2018	Comorbidities and the use of comedications in people living with HIV on antiretroviral therapy in Japan: a cross-sectional study using a hospital claims database	MDV		Nationwide		2010	2015	Clinical epidemiology, course of diseases	Medical treatment status	Certain infectious and parasitic diseases		HIV	Others	aged ≥18 years
Ruzicka 2019	Greater burden of chronic comorbidities and co-medications among people living with HIV versus people without HIV in Japan: A hospital claims database study	MDV		Nationwide		2010	2015	Clinical epidemiology, course of diseases	Medical treatment status	Certain infectious and parasitic diseases		HIV disease (ICD-10 codes B20–24)	Others	≥18 year of age
Ruzicka 2019	Switch rates, time-to-switch, and switch patterns of antiretroviral therapy in people living with human immunodeficiency virus in Japan, in a hospital-claim database	MDV		Nationwide		2008	2016	Medical treatment status		Certain infectious and parasitic diseases		HIV disease	Others	aged ≥18 years
Mizukami 2018	Resource Use and Cost of Treating Human Papillomavirus-Related Lesions in Japanese Women	Others	JMDC, MDV	Nationwide		2011	2011	Health economics		Certain infectious and parasitic diseases	Diseases of the genitourinary system	Human Papillomavirus–Related Lesions	Others	aged 15 years or older
Kishimoto 2021	Early impact of school closure and social distancing for COVID-19 on the number of inpatients with childhood non-COVID-19 acute infections in Japan	DPC	QIP	Nationwide		2018	2020	Health policy evaluation and utilization	COVID-19	Certain infectious and parasitic diseases	Diseases of the respiratory system	non-COVID-19 acute infections	Children	aged 15 years or younger
Imai 2020	Comparison of interactions between warfarin and cephalosporins with and without the N-methyl-thio-tetrazole side chain	JMDC		Nationwide		2010	2017	Intervention effect		Certain infectious and parasitic diseases	Diseases of the circulatory system	received warfarin during cephalosporin therapy, infections	Others	≥18 year of age
Nakamura 2015	Proposition of real-time precise prediction model of infectious disease patients from Prescription Surveillance using the National Database of Electronic Medical Claims	NDB	Aggregated data: Prescription Surveillance	Nationwide		2010	2013	Prediction model	Clinical epidemiology, course of diseases	Certain infectious and parasitic diseases		infectious disease		all ages
Saita 2013	[Incidence of Infectious Spondylitis Is Increasing Rapidly : Epidemiological Analysis Using the Japanese Diagnosis Procedure Combination Database]	DPC		Nationwide		2007	2009	Clinical epidemiology, course of diseases		Certain infectious and parasitic diseases	Diseases of the musculoskeletal system and connective tissue	Infectious Spondylitis		
Yasunaga 2010	Measles-related hospitalizations and complications in Japan, 2007-2008	DPC	DPC Research Group	Nationwide		2007	2008	Clinical epidemiology, course of diseases		Certain infectious and parasitic diseases		measles and related complicates		
Tanihara 2010	Evaluating measles surveillance: comparison of sentinel surveillance, mandatory notification, and data from health insurance claims	Others	several corporate health insurance societies in Aichi Prefecture, sentinel surveillance system, mandatory notification system run by the Aichi	Prefecture	Aichi	2007	2007	Clinical epidemiology, course of diseases	Research methodology	Certain infectious and parasitic diseases		measles		age groups
Yuasa 2019	Treatment procedures and associated medical costs of methicillin-resistant Staphylococcus aureus infection in Japan: A retrospective analysis using a database of Japanese employment-based health insurance	Others	MinaCare database	Nationwide		2010	2015	Medical treatment status	Health economics	Certain infectious and parasitic diseases		methicillin-resistant Staphylococcus aureus infection	Others	aged ≥15 years
Imai 2020	Association of the ward pharmacy service with active implementation of therapeutic drug monitoring for vancomycin and teicoplanin-an epidemiological surveillance study using Japanese large health insurance claims database	JMDC		Nationwide		2012	2017	Intervention effect	Medical treatment status	Certain infectious and parasitic diseases		MRSA		
Uematsu 2018	Effect of methicillin-resistant Staphylococcus aureus in Japan	DPC	QIP: DPC data linked to microbiologic data	Nationwide		2014	2016	Clinical epidemiology, course of diseases	Health economics	Certain infectious and parasitic diseases		Methicillin-resistant Staphylococcus aureus		
Uematsu 2017	Estimating the disease burden of methicillin-resistant Staphylococcus aureus in Japan: Retrospective database study of Japanese hospitals	DPC		Nationwide		2014	2015	Clinical epidemiology, course of diseases	Health economics	Certain infectious and parasitic diseases		MRSA		age categories
Tanihara 2016	Estimation of the incidence of MRSA patients: evaluation of a surveillance system using health insurance claim data	DPC	and JANIS data	Nationwide		2011	2012	Clinical epidemiology, course of diseases		Certain infectious and parasitic diseases		MRSA		
Goto 2014	[Clinical Evaluation of Therapeutic Drug Monitoring (TDM) on Antibiotic Therapy for Methicillin-resistant Staphylococcus aureus (MRSA) Infection with the Diagnosis Procedure Combination (DPC) System]	DPC		Nationwide		2010	2010	Intervention effect	Medical treatment status	Certain infectious and parasitic diseases		MRSA		
Ueno 2021	The disease burden of mucormycosis in Japan: results from a systematic literature review and retrospective database study	MDV	a systematic literature review	Nationwide		2010	2019	Clinical epidemiology, course of diseases	Health economics	Certain infectious and parasitic diseases		mucormycosis (ICD-10 code: B46.x)		
Ohfuji 2020	Mumps-related Disease Burden in Japan: Analysis of JMDC Health Insurance Reimbursement Data for 2005-2017	JMDC		Nationwide		2005	2017	Clinical epidemiology, course of diseases		Certain infectious and parasitic diseases		mumps	Others	3 month-64 year of age
Takagi 2020	Incidence of Mumps Deafness in Japan, 2005-2017: Analysis of Japanese Insurance Claims Database	JMDC		Nationwide		2005	2017	Clinical epidemiology, course of diseases		Certain infectious and parasitic diseases		Mumps Deafness	Others	3 month-64 year of age
Iwao 2020	A survey of clarithromycin monotherapy and long-term administration of ethambutol for patients with MAC lung disease in Japan: A retrospective cohort study using the database of health insurance claims	JMDC		Nationwide		2005	2017	Medical treatment status		Certain infectious and parasitic diseases		Mycobacterium avium-intracellulare complex lung disease		
Iwao 2020	Treatment of Mycobacterium avium–intracellulare complex lung disease in the real world: a retrospective big data analysis	JMDC		Nationwide		2007	2017	Medical treatment status		Certain infectious and parasitic diseases		Mycobacterium avium-intracellulare complex lung disease	Others	0-74 year of age
Izumi 2019	Epidemiology of Adults and Children Treated for Nontuberculous Mycobacterial Pulmonary Disease in Japan	NDB		Nationwide		2009	2014	Clinical epidemiology, course of diseases	Socioeconomic comparison	Certain infectious and parasitic diseases		nontuberculous mycobacterial pulmonary disease (A310 and A319)		
Igari 2020	A retrospective observational study of antimicrobial treatment for non-tuberculous mycobacteria disease using a nationwide claims database in Japan	NDB	Sampling data	Nationwide		2011	2014	Medical treatment status	Quality of care	Certain infectious and parasitic diseases		non-tuberculous mycobacteria		
Morimoto 2019	Actual practice of standard treatment for pulmonary nontuberculous mycobacteriosis in Japan	NDB	national health insurance database	Nationwide		2010	2014	Medical treatment status		Certain infectious and parasitic diseases		nontuberculous mycobacterial pulmonary disease		
Uno 2020	Comorbidities associated with nontuberculous mycobacterial disease in Japanese adults: a claims-data analysis	JMDC		Nationwide		2014	2014	Clinical epidemiology, course of diseases		Certain infectious and parasitic diseases		nontuberculous mycobacterial disease	Others	aged 20–75 years
Iwao 2018	[Survey of Drug Prescription Patterns Among Patients with Nontuberculous Mycobacterial Disease Using the Database of Health Insurance Claims]	JMDC		Nationwide		2015	2016	Medical treatment status		Certain infectious and parasitic diseases		Nontuberculous Mycobacterial Disease (A310-A319)		
Chang 2018	The incidence of medically-attended norovirus gastro-enteritis in Japan: Modelling using a medical care insurance claims database	JMDC		Nationwide		2007	2015	Clinical epidemiology, course of diseases		Certain infectious and parasitic diseases		noro virus		age group
Imai 2018	Risk of pneumococcal diseases in adults with underlying medical conditions: a retrospective, cohort study using two Japanese healthcare databases	Others	JMDC, MDV	Nationwide		2006	2014	Clinical epidemiology, course of diseases		Certain infectious and parasitic diseases		Pneumococcal disease	Others	aged ≥19 years

Shoji 2020	Recent epidemiology of Pneumocystis pneumonia in Japan	DPC		Nationwide		2010	2016	Clinical epidemiology, course of diseases		Certain infectious and parasitic diseases		Pneumocystis pneumonia		
Inoue 2019	Adjunctive Corticosteroids decreased the risk of mortality of non-HIV Pneumocystis Pneumonia	DPC		Nationwide		2010	2016	Intervention effect		Certain infectious and parasitic diseases		non-HIV Pneumocystis pneumonia	Others	≥18 year of age
Fukuda 2020	Healthcare resources attributable to methicillin-resistant Staphylococcus aureus orthopedic surgical site infections	NDB		Nationwide		2012	2018	Health economics		Certain infectious and parasitic diseases	Injury, poisoning and certain other consequences of external causes	Surgical Site Infection (MRSA)		
Okubo 2018	Dose-dependent relationships between weight status and clinical outcomes among infants hospitalized with respiratory syncytial virus infections	DPC		Nationwide		2010	2015	Clinical epidemiology, course of diseases		Certain infectious and parasitic diseases	Diseases of the respiratory system	Respiratory syncytial virus (RSV) infection	Children	aged <12 months
Kobayashi 2020	Sustained reduction in rotavirus-coded hospitalizations in children aged <5 years after introduction of self-financed rotavirus vaccines in Japan	JMDC		Nationwide		2009	2017	Clinical epidemiology, course of diseases	Health policy evaluation and utilization	Certain infectious and parasitic diseases		rotavirus gastroenteritis	Children	<10 years of age
Kimura 2019	Passive surveillance of rotavirus gastroenteritis-associated hospitalization using nationwide administrative databases in Japan	Others	MDV, JMDC	Nationwide		2009	2016	Clinical epidemiology, course of diseases		Certain infectious and parasitic diseases		rotaviral enteritis	Children	＜6 year of age
Kobayashi 2018	Decline of rotavirus-coded hospitalizations in children under 5 years: A report from Japan where rotavirus vaccines are self-financed	JMDC		Nationwide		2009	2015	Health policy evaluation and utilization		Certain infectious and parasitic diseases		rota virus	Children	aged <5 years
Aso 2020	Dexmedetomidine and Mortality From Sepsis Requiring Mechanical Ventilation: A Japanese Nationwide Retrospective Cohort Study	DPC		Nationwide		2010	2016	Intervention effect		Certain infectious and parasitic diseases		sepsis	Others	aged ≥15 years
Shime 2011	[Epidemiology of pediatric sepsis in Japanese community hospitals: analysis using a diagnosis procedure combination database]	DPC	QIP	Nationwide		2007	2007	Clinical epidemiology, course of diseases	Medical treatment status	Certain infectious and parasitic diseases		pediatric sepsis		age group
Fujimori 2020	[Effects of Polymyxin B Hemoperfusion in Patients with Sepsis Requiring CHDF：Analysis of a Nationwide Administrative Database in Japan]	DPC		Nationwide		2016	2018	Intervention effect		Certain infectious and parasitic diseases		Sepsis	Others	aged ≥20 years
Tanihara 2014	Assessment of medical expenditures for sepsis:differentiating between cases with and without ruled-out diagnoses	Others	an employee health insurance organization	Others	an employee health insurance organization	2006	2007	Medical treatment status	Health economics	Certain infectious and parasitic diseases		sepsis		
Fujimori 2021	Effects of Polymyxin B Hemoperfusion in Patients with Sepsis Requiring Continuous Hemodiafiltration: Analysis of a Nationwide Administrative Database in Japan	DPC		Nationwide		2016	2019	Intervention effect		Certain infectious and parasitic diseases		sepsis	Others	≥20 years of age
Edakubo 2020	Effect of Early Central Venous Catheterization On Mortality Among Patients with Severe Sepsis: A Nationwide Inpatient Database Study	DPC		Nationwide		2014	2016	Intervention effect		Certain infectious and parasitic diseases		severe sepsis	Others	≥18 year of age
Miyauchi 2012	Treatment with PMX-DHP improves prognoses of sepsis patients who required treatment with blood purification and is favorable for the medical fees	Others	Social Insurance Hospitals	Others	30 Social Insurance Hospital	2008	2011	Intervention effect	Health economics	Certain infectious and parasitic diseases		Sepsis		
Igari 2020	A retrospective observational study of antibiotics treatment for sepsis using a nationwide claim database in Japan	NDB	Sampling data	Nationwide		2011	2014	Medical treatment status	Clinical epidemiology, course of diseases	Certain infectious and parasitic diseases	Others	antibiotics treatment for sepsis	Others	aged 15 years and older
Ohbe 2019	Intravenous albumin for initial resuscitation and mortality in septic shock patients: propensity score analyses using a nationwide inpatient database	DPC		Nationwide		2010	2016	Intervention effect		Certain infectious and parasitic diseases		septic shock	Others	age ≥20 years
Tagami 2015	Low-dose corticosteroid treatment and mortality in refractory abdominal septic shock after emergency laparotomy	DPC		Nationwide		2010	2013	Intervention effect		Certain infectious and parasitic diseases	Diseases of the digestive system	refractory abdominal septic shock after emergency laparotomy	Others	age ≥15 years
Suzuki 2020	Histamine-2 receptor antagonists versus proton pump inhibitors for septic shock after lower gastrointestinal tract perforation: a retrospective cohort study using a national inpatient database	DPC		Nationwide		2010	2015	Intervention effect		Certain infectious and parasitic diseases	Diseases of the digestive system	septic shock after lower gastrointestinal tract	Others	≥18 year of age
Tagami 2015	Intravenous immunoglobulin and mortality in pneumonia patients with septic shock: an observational nationwide study	DPC		Nationwide		2010	2013	Intervention effect		Certain infectious and parasitic diseases	Diseases of the digestive system	pneumonia, septic shock	Others	age ≥18 years
Fujimori 2021	Effects of Polymyxin B Hemoperfusion on Septic Shock Patients Requiring Noradrenaline: Analysis of a Nationwide Administrative Database in Japan	DPC		Nationwide		2016	2019	Intervention effect		Certain infectious and parasitic diseases		septic shock	Others	aged ≥20 years
Iwagami 2016	Potential Survival Benefit of Polymyxin B Hemoperfusion in Septic Shock Patients on Continuous Renal Replacement Therapy: A Propensity-Matched Analysis	DPC		Nationwide		2007	2012	Intervention effect		Certain infectious and parasitic diseases	Diseases of the genitourinary system	Septic shock, Continuous Renal Replacement Therapy	Others	aged ≥18 years
Tagami 2015	Intravenous immunoglobulin use in septic shock patients after emergency laparotomy	DPC		Nationwide		2010	2013	Intervention effect		Certain infectious and parasitic diseases	Injury, poisoning and certain other consequences of external causes	septic shock, intestinal perforation	Others	aged 15-89 years
Tanihara 2011	[Investigation of descriptions of sexually transmitted diseases in medical fee statements]*	Others	multiple health insurance society	Nationwide		2006	2006	Clinical epidemiology, course of diseases		Certain infectious and parasitic diseases		sexually transmitted disease		
Matsubayashi 2020	Syphilis testing among spouses of patients with syphilis in Japan: an epidemiological study using an administrative claims database	JMDC		Nationwide		2010	2017	Medical treatment status		Certain infectious and parasitic diseases		syphilis	Others	20-74 years of age
Nakajima 2018	Clinical features and outcomes of tetanus: Analysis using a National Inpatient Database in Japan	DPC		Nationwide		2010	2016	Clinical epidemiology, course of diseases		Certain infectious and parasitic diseases		tetanus neonatorum (A33.1), obstetric tetanus (A34.10), or other tetanus (A35)		
Yasunaga 2011	Delay in tetracycline treatment increases the risk of complications in Tsutsugamushi disease: data from the Japanese Diagnosis Procedure Combination database	DPC	DPC Research Group	Nationwide		2007	2008	Clinical epidemiology, course of diseases	Medical treatment status	Certain infectious and parasitic diseases		Tsutsugamushi disease (A75.3)		
Tobise 2010	[A DRG/PPS simulation in the medical care of tuberculosis]	Others	19 hospitals under the National Hospital Organization	Nationwide		2007	2008	Health economics		Certain infectious and parasitic diseases		tuberculosis		
Yamana 2021	Treatment of latent tuberculosis infection in patients receiving biologic agents	JMDC		Nationwide		2005	2018	Medical treatment status		Certain infectious and parasitic diseases	Others	immune-mediated inflammatory diseases, tuberculosis	Others	aged 20-64 years
Iba 2020	Tuberculosis screening and management of latent tuberculosis infection prior to biologic treatment in patients with immune-mediated inflammatory diseases: A longitudinal population-based analysis using claims data	NHI/LSEHS	NHI	Municipality	C city, Tokyo prefecture	2012	2019	Medical treatment status	Quality of care	Certain infectious and parasitic diseases	Others	immunemediated inflammatory diseases, Tuberculosis	Others	15-74 year of age
Yamada 2021	Pneumococcal vaccine coverage in Japan among patients with a history of splenectomy: Results of a retrospective administrative database study	JMDC		Nationwide		2005	2019	Medical treatment status		Certain infectious and parasitic diseases	Others	overwhelming post-splenectomy infection	Others	aged 2-64 years
Nakamura 2015	Evaluation of the Number of Varicella Patients Estimated by Prescription Surveillance	NDB	Aggregated data: Prescription Surveillance	Nationwide		2010	2013	Clinical epidemiology, course of diseases	Research methodology	Certain infectious and parasitic diseases		Varicella		
Sugawara 2011	[Chickenpox case estimation in acyclovir pharmacy survey and early bioterrorism detection]	Others	Pharmacy surveillance	Nationwide		2010	2010	Clinical epidemiology, course of diseases		Certain infectious and parasitic diseases		chickenpox		age groups
2) Neoplasms (C00-D48)														

Yanaihara 2011	[Current state of surgical therapy for adrenal tumors based on DPC data from 2006 to 2008: A comparison between urology and surgery]*	DPC	DPC Research Group	Nationwide		2006	2008	Medical treatment status		Neoplasms		adrenalectomy cases		
Okamoto 2013	Prevalence of bile duct cancer among printing industry workers in comparison with other industries	JHIA	Japan Health Insurance Association (JHIA)	Nationwide		2009	2012	Clinical epidemiology, course of diseases		Neoplasms		Bile Duct Cancer (C22.1 and C24.0)		
Sugihara 2014	Comparison of perioperative outcomes including severe bladder injury between monopolar and bipolar transurethral resection of bladder tumors: a population based comparison	DPC		Nationwide		2007	2012	Intervention effect		Neoplasms	Injury, poisoning and certain other consequences of external causes	bladder tumor, bladder injury		
Shinjo 2019	Volume effect in paediatric brain tumour resection surgery: analysis of data from the Japanese national inpatient database	DPC		Nationwide		2012	2016	Clinical epidemiology, course of diseases	Health policy evaluation and utilization	Neoplasms		brain tumour	Children	aged 15 years old and younger
Uchida 2012	Impact of remifentanil use on early postoperative outcomes following brain tumor resection or rectal cancer surgery	DPC		Nationwide		2007	2007	Intervention effect		Neoplasms		brain tumor or rectal cancer		
Yoshimoto 2016	Current Trends and Healthcare Resource Usage in the Hospital Treatment of Primary Malignant Brain Tumor in Japan: A National Survey Using the Diagnostic Procedure Combination Database (J-ASPECT Study-Brain Tumor)	DPC	J-ASPECT Study-Brain Tumor	Nationwide		2013	2014	Medical treatment status	Health economics	Neoplasms		primary malignant brain tumors		
Yoshimoto 2015	Visionary approach for the treatment of brain tumors	DPC	J-ASPECT Study	Nationwide		2013	2014	Clinical epidemiology, course of diseases	Medical treatment status	Neoplasms		brain tumors		
Moriwaki 2021	Economic Evaluation of First-Line Pertuzumab Therapy in Patients with HER2-Positive Metastatic Breast Cancer in Japan	JMDC	CLEOPATRA trial, published sources	Nationwide		2005	2017	Health economics		Neoplasms		receptor-2 (HER2)-positive breast cancer	Others	elderly people (aged >75 years) are not included
Tajima 2021	Real-world anticancer medications for reproductive-age women with breast cancer by using a claims database in Japan	JMDC		Nationwide		2005	2018	Medical treatment status		Neoplasms		Brest cancer (ICD-10code: C50)	Others	reproductive age (15 to 49 years)
Ishii 2020	Epidemiology and practice patterns for male breast cancer compared with female breast cancer in Japan	DPC	HBCR+DPC	Nationwide		2012	2015	Clinical epidemiology, course of diseases	Medical treatment status	Neoplasms		breast cancer patients (ICD-O-3)		
Matsumoto 2020	Association between variations in the number of hospital beds and inpatient chemo/radiotherapy for breast cancer: a study using a large claim database	DPC		Nationwide		2012	2016	Health policy evaluation and utilization		Neoplasms		breast cancer patients		
Sakata 2019	Use of a Healthcare Claims Database for Post-Marketing Safety Assessments of Eribulin in Japan: A Comparative Assessment with a Prospective Post-Marketing Surveillance Study	MDV		Nationwide		2011	2016	Health policy evaluation and utilization		Neoplasms		breast cancer		
Sato 2015	The accuracy of Japanese claims data in identifying breast cancer cases	Others	claims data, standardized hospital-based cancer registry	Others	a single institution	2011	2011	Research methodology		Neoplasms		breast cancer	Others	
Goto 2017	Cost analysis of leuprorelin acetate in Japanese pre-menopausal breast-cancer patients: comparison between 6-month and 3-month depot formulations	MDV	web-based survey to determine the intangible costs	Nationwide		2008	2016	Health economics		Neoplasms		pre-menopausal breast cancer		
Iwagami 2021	Risk of Cancer in Association with Ranitidine and Nizatidine vs Other H2 Blockers: Analysis of the Japan Medical Data Center Claims Database 2005-2018	JMDC		Nationwide		2005	2018	Intervention effect		Neoplasms		ICD-10 C00–C96 (excluding C44)	Others	excluded patients aged < 18 years
Makito 2020	Volatile versus Total Intravenous Anesthesia for Cancer Prognosis in Patients Having Digestive Cancer Surgery	DPC		Nationwide		2010	2018	Intervention effect		Neoplasms		patients having digestive cancer surgery	Others	age of greater than or equal to 18 yr
Morishima 2020	Geriatric assessment domains to predict overall survival in older cancer patients: An analysis of functional status, comorbidities, and nutritional status as prognostic factors	DPC	Osaka Cancer Registry + DPC	Prefecture	Osaka	2010	2014	Prediction model	Clinical epidemiology, course of diseases	Neoplasms		gastric, colorectal, and lung cancer (ICD-O-3: C16, C18, C19, C20, C33, C34)	Older persons	patients aged 70 years or olde
Okuyama 2020	Usability of Clinical Information in Discharge Summary Data in the Diagnosis Procedure Combination Survey for Cancer Patients	DPC	HBCR + DPC	Nationwide		2012	2014	Research methodology		Neoplasms		stomach, colorectal, liver, lung, or breast cancer	Others	over the age of 20 years
Yazaki 2020	High hepatitis B virus screening rate among patients receiving systemic anticancer treatment in Japan	DPC	Hospital-based Cancer Registries + DPC	Nationwide		2013	2015	Medical treatment status		Neoplasms		patients diagnosed with cancer	Others	aged 20 and older
Morishima 2020	[Proportion of cancer detected by screening and early-stage cancer among cancer patients by type of medical insurance and family members]*	DPC	Osaka Cancer Registry + DPC	Prefecture	Osaka	2010	2015	Socioeconomic comparison		Neoplasms		stomach cancer, colon cancer, lung cancer, breast cancer, cervical cancer	Others	aged 40-59 years (cervical cancer: 20-59 years)
Bun 2019	Analysis of concordance with antiemetic guidelines in pediatric, adolescent, and young adult patients with cancer using a large-scale administrative database	DPC		Nationwide		2010	2016	Quality of care		Neoplasms		patients who were diagnosed with any cancer (ICD10 codes: C00-D48)	Others	age <30 years
Ishimaru 2019	Association between perioperative oral care and postoperative pneumonia after cancer resection: conventional versus high-dimensional propensity score matching analysis	JMDC		Nationwide		2014	2015	Intervention effect		Neoplasms		head and neck, lung, gastric, colorectal, breast, and esophageal cancer		
Momo 2019	Assessment of "look-alike" packaging designs related to medication errors using information technology	JMDC		Nationwide		2016	2016	Medical treatment status		Neoplasms		cancer patients	Others	aged 0–74 years
Yamada 2019	A descriptive epidemiological study on the treatment options for head and neck cancer: Transition before and after approval of cetuximab	MDV		Nationwide		2008	2015	Medical treatment status	Health policy evaluation and utilization	Neoplasms		lip and oral cancer, nasopharyngeal cancer, oropharyngeal cancer, hypopharyngeal cancer, laryngeal cancer, and nasal/paranasal sinus cancer (ICD10 codes: C00 - C06, C09 - C14, C30.0, C31, and C32)		
Yokoyama 2019	Integrative analysis of clinical and bioinformatics databases to identify anticancer properties of digoxin	JMDC		Nationwide		2005	2015	Intervention effect		Neoplasms		gastric cancer, colorectal cancer, prostate cancer and haematological malignancy		
Ishimaru 2018	Preoperative oral care and effect on postoperative complications after major cancer surgery	NDB		Nationwide		2012	2015	Intervention effect		Neoplasms		head and neck ,oesophageal,gastric,colorectal,lung or liver cancer	Others	Those aged <18 years were excluded
Sato 2018	Prevalence and initial prescription of psychotropics in patients with common cancers in Japan, based on a nationwide health insurance claims database	JMDC		Nationwide		2009	2014	Medical treatment status		Neoplasms		breast, colorectal, liver, lung, ovarian, pancreatic, prostate, or stomach cancer	Others	aged ≥18 years
Sato 2018	End-of-life care for cancer patients in Japanese acute care hospitals: A nationwide retrospective administrative database survey	DPC		Nationwide		2011	2014	Medical treatment status		Neoplasms		malignant neoplasm (C00-97)	Others	age ≥20 years
Urushiyama 2018	Effect of Hangeshashin-To (Japanese Herbal Medicine Tj-14) on Tolerability of Irinotecan: Propensity Score and Instrumental Variable Analyses	DPC		Nationwide		2010	2016	Intervention effect		Neoplasms		patients with a diagnosis of neoplasm defined by ICD10		
Matsuda 2018	[Big data analysis of medical and nursing care: An examination of days of the week for cancer outpatient chemotherapy]*	Others	Japan Health Insurance Association, National Health Insurance, Latter-stage elderly healthcare system	Others	A municipality in Western Japan	2013	2015	Medical treatment status		Neoplasms		patients received outpatient chemotherapy		

Okuyama 2017	Prescription of Prophylactic Antiemetic Drugs for Patients Receiving Chemotherapy With Minimal and Low Emetic Risk	DPC	linked with the hospital-based cancer registry	Nationwide		2010	2012	Medical treatment status		Neoplasms	breast, lung, colorectal, stomach, cervical, or prostate cancer	Others	20 years older
Ikeda 2016	Screening rate for hepatitis B virus infection in patients undergoing chemotherapy in Japan	JMDC		Nationwide		2008	2013	Medical treatment status		Neoplasms	cancer patients scheduled for chemotherapy		
Iwamoto 2016	Monitoring and evaluating the quality of cancer care in Japan using administrative claims data	DPC	HBCR+DPC	Nationwide		2010	2012	Quality of care		Neoplasms	colorectal, lung, stomach, liver, breast, prostate and cervical cancer patients		
Kunisawa 2016	Reducing Length of Hospital Stay Does Not Increase Readmission Rates in Early-Stage Gastric, Colon, and Lung Cancer Surgical Cases in Japanese Acute Care Hospitals	DPC		Nationwide		2010	2014	Quality of care		Neoplasms	gastric, colon, and lung cancer surgical patients		
Takada 2016	Inverse Association between Sodium Channel-Blocking Antiepileptic Drug Use and Cancer: Data Mining of Spontaneous Reporting and Claims Databases	JMDC		Nationwide		2005	2014	Intervention effect		Neoplasms	bladder cancer, lung cancer, pancreatic cancer, gastric cancer, esophageal cancer, hematological malignancies, melanoma, breast cancer, and prostate cancer		
Takaoka 2016	Staging discrepancies between Hospital-Based Cancer Registry and Diagnosis Procedure Combination data	DPC	HBCR and DPC	Others	four voluntarily participating hospitals	2011	2011	Research methodology		Neoplasms	tumor–node–metastasis classification		
Tsukada 2016	Patterns of prescribing radiotherapy and bevacizumab in nationwide practice-analysis of 101 designated cancer care hospitals in Japan	DPC	HBCR+DPC	Nationwide		2011	2012	Medical treatment status		Neoplasms	patients who received both externalbeam radiotherapy and bevacizumab		
Uda 2016	[The current status of cancer rehabilitation in Japan : an analysis from diagnosis procedure combination database]	DPC		Nationwide		2011	2014	Medical treatment status	Health policy evaluation and utilization	Neoplasms	cancer rehabilitation		
Sato 2016	[A Preliminary Survey to Measure the Quality Indicators of End-of-life Cancer Care Using the Japanese National Database]	NDB	Sampling data	Nationwide		2012	2012	Quality of care	Research methodology	Neoplasms	cancer patients	Others	excluded patients aged <20 years
Fujimoto 2015	Association between statin use and cancer: data mining of a spontaneous reporting database and a claims database	JMDC		Nationwide		2005	2013	Intervention effect		Neoplasms	lung cancer, pancreatic cancer, gastric cancer, esophageal cancer, breast cancer, hematological malignancies, melanoma, and prostate cancer		
Iwamoto 2015	Accuracy of using Diagnosis Procedure Combination administrative claims data for estimating the amount of opioid consumption among cancer patients in Japan	DPC	compared with electronic medical records	Others	Aomori Prefectural Central Hospital	2012	2013	Research methodology		Neoplasms	opioid consumption among cancer patients		
Tanaka 2015	[Cancer treatment situation in Japan with regard to the type of medical facility using medical claim data of Health Insurance Societies]	JMDC		Nationwide		2005	2011	Patient health service utilization		Neoplasms	stomach, colon, liver, lung, or breast cancer		
Morishima 2014	Association of healthcare expenditures with aggressive versus palliative care for cancer patients at the end of life: a cross-sectional study using claims data in Japan	NHI/LSEHS	National Health Insurance (NHI) and Long Life Medical Care System	Prefecture	Kyoto	2009	2010	Health economics		Neoplasms	Lung, Stomach, Pancreas, Prostate, Breast, Colorectum, Liver, Biliary tract, blood, Other		
Okuyama 2014	Prescription trends of prophylactic antiemetics for chemotherapy-induced nausea and vomiting in Japan	JMDC		Nationwide		2005	2011	Medical treatment status		Neoplasms	those who recceived chemotherapy with high or moderate emetic risks	Others	patients 18 years old or older
Shibata 2014	[Usefulness of a healthcare insurance claims database for statistical data in cancer patients]*	NDB	Patient Survey (MHLW)	Nationwide		2010	2011	Research methodology	Clinical epidemiology, course of diseases	Neoplasms	cancer patients		
Watanabe 2014	[Estimating duplicate registration rates in hospital cancer registries]*	JMDC		Nationwide		2005	2010	Clinical epidemiology, course of diseases	Research methodology	Neoplasms	lung, stomach, liver, breast, colon cancer		
Morishima 2013	Impact of hospital case volume on quality of end-of-life care in terminal cancer patients	NHI/LSEHS	National Health Insurance (NHI) and Long Life Medical Care System	Prefecture	Kyoto	2009	2010	Quality of care	Health policy evaluation and utilization	Neoplasms	died of cancers		
Nakamura 2013	Pattern of prophylaxis administration for chemotherapy-induced nausea and vomiting: an analysis of city-based health insurance data	NHI/LSEHS	a city-based insurance program	Municipality	Kure city, Hiroshima	2008	2010	Medical treatment status		Neoplasms	who received any of these chemotherapy drugs	Others	age 20 or older and under 75
Hamada 2013	[Application of Japanese Claims Database to Pharmacovigilance Activity in Pharmaceutical Industry; Analysis on Cancer Incidences and Usage of Anticancer Agents]	JMDC		Nationwide		2011	2011	Health policy evaluation and utilization		Neoplasms	cancer patients		
Higashi 2012	Prevalence of analgesic prescriptions among patients with cancer in Japan: an analysis of health insurance claims data	JMDC		Nationwide	from 8 employer-sponsored insurance companies	2005	2009	Medical treatment status		Neoplasms	breast, colorectal, stomach, lung, and liver cancers	Others	below 75 years old
Yasunaga 2012	Variation in cancer surgical outcomes associated with physician and nurse staffing: a retrospective observational study using the Japanese Diagnosis Procedure Combination Database	DPC		Nationwide		2007	2008	Health policy evaluation and utilization		Neoplasms	lung, esophageal, gastric, colorectal, and pancreatic cancer		
Maeda 2012	[Data Analysis Method for More Efficient Integration of Cancer Treatment Centers and Secondary Medical Service Areas]	NHI/LSEHS	Fukuoka	Prefecture	Fukuoke	2008	2010	Health policy evaluation and utilization		Neoplasms	gastric, colorectal, liver, lung and breast cancers		
Kuroda 2020	Survey on off-label use of antineoplastic agents in pediatric patients using a large-scale medical database in japan	MDV		Nationwide		2016	2017	Medical treatment status		Neoplasms	off-label use of antineoplastic agents in pediatric patients	Children	0–14 years of age
Minoura 2018	Practice Patterns of Medications for Patients With Malignant Bowel Obstruction Using a Nationwide Claims Database and the Association Between Treatment Outcomes and Concomitant Use of H2-Blockers/Proton Pump Inhibitors and Corticosteroids With Octreotide	MDV		Nationwide		2010	2015	Medical treatment status	Intervention effect	Neoplasms	malignant bowel obstruction impairs, intraabdominal cancers	Others	aged ≥20 years

Sugihara 2013	Regional, institutional and individual factors affecting selection of minimally invasive nephroureterectomy in Japan: a national database analysis	DPC		Nationwide		2007	2010	Medical treatment status	Socioeconomic comparison	Neoplasms		nephroureterectomy, malignant neoplasms of the renal pelvis or ureter		
Mimura 2020	Antimicrobial utilization and antimicrobial resistance in patients with haematological malignancies in Japan: a multi-centre cross-sectional study	DPC	JANIS data	Nationwide		2015	2017	Medical treatment status		Neoplasms	Certain infectious and parasitic diseases	haematological malignancies, antimicrobials		
Kishi 2020	Impact of Behavioral Health Comorbidities on Health Care Costs Among Japanese Patients With Cancer	JMDC		Nationwide		2014	2015	Health economics		Neoplasms	Mental, Behavioral and Neurodevelopmental disorders	Cancer, behavioural health		
Morita 2017	Association between Nurse Staffing and In-Hospital Bone Fractures: A Retrospective Cohort Study	DPC		Nationwide		2010	2014	Health policy evaluation and utilization		Neoplasms	Diseases of the circulatory system	cancer or cardiovascular diseases	Others	aged 50 years or older
Yamaji 2016	Association Between Colorectal Cancer and Atherosclerotic Diseases: A Study Using a National Inpatient Database in Japan	DPC		Nationwide		2010	2012	Clinical epidemiology, course of diseases		Neoplasms	Diseases of the circulatory system	Colorectal cancer and atherosclerotic diseases		age groups
Akechi 2020	Treatment of Major Depressive Disorder in Japanese Patients with Cancer: A Matched Cohort Study Using Employer-Based Health Insurance Claims Data	JMDC		Nationwide		2012	2017	Medical treatment status		Neoplasms	Mental, Behavioral and Neurodevelopmental disorders	1) Newly diagnosis with cancer (ICD10: C00-95), 2) major depressive disorder (F32 F33)	Others	adult (aged 18-74 years)
Akechi 2020	Risk of major depressive disorder in spouses of cancer patients in Japan: A cohort study using health insurance-based claims data	JMDC		Nationwide		2011	2018	Clinical epidemiology, course of diseases	Others	Neoplasms	Mental, Behavioral and Neurodevelopmental disorders	1) cancer (ICD10: C00-95), 2) major depressive disorder (F32 F33)		
Akechi 2020	Risk of major depressive disorder in Japanese cancer patients: A matched cohort study using employer-based health insurance claims data	JMDC		Nationwide		2012	2017	Clinical epidemiology, course of diseases		Neoplasms	Mental, Behavioral and Neurodevelopmental disorders	1) new diagnosis of cancer, 2) major depressive disorder (F32 F32)	Others	adult (aged 18-74 years)
Muramatsu 2013	Do the Japanese Cancer Patients Receive an Appropriate Psychiatric Support at the Acute Care Hospitals?: An Evaluation Trial by DPC Based Data	DPC		Nationwide		2010	2010	Medical treatment status	Clinical epidemiology, course of diseases	Neoplasms	Mental, Behavioral and Neurodevelopmental disorders	breast cancer, depression and anxiety symptom (as comorbidity or complication)		
Matsuda 2010	[Cancer and psychiatric care: From the results of a study based on DPC data]*	DPC	DPC Research Group	Nationwide		2008	2008	Medical treatment status	Clinical epidemiology, course of diseases	Neoplasms	Mental, Behavioral and Neurodevelopmental disorders	breast cancer, depressive disorders		
Taniguchi 2021	Recombinant Thrombomodulin in Disseminated Intravascular Coagulation Associated with Stage IV Solid Tumors: A Nationwide Observational Study in Japan	DPC		Nationwide		2010	2018	Intervention effect		Neoplasms	Diseases of the blood and blood-forming organs and certain disorders involving the immune mechanism	disseminated intravascular coagulation, solid tumors	Others	aged ≥18 years
Kobayashi 2015	The effect of centralization of health care services on travel time and its equality	JHIA	the Kyoto branch of the Japan Health Insurance Association	Prefecture	Kyoto	2008	2009	Health policy evaluation and utilization		Neoplasms	Diseases of the circulatory system	ischemic heart disease, breast cancer		
Kaneko 2020	Association of cancer with outcomes in patients hospitalized for heart failure	DPC		Nationwide		2010	2018	Clinical epidemiology, course of diseases		Neoplasms	Diseases of the circulatory system	heart failure (HF) patients with concomitant cancer	Others	aged ≥20 years
Mizuno 2021	Differences in aggressive treatments during the actively dying phase in patients with cancer and heart disease: an exploratory study using the sampling dataset of the National Database of Health Insurance Claims	NDB	Sampling data: limited to DPC dataset	Nationwide		2011	2015	Medical treatment status		Neoplasms	Diseases of the circulatory system	cancer and heart disease (aggressive treatments)		
Inaida 2020	Previous Infection Positively Correlates to the Tumor Incidence Rate of Patients with Cancer	JMDC		Nationwide		2005	2012	Clinical epidemiology, course of diseases		Neoplasms	Certain infectious and parasitic diseases	1) The case group was people who presented with malignant cancer, 2) influenza, gastroenteritis, hepatitis, and pneumonia infections	Others	patients ≥30 years of age
Sugihara 2015	Perioperative Outcome Comparisons Between Open and Laparoscopic Nephroureterectomy Among a Population-Based Cohort from 2010 to 2012	DPC		Nationwide		2010	2012	Intervention effect	Health economics	Neoplasms	Diseases of the genitourinary system	nephroureteroectomy for malignant diseases		
Sato 2017	Mortality and hemorrhagic complications associated with radiofrequency ablation for treatment of hepatocellular carcinoma in patients on hemodialysis for end-stage renal disease: A nationwide survey	DPC		Nationwide		2010	2012	Clinical epidemiology, course of diseases	Intervention effect	Neoplasms	Diseases of the genitourinary system	end-stage renal disease, hepatocellular carcinoma		
Watanabe 2018	Quality indicators for cervical cancer care in Japan	DPC	HBCR + DPC	Nationwide		2012	2014	Quality of care		Neoplasms		patients with cervical cancer (ICD-O morphology: C53.0–53.9)		
Kobayashi 2020	[Incidence of Proteinuria among Japanese Colorectal Cancer Patients after Receiving Ramucirumab-Cohort Study Using Claim Database]	MDV		Nationwide		2015	2018	Intervention effect		Neoplasms		Colorectal cancer patients (IDC10: C18-20)	Others	patients aged 18 years or older
Nakashima 2020	Effectiveness and Safety of Regorafenib vs. Trifluridine/Tipiracil in Unresectable Colorectal Cancer: A Retrospective Cohort Study	MDV		Nationwide		2013	2018	Intervention effect		Neoplasms		metastatic colorectal cancer	Others	aged over 20 years
Tajima 2020	Open colectomy vs. laparoscopic colectomy in Japan: a retrospective study using real-world data from the diagnosis procedure combination database	DPC		Nationwide		2014	2016	Intervention effect		Neoplasms		colorectal cancer (ICD10: C18 C19)		
Watanabe 2020	Preoperative rehabilitation for patients undergoing colorectal cancer surgery: a retrospective cohort study	DPC		Nationwide		2014	2017	Intervention effect		Neoplasms		colorectal cancer C18-20 (ICD10)		
Nakashima 2019	Comparison of Standard Initial Dose and Reduced Initial Dose Regorafenib for Colorectal Cancer Patients: A Retrospective Cohort Study	MDV		Nationwide		2013	2018	Intervention effect		Neoplasms		colorectal cancer	Others	aged over 20 years
Yamazaki 2018	Pre-emptive skin treatments to prevent skin toxicity caused by anti-EGFR antibody: the real-world evidence in Japan	MDV		Nationwide		2008	2017	Medical treatment status		Neoplasms		colorectal cance (ICD10: C18 C19 C20)		
Wei 2018	[The Diffusion Rate and Regional Difference of the Insured with Colorectal Cancer Outpatient Chemotherapy in Fukuoka Branch of Japan Health Insurance Association (FBJHIA)]	JHIA	Fukuoka Branch, National Health Insurance Association	Prefecture	Fukuoka	2014	2015	Medical treatment status	Socioeconomic comparison	Neoplasms		colorectal cancer		
Higuchi 2015	[THE INFLUENCE OF HOSPITAL VOLUME ON THE OUTCOME OF COLORECTAL CANCER RESECTIONS IN JAPAN]	DPC		Nationwide		2007	2008	Health policy evaluation and utilization		Neoplasms		colorectal cancer resections		
Yasunaga 2011	Effect of the Japanese herbal kampo medicine dai-kenchu-to on postoperative adhesive small bowel obstruction requiring long-tube decompression: a propensity score analysis	DPC	DPC Research Group	Nationwide		2007	2008	Intervention effect		Neoplasms		underwent radical surgery for colorectal cancer		
Niikura 2016	Factors predicting adverse events associated with therapeutic colonoscopy for colorectal neoplasia: a retrospective nationwide study in Japan	DPC		Nationwide		2013	2014	Clinical epidemiology, course of diseases	Intervention effect	Neoplasms		colorectal neoplasia		
Yamazaki 2021	Real-World Evidence on Second-Line Treatment of Metastatic Colorectal Cancer Using Fluoropyrimidine, Irinotecan, and Angiogenesis Inhibitor	MDV		Nationwide		2008	2019	Intervention effect		Neoplasms		malignat neoplasm of colon (ICD10 code: C18-20)	Others	age 20 years or higher and 90 years and below
Kawamura 2020	Effect of adjuvant chemotherapy on survival benefit in stage III colon cancer patients stratified by age: a Japanese real-world cohort study	DPC	Osaka Cancer Registry + DPC	Prefecture	Osaka	2010	2014	Intervention effect	Medical treatment status	Neoplasms		stage III colon cancer	Others	patients aged 18 years or older
Sakamoto 2018	[The influence of lifestyle-related disease on colon cancer by the decision tree analysis using DPC data]	DPC		Nationwide		2010	2012	Clinical epidemiology, course of diseases		Neoplasms		colon cancer	Others	age ≥20 years
Shin 2019	Effects of preoperative oral management by dentists on postoperative outcomes following esophagectomy: Multilevel propensity score matching and weighting analyses using the Japanese inpatient database	DPC		Nationwide		2016	2017	Intervention effect	Health economics	Neoplasms		esophageal cancer cases (C15.x)		
Tsukada 2018	The use of neoadjuvant therapy for resectable locally advanced thoracic esophageal squamous cell carcinoma in an analysis of 5016 patients from 305 designated cancer care hospitals in Japan	DPC	Combined with the hospital-based registry data	Nationwide		2012	2013	Medical treatment status		Neoplasms		patients diagnosed with resectable locally advanced thoracic esophageal squamous cell carcinoma		

Takeda 2019	Prophylactic sivelestat for esophagectomy and in-hospital mortality: a propensity score-matched analysis of claims database	MDV		Nationwide		2010	2016	Medical treatment status	Intervention effect	Neoplasms		esophageal cancer (ICD10 code C15)	Others	aged 20 years or older
Hamamoto 2018	Treatment selection for esophageal cancer: evaluation from a nationwide database	DPC	DPC + HBCRs	Nationwide		2013	2013	Medical treatment status	Health economics	Neoplasms		esophageal cancer		
Kunitomi 2021	Intergenerational comparison of 5-HT3RA in the prevention of chemotherapy-induced nausea and vomiting in gastric cancer patients receiving cisplatin-based chemotherapy: an observational study using a Japanese administrative claims database	MDV		Nationwide		2012	2017	Intervention effect		Neoplasms		gastric cancer (ICD-10 code C16)	Others	Exclude less than 20 years old
Shibao 2020	Clinical Outcomes of Laparoscopic Versus Laparotomic Distal Gastrectomy in Gastric Cancer Patients: A Multilevel Analysis Based on a Nationwide Administrative Database in Japan	DPC		Nationwide		2012	2013	Intervention effect	Health economics	Neoplasms		gastric cancer (ICD10: C16)		
Yasunaga 2013	Outcomes after laparoscopic or open distal gastrectomy for early-stage gastric cancer: a propensity-matched analysis	DPC		Nationwide		2010	2010	Intervention effect		Neoplasms		gastric cancer		
Tahara 2020	Re-challenge of Platinum-based Chemotherapy for Platinum-refractory Patients with Recurrent or Metastatic Head and Neck Cancer: Claims Data Analysis in Japan	MDV		Nationwide		2013	2016	Intervention effect		Neoplasms		head and neck cancer	Others	age ≥18 years
Suzuki 2016	Cerebral infarction after intraarterial and intravenous chemoradiotherapy for head and neck cancer: A retrospective analysis using a Japanese inpatient database	DPC		Nationwide		2010	2013	Intervention effect		Neoplasms		head and neck cancer receiving platinumbased chemotherapy and concurrent radiotherapy		
Suzuki 2018	Postoperative mechanical bowel obstruction after pharyngolaryngectomy for hypopharyngeal cancer: Retrospective analysis using a Japanese inpatient database	DPC		Nationwide		2007	2014	Intervention effect		Neoplasms		patients underwent pharyngolaryngectomy		
Ichimiya 2011	Profiling of the care processes for laryngeal cancer with the Japanese administrative database	DPC		Nationwide		2008	2008	Medical treatment status		Neoplasms		laryngeal cancer		
Tsutsumi 2019	Impact of oral voriconazole during chemotherapy for acute myeloid leukemia and myelodysplastic syndrome: a Japanese nationwide retrospective cohort study	DPC		Nationwide		2010	2015	Intervention effect		Neoplasms		acute myeloid leukemia and myelodysplastic syndrome	Others	age upon admission ≥18 years
Maeda 2014	Regional differences in performance of bone marrow transplantation, care-resource use and outcome for adult T-cell leukaemia in Japan	DPC		Nationwide		2010	2010	Socioeconomic comparison	Medical treatment status	Neoplasms		Adult T-cell leukemia		
Okinaga 2018	Short-Term Outcomes following Hepatectomy in Elderly Patients with Hepatocellular Carcinoma: An Analysis of 10,805 Septuagenarians and 2,381 Octo- and Nonagenarians in Japan	DPC		Nationwide		2011	2012	Intervention effect		Neoplasms		hepatocellular carcinoma (ICD-10 code: C220)	Others	aged ≥20 years
Soma 2010	[Treatment of liver cancer and DPC current situation in Japan]	DPC	Global health consulting, Japan	Nationwide		2008	2008	Medical treatment status	Quality of care	Neoplasms		liver cancer		
Sato 2017	Association between hospital volume and in-hospital mortality following radiofrequency ablation for hepatocellular carcinoma	DPC		Nationwide		2010	2012	Health policy evaluation and utilization		Neoplasms		hepatocellular carcinoma (C220)		
Sato 2019	In-hospital mortality associated with transcatheter arterial embolization for treatment of hepatocellular carcinoma in patients on hemodialysis for end stage renal disease: a matched-pair cohort study using a nationwide database	DPC		Nationwide		2010	2012	Intervention effect		Neoplasms		Hepatocellular carcinoma (ICD-10 code C220)		
Akada 2019	Database analysis of patients with hepatocellular carcinoma and treatment flow in early and advanced stages	MDV		Nationwide		2008	2017	Medical treatment status		Neoplasms		hepatocellular carcinoma (C220)		
Fukuda 2020	Differences in healthcare expenditure estimates according to statistical approach: A nationwide claims database study on patients with hepatocellular carcinoma	NDB		Nationwide		2010	2018	Research methodology	Health economics	Neoplasms		Hepatocellular carcinoma	Others	≥20 years of age
Sato 2012	Mortality and morbidity of hepatectomy, radiofrequency ablation, and embolization for hepatocellular carcinoma: a national survey of 54,145 patients	DPC		Nationwide		2007	2008	Clinical epidemiology, course of diseases	Intervention effect	Neoplasms		hepatocellular carcinoma (C220)		
Yasunaga 2012	Relationship between hospital volume and operative mortality for liver resection: Data from the Japanese Diagnosis Procedure Combination database	DPC		Nationwide		2007	2009	Health policy evaluation and utilization		Neoplasms		liver resection		
Shinkawa 2018	Mortality and morbidity after hepatic resection in patients undergoing hemodialysis: analysis of a national inpatient database in Japan	DPC		Nationwide		2010	2014	Clinical epidemiology, course of diseases		Neoplasms	Diseases of the genitourinary system	hepatic resection in patients undergoing hemodialysis	Others	patients aged ≥20 years
Goto 2020	Treatment Sequencing in Patients with Anaplastic Lymphoma Kinase-Positive Non-Small Cell Lung Cancer in Japan: A Real-World Observational Study	MDV		Nationwide		2010	2017	Medical treatment status	Intervention effect	Neoplasms		malignant neoplasm of bronchus and lung (ICD-10: C34)	Others	as least 18 yeas of age
Mori 2020	The role of heparin bridging in lung cancer surgery: a nationwide database analysis	DPC		Nationwide		2014	2018	Intervention effect		Neoplasms		lung cancer (ICD-10code: C34)		
Fujimoto 2019	Effect of combination of pre- and postoperative pulmonary rehabilitation on onset of postoperative pneumonia: a retrospective cohort study based on data from the diagnosis procedure combination database in Japan	MDV	DPC data collected by Medical Data Vision	Nationwide		2018	2018	Intervention effect		Neoplasms		patients admitted who received operative treatment for a new primary (ICD codes: C34)		
Urushiyama 2018	Oral fluorouracil vs vinorelbine plus cisplatin as adjuvant chemotherapy for stage II-IIIA non-small cell lung cancer: Propensity score-matched and instrumental variable analyses	DPC		Nationwide		2010	2015	Intervention effect		Neoplasms		patients with non-small cell lung cancer (ICD-10codes: C340, C341, C342, C343, C348)	Others	excluded patients aged ≤17 years
Urushiyama 2017	Adjuvant chemotherapy versus chemoradiotherapy for small cell lung cancer with lymph node metastasis: a retrospective observational study with use of a national database in Japan	DPC		Nationwide		2010	2015	Intervention effect		Neoplasms		small cell lung cancer	Others	excluded patients aged ≤17 years
Wang 2017	Systemic Treatment Patterns With Advanced or Recurrent Non-small Cell Lung Cancer in Japan: A Retrospective Hospital Administrative Database Study	MDV		Nationwide		2008	2015	Medical treatment status		Neoplasms		Non–small Cell Lung Cance	Others	age ≥18 years
Sato 2017	[Association between perioperative pulmonary rehabilitation and postoperative pneumonia in lung cancer resection patients : An analysis using Japanese administrative database]	DPC	QIP	Nationwide		2012	2015	Intervention effect		Neoplasms		lung cancer resection patients	Others	excluded patients aged <18 years
Kunisawa 2014	Survival analyses of postoperative lung cancer patients: an investigation using Japanese administrative data	DPC	QIP	Nationwide		2008	2013	Clinical epidemiology, course of diseases		Neoplasms		Non-small cell lung cancer patients (C34)		
Kondo 2012	Evaluation of the introduction of a diagnosis procedure combination system for patient outcome and hospitalisation charges for patients with hip fracture or lung cancer in Japan	DPC	JRCMC	Nationwide	92 Japanese Red Cross Medical Centres and community hospitals	2005	2008	Health policy evaluation and utilization		Neoplasms	Injury, poisoning and certain other consequences of external causes	hip fracture or lung cancer		
Otake 2011	Impact of hospital volume on chest tube duration, length of stay, and mortality after lobectomy	DPC		Nationwide		2007	2008	Health policy evaluation and utilization		Neoplasms		lung cancer		

Uda 2018	Preoperative short-term plus postoperative physical therapy versus postoperative physical therapy alone for patients undergoing lung cancer surgery: retrospective analysis of a nationwide inpatient database	DPC		Nationwide		2010	2015	Intervention effect		Neoplasms		lung cancer	Others	aged 18 years or older
Tsutsue 2021	Comparative effectiveness study of chemotherapy in follicular lymphoma patients in the rituximab era: a Japanese claims database study	MDV		Nationwide		2008	2017	Health economics	Medical treatment status	Neoplasms		ICD-10 diagnosis code for Follicular lymphoma (C82.x)		
Ohno 2020	Cost-effectiveness analysis of treatment regimens with obinutuzumab plus chemotherapy in Japan for untreated follicular lymphoma patients	Others	MDV, JMDC; localizing the model in US (Guzauskas et al. 2019)	Nationwide		2012	2018	Health economics		Neoplasms		follicular lymphoma	Others	at least 18-years-old of age
Tsutsue 2020	Nationwide claims database analysis of treatment patterns, costs and survival of Japanese patients with diffuse large B-cell lymphoma	MDV		Nationwide		2008	2017	Medical treatment status	Health economics	Neoplasms		diffuse large B-cell lymphoma		
Ikawa 2021	In-Hospital Complications After Surgery in Elderly Patients with Asymptomatic or Minor Symptom Meningioma: A Nationwide Registry Study	DPC		Nationwide		2010	2015	Clinical epidemiology, course of diseases	Intervention effect	Neoplasms		Asymptomatic or Minor Symptom Meningioma (ICD-10 code: D32)	Others	aged 18-90 years
Kikuchi 2019	[The Usage of Acetylsalicylic Acid for Lenalidomide Medication in Patients with Multiple Myeloma —Survey on Actual Condition of Clinical Data Using MDVAnalyzer—]	MDV		Nationwide		2016	2017	Medical treatment status		Neoplasms		multiple myeloma (ICD-10: C900)	Others	aged 20 years or older
Uno 2020	Current treatment patterns and medical costs for multiple myeloma in Japan: a cross-sectional analysis of a health insurance claims database	MDV		Nationwide		2008	2016	Medical treatment status	Health economics	Neoplasms		multiple myeloma (C90.0)		
Iida 2021	Medical database analysis of japanese multiple myeloma patients with planned stem cell transplantation (MEDALIST) - a focus on healthcare resource utilization and cost	MDV		Nationwide		2013	2016	Health economics		Neoplasms		multiple myeloma		
Guan 2020	Treatment Patterns in Newly Diagnosed Multiple Myeloma Patients in Japan Using A Large-scale Claims Database: Retrospective Cohort Study	MDV		Nationwide		2008	2015	Medical treatment status		Neoplasms		Multiple Myeloma (ICD-10 code: C900)	Others	aged 18 years or older
Ogura 2014	Nomogram predicting severe adverse events after musculoskeletal tumor surgery: analysis of a national administrative database	DPC		Nationwide		2007	2012	Prediction model	Intervention effect	Neoplasms		musculoskeletal tumor		
Ogura 2013	Incidence and risk factors for pulmonary embolism after primary musculoskeletal tumor surgery	DPC		Nationwide		2007	2010	Intervention effect		Neoplasms		who underwent musculoskeletal tumor surgery (C40-41, C47-49)		
Ogura 2013	Impact of hospital volume on postoperative complications and in-hospital mortality after musculoskeletal tumor surgery: analysis of a national administrative database	DPC		Nationwide		2007	2010	Health policy evaluation and utilization		Neoplasms		who underwent musculoskeletal tumor surgery(C40-41, C47-49, C795)		
Suzuki 2021	Treatment patterns in pancreatic cancer patients based on a hospital claims database in Japan	MDV		Nationwide		2010	2018	Medical treatment status		Neoplasms		Pancreatic cancer (ICD-10: C250–253 and C257–259)		
Umegaki 2018	The impact of low body mass index on postoperative outcomes in pancreatectomy patients: a retrospective analysis of Japanese administrative data	DPC	QIP	Nationwide		2010	2016	Clinical epidemiology, course of diseases		Neoplasms		pancreatic cancer	Others	excluded aged below 20 years
Tarasawa 2018	[Comparative validation of hospital death by cancer from the viewpoint of presence or absence of home medical care before hospitalization : Treatment contents and period to death in pancreatic cancer cases based on DPC data]	DPC		Nationwide		2014	2016	Medical treatment status	Patient health service utilization	Neoplasms		pancreatic cancer (ICD10: C25)	Older persons	age ≥65 years
Tsuda 2016	Complications and Postoperative Mortality Rate After Surgery for Pathological Femur Fracture Related to Bone Metastasis: Analysis of a Nationwide Database	DPC		Nationwide		2007	2012	Intervention effect		Neoplasms	Diseases of the musculoskeletal system and connective tissue	pathological femur fracture (ICD-10 code: M8445, C795)		
Tsuda 2015	[Predictors of early mortality in femur metastasis patients with pathological fracture : Analysis of DPC detabase]	DPC		Nationwide		2007	2012	Clinical epidemiology, course of diseases		Neoplasms	Diseases of the musculoskeletal system and connective tissue	Pathological fracture, femur and pelvis (ICD-10 code: M8445, C795)		
Sawada 2020	Predictive factors of the 30-day mortality after surgery for spinal metastasis: Analysis of a nationwide database	DPC		Nationwide		2010	2016	Prediction model	Clinical epidemiology, course of diseases	Neoplasms	Diseases of the musculoskeletal system and connective tissue	pathological spine fracture, bone metastasis		
Matsuda 2016	The Diagnosis Procedure Combination based situation analysis of surgical intervention for pituitary tumor in Japan	DPC		Nationwide		2011	2012	Health policy evaluation and utilization		Neoplasms		discharge cases of pituitary tumor		
Komatsu 2020	Real-world, retrospective study evaluating thromboembolic events, associated risk factors, and health-care resource utilization in Japanese patients with polycythemia vera	MDV		Nationwide		2008	2015	Clinical epidemiology, course of diseases	Health economics	Neoplasms		Polycythemia vera	Others	aged ≥20 years
Isogai 2017	Factors affecting in-hospital mortality and likelihood of undergoing surgical resection in patients with primary cardiac tumors	DPC		Nationwide		2010	2013	Clinical epidemiology, course of diseases		Neoplasms		primary cardiac tumors		
Mori 2019	The correlation between metastasis-free survival and overall survival in non-metastatic castration resistant prostate cancer patients from the Medical Data Vision claims database in Japan	MDV		Nationwide		2003	2018	Clinical epidemiology, course of diseases		Neoplasms		prostate cancer		
Satoh 2018	The Economic Burden of Metastatic Castration Resistant Prostate Cancer and Skeletal Related Events in Japanese University Hospitals	Others	Clinical Information Statistical Analysis (CISA) database: contains the electronic records of approximately 2.5 million patients	Others	13 university hospitals	2005	2016	Health economics		Neoplasms		metastatic castration resistant prostate cancer		
Goto 2017	Cost analysis of leuprorelin acetate in Japanese prostate cancer patients: comparison between 6-month and 3-month depot formulations	MDV	web-based survey to determine the intangible costs	Nationwide		2008	2016	Health economics		Neoplasms		prostate cancer (ICD-10 code: C61)		
Sugihara 2017	Accessibility to surgical robot technology and prostate-cancer patient behavior for prostatectomy	DPC		Nationwide		2012	2014	Patient health service utilization		Neoplasms		malignant neoplasm of the prostate (code C61)		
Kunisawa 2015	Realities in cost-effectiveness analyses: a study of castration-resistant prostate cancer patients using a medical claims database	JMDC		Nationwide		2005	2013	Medical treatment status	Health economics	Neoplasms		castration-resistant prostate cancer patients	Others	below 75 years old
Sugihara 2014	Robot-assisted versus other types of radical prostatectomy: population-based safety and cost comparison in Japan, 2012-2013	DPC		Nationwide		2012	2013	Intervention effect	Health economics	Neoplasms		the main diagnosis of malignant neoplasm of the prostate (ICD-10 code C61)		
Sugihara 2013	Is mechanical bowel preparation in laparoscopic radical prostatectomy beneficial? An analysis of a Japanese national database	DPC		Nationwide		2008	2010	Intervention effect		Neoplasms		prostate cancer		
Sugihara 2013	Comparisons of perioperative outcomes and costs between open and laparoscopic radical prostatectomy: a propensity-score matching analysis based on the Japanese Diagnosis Procedure Combination database	DPC		Nationwide		2007	2010	Intervention effect	Health economics	Neoplasms		prostatectomy, prostate cancer		
Sugihara 2013	Wide range and variation in minimally invasive surgery for renal malignancy in Japan: a population-based analysis	DPC	DPC Study Group	Nationwide		2007	2010	Medical treatment status	Socioeconomic comparison	Neoplasms		renal malignancy (C64)		
Takagi 2014	Cytoreductive nephrectomy for metastatic renal cell carcinoma: a population-based analysis of perioperative outcomes according to clinical stage	DPC		Nationwide		2007	2012	Intervention effect		Neoplasms		metastatic renal cell carcinoma (C64)		
Suzuki 2016	Factors Associated With Neck Hematoma After Thyroidectomy: A Retrospective Analysis Using a Japanese Inpatient Database	DPC		Nationwide		2010	2014	Intervention effect		Neoplasms	Others	patients undergoing thyroidectomy		

Sugihara 2014	Performance comparisons in major uro-oncological surgeries between the USA and Japan	DPC		Nationwide	USA and Japan	2009	2011	Medical treatment status	Socioeconomic comparison	Neoplasms		uro-oncological surgeries		
Yasunaga 2010	Impact of hospital volume on postoperative complications and in-hospital mortality after renal surgery: data from the Japanese Diagnosis Procedure Combination Database	DPC		Nationwide		2006	2007	Health policy evaluation and utilization		Neoplasms		nephrectomy or nephroureterectomy		
Kanemura 2020	Thymic epithelial tumor treatment in Japan: analysis of hospital cancer registry and insurance claims data, 2012-2014	DPC	HBCR+DPC	Nationwide		2012	2014	Medical treatment status		Neoplasms		Thymic epithelial tumors		
Konishi 2020	Comparison of outcomes after differentiated thyroid cancer surgery performed with and without energy devices: A population-based cohort study using a nationwide database in Japan	DPC		Nationwide		2010	2017	Intervention effect		Neoplasms		patients with thyroid cancer who underwent thyroidectomy		
Mizuno 2019	Recurrent laryngeal nerve paralysis after thyroid cancer surgery and intraoperative nerve monitoring	MDV		Nationwide		2014	2017	Intervention effect		Neoplasms		patients who underwent thyroid cancer surgery	Others	age ≥20 years
Mizuno 2020	Outcomes in patients diagnosed with tongue cancer before and after the age of 45 years	MDV		Nationwide		2008	2019	Clinical epidemiology, course of diseases		Neoplasms		tongue cancer (ICD10code: C020, C021, C022 or C029)	Others	patients aged ≥20 years
Sugihara 2014	Factors affecting choice between ureterostomy, ileal conduit and continent reservoir after radical cystectomy: Japanese series	DPC		Nationwide		2007	2012	Medical treatment status		Neoplasms		patients who underwent radical cystectomy for urothelial carcinoma		
Akada 2021	Real-world database analysis of the characteristics and treatment patterns of patients with endometrial cancer in Japan	MDV		Nationwide		2008	2019	Clinical epidemiology, course of diseases	Medical treatment status	Neoplasms		endometrial and uterine cancers (C541, C549)		
Muramatsu 2013	Description of Treatment of Uterus Cancer Based on the Japanese DPC Database	DPC		Nationwide		2010	2010	Medical treatment status		Neoplasms		uterus cancer		
3) Diseases of the blood and blood-forming organs and certain disorders involving the immune mechanism (D50-D89)														
Kamesaki 2020	Demographic characteristics, thromboembolism risk, and treatment patterns for patients with cold agglutinin disease in Japan	MDV		Nationwide		2008	2017	Clinical epidemiology, course of diseases	Medical treatment status	Diseases of the blood and blood-forming organs and certain disorders involving the immune mechanism		Cold agglutinin disease	Others	aged ≥18 years
Ohbe 2020	Underlying Disorders, Clinical Phenotypes, and Treatment Diversity among Patients with Disseminated Intravascular Coagulation	DPC		Nationwide		2010	2018	Clinical epidemiology, course of diseases		Diseases of the blood and blood-forming organs and certain disorders involving the immune mechanism	Pregnancy, childbirth and the puerperium	disseminated intravascular coagulation (ICD10codes: D64, O450, O460, O723, O081)	Others	excluded patients who were younger tha 18 years of age
Taniguchi 2020	Antithrombin use and mortality in patients with stage IV solid tumor-associated disseminated intravascular coagulation: a nationwide observational study in Japan	DPC		Nationwide		2010	2018	Intervention effect		Diseases of the blood and blood-forming organs and certain disorders involving the immune mechanism	Neoplasms	disseminated intravascular coagulation (admitted with the stage IV soild tumors)		
Yamakawa 2020	Time Trends of the Outcomes and Treatment Options for Disseminated Intravascular Coagulation: A Nationwide Observational Study in Japan	DPC		Nationwide		2010	2018	Clinical epidemiology, course of diseases		Diseases of the blood and blood-forming organs and certain disorders involving the immune mechanism	Pregnancy, childbirth and the puerperium	patients with disseminated intravascular coagulation (ICD-10 codes: D65, O450, O460, O723, O081)	Others	excluded patients who were younger than 18 years of age
Ichikawa 2019	[Recombinant human soluble thrombomodulin in neonatal DIC in Japan: analysis using a diagnosis procedure combination database]	DPC	DPCRI	Nationwide		2014	2016	Medical treatment status		Diseases of the blood and blood-forming organs and certain disorders involving the immune mechanism	Certain conditions originating in the perinatal period	Disseminated intravascular coagulation syndrome (D65, P60)	Children	0 years old
Murata 2017	Effects of longer use of recombinant human soluble thrombomodulin on outcomes of patients with disseminated intravascular coagulation based on a national administrative database	DPC		Nationwide		2010	2012	Intervention effect		Diseases of the blood and blood-forming organs and certain disorders involving the immune mechanism		disseminated intravascular coagulation		
Araki 2019	Incidence and In-Hospital Mortality of Neonatal Disseminated Intravascular Coagulation in Japan: An Observational Study of a Nationwide Hospital Claims Database	DPC		Nationwide		2014	2016	Clinical epidemiology, course of diseases		Diseases of the blood and blood-forming organs and certain disorders involving the immune mechanism	Certain conditions originating in the perinatal period	Neonatal Disseminated Intravascular Coagulation	Children	neonates
Ohbe 2019	Treatment with Antithrombin or Thrombomodulin and Mortality from Heatstroke-Induced Disseminated Intravascular Coagulation: A Nationwide Observational Study	DPC		Nationwide		2010	2017	Intervention effect		Diseases of the blood and blood-forming organs and certain disorders involving the immune mechanism	Injury, poisoning and certain other consequences of external causes	heatstroke-induced disseminated intravascular coagulation	Others	aged ≥19 years
Tagami 2015	Use of recombinant human soluble thrombomodulin in patients with sepsis-induced disseminated intravascular coagulation after intestinal perforation	DPC		Nationwide		2010	2013	Intervention effect		Diseases of the blood and blood-forming organs and certain disorders involving the immune mechanism	Diseases of the digestive system	sepsis-induced disseminated intravascular coagulation after intestinal perforation	Others	≥15 years of age
Tagami 2015	Supplemental dose of antithrombin use in disseminated intravascular coagulation patients after abdominal sepsis	DPC		Nationwide		2010	2013	Intervention effect		Diseases of the blood and blood-forming organs and certain disorders involving the immune mechanism	Diseases of the digestive system	disseminated intravascular coagulation patients after abdominal sepsis	Others	age ≥15 years
Tagami 2015	Recombinant human soluble thrombomodulin and mortality in severe pneumonia patients with sepsis-associated disseminated intravascular coagulation: an observational nationwide study	DPC		Nationwide		2010	2013	Intervention effect		Diseases of the blood and blood-forming organs and certain disorders involving the immune mechanism	Diseases of the respiratory system	pneumonia and sepsis-associated Disseminated intravascular coagulation	Others	aged ≥18 years
Tagami 2014	Antithrombin and mortality in severe pneumonia patients with sepsis-associated disseminated intravascular coagulation: an observational nationwide study	DPC		Nationwide		2010	2013	Intervention effect		Diseases of the blood and blood-forming organs and certain disorders involving the immune mechanism	Diseases of the respiratory system	patients with severe pneumonia with sepsis-induced Disseminated intravascular coagulation	Others	aged ≥18 years
Yamana 2016	Comparison of Procedure-Based and Diagnosis-Based Identifications of Severe Sepsis and Disseminated Intravascular Coagulation in Administrative Data	DPC	NHO, laboratory data as a reference	Others	three hospitals	2013	2014	Research methodology		Diseases of the blood and blood-forming organs and certain disorders involving the immune mechanism	Certain infectious and parasitic diseases	severe sepsis and disseminated intravascular coagulation	Others	aged ≥18 years
Okubo 2018	Recent trends in practice patterns and comparisons between immunoglobulin and corticosteroid in pediatric immune thrombocytopenia	DPC		Nationwide		2010	2015	Medical treatment status	Health economics	Diseases of the blood and blood-forming organs and certain disorders involving the immune mechanism		acute pediatric immune thrombocytopenia (ICD-10 code, D69.3)	Children	aged between 3 months and 18 years

Wong 2021	Management of primary immune thrombocytopenia in a real-world setting in Japan: eltrombopag versus corticosteroids	MDV		Nationwide		2010	2018	Intervention effect		Diseases of the blood and blood-forming organs and certain disorders involving the immune mechanism		immune thrombocytopenia	Others	aged ≥20 years
4) Endocrine, nutritional and metabolic diseases (E00-E90)														
Matsubayashi 2020	Prevalence, incidence, comorbidities, and treatment patterns among Japanese patients with acromegaly: a descriptive study using a nationwide claims database	NDB		Nationwide		2012	2018	Clinical epidemiology, course of diseases	Medical treatment status	Endocrine, nutritional and metabolic diseases		acromegary	Others	≥20 years of age
Iwasaku 2019	Impact of underlying chronic adrenal insufficiency on clinical course of hospitalized patients with adrenal crisis: A nationwide cohort study	Others	data from a claims database	Nationwide	145 acute phase hospitals across Japan	2003	2014	Clinical epidemiology, course of diseases		Endocrine, nutritional and metabolic diseases		Chronic adrenal insufficiency, adrenal crisis	Others	age ≥18 years
Iwasaku 2017	Clinical characteristics of adrenal crisis in adult population with and without predisposing chronic adrenal insufficiency: a retrospective cohort study	MDV		Nationwide		2003	2014	Clinical epidemiology, course of diseases		Endocrine, nutritional and metabolic diseases		Adrenal crisis	Others	age ≥18 years
Nakamura 2015	[Geographical distribution of primary care clinics for elderly ambulatory diabetic patients in Ibaraki Prefecture]	NHI/LSEHS	insurance claims database (latter-stage elderly healthcare system data)	Prefecture	Ibaraki	2010	2010	Health policy evaluation and utilization		Endocrine, nutritional and metabolic diseases		diabetes	Older persons	aged 75 years and older
Horii 2021	On-label use of sodium-glucose cotransporter 2 inhibitors might increase the risk of diabetic ketoacidosis in patients with type 1 diabetes	MDV		Nationwide		2018	2019	Intervention effect		Endocrine, nutritional and metabolic diseases		type 1 diabetes		
Ikesu 2021	Trends in Diabetes Care during the COVID-19 Outbreak in Japan: an Observational Study	MDV		Nationwide		2019	2020	Medical treatment status	COVID-19	Endocrine, nutritional and metabolic diseases		diabetes		
Kaneko 2021	Rates and risk factors for amputation in people with diabetes in Japan: a historical cohort study using a nationwide claims database	JMDC		Nationwide		2008	2016	Clinical epidemiology, course of diseases		Endocrine, nutritional and metabolic diseases		diabetes, amputation	Others	aged 18–72 years
Kaneko 2021	A Japanese Study Assessing Glycemic Control with Use of IDegAsp Co-formulation in Patients with Type 2 Diabetes in Clinical Practice: The JAGUAR Study	MDV		Nationwide		2018	2018	Intervention effect		Endocrine, nutritional and metabolic diseases		type 2 diabetes	Others	≥18 years of age
Kohsaka 2021	Healthcare resource utilization after initiation of sodium-glucose co-transporter-2 inhibitors versus dipeptidyl peptidase-4 inhibitors or other glucose-lowering drugs in Japanese patients with type 2 diabetes	MDV		Nationwide		2014	2018	Health economics		Endocrine, nutritional and metabolic diseases		type 2 diabetes	Others	aged 18 years or older
Komuro 2021	Lower heart failure and chronic kidney disease risks associated with sodium-glucose cotransporter-2 inhibitor use in Japanese type 2 diabetes patients without established cardiovascular and renal diseases	MDV		Nationwide		2014	2018	Intervention effect		Endocrine, nutritional and metabolic diseases	Others	diabetes, heat failure, chronic kidney disase	Others	aged ≥18 years
Iketani 2021	[Identification of Patients with Type 2 Diabetes Mellitus Requiring Concentrated Pharmaceutical Management in Health Insurance] Pharmacies:Quantitative Evaluation Using Administrative Claims Data	JMDC		Nationwide		2018	2019	Medical treatment status		Endocrine, nutritional and metabolic diseases		Type 2 Diabetes	Others	≥18 years of age
Harada 2020	Association of treatment-achieved HbA1c with incidence of coronary artery disease and severe eye disease in diabetes patients	JMDC		Nationwide		2008	2016	Clinical epidemiology, course of diseases	Intervention effect	Endocrine, nutritional and metabolic diseases	Others	coronary artery disease and severe eye disease in diabetes patients	Others	18-72 years old
Ihana-Sugiyama 2020	Comparison of effectiveness and drug cost between dipeptidyl peptidase-4 inhibitor and biguanide as the first-line anti-hyperglycaemic medication among Japanese working generation with type 2 diabetes	JMDC		Nationwide		2010	2016	Intervention effect	Health economics	Endocrine, nutritional and metabolic diseases		type 2 diabetes	Others	20-69 years old
Jiang 2020	Health Inequalities Among Elderly Type 2 Diabetes Mellitus Patients in Japan	NHI/LSEHS	Fukuoka Prefecture Wide-Area Association of Latter-Stage Elderly Healthcare	Prefecture	Fukuoka	2013	2016	Patient health service utilization	Socioeconomic comparison	Endocrine, nutritional and metabolic diseases		type 2 diabetes	Older persons	≥75 years of age
Kameda 2020	Use of oral antidiabetic drugs in Japanese working-age patients with type 2 diabetes mellitus: dosing pattern for metformin initiators	JMDC		Nationwide		2014	2017	Medical treatment status		Endocrine, nutritional and metabolic diseases		type 2 diabetes	Others	≥30 years of age
Kawaguchi 2020	Does disease management for diabetic nephropathy reduce medical expenditure? Evidence from a three-period difference-in-differences analysis	NHI/LSEHS	NHI	Municipality	Kure city	2010	2013	Health economics		Endocrine, nutritional and metabolic diseases		diabetic nephropathy		
Kume 2020	Recent Epidemiological Status of Ocular and Other Major Complications Related to Diabetes Mellitus in Japan	JMDC		Nationwide		2005	2014	Clinical epidemiology, course of diseases		Endocrine, nutritional and metabolic diseases		diabetes		
Kume 2020	Systemic and ocular diseases associated with the development of diabetic macular edema among Japanese patients with diabetes mellitus	JMDC		Nationwide		2005	2014	Clinical epidemiology, course of diseases		Endocrine, nutritional and metabolic diseases		diabetic macular edema		
Suzuki 2020	[Medical cost analysis for diabetes and complications in municipalities in Miyagi Prefecture]*	NHI/LSEHS	NHI	Municipality	Tomiya city, Miyagi	2016	2016	Clinical epidemiology, course of diseases	Health economics	Endocrine, nutritional and metabolic diseases		diabetes		
Horii 2019	Determination of factors affecting medication adherence in type 2 diabetes mellitus patients using a nationwide claim-based database in Japan	JMDC		Nationwide		2005	2013	Patient health service utilization		Endocrine, nutritional and metabolic diseases		type 2 diabetes	Others	18-74 years old
Ito 2019	Characteristics of Patients with Diabetes Initiating Sodium Glucose Co-transporter-2 Inhibitors (SGLT2i): Real-World Results from Three Administrative Databases in Japan	Others	MDV, JMDC, Kyowa Kikaku Ltd. Medi-Trend	Nationwide		2014	2017	Medical treatment status		Endocrine, nutritional and metabolic diseases		diabetes initiating SGLT2 inihibitors	Others	≥18 years of age
Ito 2019	Drug Utilization Patterns in Patients with Diabetes Initiating Sodium Glucose Co-Transporter-2 Inhibitors (SGLT2i) in Japan: A Multi-Database Study (2014-2017)	Others	MDV, JMDC, Kyowa Kikaku Ltd. Medi-Trend	Nationwide		2014	2017	Medical treatment status		Endocrine, nutritional and metabolic diseases		diabetes	Others	≥18 years of age
Ito 2019	Real-World Effectiveness of Sodium Glucose Co-Transporter-2 Inhibitors in Japanese Patients with Diabetes Mellitus	Others	MDV, JMDC	Nationwide		2014	2016	Intervention effect		Endocrine, nutritional and metabolic diseases		diabetes	Others	≥18 years of age
Kanai 2019	Characteristics and Early Hypoglycemic Medications of Patients at Risk of Progression to Type 2 Diabetes in Japan: A Retrospective Cohort Study of Health Checkup and Claims Data	JMDC		Nationwide		2005	2015	Clinical epidemiology, course of diseases	Medical treatment status	Endocrine, nutritional and metabolic diseases		type 2 diabetes	Others	≥40 years of age
Gouda 2018	Associations between eating habits and glycemic control and obesity in Japanese workers with type 2 diabetes mellitus	JMDC		Nationwide		2009	2015	Clinical epidemiology, course of diseases		Endocrine, nutritional and metabolic diseases		diabetes		
Ishii 2018	Comparative analysis between sodium-glucose Co-transporter 2 inhibitors and biguanides as add-on medications in patients with type 2 diabetes mellitus receiving dipeptidyl peptidase 4 inhibitors-results from analysis of real-world clinical database	MDV		Nationwide		2013	2017	Intervention effect		Endocrine, nutritional and metabolic diseases		type 2 diabetes		
Kubota 2018	Penetration of new antidiabetic medications in East Asian countries and the United States: A cross-national comparative study	JMDC	Taiwan, Hong Kong, Japan, US	Nationwide		2006	2014	Medical treatment status	Socioeconomic comparison	Endocrine, nutritional and metabolic diseases		type 2 diabetes		

Ebata-Kogure 2017	Clinical and economic burdens experienced by patients with painful diabetic peripheral neuropathy: An observational study using a Japanese claims database	MDV		Nationwide		2008	2015	Clinical epidemiology, course of diseases	Health economics	Endocrine, nutritional and metabolic diseases	diabetic peripheral neuropathy	Others	≥18 years of age
Chang 2016	Epidemiology of lactic acidosis in type 2 diabetes patients with metformin in Japan	MDV		Nationwide		2010	2014	Intervention effect		Endocrine, nutritional and metabolic diseases	type 2 diabetes	Others	≥18 years of age
Fukuda 2016	The Effects of Diagnostic Definitions in Claims Data on Healthcare Cost Estimates: Evidence from a Large-Scale Panel Data Analysis of Diabetes Care in Japan	JMDC		Nationwide		2005	2013	Health economics	Medical treatment status	Endocrine, nutritional and metabolic diseases	type 2 diabetes	Others	≥20 years of age
Kurtyka 2016	Adherence to dipeptidyl peptidase-4 inhibitor therapy among type 2 diabetes patients with employer-sponsored health insurance in Japan	JMDC		Nationwide		2010	2013	Patient health service utilization		Endocrine, nutritional and metabolic diseases	type 2 diabetes	Others	18-65 years old
Heianza 2014	Impact on short-term glycaemic control of initiating diabetes care versus leaving diabetes untreated among individuals with newly screening-detected diabetes in Japan	JMDC		Nationwide		2009	2010	Medical treatment status	Intervention effect	Endocrine, nutritional and metabolic diseases	diabetes		
Hidaka 2014	[Prescription and Quality of Diabetes Care Provided by Specialists and General Physicians in Japan —An Analysis of Insurance Claim and Health Checkup Data—]	Others	SANYO Electric Group Health Insurance	Others	SANYO Electric Group Health Insurance	2012	2012	Medical treatment status	Health policy evaluation and utilization	Endocrine, nutritional and metabolic diseases	diabetes	Others	0-74 years old (beneficiaries)
Suzuki 2014	[Examination of prescription status of new antidiabetic drugs using health insurance claims data]*	JMDC		Nationwide		2005	2012	Medical treatment status		Endocrine, nutritional and metabolic diseases	diabetes	Others	20-75 years old
Kimura 2012	Quantitative Evaluation of Compliance with Recommendation for Sulfonylurea Dose Co-Administered with DPP-4 Inhibitors in Japan	Others	Nihon-Chouzai pharmacy claims database	Nationwide	Nihon-Chouzai pharmacy claims database	2008	2010	Medical treatment status	Health policy evaluation and utilization	Endocrine, nutritional and metabolic diseases	diabetes		
Kubo 2011	Prevalence of type 2 diabetes among acute inpatients and its impact on length of hospital stay in Japan	DPC		Nationwide		2008	2008	Clinical epidemiology, course of diseases		Endocrine, nutritional and metabolic diseases	type 2 diabetes		
Yanase 2011	[Utility Study of a Basic Database Collected from Patients with Diabetes Mellitus in 15 Hospitals by Retrospective Analysis]	MDV	Patient Survey 2008 (MHLW)	Nationwide	15 medical facilities	2006	2010	Research methodology		Endocrine, nutritional and metabolic diseases	diabetes		
Kitazato 2010	[A Method of Investigate Medical Expenditure in Japan in Relationship of Stage Progression of Type 2 Diabetes Mellitus Complications and Macroangiopathy]	JMDC		Nationwide	10 health insurance unions	2003	2007	Research methodology		Endocrine, nutritional and metabolic diseases	type 2 diabetes	Others	40-74 years old
Nagai 2021	A Claims Database Analysis of Dose-Dependency of Metformin and Incidence of Lactic Acidosis in Japanese Patients with Type 2 Diabetes	MDV		Nationwide		2008	2018	Intervention effect		Endocrine, nutritional and metabolic diseases	Type 2 diabetes	Others	age ≥18 years
Nagai 2021	Effects of Increasing Metformin Dose vs Adding/Switching to Dipeptidyl Peptidase-4 Inhibitors on Glycemic Control in Patients with Type 2 Diabetes	MDV		Nationwide		2008	2018	Intervention effect		Endocrine, nutritional and metabolic diseases	Type 2 diabetes	Others	age ≥18 years
Okada 2021	Association between nutritional guidance or ophthalmological examination and discontinuation of physician visits in patients with newly diagnosed diabetes: A retrospective cohort study using a nationwide database	JMDC		Nationwide		2005	2018	Patient health service utilization	Quality of care	Endocrine, nutritional and metabolic diseases	diabetes	Others	age ≥20 years
Okada 2021	Potassium Concentration in Initial Fluid Therapy and In-Hospital Mortality of Patients with Diabetic Ketoacidosis	DPC		Nationwide		2010	2018	Intervention effect		Endocrine, nutritional and metabolic diseases	patients with diabetic ketoacidosis	Others	age ≥20 years
Okui 2021	Performance evaluation of case definitions of type 1 diabetes for health insurance claims data in Japan	Others	Electronic healthcare records data and health insurance claims data in a hospital	Others	a University Hospital	2009	2019	Research methodology		Endocrine, nutritional and metabolic diseases	type 1 diabetes		
Nishioka 2020	Non-financial social determinants of diabetes among public assistance recipients in Japan: A cohort study	Others	medical assistance claim data, municipal public assistance databases	Municipality	two suburban municipalities, Osaka and Tokyo	2016	2016	Clinical epidemiology, course of diseases	Socioeconomic comparison	Endocrine, nutritional and metabolic diseases	diabetes		
Nishioka 2020	Incidence and seasonality of type 1 diabetes: a population-based 3-year cohort study using the National Database in Japan	NDB		Nationwide		2013	2018	Clinical epidemiology, course of diseases		Endocrine, nutritional and metabolic diseases	Type 1 diabetes		age groups
Oh 2020	Comparison of persistence and adherence between DPP-4 inhibitor administration frequencies in patients with type 2 diabetes mellitus in Japan: a claims-based cohort study	MDV		Nationwide		2015	2018	Medical treatment status	Patient health service utilization	Endocrine, nutritional and metabolic diseases	type 2 diabetes	Others	age ≥18 years
Yaguchi 2020	Skipping breakfast, late-night eating and current smoking are associated with medication adherence in Japanese patients with diabetes	JMDC		Nationwide		2008	2014	Patient health service utilization		Endocrine, nutritional and metabolic diseases	diabetes	Others	aged 18-74 years
Morita 2019	Treatment patterns of drug-naive patients with type 2 diabetes mellitus: a retrospective cohort study using a Japanese hospital database	MDV		Nationwide		2012	2016	Medical treatment status		Endocrine, nutritional and metabolic diseases	type 2 diabetes	Others	aged ≥18 years
Nishi 2019	Association between income levels and irregular physician visits after a health checkup, and its consequent effect on glycemic control among employees: A retrospective propensity score-matched cohort study	JHIA	Fukuoka branch of the Japanese Health Insurance Association	Prefecture	Fukuoka	2011	2013	Patient health service utilization	Socioeconomic comparison	Endocrine, nutritional and metabolic diseases	diabetes	Others	aged 40-64 years
Nishimura 2019	Treatment patterns, persistence and adherence rates in patients with type 2 diabetes mellitus in Japan: a claims-based cohort study	Others	JMDC, MDV	Nationwide		2011	2015	Medical treatment status	Patient health service utilization	Endocrine, nutritional and metabolic diseases	type 2 diabetes	Others	aged ≥18 years
Nishimura 2019	Comparison of persistence and adherence between fixed-dose combinations and two-pill combinations in Japanese patients with type 2 diabetes	Others	JMDC, MDV	Nationwide		2011	2015	Medical treatment status	Patient health service utilization	Endocrine, nutritional and metabolic diseases	type 2 diabetes	Others	aged ≥18 years
Sugiyama 2019	Variation in process quality measures of diabetes care by region and institution in Japan during 2015-2016: An observational study of nationwide claims data	NDB		Nationwide		2015	2016	Quality of care	Socioeconomic comparison	Endocrine, nutritional and metabolic diseases	diabetes	Others	aged ≥20 years
Suzuki 2019	Analysis on Real World Data (RWD) using a machine learning model-exploration of the factors affecting the utility of oral hypoglycemic agents	MDV		Nationwide		2008	2017	Clinical epidemiology, course of diseases	Research methodology	Endocrine, nutritional and metabolic diseases	diabetes	Others	aged ≥18 years
Tanaka 2019	Changes in the quality of diabetes care in Japan between 2007 and 2015: A repeated cross-sectional study using claims data	JMDC		Nationwide		2006	2016	Quality of care		Endocrine, nutritional and metabolic diseases	diabetes	Others	aged 20-69 years

Ukai 2019	Effectiveness of monthly and bimonthly follow-up of patients with well-controlled type 2 diabetes: a propensity score matched cohort study	NHI/LSEHS	NHI	Municipality	Tsu city	2011	2014	Intervention effect	Medical treatment status	Endocrine, nutritional and metabolic diseases		type 2 diabetes		
Zhou 2019	Identification of subgroups of patients with type 2 diabetes with differences in renal function preservation, comparing patients receiving sodium-glucose co-transporter-2 inhibitors with those receiving dipeptidyl peptidase-4 inhibitors, using a supervised machine-learning algorithm (PROFILE study): A retrospective analysis of a Japanese commercial medical database	MDV		Nationwide		2014	2016	Intervention effect	Medical treatment status	Endocrine, nutritional and metabolic diseases		type 2 diabetes	Others	aged ≥18 years
Nishimura 2018	Efficacy of metformin add-on therapy to dipeptidyl peptidase-4 inhibitors in Japanese patients with type 2 diabetes mellitus in real-world clinical practice settings: A retrospective analysis of a health insurance administrative claims database	MDV		Nationwide		2010	2015	Intervention effect		Endocrine, nutritional and metabolic diseases		type 2 diabetes	Others	aged ≥18 years
Nishimura 2018	Effectiveness of a dipeptidyl peptidase-4 inhibitor as add-on therapy to metformin and of dose-escalation of metformin in Japanese patients with type 2 diabetes mellitus in real-world clinical practice settings a retrospective analysis of a medical information database	MDV		Nationwide		2010	2015	Intervention effect		Endocrine, nutritional and metabolic diseases		type 2 diabetes	Others	aged ≥18 years
Suzuki 2018	Characteristics, Treatment Patterns, and Economic Outcomes of Patients Initiating Injectable Medications for Management of Type 2 Diabetes Mellitus in Japan: Results from a Retrospective Claims Database Analysis	JMDC		Nationwide		2011	2015	Medical treatment status	Health economics	Endocrine, nutritional and metabolic diseases		Type 2 diabetes	Others	aged ≥18 years
Takeuchi 2018	Population-based incidence of diabetic ketoacidosis in type 2 diabetes: medical claims data analysis in Japan	JMDC		Nationwide		2005	2013	Clinical epidemiology, course of diseases		Endocrine, nutritional and metabolic diseases		Diabetes, Diabetic ketoacidosis	Others	aged 20–74 years
Tamura 2018	A study on the prescription status of metformin and DPP-4 inhibitors in patients with type 2 diabetes mellitus in Japan: An analysis of time trends by use of a health insurance administrative claims database	JMDC		Nationwide		2011	2016	Medical treatment status		Endocrine, nutritional and metabolic diseases		Type 2 diabetes		
Yamashita 2018	[The Differences between the Two Island Towns in Medical Expenses of the National Health Insurance Concerning about Diabetes]	NHI/LSEHS	NHI	Municipality	Okinoshima-cho and Ama-cho in the Oki islands	2016	2016	Health economics		Endocrine, nutritional and metabolic diseases		Diabetes		
Ono 2017	Impact of clinic follow-up visits on body weight control in people with prediabetes or diabetes mellitus: Japanese nonelderly cohort study	JMDC		Nationwide		2013	2014	Medical treatment status	Intervention effect	Endocrine, nutritional and metabolic diseases		Diabetes	Others	aged 40–74 years
Tamura 2017	A study on the prescription status of metformin and DPP-4 inhibitors in patients with type 2 diabetes mellitus in Japan: An analysis of data from health insurance administrative claims	JMDC		Nationwide		2016	2016	Medical treatment status		Endocrine, nutritional and metabolic diseases		Type 2 diabetes		
Tanabe 2017	Prescription of oral hypoglycemic agents for patients with type 2 diabetes mellitus: A retrospective cohort study using a Japanese hospital database	MDV		Nationwide		2008	2013	Medical treatment status		Endocrine, nutritional and metabolic diseases		Type 2 diabetes	Others	aged 40–70 years
Tanaka 2016	Process quality of diabetes care under favorable access to healthcare: a 2-year longitudinal study using claims data in Japan	JMDC		Nationwide		2010	2012	Quality of care		Endocrine, nutritional and metabolic diseases		diabetes	Others	aged 20–69 years
Kishi 2013	[Hypoglycemia due to the Combination of Sitagliptin and a SU Drug, and Changes in the Daily Dose of the SU Drug Based on the New Safety Measures]	JMDC		Nationwide		2009	2010	Medical treatment status	Health policy evaluation and utilization	Endocrine, nutritional and metabolic diseases		diabetes		
Tomio 2010	Quality of care for diabetes patients using National Health Insurance claims data in Japan	NHI/LSEHS	NHI	Municipality	two separate towns in Kumamoto Prefecture	2006	2007	Quality of care		Endocrine, nutritional and metabolic diseases		diabetes		
Kosiborod 2018	Cardiovascular Events Associated With SGLT-2 Inhibitors Versus Other Glucose-Lowering Drugs: The CVD-REAL 2 Study	MDV		Nationwide	6 countries	2013	2017	Intervention effect		Endocrine, nutritional and metabolic diseases		type 2 diabetes (New users of SGLT-2i and oGLDs)	Others	aged ≥18 years
Kohro 2013	Trends in antidiabetic prescription patterns in Japan from 2005 to 2011	JMDC		Nationwide		2005	2011	Medical treatment status		Endocrine, nutritional and metabolic diseases		diabetes mellitus	Others	aged ≥20 years
Yabe 2015	Use of the Japanese health insurance claims database to assess the risk of acute pancreatitis in patients with diabetes: comparison of DPP-4 inhibitors with other oral antidiabetic drugs	JMDC		Nationwide		2009	2013	Intervention effect		Endocrine, nutritional and metabolic diseases	Diseases of the digestive system	diabetes, acute pancreatitis	Others	aged 30-74 years
Urushihara 2012	Increased Risk of Acute Pancreatitis in Patients with Type 2 Diabetes: An Observational Study Using a Japanese Hospital Database	MDV		Nationwide		2003	2010	Clinical epidemiology, course of diseases		Endocrine, nutritional and metabolic diseases	Diseases of the digestive system	diabetes, acute pancreatitis	Others	aged 18 years or more
Kaku 2019	Cost-effectiveness Analysis of Empagliflozin in Japan Based on Results From the Asian subpopulation in the EMPA-REG OUTCOME Trial	MDV		Nationwide		2016	2017	Health economics		Endocrine, nutritional and metabolic diseases	Diseases of the circulatory system	type 2 diabetes mellitus, cardiovascular and renal event		
Fujihara 2021	Accuracy of Japanese claims data in identifying diabetes-related complications	DPC	electronic medical chart reviews as the gold standard	Others	Niigata University Medical & Dental Hospital	2018	2018	Research methodology		Endocrine, nutritional and metabolic diseases	Diseases of the circulatory system	diabetesrelated complications		
Kohsaka 2020	Incidence of adverse cardiovascular events in type 2 diabetes mellitus patients after initiation of glucose-lowering agents: A population-based community study from the Shizuoka Kokuho database	NHI/LSEHS	Shizuoka Kokuho database	Prefecture	Shizuoka	2013	2018	Intervention effect		Endocrine, nutritional and metabolic diseases	Diseases of the circulatory system	type 2 diabetes, adverse cardiovascular events	Others	≥40 years of age
Komamine 2019	Cardiovascular risks associated with dipeptidyl peptidase-4 inhibitors monotherapy compared with other antidiabetes drugs in the Japanese population: A nationwide cohort study	NDB		Nationwide		2010	2014	Intervention effect		Endocrine, nutritional and metabolic diseases	Diseases of the circulatory system	diabetes, cardiovascular disease		age categories
Seino 2021	Cardiovascular and renal effectiveness of empagliflozin in routine care in East Asia: Results from the EMPRISE East Asia study	MDV		Nationwide	(Japan, South Korea and Taiwan)	2014	2018	Intervention effect		Endocrine, nutritional and metabolic diseases	Diseases of the circulatory system	Diabetes, cardiovascular, renal	Others	aged 18 years or older
Nishioka 2020	Absolute risk of acute coronary syndrome after severe hypoglycemia: A population-based 2-year cohort study using the National Database in Japan	NDB		Nationwide		2014	2016	Clinical epidemiology, course of diseases		Endocrine, nutritional and metabolic diseases	Diseases of the circulatory system	Diabetes, acute coronary syndrome	Others	aged ≥35 years

Ono 2020	Validity of Claims Diagnosis Codes for Cardiovascular Diseases in Diabetes Patients in Japanese Administrative Database	Others	HCEI, patient-level electronic medical record and claims data	Nationwide	(approximately 200 hospitals)	2006	2018	Research methodology		Endocrine, nutritional and metabolic diseases	Diseases of the circulatory system	Diabetes, Cardiovascular Diseases		
Yamada-Harada 2019	Relationship Between Number of Multiple Risk Factors and Coronary Artery Disease Risk With and Without Diabetes Mellitus	JMDC		Nationwide		2008	2016	Clinical epidemiology, course of diseases		Endocrine, nutritional and metabolic diseases	Diseases of the circulatory system	Coronary artery disease risk with and without diabetes mellitus	Others	aged 18 to 72 years
Yamada 2016	Postoperative outcomes of major lower extremity amputations in patients with diabetes and peripheral artery disease: analysis using the Diagnosis Procedure Combination database in Japan	DPC		Nationwide		2007	2012	Intervention effect		Endocrine, nutritional and metabolic diseases	Diseases of the circulatory system	diabetes, peripheral artery disease		
Goto 2016	[Association between severe hypoglycemia and the onset of cardiovascular disease in patients with type 2 diabetes: A study using multiple definitions of severe hypoglycemia]*	JMDC		Nationwide		2005	2014	Clinical epidemiology, course of diseases		Endocrine, nutritional and metabolic diseases	Diseases of the circulatory system	Type 2 diabetes, hypoglycemia, cardiovascular disease	Others	aged 18–74 years
Nishi 2014	Risk of hospitalization for diabetic macrovascular complications and in-hospital mortality with irregular physician visits using propensity score matching	NHI/LSEHS	Fukuoka National Health Insurance Organization	Prefecture	Fukuoka	2009	2010	Intervention effect	Patient health service utilization	Endocrine, nutritional and metabolic diseases	Diseases of the circulatory system	diabetic macrovascular complications		
Wake 2019	Adherence and persistence to hyperlipidemia medications in patients with atherosclerotic cardiovascular disease and those with diabetes mellitus based on administrative claims data in Japan	Others	JMDC and MDV	Nationwide		2014	2015	Medical treatment status	Patient health service utilization	Endocrine, nutritional and metabolic diseases	Diseases of the circulatory system	hyperlipidemia patients with diabetes mellitus or prior atherosclerotic cardiovascular diseases	Others	age >18 years
Nagar 2018	Treatment Patterns, Statin Intolerance, and Subsequent Cardiovascular Events Among Japanese Patients With High Cardiovascular Risk Initiating Statin Therapy	JMDC		Nationwide		2006	2014	Medical treatment status	Intervention effect	Endocrine, nutritional and metabolic diseases	Diseases of the circulatory system	atherosclerotic cardiovascular disease, diabetes mellitus	Others	at least 18 years of age
Ide 2010	Relationships between diabetes and medical and dental care costs: findings from a worksite cohort study in Japan	Others	a mutual aid association (periodic health examinations and biennial oral examinations)	Prefecture	a prefecture in southwestern Japan	2000	2005	Health economics		Endocrine, nutritional and metabolic diseases	Diseases of the digestive system	diabetes, medial and dental care costs	Others	40-54 years old
Shin 2021	Effects of periodontal management for patients with type 2 diabetes on healthcare expenditure, hospitalization and worsening of diabetes: an observational study using medical, dental and pharmacy claims data in Japan	NHI/LSEHS	National Health Insurance (NHI) and the Medical Care System for the Elderly	Prefecture	a prefecture	2015	2016	Intervention effect	Health economics	Endocrine, nutritional and metabolic diseases	Diseases of the digestive system	Diabetes, periodontal	Others	aged ≥35 years
Suzuki 2021	Evaluation of Public Health Expenditure by Number of Teeth among Outpatients with Diabetes Mellitus	NDB		Nationwide		2015	2016	Clinical epidemiology, course of diseases	Health economics	Endocrine, nutritional and metabolic diseases	Diseases of the digestive system	Diabetes, periodontitis	Others	aged 50-74 years
Suzuki 2020	Evaluation of tooth loss among patients with diabetes mellitus using the National Database of Health Insurance Claims and Specific Health Checkups of Japan	NDB		Nationwide		2015	2016	Clinical epidemiology, course of diseases		Endocrine, nutritional and metabolic diseases	Diseases of the digestive system	diabetes, tooth loss	Others	aged 50–74 years
Suzuki 2019	Relationship between Blood HbA1c Level and Decayed Teeth in Patients with Type 2 Diabetes: A Cross-sectional Study	Others	MinaCare (results of dental examinations)	Nationwide		2013	2013	Clinical epidemiology, course of diseases		Endocrine, nutritional and metabolic diseases	Diseases of the digestive system	diabetes, decayed teeth	Others	aged 40-59 years
Saito 2017	Association between dental visits for periodontal treatment and type 2 diabetes mellitus in an elderly Japanese cohort	NHI/LSEHS	late- stage medical care system for the older population	Prefecture	Mie	2014	2014	Medical treatment status	Clinical epidemiology, course of diseases	Endocrine, nutritional and metabolic diseases	Diseases of the digestive system	periodontal treatment, type 2 diabetes mellitus	Older persons	aged 75 or 80 years
Osawa 2021	Severity of hypertension as a predictor of initiation of dialysis among study participants with and without diabetes mellitus	JMDC		Nationwide		2008	2016	Clinical epidemiology, course of diseases		Endocrine, nutritional and metabolic diseases	Diseases of the genitourinary system	Diabetes, dialysis	Others	aged 19-72 years
Osawa 2019	Higher pulse pressure predicts initiation of dialysis in Japanese patients with diabetes	JMDC		Nationwide		2008	2013	Clinical epidemiology, course of diseases		Endocrine, nutritional and metabolic diseases	Diseases of the genitourinary system	diabetes, Dialysis	Others	aged 19-72 years
Ebihara 2019	[A fact-finding survey of lifestyle-related diseases such as diabetes and the introduction of new dialysis in Miyazaki Prefecture]*	NHI/LSEHS	National Health Insurance and Later-Stage Elderly Healthcare System	Prefecture	Miyazaki	2015	2016	Medical treatment status		Endocrine, nutritional and metabolic diseases	Diseases of the genitourinary system	Diabetes, dialysis		
Nogami 2017	Risk of disseminated intravascular coagulation in patients with type 2 diabetes mellitus: retrospective cohort study	MDV		Nationwide		2010	2014	Clinical epidemiology, course of diseases		Endocrine, nutritional and metabolic diseases		Diabetes, disseminated intravascular coagulation	Others	aged 18–79 years
Ooba 2017	Lipid-lowering drugs and risk of new-onset diabetes: a cohort study using Japanese healthcare data linked to clinical data for health screening	JMDC		Nationwide		2005	2011	Intervention effect		Endocrine, nutritional and metabolic diseases		Diabetes, lipid-lowering drugs	Others	aged 20 to 74 years
Kawamura 2018	Influence of comorbidities on the implementation of the fundus examination in patients with newly diagnosed type 2 diabetes	JMDC		Nationwide		2005	2013	Patient health service utilization		Endocrine, nutritional and metabolic diseases	Diseases of the eye and adnexa	type 2 diabetes, diabetic eye examination	Others	>20 years of age
Kawasaki 2018	Lipid-lowering medication is associated with decreased risk of diabetic retinopathy and the need for treatment in patients with type 2 diabetes: A real-world observational analysis of a health claims database	JMDC		Nationwide		2005	2017	Intervention effect		Endocrine, nutritional and metabolic diseases	Diseases of the eye and adnexa	type 2 diabetes, diabetic retinopathy	Others	≥18 years of age
Yamamoto 2019	Pulse Pressure is a Stronger Predictor Than Systolic Blood Pressure for Severe Eye Diseases in Diabetes Mellitus	JMDC		Nationwide		2008	2015	Clinical epidemiology, course of diseases		Endocrine, nutritional and metabolic diseases	Diseases of the eye and adnexa	diabetic retinopathy	Others	aged 19-72 years
Roughead 2015	Variation in Association Between Thiazolidinediones and Heart Failure Across Ethnic Groups: Retrospective analysis of Large Healthcare Claims Databases in Six Countries	JMDC		Nationwide	Australia, Hong Kong, Japan, Korea and Taiwan	2005	2010	Intervention effect	Socioeconomic comparison	Endocrine, nutritional and metabolic diseases	Diseases of the circulatory system	diabetes, heart failure, edema		
Sekimoto 2015	Supplier-Induced Demand for Chronic Disease Care in Japan: Multilevel Analysis of the Association between Physician Density and Physician-Patient Encounter Frequency	JHIA	patient surveys (MHLW),	Nationwide		2013	2013	Health policy evaluation and utilization	Health economics	Endocrine, nutritional and metabolic diseases	Diseases of the circulatory system	Chronic Disease Care (hypertension, diabetes)	Others	at least 20 years old
Mitsutake 2020	Association of pharmacological treatments for hypertension, diabetes, and dyslipidemia with health checkup participation and identification of disease control factors among older adults in Tokyo, Japan	NHI/LSEHS	Tokyo Extended Association of Medical Care for Latter-Stage Older People	Prefecture	Tokyo	2013	2014	Medical treatment status	Patient health service utilization	Endocrine, nutritional and metabolic diseases	Diseases of the circulatory system	diabetes, hypertension, dyslipidemia	Older persons	≥75 years of age

Hashiguchi 2019	Occupational Health Services Improve Effective Coverage for Hypertension and Diabetes Mellitus at Japanese Companies	Others	general medical exam data, human resources information, and medical claims data (at a general electrical manufacturer)	Others	a general electorical manufacturer	2011	2012	Health policy evaluation and utilization		Endocrine, nutritional and metabolic diseases	Diseases of the circulatory system	diabetes, hypertension	Others	40-59 years old
Hara 2018	Association measures of claims-based algorithms for common chronic conditions were assessed using regularly collected data in Japan	JMDC		Nationwide		2013	2015	Prediction model	Clinical epidemiology, course of diseases	Endocrine, nutritional and metabolic diseases	Diseases of the circulatory system	diabetes, hypertension, dyslipidemia		
Fujita 2015	Validity assessment of self-reported medication use by comparing to pharmacy insurance claims	NHI/LSEHS	NHI (self-reported questionnaire required in the health check-up)	Municipality	Chiba City	2012	2013	Research methodology		Endocrine, nutritional and metabolic diseases	Diseases of the circulatory system	diabetes, dyslipidemia, hypertension	Others	40-74 years old
Katada 2012	[Medication Adhesion to Calcium Channel Blocker and HMG－CoA Reductase Inhibitor in Patients with Diabetes and Beneficial Effects of a Single Pill Amlodipine/Atorvastatin]	Others	pharmacy claims data	Nationwide	pharmacy claims data (100 dispensaries)	2011	2011	Patient health service utilization		Endocrine, nutritional and metabolic diseases	Diseases of the circulatory system	diabetes, dyslipidemia, hypertension		
Katada 2012	[Adherence and Persistence of a Single Pill Amlodipine/Atorvastatin vs. Two Pill Regimen Based on Japanese Health Insurance Claims]	Others	pharmacy claims data	Nationwide	pharmacy claims data (100 dispensaries)	2011	2011	Patient health service utilization		Endocrine, nutritional and metabolic diseases	Diseases of the circulatory system	dyslipidemia, hypertension		
Yamana 2020	Association between mandatory health examination attendance and diabetes treatment initiation among employees being treated for hypertension	JMDC		Nationwide		2012	2016	Patient health service utilization		Endocrine, nutritional and metabolic diseases	Diseases of the circulatory system	diabetes, hypertension	Others	aged 40-59 years
Horii 2020	Real-world risk of hypoglycemia-related hospitalization in Japanese patients with type 2 diabetes using SGLT2 inhibitors: a nationwide cohort study	MDV		Nationwide		2014	2019	Clinical epidemiology, course of diseases	Intervention effect	Endocrine, nutritional and metabolic diseases		type 2 diabetes, hypoglycemia	Others	≥18 years of age
Ikeda 2019	Retrospective analysis of medical costs and resource utilization for severe hypoglycemic events in patients with type 2 diabetes in Japan	MDV		Nationwide		2008	2014	Health economics		Endocrine, nutritional and metabolic diseases		diabetes, hypoglycemic events		
Ikeda 2018	Incidence rate and patient characteristics of severe hypoglycemia in treated type 2 diabetes mellitus patients in Japan: Retrospective Diagnosis Procedure Combination database analysis	MDV		Nationwide		2008	2015	Clinical epidemiology, course of diseases		Endocrine, nutritional and metabolic diseases		type 2 diabetes, hypoglycemia	Others	>20 years of age
Bennett 2016	Association between therapy with dipeptidyl peptidase-4 (DPP-4) inhibitors and risk of ileus: a cohort study	MDV		Nationwide		2010	2014	Intervention effect		Endocrine, nutritional and metabolic diseases	Diseases of the digestive system	type 2 diabetes, ileus	Others	≥40 years of age
Nishioka 2021	Association between influenza and the incidence rate of new-onset type 1 diabetes in Japan	NDB		Nationwide		2013	2018	Clinical epidemiology, course of diseases		Endocrine, nutritional and metabolic diseases	Diseases of the respiratory system	type 1 diabetes, influenza		
Heerspink 2020	Kidney outcomes associated with use of SGLT2 inhibitors in real-world clinical practice (CVD-REAL 3): a multinational observational cohort study	MDV	five countries	Nationwide		2014	2017	Intervention effect		Endocrine, nutritional and metabolic diseases	Diseases of the genitourinary system	type 2 diabetes, Kidney outcomes	Others	age 18 years or older
Miyazaki 2015	Does antihypertensive treatment with renin-angiotensin system inhibitors prevent the development of diabetic kidney disease?	Others	health insurance societies located in the Japanese prefectures of Fukuoka and Shizuoka	Prefecture	Fukuoka and Shizuoka	2011	2013	Intervention effect		Endocrine, nutritional and metabolic diseases	Diseases of the genitourinary system	diabetic kidney disease	Others	≥40 years of age
Ono 2020	Association Between Routine Nephropathy Monitoring and Subsequent Change in Estimated Glomerular Filtration Rate in Patients With Diabetes Mellitus: A Japanese Non-Elderly Cohort Study	JMDC		Nationwide		2005	2016	Quality of care	Intervention effect	Endocrine, nutritional and metabolic diseases	Diseases of the genitourinary system	Diabetes, non-diabetic kidney diseases	Others	age ≥18 years
Sugiyama 2019	Risk-Stratified Incidence of Renal Replacement Therapy Initiation: A Longitudinal Analysis Using Medical Claims and Health Checkup Data	NHI/LSEHS	NHI	Municipality	a large city within the Tokyo	2012	2017	Clinical epidemiology, course of diseases	Medical treatment status	Endocrine, nutritional and metabolic diseases	Diseases of the genitourinary system	diabetes, Renal Replacement Therapy	Others	aged 40-74 years
Yamamoto 2019	Overt Proteinuria, Moderately Reduced eGFR and Their Combination Are Predictive of Severe Diabetic Retinopathy or Diabetic Macular Edema in Diabetes	JMDC		Nationwide		2008	2017	Clinical epidemiology, course of diseases		Endocrine, nutritional and metabolic diseases	Diseases of the genitourinary system	diabetes, overt proteinuria, moderately reduced eGFR		
Chang 2020	Incidence and causes of mildly to moderately elevated aminotransferase in Japanese patients with type 2 diabetes	MDV		Nationwide		2010	2016	Clinical epidemiology, course of diseases		Endocrine, nutritional and metabolic diseases	Diseases of the digestive system	Type 2 Diabetes, liver disease	Others	≥18 years of age
Sato 2016	Fracture risk and healthcare resource utilization and costs among osteoporosis patients with type 2 diabetes mellitus and without diabetes mellitus in Japan: retrospective analysis of a hospital claims database	MDV		Nationwide		2008	2013	Clinical epidemiology, course of diseases	Health economics	Endocrine, nutritional and metabolic diseases	Diseases of the musculoskeletal system and connective tissue	diabetes, osteoporosis	Others	aged 50 years or older
Takeuchi 2021	Sodium-glucose cotransporter-2 inhibitors and the risk of urinary tract infection among diabetic patients in Japan: Target trial emulation using a nationwide administrative claims database	NDB		Nationwide		2014	2015	Intervention effect		Endocrine, nutritional and metabolic diseases	Diseases of the genitourinary system	Diabetes, urinary tract infection (UTI)	Others	aged ≥40 years
Nozawa 2021	Antidyslipidemic Drug Prescriptions and Lipid Control Status After Unfavorable Annual Health Checkup Results: A Retrospective Cohort Study Using a Health Insurance Database	Others	MinaCare	Nationwide		2015	2017	Patient health service utilization		Endocrine, nutritional and metabolic diseases		dyslipidemia	Others	aged 20–74 years
Umeda 2019	Medication Adherence/Persistence and Demographics of Japanese Dyslipidemia Patients on Statin-Ezetimibe as a Separate Pill Combination Lipid-Lowering Therapy - An Observational Pharmacy Claims Database Study	Others	the IQVIA National Prescription Audit database	Nationwide		2015	2018	Patient health service utilization		Endocrine, nutritional and metabolic diseases		dyslipidemia	Others	aged ≥20 years
Wake 2018	Treatment patterns in hyperlipidaemia patients based on administrative claim databases in Japan	Others	JMDC and MDV	Nationwide		2014	2015	Medical treatment status	Patient health service utilization	Endocrine, nutritional and metabolic diseases		hyperlipidaemia (ICD-10: E78)	Others	aged ≥18 years
Umeda 2018	Low-Density Lipoprotein Cholesterol Goal Attainment Rates by Initial Statin Monotherapy Among Patients With Dyslipidemia and High Cardiovascular Risk in Japan - A Retrospective Database Analysis	MDV		Nationwide		2012	2016	Medical treatment status	Quality of care	Endocrine, nutritional and metabolic diseases	Diseases of the circulatory system	Dyslipidemia and High Cardiovascular Risk	Others	aged ≥20 years
Ito 2020	Prevalence of gout and asymptomatic hyperuricemia in the pediatric population: a cross-sectional study of a Japanese health insurance database	JMDC		Nationwide		2016	2017	Clinical epidemiology, course of diseases	Medical treatment status	Endocrine, nutritional and metabolic diseases	Diseases of the musculoskeletal system and connective tissue	gout and asymptomatic hyperuricemia	Children	0–18 years of age

Koto 2020	Real-world treatment of gout and asymptomatic hyperuricemia: A cross-sectional study of Japanese health insurance claims data	JMDC		Nationwide		2016	2017	Clinical epidemiology, course of diseases	Medical treatment status	Endocrine, nutritional and metabolic diseases	Diseases of the musculoskeletal system and connective tissue	gout and asymptomatic hyperuricemia	Others	Age 18–65 years
Hakoda 2019	Increasing trend of asymptomatic hyperuricemia under treatment with urate-lowering drugs in Japan	JMDC		Nationwide		2010	2014	Clinical epidemiology, course of diseases		Endocrine, nutritional and metabolic diseases	Diseases of the musculoskeletal system and connective tissue	gout and asymptomatic hyperuricemia		
Teramoto 2018	Treatment Patterns and Lipid Profile in Patients with Familial Hypercholesterolemia in Japan	MDV		Nationwide		2013	2013	Medical treatment status		Endocrine, nutritional and metabolic diseases		familial hypercholesterolemia	Others	aged ≥20 years
Sako 2017	Hospitalization with hypoglycemia in patients without diabetes mellitus: A retrospective study using a national inpatient database in Japan, 2008-2012	DPC		Nationwide		2008	2013	Clinical epidemiology, course of diseases		Endocrine, nutritional and metabolic diseases		hypoglycemia	Others	aged ≥15 years
Kanda 2020	Clinical and Economic Burden of Hyperkalemia: A Nationwide Hospital-Based Cohort Study in Japan	MDV		Nationwide		2008	2018	Clinical epidemiology, course of diseases	Health economics	Endocrine, nutritional and metabolic diseases		hyperkalemia	Others	aged ≥18 years
Kashihara 2019	Hyperkalemia in Real-World Patients Under Continuous Medical Care in Japan	MDV		Nationwide		2008	2017	Clinical epidemiology, course of diseases	Medical treatment status	Endocrine, nutritional and metabolic diseases		hyperkalemia	Others	aged ≥18 years
Higa 2020	A Retrospective, Cross-Sectional Study on the Prevalence of Hyperuricemia Using a Japanese Healthcare Database	Others	MinaCare	Nationwide		2010	2014	Clinical epidemiology, course of diseases		Endocrine, nutritional and metabolic diseases		Hyperuricemia		
Tsujimura 2014	Predictors of hyperglycaemic individuals who do not follow up with physicians after screening in Japan: a cohort study	JMDC		Nationwide		2005	2010	Patient health service utilization	Clinical epidemiology, course of diseases	Endocrine, nutritional and metabolic diseases		hyperglycaemic	Others	aged 20 to 68 years
Tatebe 2020	Hypoglycemia associated with pivalate-conjugated antibiotics in young children: A retrospective study using a medical and pharmacy claims database in Japan	JMDC		Nationwide		2011	2013	Intervention effect		Endocrine, nutritional and metabolic diseases		Hypoglycemia (PCA-induced hypoglycemia)	Children	1month-5year of age
Hakoda 2013	[A cause for age-dependent differences in the frequency of hyperuricemia--An analysis utilizing a database of health insurance claims]	JMDC		Nationwide		2008	2008	Clinical epidemiology, course of diseases		Endocrine, nutritional and metabolic diseases		hyperuricemia	Others	aged ≥20 years
Momo 2019	Risk factors affecting the failed low-density lipoprotein level achievement rate in working-age male population at high cardiovascular risk	JMDC		Nationwide		2004	2016	Medical treatment status	Patient health service utilization	Endocrine, nutritional and metabolic diseases		low-density lipoprotein (LDL)	Others	aged 18-60 years
Ibayashi 2020	[A descriptive study of mitochondrial diseases using DPC data]*	DPC	DPC Research Institute	Nationwide		2014	2015	Clinical epidemiology, course of diseases		Endocrine, nutritional and metabolic diseases	Diseases of the nervous system	mitochondrial disease		age group
Nishikawa 2020	Combined Associations of Body Mass Index and Metabolic Health Status on Medical and Dental Care Days and Costs in Japanese Male Employees: A 4-Year Follow-Up Study	Others	health insurance claims data; the Kobe Steel Health Insurance Association	Nationwide		2010	2013	Health economics		Endocrine, nutritional and metabolic diseases		body mass index (BMI) levels and metabolic dysfunction		
Okamoto 2013	Effects of health guidance on outpatient and pharmacy expenditures: a disease- and drug-specific 3-year observational study using propensity-score matching	Others	a health insurance society	Others	a health insurance society in the manufacturing	2008	2012	Health economics	Intervention effect	Endocrine, nutritional and metabolic diseases		metabolic syndrome	Others	age ≥40 years
Funayama 2016	[[How are the effects of long-term care prevention and health guidance evaluated?] The relationship between waist circumference and average annual medical expenses using specific health checkup results and claim data]*	JHIA		Municipality	Yokohama city	2012	2013	Health economics		Endocrine, nutritional and metabolic diseases		abdominal circumference	Others	35-74 years old
Okamoto 2013	[Evaluation of the health check up and guidance program through linkage with health insurance claims]	NHI/LSEHS	National Health Insurance, Health Care System for the Late Elderly	Municipality	Mishima City	2008	2012	Intervention effect		Endocrine, nutritional and metabolic diseases		metabolic syndrome	Others	aged 40 to 74
Hurst 2018	Effects of changes in eating speed on obesity in patients with diabetes: a secondary analysis of longitudinal health check-up data	JMDC		Nationwide		2008	2013	Clinical epidemiology, course of diseases		Endocrine, nutritional and metabolic diseases		obesity		
Takeuchi 2010	[Relationship between community-based dental health programs and health care costs for the metabolic syndrome]	NHI/LSEHS	NHI	Prefecture	Okayama	1997	2007	Health economics	Health policy evaluation and utilization	Endocrine, nutritional and metabolic diseases		metabolic syndrome	Others	age ≥40 years
Fujita 2018	Medical costs attributable to overweight and obesity in Japanese individuals	NHI/LSEHS	NHI	Municipality	Chiba City	2012	2016	Health economics		Endocrine, nutritional and metabolic diseases		overweight and obesity	Others	aged 40–69 years
Sairenchi 2017	Impact and attribute of each obesity-related cardiovascular risk factor in combination with abdominal obesity on total health expenditures in adult Japanese National Health insurance beneficiaries: The Ibaraki Prefectural health study	NHI/LSEHS	NHI	Prefecture	Ibaraki	2009	2013	Health economics		Endocrine, nutritional and metabolic diseases	Diseases of the circulatory system	abdominal obesity, cardiovascular risk	Others	aged 40–75 years
Itoh 2021	Metabolically Healthy Obesity and the Risk of Cardiovascular Disease in the General Population - Analysis of a Nationwide Epidemiological Database	JMDC		Nationwide		2005	2018	Clinical epidemiology, course of diseases		Endocrine, nutritional and metabolic diseases	Diseases of the circulatory system	metabolic disorders, cardiovascular disease	Others	aged ≥20 years
Hattori 2020	Pituitary surgery's epidemiology using a national inpatient database in Japan	DPC		Nationwide		2010	2016	Medical treatment status	Intervention effect	Endocrine, nutritional and metabolic diseases	Others	pituitary surgery		age group
Senda 2020	Early administration of glucocorticoid for thyroid storm: analysis of a national administrative database	DPC		Nationwide		2013	2017	Intervention effect		Endocrine, nutritional and metabolic diseases		thyroid storm	Others	age ≥16 years
Sasabuchi 2020	A survey on total parenteral nutrition in 55,000 hospitalized patients: Retrospective cohort study using a medical claims database	MDV		Nationwide		2009	2018	Clinical epidemiology, course of diseases		Endocrine, nutritional and metabolic diseases	Others	underfeeding, total parenteral nutrition	Others	aged ≥18 years
Ogawa 2019	Malnutrition-Related Health Care Cost in Japan: An Analysis of Health Insurance Claims Data	NHI/LSEHS	the community based health insurance and the Medical Care System for the Advanced Elderly	Nationwide	several municipalities throughout Japan	2013	2016	Clinical epidemiology, course of diseases	Health economics	Endocrine, nutritional and metabolic diseases	Others	strictly diagnosed malnutrition and disease-associated malnutrition		all ages
Itoh 2017	Vitamin D-Deficient Rickets in Japan	JMDC		Nationwide		2005	2014	Clinical epidemiology, course of diseases		Endocrine, nutritional and metabolic diseases		vitamin D deficiency or vitamin D–deficient rickets	Children	aged 0 to 15 years
5) Mental, Behavioral and Neurodevelopmental disorders (F00-F99)														
Obara 2015	Prescription of drugs for children with attention-deficit/hyperactivity disorder(ADHD) in Japan: a study based on health insurance claims data	JMDC		Nationwide		2005	2010	Medical treatment status	Health policy evaluation and utilization	Mental, Behavioral and Neurodevelopmental disorders		attention deficit hyperactivity disorder (ADHD)	Children	aged 2 to 17 years
Yoshida 2020	Drug Prescriptions for Children With ADHD in Japan: A Study Based on Health Insurance Claims Data Between 2005 and 2015	JMDC		Nationwide		2005	2015	Medical treatment status		Mental, Behavioral and Neurodevelopmental disorders		ADHD (ICD-10 code: F900)	Children	aged 1 to 17 years
Imagawa 2018	Treatment patterns, health care resource utilization, and costs in Japanese adults with attention-deficit hyperactivity disorder treated with atomoxetine	JMDC		Nationwide		2012	2015	Medical treatment status	Health economics	Mental, Behavioral and Neurodevelopmental disorders		attention-deficit/hyperactivity disorder (ADHD)	Others	at least 18 years of age
Nakamura 2013	Body mass index and in-hospital mortality in anorexia nervosa: data from the Japanese Diagnosis Procedure Combination database	DPC		Nationwide		2010	2010	Clinical epidemiology, course of diseases		Mental, Behavioral and Neurodevelopmental disorders		anorexia nervosa		

Yamamoto-Sasaki 2020	Association between antidepressant use during pregnancy and congenital anomalies in children: A retrospective cohort study based on Japanese claims data	JMDC		Nationwide		2005	2014	Intervention effect		Mental, Behavioral and Neurodevelopmental disorders	Congenital malformations, deformations and chromosomal abnormalities	congenital anomaly, antidepressants		
Yamamoto-Sasaki 2019	Association between antidepressant use during pregnancy and autism spectrum disorder in children: a retrospective cohort study based on Japanese claims data	JMDC		Nationwide		2005	2014	Intervention effect		Mental, Behavioral and Neurodevelopmental disorders	Pregnancy, childbirth and the puerperium	antidepressant use during pregnancy and autism spectrum disorder in children		
Ishikawa 2020	Antidepressant prescriptions for prenatal and postpartum women in Japan: A health administrative database study	JMDC		Nationwide		2005	2016	Medical treatment status		Mental, Behavioral and Neurodevelopmental disorders	Pregnancy, childbirth and the puerperium	antidepressant prescriptions to prenatal and postpartum women		
Ogino 2020	Impact of class-level labelling change on prescriptions of antidepressants for adolescents: An interrupted time-series study using a health insurance claims database in Japan, 2005-2013	JMDC	Vital Statistics records	Nationwide		2005	2013	Health policy evaluation and utilization		Mental, Behavioral and Neurodevelopmental disorders	Others	antidepressants, suicide	Others	aged 10 to 24 years (target population)
Su 2020	Incidence, prevalence and prescription patterns of antipsychotic medications use in Asia and US: A cross-nation comparison with common data model	JMDC		Nationwide	Taiwan, Hong Kong, Japan, and the United States	2007	2014	Medical treatment status	Socioeconomic comparison	Mental, Behavioral and Neurodevelopmental disorders		antipsychotic medications use	Others	age<65
Kochi 2017	Trends in antipsychotic prescriptions for Japanese outpatients during 2006-2012: a descriptive epidemiological study	Others	claims data from 1038 community pharmacies	Nationwide		2006	2012	Medical treatment status		Mental, Behavioral and Neurodevelopmental disorders		antipsychotic prescriptions	Others	18–64 years old, ≥65 years old
Kuroda 2019	Antipsychotic use and related factors among people with dementia aged 75 years or older in Japan: A comprehensive population-based estimation using medical and long-term care data	NHI/LSEHS	medical and LTC data	Municipality	a middle-sized suburban city in the Tokyo metropolitan	2012	2013	Medical treatment status		Mental, Behavioral and Neurodevelopmental disorders	Diseases of the nervous system	antipsychotics among older adults with dementia	Older persons	over 75 years (65 to 74 years)
Okumura 2018	Glucose and Prolactin Monitoring in Children and Adolescents Initiating Antipsychotic Therapy	NDB		Nationwide		2014	2015	Medical treatment status		Mental, Behavioral and Neurodevelopmental disorders	Endocrine, nutritional and metabolic diseases	antipsychotics, diabetes	Children	aged ≤18 years
Takeuchi 2015	Atypical Antipsychotics and the Risk of Hyperlipidemia: A Sequence Symmetry Analysis	JMDC		Nationwide		2005	2013	Intervention effect		Mental, Behavioral and Neurodevelopmental disorders	Endocrine, nutritional and metabolic diseases	atypical antipsychotic medication, hyperlipidemia		
Takahashi 2020	Factors associated with high-dose antipsychotic prescriptions in outpatients with schizophrenia: An analysis of claims data from a Japanese prefecture	NHI/LSEHS	National Health Insurance and Long Life Medical Care System	Prefecture	a single prefecture	2014	2015	Medical treatment status		Mental, Behavioral and Neurodevelopmental disorders		high-dose antipsychotic prescriptions in outpatients with schizophrenia	Others	aged 18 years and older
Qiu 2018	Antipsychotic polypharmacy in the treatment of schizophrenia in China and Japan	JMDC		Nationwide	China and Japan	2010	2014	Medical treatment status	Socioeconomic comparison	Mental, Behavioral and Neurodevelopmental disorders		schizophrenia (antipsychotic polypharmacy)		
Yokoyama 2020	Association Between Antipsychotics and Osteoporosis Based on Real-World Data	JMDC	FAERS	Nationwide		2005	2017	Intervention effect		Mental, Behavioral and Neurodevelopmental disorders	Diseases of the musculoskeletal system and connective tissue	antipsychotic use and onset of osteoporosis		
Inoue 2021	Real-World Treatment Patterns and Adherence to Oral Medication Among Patients with Bipolar Disorders: A Retrospective, Observational Study Using a Healthcare Claims Database	JMDC		Nationwide		2013	2018	Medical treatment status	Patient health service utilization	Mental, Behavioral and Neurodevelopmental disorders		bipolar disorder (F30 or 31)		
Shinjo 2019	Risk factors for early unplanned readmission in patients with bipolar disorder: A retrospective observational study	DPC		Nationwide		2012	2014	Medical treatment status	Health policy evaluation and utilization	Mental, Behavioral and Neurodevelopmental disorders		bipolar patients (ICD-10; F31)	Others	aged ≥15 years
Ning 2014	[The length of stay for hospitalized patients with dementia in Fukuoka Prefecture : A crosssectional study]	NHI/LSEHS	Fukuoka National Health Insurance Organization	Prefecture	Fukuoka	2009	2010	Medical treatment status	Health policy evaluation and utilization	Mental, Behavioral and Neurodevelopmental disorders	Diseases of the nervous system	dementia		
Morioka 2021	Dementia and patient outcomes after hip surgery in older patients: A retrospective observational study using nationwide administrative data in Japan	DPC	Diagnosis Procedure Combination database and reports of the medical functions of hospital beds	Nationwide		2016	2017	Clinical epidemiology, course of diseases		Mental, Behavioral and Neurodevelopmental disorders	Diseases of the nervous system	Dementia (after hip surgery)	Older persons	aged 65 or older
Nakaoku 2021	Predictors of New Dementia Diagnoses in Elderly Individuals: A Retrospective Cohort Study Based on Prefecture-Wide Claims Data in Japan	NHI/LSEHS	administrative claims database of the medical care system for the elderly	Prefecture	Niigata	2012	2016	Clinical epidemiology, course of diseases		Mental, Behavioral and Neurodevelopmental disorders	Diseases of the nervous system	dementia	Older persons	aged ≥75 years
Morioka 2020	Structure and process of dementia care and patient outcomes after hip surgery in elderly people with dementia: A retrospective observational study in Japan	DPC		Nationwide		2016	2017	Health policy evaluation and utilization		Mental, Behavioral and Neurodevelopmental disorders	Diseases of the nervous system	dementia (underwent hip surgeries)	Older persons	aged 65 years or older
Tashiro 2020	[Regional differences in dementia seen from health insurance claims data: A study on all old-old people in Niigata Prefecture]*	NHI/LSEHS		Prefecture	Niigata	2012	2016	Health economics	Socioeconomic comparison	Mental, Behavioral and Neurodevelopmental disorders	Diseases of the nervous system	dementia	Older persons	75 years old and older
Maeda 2018	Surveillance of First-Generation H1-Antihistamine Use for Older Patients with Dementia in Japan: A Retrospective Cohort Study	NHI/LSEHS	Fukuoka Late Elders' Health Insurance	Prefecture	Fukuoka	2010	2013	Medical treatment status		Mental, Behavioral and Neurodevelopmental disorders		dementia	Older persons	aged 75 years or older
Maeda 2018	Effects of gastrostomy fee schedule revision on artificial nutrition routes among older people with dementia in Japan: A time series observational study	NHI/LSEHS	Fukuoka Late Elders' Health Insurance	Prefecture	Fukuoka	2010	2016	Medical treatment status	Health policy evaluation and utilization	Mental, Behavioral and Neurodevelopmental disorders		dementia	Older persons	aged ≥75 years and those aged 65–74 years with a specific disability
Sakata 2018	Thyroid function tests before prescribing anti-dementia drugs: a retrospective observational study	NDB		Nationwide		2015	2016	Medical treatment status	Quality of care	Mental, Behavioral and Neurodevelopmental disorders	Diseases of the nervous system	dementia	Older persons	aged ≥65 years
Lin 2015	The effects of dementia and long-term care services on the deterioration of care-needs levels of the elderly in Japan	NHI/LSEHS	National Healthcare Insurance Database and Old-Old Adults Medical Care Program Database	Prefecture	Kyoto	2010	2011	Clinical epidemiology, course of diseases	Health policy evaluation and utilization	Mental, Behavioral and Neurodevelopmental disorders	Diseases of the nervous system	dementia	Older persons	aged 65 years and above
Sakata 2017	Job Loss After Diagnosis of Early-Onset Dementia: A Matched Cohort Study	JMDC		Nationwide		2013	2015	Others		Mental, Behavioral and Neurodevelopmental disorders	Diseases of the nervous system	Early-onset dementia	Others	aged 40–59 years

Mine 2020	Hospitalization and discharge routes of elderly hip fracture patients with and without dementia: a nationwide cross-sectional exploratory study using the Japanese Diagnostic Procedure Combination database	DPC		Nationwide		2014	2016	Clinical epidemiology, course of diseases		Mental, Behavioral and Neurodevelopmental disorders	Injury, poisoning and certain other consequences of external causes	hip fracture, dementia	Older persons	aged 65 years or older
Tomioka 2020	An analysis of equity in treatment of hip fractures for older patients with dementia in acute care hospitals: observational study using nationwide hospital claims data in Japan	DPC		Nationwide		2014	2018	Medical treatment status		Mental, Behavioral and Neurodevelopmental disorders	Injury, poisoning and certain other consequences of external causes	hip fracture, dementia	Older persons	65 years old and older
Tsuda 2015	Association between dementia and postoperative complications after hip fracture surgery in the elderly: analysis of 87,654 patients using a national administrative database	DPC		Nationwide		2007	2010	Clinical epidemiology, course of diseases		Mental, Behavioral and Neurodevelopmental disorders	Injury, poisoning and certain other consequences of external causes	neck fracture and intertrochanteric fracture, dementia	Others	age ≥70 years
Maeda 2016	Investigation into the causes of indwelling urethral catheter implementation and its effects on clinical outcomes and health care resources among dementia patients with pneumonia A retrospective cohort study	NHI/LSEHS	Fukuoka Late Elders' Health Insurance	Prefecture	Fukuoka	2010	2013	Quality of care	Health economics	Mental, Behavioral and Neurodevelopmental disorders	Diseases of the respiratory system	dementia patients with pneumonia	Older persons	75 years or older
Song 2017	Association between eicosapentaenoic acid (EPA) medication intake and new onset of depression among Japanese patients with hyperlipidemia: A 3-year follow-up study	JMDC		Nationwide		2005	2014	Intervention effect		Mental, Behavioral and Neurodevelopmental disorders	Endocrine, nutritional and metabolic diseases	depression, hyperlipidemia	Others	aged ≥18 years
Inada 2021	Effect of residual insomnia and use of hypnotics on relapse of depression: a retrospective cohort study using a health insurance claims database	JMDC		Nationwide		2006	2017	Clinical epidemiology, course of diseases	Medical treatment status	Mental, Behavioral and Neurodevelopmental disorders		residual insomnia, relapse of depression	Others	aged 20-65 years
Yamato 2021	Patterns of hypnotic prescribing for residual insomnia and recurrence of major depressive disorder: a retrospective cohort study using a Japanese health insurance claims database	JMDC		Nationwide		2005	2018	Intervention effect	Medical treatment status	Mental, Behavioral and Neurodevelopmental disorders		residual insomnia, recurrence of major depressive disorder	Others	20–56 years
Fujita 2019	Risk of depressive disorders after tobacco smoking cessation: a retrospective cohort study in Fukuoka, Japan	JHIA	The Fukuoka branch of the Japan Health Insurance Association	Prefecture	Fukuoka	2010	2014	Clinical epidemiology, course of diseases		Mental, Behavioral and Neurodevelopmental disorders		depressive disorders	Others	30-69 years old
Hirano 2018	Possibility of Database Research as a Means of Pharmacovigilance in Japan Based on a Comparison with Sertraline Postmarketing Surveillance	Others	MinaCare, postmarketing surveillance results	Nationwide		2008	2013	Health policy evaluation and utilization	Research methodology	Mental, Behavioral and Neurodevelopmental disorders		depression/depressive symptoms and panic disorder		
Kimura 2015	Treatment persistence with antidepressants in Japan: Assessment of the first 60 days of treatment based on pharmacy claims data from reimbursement database	Others	JMIRI database	Nationwide		2012	2012	Patient health service utilization		Mental, Behavioral and Neurodevelopmental disorders		depression	Others	aged ≥20 years
Kitanishi 2021	Patient journey through cases of depression from claims database using machine learning algorithms	JMDC		Nationwide		2005	2018	Prediction model	Clinical epidemiology, course of diseases	Mental, Behavioral and Neurodevelopmental disorders		depression		
Yamazaki 2021	Early Electroconvulsive Therapy in Patients With Major Depressive Disorder: A Propensity Score-Matched Analysis Using a Nationwide Inpatient Database in Japan	DPC		Nationwide		2011	2018	Intervention effect	Health economics	Mental, Behavioral and Neurodevelopmental disorders		major depressive disorder		
Mahlich 2018	Estimating Prevalence and Healthcare Utilization for Treatment-Resistant Depression in Japan: A Retrospective Claims Database Study	JMDC		Nationwide		2012	2015	Clinical epidemiology, course of diseases	Health economics	Mental, Behavioral and Neurodevelopmental disorders		treatment-resistant depression	Others	18–60 years of age
Furukawa 2013	Prescription patterns following first-line new generation antidepressants for depression in Japan: a naturalistic cohort study based on a large claims database	JMDC		Nationwide		2008	2011	Medical treatment status	Quality of care	Mental, Behavioral and Neurodevelopmental disorders		unipolar depression	Others	Aged 18 or older
Onishi 2013	Psychotropic prescription patterns among patients diagnosed with depressive disorder based on claims database in Japan	JMDC		Nationwide		2008	2011	Medical treatment status	Quality of care	Mental, Behavioral and Neurodevelopmental disorders		depressive disorder	Others	age ≥18 years
Otsubo 2012	[A fact-finding survey on pain comorbidity in patients with depression: A retrospective study using a medical fee claims database]*	MDV		Nationwide		2003	2010	Clinical epidemiology, course of diseases	Medical treatment status	Mental, Behavioral and Neurodevelopmental disorders	Others	Depression, Pain	Others	age ≥20 years
Michihata 2014	Comparison between enteral nutrition and intravenous hyperalimentation in patients with eating disorders: results from the Japanese diagnosis procedure combination database	DPC		Nationwide		2010	2013	Intervention effect		Mental, Behavioral and Neurodevelopmental disorders		Eating disorders	Others	aged 10-59 years
Hosomi 2016	[Association of Antipsychotic Use with Extrapyramidal Symptoms: Data Mining of the Japanese National Insurance Claims Database]	NDB		Nationwide		2010	2012	Intervention effect		Mental, Behavioral and Neurodevelopmental disorders	Diseases of the nervous system	Extrapyramidal Symptoms (Antipsychotic Use)		
Hayashi 2020	How was cognitive behavioural therapy for mood disorder implemented in Japan? A retrospective observational study using the nationwide claims database from FY2010 to FY2015	NDB	Aggregated data	Nationwide		2010	2015	Medical treatment status		Mental, Behavioral and Neurodevelopmental disorders		mood disorder		
Fujita 2019	Inequality within a community at the neighborhood level and the incidence of mood disorders in Japan: a multilevel analysis	NHI/LSEHS	NHI	Municipality	Chiba City	2013	2016	Clinical epidemiology, course of diseases	Socioeconomic comparison	Mental, Behavioral and Neurodevelopmental disorders		mood disorder (F30–F39)	Others	aged 20 to 69 years
Sakakibara 2019	[Study on the Risk Factors for Postoperative Delirium Using the National Health Insurance Claims Database in Japan]	NDB		Nationwide		2015	2016	Clinical epidemiology, course of diseases		Mental, Behavioral and Neurodevelopmental disorders		postoperative delirium		
Yamanashi 2020	The role of mental disease on the association between multimorbidity and medical expenditure	NHI/LSEHS	Kokuho-Database System	Municipality	Goto city, western Japan	2016	2017	Clinical epidemiology, course of diseases	Health economics	Mental, Behavioral and Neurodevelopmental disorders	Others	mental disease, multimorbidity	Others	ageed 0-75 years
Satoh 2016	Prescription trends in children with pervasive developmental disorders: a claims data-based study in Japan	JMDC		Nationwide		2005	2010	Medical treatment status		Mental, Behavioral and Neurodevelopmental disorders		pervasive developmental disorders	Children	younger than 18 years of age
Muramatsu 2019	Relationship between treatment and period of absence among employees on sick leave due to mental disease	Others	health insurance claims data provided by one health insurance society, data on accident and sickness benefits paid during the sick leave	Others	a property insuranc e compan y group	2008	2014	Intervention effect		Mental, Behavioral and Neurodevelopmental disorders		mental disease, psychotherapy		
Okumura 2019	Psychiatric Admissions and Length of Stay During Fiscal Years 2014 and 2015 in Japan: A Retrospective Cohort Study Using a Nationwide Claims Database	NDB	630 survey	Nationwide		2014	2016	Medical treatment status		Mental, Behavioral and Neurodevelopmental disorders		psychiatric care		age group
Inaida 2018	Psychogenic non-epileptic seizures in Japan: Trends in prevalence, delay in diagnosis, and frequency of hospital visits	JMDC		Nationwide		2006	2014	Clinical epidemiology, course of diseases	Medical treatment status	Mental, Behavioral and Neurodevelopmental disorders		Psychogenic non-epileptic seizures		all ages
Okumura 2018	Association of high psychiatrist staffing with prolonged hospitalization, follow-up visits, and readmission in acute psychiatric units: a retrospective cohort study using a nationwide claims database	NDB		Nationwide		2014	2016	Health policy evaluation and utilization		Mental, Behavioral and Neurodevelopmental disorders		Patients newly admitted to acute psychiatric units		
Shinjo 2017	Factors affecting prolonged length of stay in psychiatric patients in Japan: A retrospective observational study	DPC		Nationwide		2012	2014	Clinical epidemiology, course of diseases	Health policy evaluation and utilization	Mental, Behavioral and Neurodevelopmental disorders		psychiatric patients	Others	aged ≥15 years
Yamaoka 2017	[Mental Health of Small and Medium Enterprise Workers: An Empirical Analysis Using Medical Claims Data from Japan Health Insurance Association]	JHIA	Japan Health Insurance Association Hyogo Branch	Prefecture	Hyogo	2013	2013	Clinical epidemiology, course of diseases		Mental, Behavioral and Neurodevelopmental disorders		mental disorder		

Matsuda 2016	Refinement of DPC classification of psychiatric disorders	DPC		Nationwide		2011	2012	Health economics		Mental, Behavioral and Neurodevelopmental disorders		psychiatric MDC		age category
Murakami 2016	Importance of psychiatric disorders as comorbidities and complications among the acute in-patient cases in Japan	DPC		Nationwide		2010	2010	Clinical epidemiology, course of diseases		Mental, Behavioral and Neurodevelopmental disorders		psychiatric problem among the acute in-patient cases		
Murata 2015	[Analysis of outcomes of acute abdominal patients with mental disorder using Diagnosis Procedure Combination database]	DPC		Nationwide		2009	2011	Clinical epidemiology, course of diseases		Mental, Behavioral and Neurodevelopmental disorders	Others	acute abdomen, psychiatric complications		age group
Inagaki 2014	[Metabolic and Lithium Monitoring in Japanese Psychiatric Outpatient Clinics]	Others	health insurance claims of psychiatric outpatients (a database constructed from research on medical policies)	Nationwide		2006	2006	Medical treatment status	Quality of care	Mental, Behavioral and Neurodevelopmental disorders		psychiatric outpatient		
Matsuda 2010	[Evaluation of Psychiatric medicine under the DPC system]	DPC		Nationwide		2008	2008	Medical treatment status	Health economics	Mental, Behavioral and Neurodevelopmental disorders	Diseases of the nervous system	Psychiatric disorders		
Baba 2020	[First-Time Use versus Continuing Use of Benzodiazepine Receptor Agonists in Japan]	JMDC		Nationwide		2013	2014	Medical treatment status		Mental, Behavioral and Neurodevelopmental disorders		Benzodiazepine receptor agonists	Others	aged between 40 and 74
Okumura 2016	Prevalence, prescribed quantities, and trajectory of multiple prescriber episodes for benzodiazepines: A 2-year cohort study	JMDC		Nationwide		2012	2014	Medical treatment status		Mental, Behavioral and Neurodevelopmental disorders		patients with a benzodiazepine	Others	aged 0-74 years
Takeshima 2016	Continuation and discontinuation of benzodiazepine prescriptions: A cohort study based on a large claims database in Japan	JMDC		Nationwide		2005	2014	Medical treatment status	Patient health service utilization	Mental, Behavioral and Neurodevelopmental disorders		new benzodiazepines users	Others	Aged 18 or older
Takeuchi 2012	[Relationship between Implementation of Community-based Dental Health Programs and Health Care Costs for Mental and Behavioral Disorders]	NHI/LSEHS	NHI	Prefecture	Okayama	1997	2009	Health economics	Health policy evaluation and utilization	Mental, Behavioral and Neurodevelopmental disorders	Diseases of the digestive system	Mental and behavioral disorders, dental	Others	age <75 years
Shigemi 2021	Suicide attempts during pregnancy and perinatal outcomes	DPC		Nationwide		2016	2018	Clinical epidemiology, course of diseases		Mental, Behavioral and Neurodevelopmental disorders	Pregnancy, childbirth and the puerperium	suicide attempts during pregnancy	Others	aged ≥15 years
Shigemi 2020	Suicide Attempts Among Pregnant and Postpartum Women in Japan: A Nationwide Retrospective Cohort Study	DPC		Nationwide		2016	2018	Clinical epidemiology, course of diseases		Mental, Behavioral and Neurodevelopmental disorders	Pregnancy, childbirth and the puerperium	Suicide attempts among pregnant and postpartum women	Others	aged ≥13 years
Asami 2016	[A Perspective of Pharmacovigilance Using the Healthcare Claim Database, Based on Comparison with Sertraline Post Marketing Surveillance]	Others	MinaCare, Sertraline Post Marketing Surveillance	Nationwide		2008	2013	Research methodology	Health policy evaluation and utilization	Mental, Behavioral and Neurodevelopmental disorders		Sertraline		
Ooba 2018	Prevalence of Therapeutic Drug Monitoring for Lithium and the Impact of Regulatory Warnings: Analysis Using Japanese Claims Database	JMDC		Nationwide		2005	2014	Quality of care	Health policy evaluation and utilization	Mental, Behavioral and Neurodevelopmental disorders		Therapeutic drug monitoring for lithium	Others	aged 20–74 years old
Hirano 2019	Changes in Prescription of Psychotropic Drugs After Introduction of Polypharmacy Reduction Policy in Japan Based on a Large-Scale Claims Database	Others	MinaCare	Nationwide		2011	2017	Medical treatment status	Health policy evaluation and utilization	Mental, Behavioral and Neurodevelopmental disorders		psychotropic drug prescribed patients	Others	age<75
Tanemura 2019	Pediatric off-label use of psychotropic drugs approved for adult use in Japan in the light of approval information regarding pediatric patients in the United States: a study of a pharmacy prescription database	Others	database of a company owning a nationwide chain pharmacies	Nationwide		2016	2017	Medical treatment status		Mental, Behavioral and Neurodevelopmental disorders		pediatric patients' off-label use of psychotropics approved only for adults	Children	aged <16 years
Orime 2018	Survey on the management of physical risks induced by psychotropic agents in Japan	JMDC		Nationwide		2014	2014	Medical treatment status	Quality of care	Mental, Behavioral and Neurodevelopmental disorders		persistent users of psychotropic agents	Others	aged 6-17 years and 18-74 years
Matsuda 2015	Analysis of Health Care Region for Psychiatric Care Based on the National Database	NDB		Prefecture	Fukuoka	2010	2010	Health policy evaluation and utilization		Mental, Behavioral and Neurodevelopmental disorders		Psychiatric medicine		
Arakawa 2015	[Actual status of prescription patterns of anxiolytics and hypnotics in outpatients using National Database of Health Insurance Claim Information and Specific Medical Checkups]	NDB	Sampling data	Nationwide		2011	2011	Medical treatment status		Mental, Behavioral and Neurodevelopmental disorders		anxiolytics, hypnotics		
Okumura 2014	[Geographical variation in sedative – hypnotic use among recipients of public assistance: a nationwide survey]	Others	Fact-finding Survey on Medical Assistance	Nationwide		2011	2012	Medical treatment status	Socioeconomic comparison	Mental, Behavioral and Neurodevelopmental disorders		sedative-hypnotic use		
Abe 2012	Prescription hypnotics and associated background factors in a large-scale Japanese database	JMDC		Nationwide		2005	2005	Medical treatment status		Mental, Behavioral and Neurodevelopmental disorders		prescribed hypnotics	Others	aged 20 to 74 years
Shimane 2012	Prevention of overlapping prescriptions of psychotropic drugs by community pharmacists	Others	Saitama Pharmaceutical Association (health insurance claims for dispensing)	Nationwide		2010	2010	Medical treatment status		Mental, Behavioral and Neurodevelopmental disorders		psychotropic drugs		
Yatomi 2020	Prescription patterns of psychotropics in patients receiving synthetic glucocorticoids	NDB	Sampling data	Nationwide		2015	2015	Medical treatment status	Intervention effect	Mental, Behavioral and Neurodevelopmental disorders	Endocrine, nutritional and metabolic diseases	psychotropics in patients receiving synthetic glucocorticoids		age-groups
Okumura 2017	Risk of recurrent overdose associated with prescribing patterns of psychotropic medications after nonfatal overdose	NDB		Nationwide		2012	2013	Intervention effect	Medical treatment status	Mental, Behavioral and Neurodevelopmental disorders	Injury, poisoning and certain other consequences of external causes	overdose (T360–T509), psychotropic medications	Others	19- to 64-year-old
Okumura 2015	Exposure to psychotropic medications prior to overdose: a case-control study	JMDC		Nationwide		2012	2013	Medical treatment status	Intervention effect	Mental, Behavioral and Neurodevelopmental disorders	Injury, poisoning and certain other consequences of external causes	drug poisoning, psychotropic medications	Others	aged 12–74 years
Inoue 2016	[Psychotropic Prescribing Practices for Children and Adolescents with Intellectual Disabilities : A Cohort Study Using a Large-scale Health Insurance Database]	JMDC		Nationwide		2012	2013	Medical treatment status		Mental, Behavioral and Neurodevelopmental disorders		Intellectual Disabilities, Psychotropic Prescribing Practices	Children	aged 3-17 years
Obara 2020	[Elucidation of the relationship between use of psychotropic drugs in pregnant women and malformation of infants]	JMDC		Nationwide		2005	2017	Medical treatment status		Mental, Behavioral and Neurodevelopmental disorders	Pregnancy, childbirth and the puerperium	psychotropic drug use in pregnant women		
Cheung 2017	Drug Utilization of Japanese Patients Diagnosed with Schizophrenia: An Administrative Database Analysis	MDV		Nationwide		2013	2015	Clinical epidemiology, course of diseases	Intervention effect	Mental, Behavioral and Neurodevelopmental disorders		schizophrenia	Others	aged between 18 and 65 years
Hata 2020	The 12-year trend report of antipsychotic usage in a nationwide claims database derived from four million people in Japan	JMDC		Nationwide		2005	2016	Medical treatment status	Quality of care	Mental, Behavioral and Neurodevelopmental disorders		schizophrenia	Others	aged 18 years or older
Iwata 2020	Treatment Persistence Between Long-Acting Injectable Versus Orally Administered Aripiprazole Among Patients with Schizophrenia in a Real-World Clinical Setting in Japan	JMDC		Nationwide		2014	2018	Patient health service utilization	Medical treatment status	Mental, Behavioral and Neurodevelopmental disorders		schizophrenia	Others	aged 18 years or more
Kusumi 2020	A propensity score matching analysis for cardio metabolic risk of antipsychotics in patients with schizophrenia using Japanese claims data	JMDC		Nationwide		2005	2017	Intervention effect		Mental, Behavioral and Neurodevelopmental disorders		schizophrenia	Others	aged ≤65 years
Takekita 2020	Rehospitalization Risk of Receptor-Affinity Profile in Antipsychotic Drug Treatment: A Propensity Score Matching Analysis Using a Japanese Employment-Based Health Insurance Database	JMDC		Nationwide		2005	2017	Medical treatment status	Intervention effect	Mental, Behavioral and Neurodevelopmental disorders		schizophrenia	Others	aged ≤65 years
Okumura 2018	Timely follow-up visits after psychiatric hospitalization and readmission in schizophrenia and bipolar disorder in Japan	NDB		Nationwide		2014	2015	Intervention effect	Patient health service utilization	Mental, Behavioral and Neurodevelopmental disorders		schizophrenia or bipolar disorder	Others	aged <65 years

Toyota 2017	[A study on the effect of psychiatry emergency medical care]	JHIA	Fukuoka branch of the Japanese Health Insurance Association	Prefecture	Fukuoka	2010	2014	Intervention effect	Health policy evaluation and utilization	Mental, Behavioral and Neurodevelopmental disorders		schizophrenia, psychiatric emergency care		
Kuwabara 2015	Adherence and rehospitalizations in patients with schizophrenia: evidence from Japanese claims data	JMDC		Nationwide		2009	2013	Intervention effect	Patient health service utilization	Mental, Behavioral and Neurodevelopmental disorders		schizophrenia diagnosis (ICD-10 code: F20)	Others	aged 18-65 years
Ochiai 2014	[Factors associated with high-dose prescription of antipsychotics in outpatients with schizophrenia -An analysis of claims data from a Japanese prefecture-]	NHI/LSEHS	National Health Insurance and Later-Stage Elderly Healthcare System	Prefecture	Kyoto	2011	2012	Medical treatment status	Health policy evaluation and utilization	Mental, Behavioral and Neurodevelopmental disorders		schizophrenia		
Nakamura 2013	Length of mechanical restraint following haloperidol injections versus oral atypical antipsychotics for the initial treatment of acute schizophrenia: a propensity-matched analysis from the Japanese diagnosis procedure combination database	DPC		Nationwide		2006	2009	Intervention effect		Mental, Behavioral and Neurodevelopmental disorders		acute schizophrenia		
Okumura 2013	[Antipsychotics prescribing patterns of patients with schizophrenia in Japan : Using the National Database of Health Insurance Claim Information and Specified Medical Checkups]	NDB	Sampling data	Nationwide		2011	2011	Medical treatment status	Quality of care	Mental, Behavioral and Neurodevelopmental disorders		schizophrenia		
Ye 2012	Antipsychotic monotherapy among outpatients with schizophrenia treated with olanzapine or risperidone in Japan: a health care database analysis	JMDC		Nationwide		2003	2009	Medical treatment status		Mental, Behavioral and Neurodevelopmental disorders		schizophrenia	Others	between 20 and 65 years old
Ye 2011	Characteristics of Outpatients Initiated on Olanzapine versus Risperidone in the Treatment of Schizophrenia in Japan: A Healthcare Database Analysis	JMDC		Nationwide		2003	2009	Medical treatment status		Mental, Behavioral and Neurodevelopmental disorders		schizophrenia or schizoaffective disorder	Others	adults aged 20 to 65
Iwata 2011	[Comparison between the treatment outcomes of monotherapy with new antipsychotic drugs using medical fee receipts]	JMDC		Nationwide		2007	2010	Patient health service utilization	Health economics	Mental, Behavioral and Neurodevelopmental disorders		schizophrenia	Others	aged 25 to 49 years
Nakahara 2010	[Analysis of the treatment outcomes for schizophrenia using medical fee receipts]	JMDC		Nationwide		2004	2006	Medical treatment status	Health economics	Mental, Behavioral and Neurodevelopmental disorders		schizophrenia (F20-29)	Others	aged 20 to 49 years
Suwa 2018	The association between smoking cessation outpatient visits and total medical costs: a retrospective, observational analysis of Japanese employee-based public health insurance data	JMDC		Nationwide		2005	2013	Intervention effect	Health economics	Mental, Behavioral and Neurodevelopmental disorders		nicotine dependence (F17.2)	Others	aged 21–60 years
Yuzuriha 2019	[Burden of illness for alcohol dependence using health insurance claims database in Japan]	JMDC	national statistics data (Patients Survey and Statistics of Medical Care Activities in Public Health Insurance)	Nationwide		2015	2017	Health economics		Mental, Behavioral and Neurodevelopmental disorders		alcohol dependence		
6) Diseases of the nervous system (G00-G99)														
Fukunishi 2020	Alzheimer-type dementia prediction by sparse logistic regression using claim data	Others	Health insurance and long-term care insurance databases in a large city	Municipality	a large city in the metropo litan area in	2012	2015	Prediction model	Clinical epidemiology, course of diseases	Diseases of the nervous system		Dementia in Alzheimer disease	Older persons	being over 75 years old
Kuboyama 2019	Medical expenses for cilostazol to treat Alzheimer's disease in Japan	NHI/LSEHS	National Health Insurance and the Long-term Care Insurance systems	Prefecture	a prefectu re in Japan	2010	2011	Health economics		Diseases of the nervous system		Cilostazol to treat Alzheimer's disease		
Kadohara 2017	Prescription Patterns of Medications for Alzheimer's Disease in Japan from 2010 to 2015: A Descriptive Pharmacy Claims Database Study	Others	pharmacy claims databases (operated by four community pharmacy chains)	Nationwide		2010	2014	Medical treatment status		Diseases of the nervous system		Alzheimer's disease	Others	20 years or older
Kadohara 2017	Diabetes mellitus and risk of early-onset Alzheimer's disease: a population-based case-control study	JMDC		Nationwide		2005	2016	Clinical epidemiology, course of diseases		Diseases of the nervous system	Endocrine, nutritional and metabolic diseases	Alzheimer's disease (AD), Diabetes mellitus	Others	aged 40–64 years
Fukamachi 2018	Exploratory evaluation of a commercially available Japanese medical record database as an external control for comparative clinical trials in new drug development: Amyotrophic lateral sclerosis	MDV	compared with phase 3 trials	Nationwide		2008	2016	Intervention effect	Research methodology	Diseases of the nervous system		Amyotrophic lateral sclerosis	Others	aged 18-75 years
Ishikawa 2020	Folic acid prescribed to prenatal and postpartum women who are also prescribed antiepileptic drugs in Japan: Data from a health administrative database	JMDC		Nationwide		2005	2016	Medical treatment status		Diseases of the nervous system	Pregnancy, childbirth and the puerperium	antiepileptic drugs, folic acid (prenatal and postpartum women)		
Ishikawa 2019	Examination of the prescription of antiepileptic drugs to prenatal and postpartum women in Japan from a health administrative database	JMDC		Nationwide		2005	2016	Medical treatment status		Diseases of the nervous system	Pregnancy, childbirth and the puerperium	antiepileptic drugs, prenatal and postpartum women		
Toyokawa 2017	Estimation of the number of children with cerebral palsy using nationwide health insurance claims data in Japan	NDB		Nationwide		2012	2013	Clinical epidemiology, course of diseases		Diseases of the nervous system		cerebral palsy	Children	aged below 20 years
Kishi 2020	General medical claims for behavioral health patients in Japan	JMDC		Nationwide		2014	2015	Health economics		Diseases of the nervous system	Others	behavioral health patients: G30-31 (dementia), chronic medical condition	Others	18 years and older
Shigemi 2019	Recent overview of patients with anti-N-methyl-D-aspartate receptor encephalitis using a national inpatient database in Japan	DPC		Nationwide		2010	2017	Medical treatment status	Intervention effect	Diseases of the nervous system		anti-NMDAR encephalitis (ICD-10 code G048 or G049)		age categories
Hatachi 2020	Prognostic factors among children with acute encephalitis/encephalopathy associated with viral and other pathogens	DPC		Nationwide		2010	2018	Clinical epidemiology, course of diseases		Diseases of the nervous system	Certain infectious and parasitic diseases	Acute encephalitis/encephalopathy	Children	aged ≤18 years
Hayakawa 2020	Recent treatment patterns and variations for pediatric acute encephalopathy in Japan	DPC		Nationwide		2010	2016	Medical treatment status	Health policy evaluation and utilization	Diseases of the nervous system		pediatric acute encephalopathy	Children	between 1 month and 15 years of age
Nakamura 2020	Phenytoin versus fosphenytoin for second-line treatment of status epilepticus: propensity score matching analysis using a nationwide inpatient database	DPC		Nationwide		2011	2015	Intervention effect		Diseases of the nervous system		epilepticus (ICD-10 code: G41)	Others	≥16 year of age
Chen 2021	Prescription patterns of antiepileptic drugs for adult patients with newly diagnosed focal epilepsy from 2006 to 2017 in Japan	JMDC		Nationwide		2006	2017	Medical treatment status	Quality of care	Diseases of the nervous system		epilepsy	Others	aged 20-65 years
Nakamura 2020	Levetiracetam vs. Fosphenytoin for Second-Line Treatment of Status Epilepticus: Propensity Score Matching Analysis Using a Nationwide Inpatient Database	DPC		Nationwide		2011	2018	Intervention effect		Diseases of the nervous system		epilepticus	Others	aged ≥15 years
Nakamura 2020	Changes in Real-world Practice Patterns of Antiepileptic Drugs for Status Epilepticus: A Nationwide Observational Study in Japan	DPC		Nationwide		2011	2018	Medical treatment status	Intervention effect	Diseases of the nervous system		epilepticus	Others	aged ≥15 years
Hirano 2018	Risk of Extrapyramidal Syndromes Associated With Psychotropic Polypharmacy: A Study Based on Large-Scale Japanese Claims Data	Others	MinaCare	Nationwide		2011	2016	Intervention effect		Diseases of the nervous system		Extrapyramidal syndromes		
Inokuchi 2014	Effect of rehabilitation on mortality of patients with Guillain-Barre Syndrome: a propensity-matched analysis using nationwide database	DPC		Nationwide		2007	2011	Intervention effect		Diseases of the nervous system		Guillain-Barre Syndrome (G610)		age category

Kobori 2017	Coexisting infectious diseases on admission as a risk factor for mechanical ventilation in patients with Guillain-Barre syndrome	DPC	DPC research group	Nationwide		2010	2012	Clinical epidemiology, course of diseases		Diseases of the nervous system		Guillain-Barre Syndrome		age group
Yonezawa 2020	Effect of Early Tracheostomy on Mortality of Mechanically Ventilated Patients with Guillain-Barre Syndrome: A Nationwide Observational Study	DPC		Nationwide		2010	2018	Intervention effect		Diseases of the nervous system		Guillain–Barré Syndrome (ICD-10 code: G610)	Others	aged 18 years or older
Matsuda 2010	[Epidemiological study of Guillain-Barre syndrome cased on DPC database]	DPC		Nationwide		2008	2008	Research methodology	Medical treatment status	Diseases of the nervous system		Guillain-Barre syndrome		
Mizobuchi 2017	The Current Status of Microvascular Decompression for the Treatment of Hemifacial Spasm in Japan: An Analysis of 2907 Patients Using the Japanese Diagnosis Procedure Combination Database	DPC	DPC Research Institute	Nationwide		2010	2013	Medical treatment status	Intervention effect	Diseases of the nervous system		Hemifacial Spasm (G 513)		
Meyers 2019	Treatment patterns and characteristics of patients with migraine in Japan: A retrospective analysis of health insurance claims data	JMDC		Nationwide		2011	2014	Clinical epidemiology, course of diseases	Medical treatment status	Diseases of the nervous system		migraine	Others	aged ≥18 years
Kawachi 2019	Recent transition of medical cost and relapse rate of multiple sclerosis in Japan based on analysis of a health insurance claims database	MDV		Nationwide		2008	2016	Health economics	Intervention effect	Diseases of the nervous system		multiple sclerosis (G35)		
Ogino 2018	Treatment and comorbidities of multiple sclerosis in an employed population in Japan: analysis of health claims data	JMDC		Nationwide		2005	2014	Clinical epidemiology, course of diseases	Medical treatment status	Diseases of the nervous system		multiple sclerosis (G35)	Others	<65 years old
Ogino 2017	Prevalence, treatments and medical cost of multiple sclerosis in Japan based on analysis of a health insurance claims database	JMDC		Nationwide		2005	2016	Clinical epidemiology, course of diseases	Health economics	Diseases of the nervous system		multiple sclerosis (G35)		
Ogino 2016	Current treatment status and medical cost for multiple sclerosis based on analysis of a Japanese claims database	MDV		Nationwide		2008	2014	Medical treatment status	Health economics	Diseases of the nervous system		multiple sclerosis (G35)		
Mouri 2020	Effect of Sugammadex on Postoperative Myasthenic Crisis in Myasthenia Gravis Patients: Propensity Score Analysis of a Japanese Nationwide Database	DPC		Nationwide		2010	2016	Intervention effect		Diseases of the nervous system		Myasthenia Gravis (ICD-10 code: G70.0)	Others	aged ≥20 years
Murai 2019	Clinical burden and healthcare resource utilization associated with myasthenia gravis: Assessments from a Japanese claims database	MDV		Nationwide		2008	2016	Clinical epidemiology, course of diseases	Patient health service utilization	Diseases of the nervous system		myasthenia gravis (G70.0)	Others	aged ≥18 years
Ogino 2015	[The Analysis of the Unevenness of Treatment for the Nervous Diseases by the Area Judging from Receipt Data]	Others	Health Insurance Claims Review & Reimbursement services, National Health Insurance Organization	Nationwide		2009	2011	Socioeconomic comparison	Health economics	Diseases of the nervous system		Nervous Diseases		
Ogino 2014	[High-cost medical care in intractable neuroimmunological diseases : Is the DPC/PDPS payment system efficient for this population?]	Others	Health Insurance Claims Review & Reimbursement services	Nationwide		2011	2011	Health economics	Medical treatment status	Diseases of the nervous system		intractable neuroimmunological diseases		
Nakamura 2012	Mortality of neuroleptic malignant syndrome induced by typical and atypical antipsychotic drugs: a propensity-matched analysis from the Japanese Diagnosis Procedure Combination database	DPC		Nationwide		2004	2008	Intervention effect		Diseases of the nervous system		neuroleptic malignant syndrome (ICD-10 code: G210)		
Hirakata 2018	Pregabalin Prescription for Neuropathic Pain and Fibromyalgia: A Descriptive Study Using Administrative Database in Japan	MDV		Nationwide		2010	2013	Medical treatment status		Diseases of the nervous system		neuropathic pain and fibromyalgia (pregabalin use)		
Suzuki 2020	Adherence to treatment guideline recommendations for Parkinson's disease in Japan: A longitudinal analysis of a nationwide medical claims database between 2008 and 2016	MDV		Nationwide		2008	2016	Medical treatment status	Quality of care	Diseases of the nervous system		Parkinson's disease	Others	≥30 years of age
Nakaoka 2014	Prescribing pattern of anti-Parkinson drugs in Japan: a trend analysis from 2005 to 2010	JMDC		Nationwide		2005	2010	Medical treatment status	Health policy evaluation and utilization	Diseases of the nervous system		Parkinson's disease (G-20)	Others	younger than 30 years were excluded
Oichi 2017	Mortality and morbidity after spinal surgery in patients with Parkinson's disease: a retrospective matched-pair cohort study	DPC		Nationwide		2010	2013	Clinical epidemiology, course of diseases		Diseases of the nervous system	Others	Parkinson's disease, spinal surgery	Others	aged 20 years or older
Ooba 2011	The impact in Japan of regulatory action on prescribing of dopamine receptor agonists: analysis of a claims database between 2005 and 2008	JMDC		Nationwide		2005	2008	Health policy evaluation and utilization		Diseases of the nervous system		Parkinson's disease	Others	age ≥40 years
Iketani 2020	Risk stratification for physical morbidity using factors associated with atypical antipsychotic treatment in Parkinson's disease: A retrospective observational study using administrative claims data	Others	NHO: MIA databank	Nationwide		2012	2017	Prediction model	Medical treatment status	Diseases of the nervous system		Parkinson's disease	Others	aged over 50 years
Suzuki 2020	Prescription pattern of anti-Parkinson's disease drugs in Japan based on a nationwide medical claims database	MDV		Nationwide		2008	2016	Medical treatment status		Diseases of the nervous system		Parkinson's disease	Others	aged ≥30 years
Iketani 2019	Comparison of the association of risperidone and quetiapine with deteriorating performance in walking and dressing in subjects with Parkinson's disease: a retrospective cohort study using administrative claims data	DPC	NHO: MIA databank	Nationwide		2012	2017	Intervention effect		Diseases of the nervous system		Parkinson's disease		
Iwaki 2019	Comparison of zonisamide with non-levodopa, anti-Parkinson's disease drugs in the incidence of Parkinson's disease-relevant symptoms	MDV		Nationwide		2008	2014	Intervention effect		Diseases of the nervous system		Parkinson's disease	Others	aged ≥40 years
Kasamo 2019	Real-world pharmacological treatment patterns of patients with young-onset Parkinson's disease in Japan: a medical claims database analysis	JMDC		Nationwide		2005	2016	Clinical epidemiology, course of diseases	Medical treatment status	Diseases of the nervous system		young-onset Parkinson's disease	Others	aged 21–50 years
Akazawa 2018	Cost-Minimization Analysis of Deep-Brain Stimulation Using National Database of Japanese Health Insurance Claims	NDB		Nationwide		2009	2015	Health economics		Diseases of the nervous system		Parkinson's disease and other movement disorders		no restriction of age
Nakaoka 2011	Echocardiography for the detection of valvulopathy associated with the use of ergot-derived dopamine agonists in patients with Parkinson's disease	JMDC	JMDC-MDB	Nationwide		2005	2008	Medical treatment status	Quality of care	Diseases of the nervous system	Diseases of the circulatory system	Parkinson's Disease, valvulopathy	Others	excluded younger than 30 years
Sengoku 2019	[Treatment of peripheral facial paralysis in Japan: observation study using a health insurance claims database]	JMDC		Nationwide		2010	2012	Medical treatment status		Diseases of the nervous system		peripheral facial paralysis		
Makito 2020	Incidences and risk factors for post--dural puncture headache after neuraxial anaesthesia: A national inpatient database study in Japan	DPC		Nationwide		2010	2017	Intervention effect	Clinical epidemiology, course of diseases	Diseases of the nervous system	Injury, poisoning and certain other consequences of external causes	post–dural puncture headache after neuraxial anaesthesia	Others	aged ≥20 years
Miyashita 2020	The Association Between Hemoglobin Upswing in the Reference Range and Sleep Apnea Syndrome	JMDC		Nationwide		2013	2016	Clinical epidemiology, course of diseases		Diseases of the nervous system		Sleep apnea syndrome	Others	aged 40－59 years
Inada 2021	Prescribing Pattern of Hypnotic Medications in Patients Initiating Treatment at Japanese Hospitals: A Nationwide, Retrospective, Longitudinal, Observational Study Using a Claims Database	MDV		Nationwide		2012	2016	Medical treatment status	Quality of care	Diseases of the nervous system		insomnia		
Maeda 2016	Quantification of adverse effects of regular use of triazolam on clinical outcomes for older people with insomnia: a retrospective cohort study	NHI/LSEHS	Fukuoka Late Stage Elderly Healthcare Insurance Organization	Prefecture	Fukuoka Late Stage Elderly Healthcare Insurance.	2010	2013	Intervention effect		Diseases of the nervous system		insomnia	Older persons	aged 75 years and older, or those aged 65–74 years

Fujimoto 2014	[The Association between Statin Use and the Risk of Sleep Disturbances: Data Mining of Claims Database]	JMDC		Nationwide		2005	2011	Intervention effect		Diseases of the nervous system	Others	statin use and the risk of sleep disturbances		
Ishikawa 2021	Spinal Cord Infarction after Bronchial Artery Embolization for Hemoptysis: A Nationwide Observational Study in Japan	DPC		Nationwide		2010	2018	Clinical epidemiology, course of diseases	Intervention effect	Diseases of the nervous system	Others	hemoptysis (ICD-10 codes R042, R048, and R049, spinal cord infarction	Others	aged ≥18 years
Kawahara 2013	[[Development of medical care for spinocerebellar degeneration] (Part 5) Characteristic analysis of medical costs required for SCD and SLE]*	MDV		Nationwide		2010	2012	Health economics		Diseases of the nervous system	Diseases of the musculoskeletal system and connective tissue	spinocerebellar degeneration, systemic lupus erythematosus		
Mizobuchi 2018	The Current Status of Microvascular Decompression for the Treatment of Trigeminal Neuralgia in Japan: An Analysis of 1619 Patients Using the Japanese Diagnosis Procedure Combination Database	DPC		Nationwide		2010	2013	Intervention effect	Medical treatment status	Diseases of the nervous system		Trigeminal Neuralgia		
7) Diseases of the eye and adnexa (H00-H59)														
Sakai 2013	DPC Based Situation Analysis of Generic Antibiotics Use among the Japanese Acute Care Hospitals	DPC		Nationwide		2010	2010	Medical treatment status	Health policy evaluation and utilization	Diseases of the eye and adnexa	Others	surgical cataract cases, antibiotics use		
Kido 2020	Nationwide incidence of central retinal artery occlusion in Japan: an exploratory descriptive study using the National Database of Health Insurance Claims (2011-2015)	NDB	Sampling data	Nationwide		2011	2015	Clinical epidemiology, course of diseases		Diseases of the eye and adnexa		central retinal artery occulusion (H34.1)		
Furuta 2016	[Retrospective Analysis of a Health-Insurance Claims Database to Investigate the Prevalence of Dacryocystitis and Dacryostenosis Related Cases and its Correlation with Rebamipide in Ophthalmic Suspensions Administered to Dry-Eye Patients]	JMDC		Nationwide		2014	2015	Intervention effect		Diseases of the eye and adnexa		Dacryocystitis and dacryostenosis related cases		
Fukuda 2021	Medical Costs of and Changes in Glaucoma Treatment among Patients Newly Starting Glaucoma Care	JMDC		Nationwide		2005	2016	Health economics	Medical treatment status	Diseases of the eye and adnexa		glaucoma		
Ooba 2020	Comparison between high and low potency statins in the incidence of open-angle glaucoma: A retrospective cohort study in Japanese working-age population	JMDC		Nationwide		2005	2014	Intervention effect		Diseases of the eye and adnexa		glaucoma (H40 in ICD-10 code)	Others	20- to 74-year age group
Sakamoto 2017	Changes in Glaucoma Medication during the Past Eight Years and Future Directions in Japan Based on an Insurance Medical Claim Database	JMDC		Nationwide		2006	2013	Medical treatment status	Clinical epidemiology, course of diseases	Diseases of the eye and adnexa		Glaucoma		
Kashiwagi 2014	Persistence with topical glaucoma therapy among newly diagnosed Japanese patients	JMDC		Nationwide		2005		Patient health service utilization		Diseases of the eye and adnexa		glaucoma		
Hashimoto 2020	Intraocular pressure-lowering medications during pregnancy and risk of neonatal adverse outcomes: a propensity score analysis using a large database	JMDC		Nationwide		2005	2018	Intervention effect		Diseases of the eye and adnexa	Certain conditions originating in the perinatal period	glaucoma, neonatal adverse outcomes (congenital anomalies, preterm birth, low birth weight)		pregnant women
Kashiwagi 2020	Persistence and treatment patterns of fixed combination drugs for glaucoma: a retrospective claims database study in Japan	JMDC		Nationwide		2005	2016	Medical treatment status	Patient health service utilization	Diseases of the eye and adnexa		glaucoma	Others	aged ≥20 years
Hashimoto 2019	[Clinical Science : Recent Trends in Glaucoma Surgery in Japan : a Descriptive Study Using Large-scale Databases]	Others	NDB open data, DPC	Nationwide		2010	2016	Medical treatment status		Diseases of the eye and adnexa		Glaucoma Surgery		
Shirai 2021	Adherence and Persistence with First-Line Therapy and Compliance with Glaucoma Guidelines Using Japanese Health Care/Pharmacy Claims Database	Others	MinaCare	Nationwide		2011	2016	Patient health service utilization	Quality of care	Diseases of the eye and adnexa		topical glaucoma		
Shirai 2021	Comparison of adherence between fixed and unfixed topical combination glaucoma therapies using Japanese healthcare/pharmacy claims database: a retrospective non-interventional cohort study	Others	MinaCare	Nationwide		2011	2016	Patient health service utilization		Diseases of the eye and adnexa		glaucoma		
Shirai 2021	Risk factors leading to trabeculectomy surgery of glaucoma patient using Japanese nationwide administrative claims data: a retrospective non-interventional cohort study	DPC		Nationwide		2012	2018	Clinical epidemiology, course of diseases		Diseases of the eye and adnexa		glaucoma		age groups
Yamano 2017	[Relationship between Medical History of Patients Administered Mikelan (Carteolol Hydrochloride) Ophthalmic Solution and Occurrence of Asthma-related Adverse Events]	JMDC	Otsuka Pharmaceutical Safety Database	Nationwide		2014	2015	Intervention effect		Diseases of the eye and adnexa	Diseases of the respiratory system	glaucoma, asthma		
Kawano 2019	Current status of late and recurrent intraocular lens dislocation: analysis of real-world data in Japan	MDV		Nationwide		2008	2016	Clinical epidemiology, course of diseases		Diseases of the eye and adnexa		late intraocular lens (IOL) dislocation		
Kume 2016	Treatment Patterns and Health Care Costs for Age-Related Macular Degeneration in Japan: An Analysis of National Insurance Claims Data	JMDC		Nationwide		2005	2013	Medical treatment status	Health economics	Diseases of the eye and adnexa		Age-Related Macular Degeneration	Others	40 years of age or older
Tamiya 2021	Validation study of the claims-based definition for age-related macular degeneration at a single university hospital in Japan	Others	claims' data, electronic medical record	Others	Kyoto University Hospital	2011	2017	Research methodology		Diseases of the eye and adnexa		Age-Related Macular Degeneration	Others	
Kawasaki 2021	Treatment patterns for retinal diseases in patients newly-treated with anti-VEGF agents: A retrospective analysis of claims data from the Japan Medical Data Center database	JMDC		Nationwide		2007	2015	Medical treatment status		Diseases of the eye and adnexa	Endocrine, nutritional and metabolic diseases	retinal disease	Others	21-75 years old
Hashimoto 2020	Recent trends in vitreoretinal surgery: a nationwide database study in Japan, 2010–2017	DPC		Nationwide		2010	2017	Medical treatment status		Diseases of the eye and adnexa		vitreoretinal surgery		
8) Diseases of the ear and mastoid process (H60-H95)														
Imai 2021	Probiotic Prescription Status of Pediatric Patients with Otitis Media Receiving Oral Amoxicillin or Amoxicillin/Clavulanate from April 2016 to March 2017 Using a Japanese Health Insurance Claims Database	JMDC		Nationwide		2016	2017	Medical treatment status	Quality of care	Diseases of the ear and mastoid process		otitis media (H659, H660, H669)	Children	≤15 years of age
Yamanaka 2015	[Influence of the Protein-conjugated Pneumococcal Vaccine (PCV7) and the Newly Developed Antimicrobials on the Incidence of Acute Otitis Media and the Frequency of Myringotomy]	JMDC		Nationwide		2007	2013	Intervention effect	Health policy evaluation and utilization	Diseases of the ear and mastoid process		acute otitis media	Children	aged <15 years
Suzuki 2016	Factors associated with prolonged duration of post-tympanoplasty local treatment in adult chronic otitis media patients: A retrospective observational study using a Japanese inpatient database	DPC		Nationwide		2010	2013	Clinical epidemiology, course of diseases	Intervention effect	Diseases of the ear and mastoid process		chronic otitis media (post-tympanoplasty local treatment)	Others	aged 20 years or older
Sasaki 2018	Influence of pneumococcal conjugate vaccines on acute otitis media in Japan	JMDC		Nationwide		2005	2015	Intervention effect	Health policy evaluation and utilization	Diseases of the ear and mastoid process	Diseases of the respiratory system	acute otitis media, pneumococcal conjugate vaccines	Children	patients <15 years
9) Diseases of the circulatory system (I00-I99)														
Ohnuma 2016	Hospital mortality of patients aged 80 and older after surgical repair for type A acute aortic dissection in Japan	DPC		Nationwide		2011	2013	Clinical epidemiology, course of diseases		Diseases of the circulatory system		type A acute aortic dissection (ICD-10 of I71.0)	Older persons	aged 80 and older
Ohbe 2020	Extracorporeal cardiopulmonary resuscitation for acute aortic dissection during cardiac arrest: A nationwide retrospective observational study	DPC		Nationwide		2010	2018	Intervention effect	Health economics	Diseases of the circulatory system		acute aortic dissection	Others	aged ≥18 years
Yamaguchi 2020	Current status of the management and outcomes of acute aortic dissection in Japan: Analyses of nationwide Japanese Registry of All Cardiac and Vascular Diseases-Diagnostic Procedure Combination data	DPC	JROAD-DPC	Nationwide		2012	2015	Clinical epidemiology, course of diseases	Medical treatment status	Diseases of the circulatory system		acute aortic dissection		
Horiguchi 2010	Impact of drug-eluting stents on treatment option mix for coronary artery disease in Japan	DPC		Nationwide		2004	2007	Medical treatment status		Diseases of the circulatory system		angina pectoris, acute myocardial infarction		
Izumi 2015	[Surveillance on Concomitant Medications and Adherence to Anticoagulants in patients Treated with Oral Anticoagulants]	Others	JMIRI	Nationwide		2014	2014	Medical treatment status	Patient health service utilization	Diseases of the circulatory system		oral anticoagulants		

Yamaguchi 2020	Impact of Endovascular Repair on the Outcomes of Octogenarians with Ruptured Abdominal Aortic Aneurysms: A Nationwide Japanese Study	DPC	JROAD-DPC	Nationwide		2012	2015	Intervention effect	Socioeconomic comparison	Diseases of the circulatory system	ruptured abdominal aortic aneurysm	Others	aged ≥20 years
Yamaguchi 2019	The impact of institutional case volume on the prognosis of ruptured aortic aneurysms: a Japanese nationwide study	DPC	JROAD-DPC	Nationwide		2012	2015	Health policy evaluation and utilization		Diseases of the circulatory system	Ruptured aortic aneurysms	Others	aged ≥20 years
Yamaguchi 2019	Editor's Choice - Endovascular Repair Versus Surgical Repair for Japanese Patients With Ruptured Thoracic and Abdominal Aortic Aneurysms: A Nationwide Study	DPC	JROAD-DPC	Nationwide		2012	2015	Intervention effect		Diseases of the circulatory system	Ruptured Thoracic and Abdominal Aortic Aneurysms	Others	aged ≥20 years
Fukuda 2020	Comparing Retreatments and Expenditures in Flow Diversion Versus Coiling for Unruptured Intracranial Aneurysm Treatment: A Retrospective Cohort Study Using a Real-World National Database	NDB		Nationwide		2015	2018	Intervention effect	Health economics	Diseases of the circulatory system	unruptured intracranial aneurysms		
Uchida 2014	Effects of remifentanil on in-hospital mortality and length of stay following clipping of intracranial aneurysm: a propensity score-matched analysis	DPC	DPC-Research Group	Nationwide		2007	2007	Intervention effect		Diseases of the circulatory system	intracranial aneurysm		
Umegaki 2018	Comparison of In-hospital Outcomes Between Transcatheter and Surgical Aortic Valve Replacement in Patients with Aortic Valve Stenosis: A Retrospective Cohort Study Using Administrative Data	DPC	DPC Research Group	Nationwide		2012	2015	Intervention effect		Diseases of the circulatory system	aortic valve stenosis	Others	aged ≥20 years
Matsuda 2011	[Forefront of aortic stenosis treatment: Current status of aortic stenosis treatment as seen from DPC data]*	DPC		Nationwide		2008	2008	Medical treatment status		Diseases of the circulatory system	aortic valve stenosis		
Yamashita 2017	[Comparison of the Length of Stay in Patients Hospitalized and Initiated with Dabigatran or Warfarin for a Concomitant Non-Valvular Atrial Fibrillation in Real-world Japanese Therapeutic Practice (SHORT-J)]	MDV		Nationwide		2011	2016	Intervention effect	Health economics	Diseases of the circulatory system	non-valvular atrial fibrillation		
Hori 2015	[Disease burden of cardiovascular events in patients with atrial fibrillation: Medical and economic effects of dabigatran and warfarin]*	MDV	RE-LY study, Japanese prevalence studies	Nationwide		2014	2014	Health economics		Diseases of the circulatory system	atrial fibrillation		
Okumura 2014	[Estimation of event cost reduction from the administration of apixaban in patients with non-valvular atrial fibrillation]*	MDV	ARISTOTLE study	Nationwide		2012	2013	Health economics		Diseases of the circulatory system	non-valvular atrial fibrillation		
Ohshima 2019	Oral anticoagulants usage in Japanese patients aged 18-74 years with non-valvular atrial fibrillation: a retrospective analysis based on insurance claims data	JMDC		Nationwide		2005	2014	Medical treatment status	Intervention effect	Diseases of the circulatory system	non-valvular atrial fibrillation patients (ICD-10; I48-)	Others	aged 18-74 years
Momo 2020	Predictive factors associated with bleeding in atrial fibrillation patients treated with anti-coagulant drugs using a large claims database	JMDC		Nationwide		2004	2016	Clinical epidemiology, course of diseases		Diseases of the circulatory system	atrial fibrillation, anti-coagulants		
Momo 2019	Prevalence of drug-drug interaction in atrial fibrillation patients based on a large claims data	JMDC		Nationwide		2005	2016	Medical treatment status	Intervention effect	Diseases of the circulatory system	drug-drug interaction (DDI) and bleeding rate in atrial fibrillation		
Kubota 2018	The use of anticoagulants in patients with non-valvular atrial fibrillation between 2005 and 2014: A drug utilization study using claims data in Japan	JMDC		Nationwide		2005	2014	Medical treatment status	Quality of care	Diseases of the circulatory system	Atrial fibrillation (I48)	Others	aged 20-74 years
Yokoyama 2018	Bleeding Risk of Warfarin and Direct Oral Anticoagulants in Younger Population: A Historical Cohort Study Using a Japanese Claims Database	JMDC		Nationwide		2012	2015	Intervention effect		Diseases of the circulatory system	Atrial fibrillation (AF)	Others	18 years or older
Kohsaka 2017	Bleeding risk of apixaban, dabigatran, and low-dose rivaroxaban compared with warfarin in Japanese patients with non-valvular atrial fibrillation: a propensity matched analysis of administrative claims data	MDV		Nationwide		2011	2016	Intervention effect		Diseases of the circulatory system	non-valvular atrial fibrillation (I48)	Others	aged ≥18 years
Kohsaka 2018	Safety and effectiveness of apixaban in comparison to warfarin in patients with nonvalvular atrial fibrillation: a propensity-matched analysis from Japanese administrative claims data	MDV		Nationwide		2011	2017	Intervention effect		Diseases of the circulatory system	atrial fibrillation (I48)	Others	aged ≥18 years
Maeda 2021	Residual risks of ischaemic stroke and systemic embolism among atrial fibrillation patients with anticoagulation: large-scale real-world data (F-CREATE project)	JMDC		Others	Fukuoka Comprehensive Risk Evaluation of Atrial Fibrillation with Claim Database project (F-CREATE project)	2005	2017	Clinical epidemiology, course of diseases		Diseases of the circulatory system	atrial fibrillation (ICD-10: I48)	Others	20-74 years of age
Narita 2020	Trends in Prevalence of Non-Valvular Atrial Fibrillation and Anticoagulation Therapy in a Japanese Region: Analysis Using the National Health Insurance Database	NHI/LSEHS	Japanese National Health Insurance database ("Kokuho" database [KDB])	Municipality	Tsugaru region (5 municip	2014	2017	Clinical epidemiology, course of diseases	Medical treatment status	Diseases of the circulatory system	non-valvular atrial fibrillation	Others	aged 40–74 years
Yasaka 2020	Incidence Rates of Bleeding and Emergency Surgery Due to Trauma or Fracture Among Japanese Patients with Non-valvular Atrial Fibrillation Receiving Oral Anticoagulation Therapy	MDV		Nationwide		2011	2016	Clinical epidemiology, course of diseases		Diseases of the circulatory system	nonvalvular atrial fibrillation (I48)	Others	age >18 years
Suzuki 2019	Potential of machine learning methods to identify patients with nonvalvular atrial fibrillation	MDV		Nationwide		2008	2017	Prediction model	Medical treatment status	Diseases of the circulatory system	nonvalvular atrial fibrillation		
Koretsune 2017	Usefulness of a healthcare database for epidemiological research in atrial fibrillation	MDV	comparable with J-RHYTHM Registry and Fushimi AF Registry	Nationwide		2008	2013	Research methodology	Clinical epidemiology, course of diseases	Diseases of the circulatory system	non-valvular atrial fibrillation		
Yagi 2021	Current status of oral anticoagulant adherence in Japanese patients with atrial fibrillation: A claims database analysis	JMDC		Nationwide		2010	2019	Patient health service utilization		Diseases of the circulatory system	atrial fibrillation		
Kario 2020	Impact of pre-existing hypertension and control status before atrial fibrillation onset on cardiovascular prognosis in patients with non-valvular atrial fibrillation: A real-world database analysis in Japan	JMDC		Nationwide		2005	2016	Clinical epidemiology, course of diseases		Diseases of the circulatory system	atrial fibrillation	Others	aged 0-75 years
Kohsaka 2020	Safety and effectiveness of non-vitamin K oral anticoagulants versus warfarin in real-world patients with non-valvular atrial fibrillation: a retrospective analysis of contemporary Japanese administrative claims data	MDV		Nationwide		2011	2018	Intervention effect		Diseases of the circulatory system	non-valvular atrial fibrillation	Others	aged ≥18 years
Maeda 2020	Risks of Bleeding and Stroke Based on CHA2DS2-VASc Scores in Japanese Patients With Atrial Fibrillation: A Large-Scale Observational Study Using Real-World Data	JMDC		Nationwide		2005	2017	Clinical epidemiology, course of diseases		Diseases of the circulatory system	atrial fibrillation or atrial flatter	Others	aged 21-74 years

Koretsune 2019	Comparative effectiveness and safety of warfarin and dabigatran in patients with non-valvular atrial fibrillation in Japan: A claims database analysis	MDV		Nationwide		2011	2016	Intervention effect	Medical treatment status	Diseases of the circulatory system		non-valvular atrial fibrillation	Others	aged ≥18 years
Satoh 2020	Blood Pressure and Chronic Kidney Disease Stratified by Gender and the Use of Antihypertensive Drugs	JMDC		Nationwide		2008	2017	Clinical epidemiology, course of diseases		Diseases of the circulatory system	Diseases of the genitourinary system	blood pressure and the risk of chronic kidney disease	Others	30-74 years of age
Kawamura 2019	Patient characteristics and in-hospital complications of subcutaneous implantable cardioverter-defibrillator for Brugada syndrome in Japan	DPC		Nationwide		2016	2017	Intervention effect		Diseases of the circulatory system		Brugada syndrome (I490)		
Kido 2020	Outcomes of paediatric out-of-hospital cardiac arrest according to hospital characteristic defined by the annual number of paediatric patients with invasive mechanical ventilation: A nationwide study in Japan	DPC		Nationwide		2010	2017	Quality of care	Health policy evaluation and utilization	Diseases of the circulatory system		paediatric out-of-hospital cardiac arrest	Children	children (<18 years)
Zamami 2017	[Drug Repositioning Research Utilizing a Large-scale Medical Claims Database to Improve Survival Rates after Cardiopulmonary Arrest]	JMDC		Nationwide		2005	2014	Medical treatment status	Intervention effect	Diseases of the circulatory system		Cardiopulmonary Arrest	Others	aged 18 years or older
Fukuda 2013	Health care costs related to out-of-hospital cardiopulmonary arrest in Japan	DPC		Nationwide		2008	2009	Health economics		Diseases of the circulatory system		cardiopulmonary arrest (OHCA)	Others	age ≥18 years
Takiguchi 2021	Intermittent versus continuous neuromuscular blockade during target temperature management after cardiac arrest: A nationwide observational study	DPC		Nationwide		2010	2018	Intervention effect		Diseases of the circulatory system		postcardiac arrest patients	Others	aged ≥18 years
Zamami 2019	Search for Therapeutic Agents for Cardiac Arrest Using a Drug Discovery Tool and Large-Scale Medical Information Database	JMDC		Nationwide		2005	2014	Intervention effect		Diseases of the circulatory system		cardiac arrest patients	Others	excluded <18 years old
Joo 2019	Effectiveness and safety of early enteral nutrition for patients who received targeted temperature management after out-of-hospital cardiac arrest	MDV		Nationwide		2008	2017	Intervention effect		Diseases of the circulatory system		out-of-hospital cardiac arrest	Others	≥20 years of age
Niimura 2017	Hydrocortisone administration was associated with improved survival in Japanese patients with cardiac arrest	JMDC		Nationwide		2005	2014	Intervention effect		Diseases of the circulatory system		Cardiac arrest		
Tagami 2016	Early antibiotics administration during targeted temperature management after out-of-hospital cardiac arrest: a nationwide database study	DPC		Nationwide		2007	2013	Intervention effect		Diseases of the circulatory system		cardiac arrest	Others	age ≥18 years
Tagami 2016	Changes in Therapeutic Hypothermia and Coronary Intervention Provision and In-Hospital Mortality of Patients With Out-of-Hospital Cardiac Arrest: A Nationwide Database Study	DPC		Nationwide		2008	2012	Medical treatment status	Intervention effect	Diseases of the circulatory system		Out-of-Hospital Cardiac Arrest	Others	aged ≥18 years
Tagami 2016	Amiodarone or nifekalant upon hospital arrival for refractory ventricular fibrillation after out-of-hospital cardiac arrest	DPC		Nationwide		2007	2013	Intervention effect		Diseases of the circulatory system		Out-of-Hospital Cardiac Arrest	Others	aged ≥18 years
Tagami 2016	Amiodarone Compared with Lidocaine for Out-Of-Hospital Cardiac Arrest with Refractory Ventricular Fibrillation on Hospital Arrival: a Nationwide Database Study	DPC		Nationwide		2007	2013	Intervention effect		Diseases of the circulatory system		out-of-hospital cardiac arrest and refractory ventricular fibrillation	Others	aged ≥18 years
Shakya 2017	In-hospital complications after implantation of cardiac implantable electronic devices: Analysis of a national inpatient database in Japan	DPC		Nationwide		2010	2014	Intervention effect		Diseases of the circulatory system		complications after implantation of cardiac implantable electronic devices		age categories
Aso 2016	The Effect of Intraaortic Balloon Pumping Under Venoarterial Extracorporeal Membrane Oxygenation on Mortality of Cardiogenic Patients: An Analysis Using a Nationwide Inpatient Database	DPC		Nationwide		2010	2013	Intervention effect		Diseases of the circulatory system		cardiogenic shock (ICD-10 codes: I05, I07, I08, I20–I22, I33–I35, I40–I42, I46, and I49–I51)		
Ohbe 2018	Early enteral nutrition for cardiogenic or obstructive shock requiring venoarterial extracorporeal membrane oxygenation: a nationwide inpatient database study	DPC		Nationwide		2010	2016	Intervention effect		Diseases of the circulatory system		cardiogenic or obstructive shock	Others	aged ≥18 years
Higa 2021	Relationship of Annual Health Checkups with the Subject's Subsequent Behavior of Cardiovascular Risk Management in a Real-World Setting in Japan: A Retrospective Cohort Study on Changes in Antihypertensive Drug Prescription and Blood Pressure from 2015 to 2017	Others	MinaCare database	Nationwide		2014	2017	Patient health service utilization		Diseases of the circulatory system		Cardiovascular Risk	Others	20 and 74 years
Okuno 2021	Intra-operative autologous blood donation for cardiovascular surgeries in Japan: A retrospective cohort study	DPC		Nationwide		2016	2019	Intervention effect		Diseases of the circulatory system		cardiovascular surgeries	Others	aged 18–84 years
Kanaoka 2021	Current Status and Effect of Outpatient Cardiac Rehabilitation After Percutaneous Coronary Intervention in Japan	NDB		Nationwide		2014	2018	Intervention effect		Diseases of the circulatory system		cardiovascular diseases	Others	aged ≥20 years
Kaneko 2021	Possible association between eating behaviors and cardiovascular disease in the general population: Analysis of a nationwide epidemiological database	JMDC		Nationwide		2005	2018	Clinical epidemiology, course of diseases		Diseases of the circulatory system		cardiovascular disease	Others	aged ≥20 years
Kaneko 2021	Association of Blood Pressure Classification Using the 2017 American College of Cardiology/American Heart Association Blood Pressure Guideline with Risk of Heart Failure and Atrial Fibrillation	JMDC		Nationwide		2005	2018	Clinical epidemiology, course of diseases		Diseases of the circulatory system		Heart failure, atrial fibrillation, hypertension	Others	aged ≥20 years
Shima 2021	Validation of novel identification algorithms for major adverse cardiovascular events in a Japanese claims database	Others	claims database of Jichi Medical University Hospital, electrical medical records	Others	Jichi Medical University Hospital	2012	2014	Research methodology		Diseases of the circulatory system		adverse cardiovascular events	Others	aged at least 20 years
Takura 2021	Development of a predictive model for integrated medical and long-term care resource consumption based on health behaviour: application of healthcare big data of patients with circulatory diseases	NHI/LSEHS	national healthcare database (Kokuho Database, KDB)	Others	(The regions included in the database account for 6.1% of the total population of Japan)	2014	2018	Prediction model	Health economics	Diseases of the circulatory system		circulatory disease (ICD-10 I 00-99)		all ages
Kaneko 2020	Restfulness from sleep and subsequent cardiovascular disease in the general population	JMDC		Nationwide		2005	2018	Clinical epidemiology, course of diseases		Diseases of the circulatory system		cardiovascular disease	Others	aged ≥20 years
Maeda 2017	Safety of Tranexamic Acid in Pediatric Cardiac Surgery: A Nationwide Database Study	DPC		Nationwide		2010	2014	Intervention effect		Diseases of the circulatory system		pediatric cardiac surgery population	Children	aged ≤12 years
Davis 2015	High-Risk Atherosclerotic Cardiovascular Disease in a Real-World Employed Japanese Population: Prevalence, Cardiovascular Event Rates, and Costs	JMDC		Nationwide		2008	2009	Clinical epidemiology, course of diseases	Health economics	Diseases of the circulatory system		atherosclerotic cardiovascular disease	Others	aged ≥18 years
Kaneko 2021	Fasting plasma glucose and subsequent cardiovascular disease among young adults: Analysis of a nationwide epidemiological database	JMDC		Nationwide		2005	2018	Clinical epidemiology, course of diseases		Diseases of the circulatory system		cardiovascular disease	Others	aged 20–49 years
Kitamura 2021	Safety and effectiveness of intracardiac echocardiography in ventricular tachycardia ablation: a nationwide observational study	DPC		Nationwide		2011	2017	Intervention effect		Diseases of the circulatory system		ventricular tachycardia ablation	Others	aged ≥18 years
Kaneko 2020	Cardiovascular Health Metrics of 87,160 Couples: Analysis of a Nationwide Epidemiological Database	JMDC		Nationwide		2005	2016	Clinical epidemiology, course of diseases		Diseases of the circulatory system		cardiovascular health metrics	Others	aged ≥20 years

Ugata 2020	Periprocedural hypotension after conscious sedation versus local anesthesia during defibrillator implantation for left ventricular dysfunction: analysis of a national inpatient database in Japan	DPC		Nationwide		2010	2016	Intervention effect		Diseases of the circulatory system		Implantable cardioverter defibrillators and cardiac resynchronization therapy devices	Others	aged ≥18 years
Watanabe 2020	Impact of Board-Certified Cardiologist Characteristics on Risk of In-Hospital Mortality	DPC	JROAD-DPC	Nationwide		2012	2014	Health policy evaluation and utilization		Diseases of the circulatory system		cardiovascular disease	Others	aged ≥18 years
Gohbara 2018	Low Activities of Daily Living Associated With Increased Cardiovascular Disease Mortality in Japan - Analysis of Health Records From a Nationwide Claim-Based Database, JROAD-DPC	DPC	JROAD-DPC	Nationwide		2012	2014	Clinical epidemiology, course of diseases		Diseases of the circulatory system		acute coronary syndrome, heart failure, or aortic aneurysm/dissection	Others	aged ≥20 years
Isogai 2016	Relationship between hospital volume and major cardiac complications of rotational atherectomy: A nationwide retrospective cohort study in Japan	DPC		Nationwide		2010	2013	Health policy evaluation and utilization		Diseases of the circulatory system		cardiac complications of rotational atherectomy	Others	aged ≥20 years
Isogai 2015	Serious cardiac complications in coronary spasm provocation tests using acetylcholine or ergonovine: analysis of 21 512 patients from the diagnosis procedure combination database in Japan	DPC		Nationwide		2010	2013	Intervention effect		Diseases of the circulatory system		Cardiac complications in coronary spasm provocation tests using acetylcholine or ergonovine	Others	aged ≥20 years
Kitazawa 2019	Risk of coronary artery disease according to glucose abnormality status and prior coronary artery disease in Japanese men	JMDC		Nationwide		2008	2016	Clinical epidemiology, course of diseases		Diseases of the circulatory system	Endocrine, nutritional and metabolic diseases	glycemic level, cardiovascular disease	Others	18-72 years old
Fujihara 2017	Impact of body mass index and metabolic phenotypes on coronary artery disease according to glucose tolerance status	JMDC		Nationwide		2008	2015	Clinical epidemiology, course of diseases		Diseases of the circulatory system	Endocrine, nutritional and metabolic diseases	coronary artery disease, glucose tolerance status	Others	18-72 years old
Hashikata 2011	Usefulness of a large automated health records database in pharmacoepidemiology	MDV	previous epidemiological survey (Hisayama survey, JIKEI-Heart study, KYOTO-Heart study)	Nationwide	15 medical facilities	2006	2009	Research methodology		Diseases of the circulatory system	Endocrine, nutritional and metabolic diseases	cardio-cerebrovascular events, diabetes, dialysis initiation		
Teramoto 2016	Low-density lipoprotein cholesterol levels and lipid-modifying therapy prescription patterns in the real world: An analysis of more than 33,000 high cardiovascular risk patients in Japan	MDV		Nationwide		2013	2013	Medical treatment status	Quality of care	Diseases of the circulatory system	Endocrine, nutritional and metabolic diseases	acute coronary syndrome , other coronary heart disease, ischemic stroke, peripheral arterial disease or diabetes mellitus	Others	aged ≥20 years
Sakamoto 2020	Clinical features and outcomes of nonocclusive mesenteric ischemia after cardiac surgery: a retrospective cohort study	DPC		Nationwide		2010	2017	Clinical epidemiology, course of diseases		Diseases of the circulatory system	Diseases of the digestive system	Nonocclusive mesenteric ischemia after cardiac surgery		
Kaneko 2020	Lipid Profile and Subsequent Cardiovascular Disease among Young Adults Aged < 50 Years	JMDC		Nationwide		2005	2018	Clinical epidemiology, course of diseases		Diseases of the circulatory system	Endocrine, nutritional and metabolic diseases	lipid profile and cardiovascular disease (CVD) events	Others	20-49 years of age
Kajinami 2019	Real-World Data to Identify Hypercholesterolemia Patients on Suboptimal Statin Therapy	MDV		Nationwide		2008	2017	Medical treatment status		Diseases of the circulatory system	Endocrine, nutritional and metabolic diseases	dyslipidemia patients with atherosclerotic cardiovascular disease	Others	over 20 years
Seposo 2020	Short-term effects of air pollution on daily single- and co-morbidity cardiorespiratory outpatient visits	JMDC		Nationwide		2013	2016	Clinical epidemiology, course of diseases		Diseases of the circulatory system	Diseases of the respiratory system	Cardiovascular-related (I00-I99) and respiratory-related (J00-J99) outpatient clinic visit visits		all ages
Sasabuchi 2020	Clinical Impact of Prescribed Doses of Nutrients for Patients Exclusively Receiving Parenteral Nutrition in Japanese Hospitals: A Retrospective Cohort Study	MDV		Nationwide		2009	2018	Intervention effect	Quality of care	Diseases of the circulatory system		hospitalized patients who underwent central venous catheter (CVC) insertion	Others	aged ≥18 years
Toba 2016	[Selection of a method for detecting cases of severe pneumothorax associated with central venous catheterization using information on fee-for-medical-service : Potential utilizability of information from Fee-for-medical-service for internal audit]	DPC	medical records	Others	Medical Hospital, Tokyo Medical and Dental University	2012	2015	Research methodology	Health policy evaluation and utilization	Diseases of the circulatory system		severe pneumothorax associated with central venous catheterization		
Matsuda 2018	[Big data analysis of medical and nursing care: An analysis of the current state of rehabilitation in patients with ischemic stroke using consolidated data on medical and nursing care]*	NHI/LSEHS	medical insurance claims and LTC insurance claims data	Others	A municipality in Western Japan	2011	2015	Medical treatment status		Diseases of the circulatory system		brain infarction		
Matsuda 2018	[Big data analysis of medical and nursing care: An analysis of the usage of medical and nursing care services of stroke patients using consolidated data on health insurance claims in medical and nursing care]*	NHI/LSEHS	medical insurance claims and LTC insurance claims data	Others	A municipality in Western Japan	2012	2016	Medical treatment status	Health policy evaluation and utilization	Diseases of the circulatory system		brain infarction		
Matsuda 2010	[DPC based situation analysis of acute rehabilitation care for stroke patients in Japan]	DPC	DPC Research Group	Nationwide		2008	2008	Medical treatment status	Intervention effect	Diseases of the circulatory system		brain infarction		
Matsuda 2015	Analysis of Disease Structure for the Regional Health Care Plan Based on the National Database	NDB		Prefecture	Fukuoka	2010	2010	Health policy evaluation and utilization		Diseases of the circulatory system		cerebral infarction		
Kato 2019	[[Introduction to health economics for healthcare professionals] Information asymmetry between doctors and patients]*	DPC		Nationwide		2010	2016	Quality of care	Socioeconomic comparison	Diseases of the circulatory system		cerebral infarction		
Fujino 2014	Impact of regional clinical pathways on the length of stay in hospital among stroke patients in Japan	DPC		Nationwide		2011	2012	Health policy evaluation and utilization		Diseases of the circulatory system		cerebral infarction		
Nagayama 2021	Medical Costs and Readmissions After Intensive Poststroke Rehabilitation: Japanese Claims Data	JMDC		Nationwide		2005	2017	Intervention effect	Health economics	Diseases of the circulatory system		cerebrovascular disorder (ICD-10: I60-I69)		
Hatanaka 2016	[Impact of drinking and smoking habits on cerebrovascular disease risk among male employees]	Others	DENSO Health Insurance Society	Others	DENSO Health Insurance	2004	2013	Clinical epidemiology, course of diseases		Diseases of the circulatory system		cerebrovascular disease	Others	aged 20-46 years
Sayama 2015	Visualization of medical care for cerebrovascular disorders	DPC	J-ASPECT Study	Nationwide		2012	2013	Intervention effect	Research methodology	Diseases of the circulatory system		Cerebrovascular Disorders		
Tanaka 2020	Proportion and risk factors of cholesterol crystal embolization after cardiovascular procedures: a retrospective national database study	DPC		Nationwide		2010	2017	Clinical epidemiology, course of diseases	Intervention effect	Diseases of the circulatory system		Cholesterol crystal embolization (I749)	Others	aged ≥40 years
Isogai 2015	Clinical Practice Patterns in Constrictive Pericarditis Patients With Heart Failure: A Retrospective Cohort Study Using a National Inpatient Database in Japan	DPC		Nationwide		2007	2013	Clinical epidemiology, course of diseases	Medical treatment status	Diseases of the circulatory system		Constrictive pericarditis (ICD-10 code I31.1)		
Ohbe 2021	Effect of Early Rehabilitation on Physical Function in Patients Undergoing Coronary Artery Bypass Grafting: A Nationwide Inpatient Database Study	DPC		Nationwide		2010	2018	Intervention effect		Diseases of the circulatory system		patients who underwent a coronary artery bypass grafting	Others	excluded patients aged <18 years
Kawamoto 2013	[The Adherence with Critical Pathways for Angina Pectoris Undergoing Percutaneous Coronary Intervention by Utilizing the Diagnosis Procedure Combination Database]	DPC	PCI critical pathways data	Others	10 hospitals (NHO)	2011	2011	Medical treatment status	Quality of care	Diseases of the circulatory system		Percutaneous coronary intervention for angina pectoris		

Shimada 2020	Guideline-Based Medications for Older Adults Discharged after Percutaneous Coronary Intervention in a Suburban City of Japan: A Cohort Study Using Claims Data	NHI/LSEHS	The National Health Insurance system and Late Elders' Health Insurance	Municipality	Kashiwa City	2012	2015	Quality of care		Diseases of the circulatory system		after discharge following percutaneous coronary intervention	Older persons	Older Adults
Nakajima 2020	Prevalence of myocardial injury requiring percutaneous coronary intervention after acute carbon monoxide poisoning	DPC		Nationwide		2010	2017	Clinical epidemiology, course of diseases		Diseases of the circulatory system	Injury, poisoning and certain other consequences of external causes	myocardial injury, acute carbon monoxide poisoning (T58)		
Hagiwara 2021	A comparison of the safety and effectiveness of prasugrel and clopidogrel in younger population undergoing percutaneous coronary intervention: A retrospective study using a Japanese claims database	JMDC		Nationwide		2014	2018	Intervention effect		Diseases of the circulatory system		patients undergoing percutaneous coronary intervention		
Maeda 2021	Changes in percutaneous coronary intervention practice in Japan during the COVID-19 outbreak: LIFE Study	NHI/LSEHS	National Health Insurance and Later-Stage Elderly Healthcare System	Municipality	Kobe City	2018	2020	COVID-19	Medical treatment status	Diseases of the circulatory system		percutaneous coronary intervention		age categories
Seki 2019	Follow-up tests and outcomes for patients undergoing percutaneous coronary intervention: analysis of a Japanese administrative database	MDV		Nationwide		2010	2013	Intervention effect		Diseases of the circulatory system		patients undergoing percutaneous coronary intervention	Others	aged ≥18 years
Ono 2018	Utilization of Anticoagulant and Antiplatelet Agents Among Patients With Atrial Fibrillation Undergoing Percutaneous Coronary Intervention - Retrospective Cohort Study Using a Nationwide Claims Database in Japan	NDB		Nationwide		2014	2015	Medical treatment status		Diseases of the circulatory system		Atrial Fibrillation Undergoing Percutaneous Coronary Intervention		
Ochibe 2020	Study of the Effect of the Perioperative Administration of Statin on the Prognosis of Percutaneous Coronary Intervention Using the National Health Insurance Claims Database in Japan	NDB		Nationwide		2015	2016	Intervention effect		Diseases of the circulatory system	Others	statin administration during PCI and cardiovascular events		
Pietzsch 2018	Cost-effectiveness of orbital atherectomy compared to rotational atherectomy in treating patients with severely calcified coronary artery lesions in Japan	MDV	Clinical data: published literature	Nationwide		2014	2016	Health economics		Diseases of the circulatory system		severely calcified coronary artery lesions		
Kanazawa 2017	Underuse of Cardiac Rehabilitation in Workers With Coronary Artery Disease - Claims Database Survey in Japan	JMDC		Nationwide		2006	2013	Patient health service utilization		Diseases of the circulatory system		coronary artery disease	Others	aged ≥18 years
Morishita 2021	Trends, Treatment Approaches, and In-Hospital Mortality for Acute Coronary Syndrome in Japan During the Coronavirus Disease 2019 Pandemic	DPC	QIP	Nationwide		2018	2020	COVID-19	Medical treatment status	Diseases of the circulatory system		acute coronary syndrome	Others	aged 18 years or older
Tanaka 2019	Treatment strategy modification and its implication on the medical cost of fractional flow reserve-guided percutaneous coronary intervention in Japan	MDV	CVIT-DEFER Registry study	Nationwide		2008		Health economics	Health policy evaluation and utilization	Diseases of the circulatory system		stable coronary artery disease		
Yamada 2015	Benchmark Analysis and Comparative Study of the Quality Indicators of ACS/AMI with the DPC Data	DPC	MDV: EVE	Others	JADECOM: 13 hospitals	2012	2012	Quality of care		Diseases of the circulatory system		acute coronary syndrome, acute myocardial infarction		
Isogai 2015	Hospital volume and cardiac complications of endomyocardial biopsy: a retrospective cohort study of 9508 adult patients using a nationwide inpatient database in Japan	DPC		Nationwide		2010	2013	Health policy evaluation and utilization		Diseases of the circulatory system		complications with endomyocardial biopsy	Others	aged ≥20 years
Ooba 2020	Frequency of Clinical Monitoring of Serum Concentrations of Digoxin, Potassium, and Creatinine, and Recording of Electrocardiograms in Digoxin-Treated Patients: A Japanese Claims Database Analysis	JMDC		Nationwide		2005	2014	Medical treatment status	Quality of care	Diseases of the circulatory system		digoxin prescription	Others	aged 20–74 years
Kitamura 2020	Patient characteristics, procedure details including catheter devices, and complications of catheter ablation for ventricular tachycardia: a nationwide observational study	DPC		Nationwide		2010	2017	Clinical epidemiology, course of diseases	Medical treatment status	Diseases of the circulatory system		structural heart disease	Others	aged ≥18 years
Mizuno 2020	Impact of facilities accredited by both adult and pediatric cardiology societies on the outcome of patients with adult congenital heart disease	DPC	JROAD-DPC	Nationwide	JCS-certified teaching hospitals	2013	2014	Health policy evaluation and utilization		Diseases of the circulatory system		adult congenital heart disease	Others	aged ≥15 years
Isogai 2018	Clinical features and peripartum outcomes in pregnant women with cardiac disease: a nationwide retrospective cohort study in Japan	DPC		Nationwide		2008	2014	Medical treatment status	Clinical epidemiology, course of diseases	Diseases of the circulatory system	Pregnancy, childbirth and the puerperium	cardiac disease (pregnant women)		
Wakabayashi 2019	Impact of Body Mass Index on Activities of Daily Living in Inpatients with Acute Heart Failure	JMDC		Nationwide		2014	2017	Clinical epidemiology, course of diseases		Diseases of the circulatory system	Endocrine, nutritional and metabolic diseases	acute heart failur, overweight and obese	Others	aged 20 year or older
Tamaki 2019	Characteristics of heart failure patients incurring high medical costs via matching specific health examination results and medical claim data: a cross-sectional study	NHI/LSEHS	NHI	Municipality	Hiroshima City	2016	2017	Health economics	Clinical epidemiology, course of diseases	Diseases of the circulatory system		heart failure	Others	40 to 74 years
Sasaki 2017	Differences between determinants of in-hospital mortality and hospitalisation costs for patients with acute heart failure: a nationwide observational study from Japan	DPC	QIP	Nationwide		2010	2011	Health economics		Diseases of the circulatory system		heart failure (I50.x)	Others	aged 20 years or older
Nakao 2015	[Actual use of a vasopressin V2 receptor antagonist in hospitalized heart failure patients : using a Japanese hospital database]	MDV		Nationwide		2011	2013	Medical treatment status		Diseases of the circulatory system		heart failure	Others	aged 20 years or older
Matsuda 2019	[Big data analysis of medical and nursing care: An analysis of the usage of medical and nursing care services of elderly heart failure patients using consolidated data on health insurance claims in medical and nursing care]*	NHI/LSEHS	medical insurance claims and LTC insurance claims data	Others	A municipality in Western Japan	2012	2016	Clinical epidemiology, course of diseases	Health policy evaluation and utilization	Diseases of the circulatory system		heart failure	Older persons	aged 65 years or older
Yagi 2018	Association Between Early Rehabilitation and Mobility Status in Elderly Inpatients with Heart Failure: A Nationwide Retrospective Cohort Study	DPC		Nationwide		2010	2014	Intervention effect		Diseases of the circulatory system		heart failure	Others	excluded aged less than 60 years
Matsuda 2018	[Big data analysis of medical and nursing care: An analysis of factors related to medical and long-term care benefit costs for elderly people requiring long-term care with chronic heart failure using the comprehensive medical and long-term care database]*	NHI/LSEHS	medical insurance claims and LTC insurance claims data	Others	A municipality in Western Japan	2011	2016	Health economics		Diseases of the circulatory system		chronic heart failure	Older persons	aged 65 years or older
Matsuda 2018	[Big data analysis of medical and nursing care: An analysis of factors related to the deaths of elderly people requiring long-term care with chronic heart failure using the comprehensive medical and long-term care database]*	NHI/LSEHS	medical insurance claims and LTC insurance claims data	Others	A municipality in Western Japan	2008	2016	Clinical epidemiology, course of diseases		Diseases of the circulatory system		chronic heart failure	Older persons	aged 65 years or older
Abe 2021	Actual state of "triple therapy" for heart failure patients in eight regions of Japan: An analysis of a nationwide medical claims database	MDV		Nationwide		2017	2018	Medical treatment status	Socioeconomic comparison	Diseases of the circulatory system		heart failure	Others	≥15 years of age
Kusunose 2021	Association between Vitamin D and Heart Failure Mortality in 10,974 Hospitalized Individuals	DPC	JROAD-DPC	Nationwide		2012	2017	Intervention effect		Diseases of the circulatory system		heart failure	Others	≥20 years of age
Sasaki 2014	The relationship between the number of cardiologists and clinical practice patterns in acute heart failure: a cross-sectional observational study	DPC	ATTEND registry, JCARE-CARD registry	Nationwide		2010	2011	Quality of care	Research methodology	Diseases of the circulatory system		acute heart failure	Others	older than 20 years of age

Kaneko 2021	Impact of hospital volume on clinical outcomes of hospitalized heart failure patients: analysis of a nationwide database including 447,818 patients with heart failure	DPC	DPC Study Group	Nationwide		2010	2018	Health policy evaluation and utilization		Diseases of the circulatory system	heart failure (ICD-10 codes I50.0, I50.1, and I50.9)	Others	aged ≥20 years
Kuragaichi 2021	Temporal trends of a vasopressin V2 receptor antagonist in heart failure using a nationwide database in Japan	MDV		Nationwide		2008	2018	Medical treatment status		Diseases of the circulatory system	heart failure (I50,I11.0, I13.0, and I13.2)		
Itoh 2020	Reverse J-shaped relationship between body mass index and in-hospital mortality of patients hospitalized for heart failure in Japan	DPC		Nationwide		2010	2018	Clinical epidemiology, course of diseases		Diseases of the circulatory system	hospitalized heart failure patients	Others	aged ≥20 years
Kaku 2020	Impact of Hospital Practice Factors on Mortality in Patients Hospitalized for Heart Failure in Japan - An Analysis of a Large Number of Health Records From a Nationwide Claims-Based Database, the JROAD-DPC	DPC	JROAD-DPC	Nationwide	(hospitals agreed to participate in the JROAD-DPC database study)	2012	2014	Health policy evaluation and utilization		Diseases of the circulatory system	hospitalized heart failure patients		
Kaneko 2020	Characteristics and Outcomes of Super-Elderly Patients (Aged >=90 Years) Hospitalized for Heart Failure - Analysis of a Nationwide Inpatient Database	DPC		Nationwide		2010	2018	Clinical epidemiology, course of diseases		Diseases of the circulatory system	super-elderly heart failure	Others	aged ≥20 years
Nagai 2019	Effect of intravenous carperitide versus nitrates as first-line vasodilators on in-hospital outcomes in hospitalized patients with acute heart failure: Insight from a nationwide claim-based database	DPC	JROAD-DPC	Nationwide		2012	2014	Intervention effect		Diseases of the circulatory system	acute heart failure	Others	aged ≥20 years
Kunisawa 2019	[Regional comparison of health care quality using DPC data]*	DPC		Nationwide		2011	2016	Quality of care	Socioeconomic comparison	Diseases of the circulatory system	acute heart failure, stroke		
Chen 2021	Disease Management and Outcomes in Patients Hospitalized for Acute Heart Failure in Japan	MDV		Nationwide		2013	2017	Medical treatment status		Diseases of the circulatory system	acute heart failure	Others	Adult patients
Ide 2021	Clinical Characteristics and Outcomes of Hospitalized Patients With Heart Failure From the Large-Scale Japanese Registry Of Acute Decompensated Heart Failure (JROADHF)	DPC	JROAD	Nationwide		2013	2017	Clinical epidemiology, course of diseases		Diseases of the circulatory system	heart failure		
Kaneko 2021	Early Initiation of Feeding and In-Hospital Outcomes in Patients Hospitalized for Acute Heart Failure	DPC		Nationwide		2010	2018	Intervention effect		Diseases of the circulatory system	acute heart failure	Others	aged ≥20 years
Yasui 2021	Association between ambulance use and hospitalization costs among heart failure patients	DPC		Nationwide		2014	2015	Health economics		Diseases of the circulatory system	heart failure		
Itoh 2020	Serial Changes in Clinical Presentations and Outcomes of 5,740 Patients Requiring Repeated Hospital Admissions (Four or More Times) due to Worsened Heart Failure	DPC		Nationwide		2010	2018	Clinical epidemiology, course of diseases		Diseases of the circulatory system	heart failure	Others	aged ≥20 years
Kaneko 2020	Association between the number of hospital admissions and in-hospital outcomes in patients with heart failure	DPC		Nationwide		2010	2018	Clinical epidemiology, course of diseases	Medical treatment status	Diseases of the circulatory system	heart failure	Others	aged ≥20 years
Konishi 2020	Impact of population density on mortality in patients hospitalized for heart failure - JROAD-DPC Registry Analysis	DPC	J-ROAD-DPC	Nationwide		2012	2015	Clinical epidemiology, course of diseases	Socioeconomic comparison	Diseases of the circulatory system	heart failure	Others	aged ≥20 years
Mitani 2020	In-hospital resource utilization, worsening heart failure, and factors associated with length of hospital stay in patients with hospitalized heart failure: A Japanese database cohort study	MDV		Nationwide		2012	2016	Clinical epidemiology, course of diseases	Medical treatment status	Diseases of the circulatory system	heart failure	Others	aged ≥20 years
Kanaoka 2019	Hospitalization Costs for Patients With Acute Congestive Heart Failure in Japan	DPC	J-ROAD	Nationwide		2012	2015	Health economics		Diseases of the circulatory system	Acute Congestive Heart Failure	Others	aged ≥20 years
Kanaoka 2019	Number of Cardiologists per Cardiovascular Beds and In-Hospital Mortality for Acute Heart Failure: A Nationwide Study in Japan	DPC	J-ROAD	Nationwide		2012	2014	Health policy evaluation and utilization		Diseases of the circulatory system	Acute Heart Failure	Others	aged ≥20 years
Suzuki 2019	Effectiveness of Early Rehabilitation for Acute Heart Failure: A RETROSPECTIVE COHORT STUDY	JMDC		Nationwide		2014	2017	Intervention effect		Diseases of the circulatory system	Acute Heart Failure	Older persons	aged ≥65 years
Mizuno 2017	The impact of carperitide usage on the cost of hospitalization and outcome in patients with acute heart failure: High value care vs. low value care campaign in Japan	DPC		Nationwide		2014	2015	Intervention effect	Health economics	Diseases of the circulatory system	acute heart failure	Others	aged ≥18 years
Aizawa 2015	Factors associated with 30-day readmission of patients with heart failure from a Japanese administrative database	DPC		Nationwide		2012	2013	Clinical epidemiology, course of diseases		Diseases of the circulatory system	Heart failure		age categories
Akazawa 2013	Economic impact of switching to fixed-dose combination therapy for Japanese hypertensive patients: a retrospective cost analysis	Others	Nihon Chouzai pharmacy claims database	Prefecture	Tokyo	2010	2011	Health economics	Health policy evaluation and utilization	Diseases of the circulatory system	hypertensive patients		
Ishida 2019	Treatment patterns of antihypertensive fixed-dose combinations according to age and number of agents prescribed: Retrospective analysis using a Japanese claims database	MDV		Nationwide		2014	2015	Medical treatment status		Diseases of the circulatory system	hypertension		
Ishida 2018	Current use of antihypertensive drugs in Japanese patients with hypertension: Analysis by age group	MDV		Nationwide		2013	2015	Medical treatment status	Socioeconomic comparison	Diseases of the circulatory system	hypertension		age group
Kuriyama 2015	Predicting failure to follow-up screened high blood pressure in Japan: a cohort study	JMDC		Nationwide		2005	2011	Patient health service utilization		Diseases of the circulatory system	hypertension	Others	aged ≥20 years
Nishimura 2020	Adherence to antihypertensive medication and its predictors among non-elderly adults in Japan	JMDC		Nationwide		2014	2016	Patient health service utilization		Diseases of the circulatory system	hypertensive patients	Others	30-74 years of age
Fukushima 2019	Impact of Hypertension on Hospitalizations for Cardiovascular Diseases in a Worksite Population: An Epidemiologic Study Using Claims Data for Workers	JMDC		Nationwide		2008	2015	Clinical epidemiology, course of diseases	Intervention effect	Diseases of the circulatory system	Cardiovascular Diseases, hypertension	Others	aged 40–64 years
Ishida 2019	Current prescription status of antihypertensive drugs in Japanese patients with hypertension: Analysis by type of comorbidities	MDV		Nationwide		2014	2015	Quality of care		Diseases of the circulatory system	hypertension	Others	aged ≥20 years
Takahashi 2017	[Antihypertensive Drug Use for Resistant Hypertension in Real-World Settings at Hospitals with Diagnosis Procedure Combination (DPC) System in Japan]	MDV	MDV	Nationwide		2013	2013	Medical treatment status		Diseases of the circulatory system	Resistant Hypertension	Others	aged ≥36 years
Hiroi 2016	Analysis of second- and third-line antihypertensive treatments after initial therapy with an angiotensin II receptor blocker using real-world Japanese data	Others	JMDC and MDV	Nationwide		2008	2015	Medical treatment status		Diseases of the circulatory system	hypertensive Japanese patients		
Hiragi 2021	Association between the size of healthcare facilities and the intensity of hypertension therapy: a cross-sectional comparison of prescription data from insurance claims data	NDB	Sampling data	Nationwide		2014	2014	Medical treatment status	Health policy evaluation and utilization	Diseases of the circulatory system	antihypertensive therapies		
Kaneko 2020	Association of Isolated Diastolic Hypertension Based on the Cutoff Value in the 2017 American College of Cardiology/American Heart Association Blood Pressure Guidelines With Subsequent Cardiovascular Events in the General Population	JMDC		Nationwide		2005	2018	Clinical epidemiology, course of diseases	Intervention effect	Diseases of the circulatory system	isolated diastolic hypertension	Others	aged ≥20 years
Ishida 2019	Treatment patterns and adherence to antihypertensive combination therapies in Japan using a claims database	MDV		Nationwide		2014	2016	Medical treatment status	Patient health service utilization	Diseases of the circulatory system	hypertension	Others	aged ≥20 years
Ohishi 2019	Analysis of antihypertensive treatment using real-world Japanese data: the retrospective study of antihypertensives for lowering blood pressure(REAL) study	Others	JMDC, MDV	Nationwide		2008	2016	Medical treatment status		Diseases of the circulatory system	hypertension		
Saito 2016	Medication-taking behavior in hypertensive patients with a single-tablet, fixed-dose combination in Japan	Others	Jamm Net (Study 2)	Nationwide		2013	2014	Patient health service utilization		Diseases of the circulatory system	hypertensive patients	Others	aged less than 75 years old

Shima 2015	[Evaluation of Concomitant Drug Use Among Patients Prescribed Amlodipine 10 mg Daily]	Others	MinaCare	Nationwide		2010	2014	Medical treatment status		Diseases of the circulatory system		hypertension		
Suzuki 2015	[Patients' Adherence of Fixed-Dose Single Pill of Amlodipine/Atorvastatin in Comparison with Separate-Pill Amlodipine + Rosuvastatin Combination Therapy (Retrospective Cohort Study)]	Others	Iyaku-Joho-Kenkyujo	Nationwide		2011	2013	Patient health service utilization		Diseases of the circulatory system		anti-hypertensive pill		
Kohro 2013	The impact of a change in hypertension management guidelines on diuretic use in Japan: trends in antihypertensive drug prescriptions from 2005 to 2011	JMDC		Nationwide		2005	2011	Medical treatment status	Health policy evaluation and utilization	Diseases of the circulatory system		hypertension	Others	aged ≥20 years
Kiriyama 2021	Surgical treatment for infective endocarditis in the ageing society: a nationwide retrospective study in Japan	DPC		Nationwide		2010	2018	Clinical epidemiology, course of diseases	Medical treatment status	Diseases of the circulatory system		infective endocarditis	Others	aged ≥20 years
Ohbe 2021	Increased risk of infective endocarditis after traumatic skin wound	Others	JMDC, National Health Insurance and Kumamoto database (Later-Stage Elderly Healthcare System)	Others	JMDC and Kumamoto database	2012	2018	Intervention effect		Diseases of the circulatory system		infective endocarditis (I33)	Others	aged ≥20 years
Kurogi 2018	Comparing intracerebral hemorrhages associated with direct oral anticoagulants or warfarin	DPC	J-ASPECT Study	Nationwide		2010	2015	Clinical epidemiology, course of diseases	Intervention effect	Diseases of the circulatory system		intracerebral hemorrhages (I61.0-9, I62.0-1, and I62.9)		
Matsuda 2018	[Big data analysis of medical and nursing care: An analysis of the current status of PCI implementation for elderly people with ischemic heart disease requiring long-term care]*	NHI/LSEHS	medical insurance claims and LTC insurance claims data	Others	A municipality in Western Japan	2011	2017	Medical treatment status	Quality of care	Diseases of the circulatory system		ischemic heart disease	Older persons	Older People
Hatanaka 2015	[Risk factors for ischemic heart disease in males in the prime of life: An eight-year follow-up study]	Others	DENSO Health Insurance Society	Others	DENSO Health Insurance	2003	2011	Clinical epidemiology, course of diseases		Diseases of the circulatory system		ischemic heart disease	Others	30-55 years old
Huang 2012	Impact of hypertension, diabetes and dyslipidemia on ischemic heart disease among Japanese: A case-control study based on National Health Insurance medical claims	NHI/LSEHS	NHI	Prefecture	Nagasaki	2010	2010	Clinical epidemiology, course of diseases		Diseases of the circulatory system	Endocrine, nutritional and metabolic diseases	Ischemic heart disease (hypertension, diabetes and dyslipidemia)	Others	40-79 years old
Tsujita 2020	Upper gastrointestinal bleeding in Japanese patients with ischemic heart disease receiving vonoprazan or a proton pump inhibitor with multiple antithrombotic agents: A nationwide database study	MDV		Nationwide		2016	2017	Intervention effect		Diseases of the circulatory system		ischemic heart disease (I20–I25)		
Fujita 2017	[Evaluation of Secondary Healthcare Area on ischemic heart diseases from data of beneficiaries of Fukuoka Branch of National Health Insurance Association]	JHIA	Fukuoka Branch	Prefecture	Fukuoka	2014	2015	Medical treatment status	Health policy evaluation and utilization	Diseases of the circulatory system		ischemic heart diseases		
Fujita 2017	[A study on hospitalization behavior and setting of secondary medical care zones by insurer for ischemic heart disease using data on health insurance claims]*	NHI/LSEHS	National Health Insurance and Later-Stage Elderly Healthcare System	Prefecture	Fukuoka	2014	2015	Medical treatment status	Health policy evaluation and utilization	Diseases of the circulatory system		ischemic heart diseases		
Chikuda 2014	Ischemic stroke after cervical spine injury: analysis of 11,005 patients using the Japanese Diagnosis Procedure Combination database	DPC		Nationwide		2007	2010	Clinical epidemiology, course of diseases		Diseases of the circulatory system		Ischemic stroke		
Yamashita 2020	Comparison of hospital length of stay of acute ischemic stroke patients with non-valvular atrial fibrillation started on rivaroxaban or warfarin treatment during hospitalization	MDV		Nationwide		2012	2015	Intervention effect		Diseases of the circulatory system		acute ischemic stroke	Others	≥20 year of age
Miyamoto 2020	Expanded Indication for Recombinant Tissue Plasminogen Activator from 3 to 4.5 h after Onset of Stroke in Japan	DPC		Nationwide		2010	2014	Intervention effect		Diseases of the circulatory system		acute ischemic stroke	Others	aged ≥18 years
Kada 2019	National trends in outcomes of ischemic stroke and prognostic influence of stroke center capability in Japan, 2010-2016	DPC	J-ASPECT (Japan Neurosurgical Society and Japan Stroke Society)	Nationwide		2010	2016	Clinical epidemiology, course of diseases	Health policy evaluation and utilization	Diseases of the circulatory system		acute ischemic stroke		
Otsubo 2015	Regional variations in in-hospital mortality, care processes, and spending in acute ischemic stroke patients in Japan	NDB	National Health Insurance beneficiaries and Long-Life Medical Care System beneficiaries	Prefecture	8 prefectures (Fukui, Mie, Shiga, Kyoto, Osaka, Nara, Wakayama, and Hyogo)	2010	2012	Medical treatment status	Socioeconomic comparison	Diseases of the circulatory system		ischemic stroke	Older persons	aged ≥65 years
Lee 2013	Derivation and validation of in-hospital mortality prediction models in ischaemic stroke patients using administrative data	DPC	QIP	Nationwide	176 hospitals	2010	2011	Prediction model	Clinical epidemiology, course of diseases	Diseases of the circulatory system		ischaemic stroke	Others	aged ≥18 years
Maeda 2021	Risk of Stroke in Atrial Fibrillation According to Sex in Patients Aged Younger Than 75 Years: A Large-Scale, Observational Study Using Real-World Data	JMDC		Nationwide		2005	2017	Clinical epidemiology, course of diseases		Diseases of the circulatory system		ischaemic stroke in patients with atrial fibrillation	Others	aged ≥20 years
Yagi 2017	Impact of Rehabilitation on Outcomes in Patients With Ischemic Stroke: A Nationwide Retrospective Cohort Study in Japan	DPC		Nationwide		2012	2014	Intervention effect		Diseases of the circulatory system		Ischemic Stroke	Others	aged >20 years
Iwamoto 2015	Effectiveness of Hospital Functions for Acute Ischemic Stroke Treatment on In-Hospital Mortality: Results From a Nationwide Survey in Japan	DPC		Nationwide		2010	2010	Health policy evaluation and utilization		Diseases of the circulatory system		acute ischemic stroke	Others	aged ≥20 years
Wada 2015	Relationship between hospital volume and early outcomes in acute ischemic stroke patients treated with recombinant tissue plasminogen activator	DPC		Nationwide		2010	2012	Health policy evaluation and utilization		Diseases of the circulatory system		ischemic stroke		
Iihara 2014	Effects of comprehensive stroke care capabilities on in-hospital mortality of patients with ischemic and hemorrhagic stroke: J-ASPECT study	DPC	J-ASPECT Study	Nationwide		2010	2011	Health policy evaluation and utilization		Diseases of the circulatory system		Ischemic and Hemorrhagic Stroke		
Kunisawa 2013	Association of geographical factors with administration of tissue plasminogen activator for acute ischemic stroke	DPC		Nationwide		2010	2012	Socioeconomic comparison	Medical treatment status	Diseases of the circulatory system		Acute ischemic stroke	Others	aged ≥18 years
Wada 2014	Effects of edaravone on early outcomes in acute ischemic stroke patients treated with recombinant tissue plasminogen activator	DPC		Nationwide		2010	2012	Intervention effect		Diseases of the circulatory system		acute ischemic stroke		
Usui 2019	Association of dialysis with in-hospital disability progression and mortality in community-onset stroke	DPC		Nationwide		2010	2013	Clinical epidemiology, course of diseases	Intervention effect	Diseases of the circulatory system	Diseases of the genitourinary system	stroke, dialysis	Others	aged ≥20 years

Maeda 2020	Increased Incident Ischemic Stroke Risk in Advanced Kidney Disease: A Large-Scale Real-World Data Study	JMDC		Nationwide		2005	2017	Clinical epidemiology, course of diseases	Intervention effect	Diseases of the circulatory system	Diseases of the genitourinary system	Ischemic Stroke, Advanced Kidney Disease	Others	aged ≥20 years
Ren 2021	Measuring quality of care for ischemic stroke treated with acute reperfusion therapy in Japan — the close the gap-stroke —	DPC	J-ASPECT study	Nationwide		2013	2015	Quality of care		Diseases of the circulatory system	Diseases of the nervous system	acute ischemic stroke (ICD-10 codes: I63.0–I63.9) or transient ischemic attack (G45)	Others	aged ≥18 years
Naganuma 2016	Clinical features of isolated dissections of abdominal aortic branches	DPC		Nationwide		2010	2013	Clinical epidemiology, course of diseases		Diseases of the circulatory system		isolated dissections of abdominal aortic branches		
Mizuno 2018	Real clinical management of patients with isolated superior mesenteric artery dissection in Japan	DPC		Nationwide	141 hospitals	2015	2015	Clinical epidemiology, course of diseases	Medical treatment status	Diseases of the circulatory system		isolated superior mesenteric artery dissection		
Otsubo 2012	[Readmission rate for health care delivery system assessment]	NHI/LSEHS	National Health Insurance and Later-Stage Elderly Healthcare System	Prefecture	Kyoto	2009	2010	Research methodology	Health policy evaluation and utilization	Diseases of the circulatory system		acute myocardial infarction		
Kanazawa 2020	In-hospital cardiac rehabilitation and clinical outcomes in patients with acute myocardial infarction after percutaneous coronary intervention: a retrospective cohort study	DPC		Nationwide		2012	2014	Intervention effect		Diseases of the circulatory system		acute myocardial infarction	Others	≥18 years of age
Miki 2019	Association of intensive care unit admission and mortality in patients with acute myocardial infarction	MDV		Nationwide		2014	2016	Intervention effect		Diseases of the circulatory system		acute myocardial infarction		age categories
Kanaoka 2018	Number of Board-Certified Cardiologists and Acute Myocardial Infarction-Related Mortality in Japan - JROAD and JROAD-DPC Registry Analysis	DPC	JROAD-DPC	Nationwide		2012	2014	Health policy evaluation and utilization		Diseases of the circulatory system		acute myocardial infarction	Others	aged ≥20 years
Isogai 2015	Effect of weekend admission for acute myocardial infarction on in-hospital mortality: a retrospective cohort study	DPC	DPC Study Group	Nationwide		2010	2013	Health policy evaluation and utilization		Diseases of the circulatory system		acute myocardial infarction	Others	aged ≥20 years
Matoba 2021	Institutional Characteristics and Prognosis of Acute Myocardial Infarction With Cardiogenic Shock in Japan - Analysis From the JROAD/JROAD-DPC Database	DPC	J-ROAD-DPC	Nationwide		2012	2016	Clinical epidemiology, course of diseases	Health policy evaluation and utilization	Diseases of the circulatory system		Acute Myocardial Infarction		
Nakao 2019	Prescription Rates of Guideline-Directed Medications Are Associated With In-Hospital Mortality Among Japanese Patients With Acute Myocardial Infarction: A Report From JROAD - DPC Study	DPC	JROAD-DPC	Nationwide		2012	2013	Intervention effect	Quality of care	Diseases of the circulatory system		Acute Myocardial Infarction	Others	aged ≥18 years
Uemura 2019	Primary Percutaneous Coronary Intervention in Elderly Patients With Acute Myocardial Infarction - An Analysis From a Japanese Nationwide Claim-Based Database	DPC	JROAD-DPC	Nationwide		2012	2015	Intervention effect	Medical treatment status	Diseases of the circulatory system		Acute Myocardial Infarction		
Ando 2018	Positive predictive value of ICD-10 codes for acute myocardial infarction in Japan: a validation study at a single center	Others	DPC and non-DPC claims data, electronic medical records	Others	a large tertiary-care university hospital	2009	2011	Research methodology		Diseases of the circulatory system		acute myocardial infarction		
Isogai 2016	Atrial natriuretic peptide therapy and in-hospital mortality in acute myocardial infarction patients undergoing percutaneous coronary intervention	DPC		Nationwide		2010	2014	Intervention effect		Diseases of the circulatory system		acute myocardial infarction	Others	aged ≥20 years
Yasuda 2016	The current status of cardiovascular medicine in Japan – Analysis of a large number of health records from a nationwide claim-based database, JROAD-DPC	DPC	JROAD-DPC	Nationwide		2012	2013	Clinical epidemiology, course of diseases	Medical treatment status	Diseases of the circulatory system		acute myocardial infarction and heart failure		
Nakai 2021	Validation of Acute Myocardial Infarction and Heart Failure Diagnoses in Hospitalized Patients With the Nationwide Claim-Based JROAD-DPC Database	DPC	JROAD-DPC, clinical dataset (from chart review)	Others	5 institutions	2012	2014	Research methodology		Diseases of the circulatory system		Acute Myocardial Infarction and Heart Failure (I21.0–I21.9 and I50.0–I50.9)		
Kikuchi 2021	Database Analysis on the Relationships Between Nonsteroidal Anti-inflammatory Drug Treatment Variables and Incidence of Acute Myocardial Infarction in Japanese Patients with Osteoarthritis and Chronic Low Back Pain	JMDC		Nationwide		2009	2018	Intervention effect		Diseases of the circulatory system	Diseases of the musculoskeletal system and connective tissue	acute myocardinal infarction, osteoarthritis and chronic low back pain	Others	≥18 years of age
Isogai 2017	Treatments and in-hospital mortality in acute myocardial infarction patients with rheumatoid arthritis: a nationwide retrospective cohort study in Japan	DPC		Nationwide		2010	2014	Medical treatment status	Clinical epidemiology, course of diseases	Diseases of the circulatory system	Diseases of the musculoskeletal system and connective tissue	acute myocardial infarction, rheumatoid arthritis	Others	aged ≥20 years
Ohki 2021	Pediatric Fulminant Myocarditis in Japan: A Retrospective Nationwide Database Study of Hospital Volume, Management Practices, and Mortality	DPC		Nationwide		2012	2018	Medical treatment status	Clinical epidemiology, course of diseases	Diseases of the circulatory system		acute myocarditis	Children	less than 18 years old
Isogai 2015	Effect of intravenous immunoglobulin for fulminant myocarditis on in-hospital mortality: propensity score analyses	DPC	DPC Study Group	Nationwide		2007	2013	Intervention effect		Diseases of the circulatory system		Fulminant myocarditis	Others	aged ≥16 years
Hosaka 2014	Clinical and economic burden in patients with diagnosis of peripheral arterial disease in a claims database in Japan	JMDC		Nationwide		2005	2011	Clinical epidemiology, course of diseases	Health economics	Diseases of the circulatory system		peripheral arterial disease	Others	45-64 years old
Isogai 2015	Effectiveness of inferior vena cava filters on mortality as an adjuvant to antithrombotic therapy	DPC		Nationwide		2007	2013	Intervention effect		Diseases of the circulatory system		pulmonary embolism (ICD-10 codes: I26.0, I26.9)		
Makito 2021	Spinal epidural hematoma and abscess after neuraxial anesthesia: a historical cohort study using the Japanese Diagnosis Procedure Combination database	DPC		Nationwide		2010	2017	Intervention effect		Diseases of the circulatory system		Spinal epidural hematoma	Others	aged ≥20 years
Kakizaki 2018	[Study on the probability of incident stroke and acute myocardial infarction using DPC data]	DPC	DPC data, Disease Registries for JPHC Research (comparison of the number of cases)	Others	a single center	2011	2012	Research methodology		Diseases of the circulatory system		stroke, acute myocardial infarction	Others	aged ≥40, <70 years
Kumazawa 2019	Association between Angiotensin-Converting Enzyme Inhibitors and Post-Stroke Aspiration Pneumonia	DPC		Nationwide		2010	2016	Intervention effect		Diseases of the circulatory system	Diseases of the respiratory system	post-stroke (ICD-10 codes: I60-I63) aspiration pneumonia (J13-J18 and J69)	Others	aged ≥50 years
Momosaki 2016	Proton Pump Inhibitors versus Histamine-2 Receptor Antagonists and Risk of Pneumonia in Patients with Acute Stroke	DPC		Nationwide		2010	2013	Intervention effect		Diseases of the circulatory system	Diseases of the respiratory system	occurrence of pneumonia in acute stroke patients		
Sakai 2013	How Does the Accuracy of ICD Coding Influence for Resource Consumption Under the Casemix Based Evaluation	DPC		Nationwide		2008	2008	Health economics	Health policy evaluation and utilization	Diseases of the circulatory system		stroke cases		
Hosoi 2020	Association between comprehensive geriatric assessment and short-term outcomes among older adult patients with stroke: A nationwide retrospective cohort study using propensity score and instrumental variable methods	DPC		Nationwide		2014	2017	Intervention effect		Diseases of the circulatory system		stroke (ICD-10 code: I63)	Older persons	aged 65 or older
Nishi 2017	Impact of financial incentives for inter-provider care coordination on health-care resource utilization among elderly acute stroke patients	NHI/LSEHS	claims data of the DPC/PDPS	Prefecture	Fukuoka Prefecture	2010	2015	Health policy evaluation and utilization		Diseases of the circulatory system		acute stroke (ICD10 codes: I60-63)	Older persons	aged 75 years or older
Nishimura 2019	Development of Quality Indicators of Stroke Centers and Feasibility of Their Measurement Using a Nationwide Insurance Claims Database in Japan - J-ASPECT Study	DPC	J-ASPECT	Nationwide		2013	2015	Quality of care		Diseases of the circulatory system		stroke	Others	Exclusion: <18 years of age
Yasaka 2018	Recurrent Stroke and Bleeding Events after Acute Cardioembolic Stroke-Analysis Using Japanese Healthcare Database from Acute-Care Institutions	MDV		Nationwide		2008	2013	Clinical epidemiology, course of diseases	Medical treatment status	Diseases of the circulatory system		cardioembolic stroke		

Wada 2016	Outcomes of Argatroban Treatment in Patients With Atherothrombotic Stroke: Observational Nationwide Study in Japan	DPC		Nationwide		2010	2012	Intervention effect		Diseases of the circulatory system		atherothrombotic stroke	Others	aged ≥40 years
Amin 2019	Trends in hospital standardized mortality ratios for stroke in Japan between 2012 and 2016: a retrospective observational study	DPC	Medi-Target benchmarking project (All Japan Hospital Association: AJHA)	Nationwide		2012	2016	Clinical epidemiology, course of diseases		Diseases of the circulatory system		stroke		
Wada 2016	Ozagrel for Patients With Noncardioembolic Ischemic Stroke: A Propensity Score-Matched Analysis	DPC		Nationwide		2010	2012	Intervention effect		Diseases of the circulatory system		noncardioembolic stroke	Others	aged ≥40 years
Matsui 2015	Variation in Risk-Standardized Mortality of Stroke among Hospitals in Japan	DPC		Nationwide		2012	2013	Health policy evaluation and utilization		Diseases of the circulatory system		stroke	Others	aged ≥20 years
Ido 2020	Effect of treatment modality and cerebral vasospasm agent on patient outcomes after aneurysmal subarachnoid hemorrhage in the elderly aged 75 years and older	DPC	J-ASPECT Study Diagnosis Procedure Combination	Nationwide		2010	2014	Intervention effect		Diseases of the circulatory system		subarachnoid hemorrhage		
Matsuda 2018	[Big data analysis of medical and nursing care: An analysis of the prognosis of patients with subarachnoid hemorrhage: In relation to normal pressure hydrocephalus]*	NHI/LSEHS	medical insurance claims and LTC insurance claims data	Others	A municip ality in Western Japan	2011	2016	Clinical epidemiology, course of diseases		Diseases of the circulatory system		subarachnoid hemorrhage		
Hironaka 2020	Outcomes in Elderly Japanese Patients Treated for Aneurysmal Subarachnoid Hemorrhage: A Retrospective Nationwide Study	DPC		Nationwide		2010	2016	Clinical epidemiology, course of diseases	Medical treatment status	Diseases of the circulatory system		aneurysmal subarachnoid hemorrhage (ICD10 codes, I60.0-I60.7)		age groups
Ikawa 2020	Risk Management of Aneurysmal Subarachnoid Hemorrhage by Age and Treatment Method from a Nationwide Database in Japan	DPC		Nationwide		2010	2015	Intervention effect		Diseases of the circulatory system		aneurysmal subarachnoid hemorrhage		age group
Ikawa 2020	In-hospital mortality and poor outcome after surgical clipping and endovascular coiling for aneurysmal subarachnoid hemorrhage using nationwide databases: a systematic review and meta-analysis	DPC	systematic review	Nationwide		2010	2015	Intervention effect		Diseases of the circulatory system		aneurysmal subarachnoid hemorrhage		
Kurogi 2020	Effects of case volume and comprehensive stroke center capabilities on patient outcomes of clipping and coiling for subarachnoid hemorrhage	DPC	J-ASPECT	Nationwide		2010	2015	Health policy evaluation and utilization	Intervention effect	Diseases of the circulatory system		subarachnoid hemorrhage		
Kurogi 2018	Effect of treatment modality on in-hospital outcome in patients with subarachnoid hemorrhage: a nationwide study in Japan (J-ASPECT Study)	DPC	J-ASPECT Study	Nationwide		2012	2013	Intervention effect		Diseases of the circulatory system		subarachnoid hemorrhage (I60.0–9)		
Oda 2021	Snowfall reduces the risk of chronic subdural hematoma onset: Analysis of an administrative database in Japan	DPC		Nationwide		2010	2016	Clinical epidemiology, course of diseases		Diseases of the circulatory system	Injury, poisoning and certain other consequences of external causes	chronic subdural hematoma (I62.0, S65.0)	Others	aged ≥40 years
Kanaoka 2020	Role of climatic factors in the incidence of Takotsubo syndrome: A nationwide study from 2012 to 2016	DPC	JROAD-DPC (Japanese Circulation Society)	Nationwide		2012	2016	Clinical epidemiology, course of diseases		Diseases of the circulatory system		Takotsubo syndrom (ICD-10 code I518)	Others	aged ≥20 years
Isogai 2018	Clinical characteristics of patients with Takotsubo syndrome diagnosed without coronary artery evaluation: A retrospective nationwide study	DPC		Nationwide		2011	2014	Medical treatment status		Diseases of the circulatory system		Takotsubo syndrome	Others	aged ≥20 years
Isogai 2017	Seasonal variation in patient characteristics and in-hospital outcomes of Takotsubo syndrome: a nationwide retrospective cohort study in Japan	DPC		Nationwide		2011	2013	Clinical epidemiology, course of diseases		Diseases of the circulatory system		Takotsubo syndrome (I42.8, I42.9 or I51.8)	Others	aged ≥20 years
Isogai 2014	Out-of-hospital versus in-hospital Takotsubo cardiomyopathy: analysis of 3719 patients in the Diagnosis Procedure Combination database in Japan	DPC		Nationwide		2010	2013	Clinical epidemiology, course of diseases		Diseases of the circulatory system		Takotsubo cardiomyopathy		
Isogai 2019	In-hospital Takotsubo syndrome versus in-hospital acute myocardial infarction among patients admitted for non-cardiac diseases: a nationwide inpatient database study	DPC		Nationwide		2010	2014	Clinical epidemiology, course of diseases		Diseases of the circulatory system		In-hospital Takotsubo syndrome versus in-hospital acute myocardial infarction	Others	aged ≥20 years
Isogai 2016	Early beta-blocker use and in-hospital mortality in patients with Takotsubo cardiomyopathy	DPC		Nationwide		2010	2014	Intervention effect		Diseases of the circulatory system		Takotsubo cardiomyopathy	Others	aged ≥20 years
Winburn 2019	Estimating the Prevalence of Transthyretin Amyloid Cardiomyopathy in a Large In-Hospital Database in Japan	MDV		Nationwide		2010	2018	Clinical epidemiology, course of diseases		Diseases of the circulatory system		Transthyretin Amyloid Cardiomyopathy	Others	aged ≥20 years
Ikawa 2020	Propensity Score Matching Analysis for the Patients of Unruptured Cerebral Aneurysm from a Post Hoc Analysis of a Nationwide Database in Japan	DPC		Nationwide		2010	2015	Intervention effect		Diseases of the circulatory system		unruptured cerebral aneurysms		age group
Ikawa 2019	Treatment Risk for Elderly Patients with Unruptured Cerebral Aneurysm from a Nationwide Database in Japan	DPC		Nationwide		2010	2015	Intervention effect		Diseases of the circulatory system		Unruptured Cerebral Aneurysm	Others	12 to 93 years of age
Sakon 2015	Economic Burden of Venous Thromboembolism in Patients Undergoing Major Abdominal Surgery	MDV		Nationwide		2003	2009	Clinical epidemiology, course of diseases	Health economics	Diseases of the circulatory system		venous thromboembolosm	Others	aged 18 years or older
Takai 2013	Economic Impact of Venous Thromboembolism Following Major Orthopaedic Surgery in Japan	MDV		Nationwide		2003	2009	Intervention effect	Health economics	Diseases of the circulatory system		Venous thromboembolism	Others	age ≥18 years
Yamashita 2020	Temporal Trends in the Practice Pattern for Venous Thromboembolism in Japan: Insight From JROAD-DPC	DPC	JROAD-DPC	Nationwide		2012	2017	Medical treatment status		Diseases of the circulatory system		venous thromboembolism	Others	aged ≥20 years
Ohyama 2019	Effect of Inferior Vena Cava Filter on Venous Thromboembolism Mortality in Japan - JROAD and JROAD-DPC Registry Analysis	DPC	JROAD-DPC	Nationwide		2012	2015	Intervention effect		Diseases of the circulatory system	Pregnancy, childbirth and the puerperium	venous thromboembolism	Others	aged ≥20 years
Nakamura 2017	Predictors of venous thromboembolism recurrence and the bleeding events identified using a Japanese healthcare database	MDV		Nationwide		2008	2013	Medical treatment status	Clinical epidemiology, course of diseases	Diseases of the circulatory system	Pregnancy, childbirth and the puerperium	venous thromboembolism		
10) Diseases of the respiratory system (J00-J99)														
Maeda 2021	Nutritional management in inpatients with aspiration pneumonia: a cohort medical claims database study	MDV		Nationwide		2013	2018	Medical treatment status		Diseases of the respiratory system		aspiration pneumonia (ICD-10, J69)	Older persons	aged ≥65 years
Matsuda 2018	[Big data analysis of medical and nursing care: An analysis of presymptomatic situations in patients with aspiration pneumonia]*	NHI/LSEHS	medical insurance claims and LTC insurance claims data	Others	A municip ality in Western Japan	2013	2014	Clinical epidemiology, course of diseases		Diseases of the respiratory system		aspiration pneumonitis	Older persons	aged 65 years or older
Momosaki 2016	Predictive factors for oral intake after aspiration pneumonia in older adults	DPC		Nationwide		2011	2012	Clinical epidemiology, course of diseases		Diseases of the respiratory system		aspiration pneumonia (ICD-10 code, J69)	Older persons	aged ≥65 years
Yagi 2016	Effect of early rehabilitation on activities of daily living in patients with aspiration pneumonia	DPC		Nationwide		2010	2013	Intervention effect		Diseases of the respiratory system		aspiration pneumonia (ICD-10 code: J69)	Older persons	excluded aged under 60 years (elderly patients)
Momosaki 2015	Effect of early rehabilitation by physical therapists on in-hospital mortality after aspiration pneumonia in the elderly	DPC		Nationwide		2010	2012	Intervention effect		Diseases of the respiratory system		aspiration pneumonia (ICD-10 codes, J69)	Older persons	aged 70 to 100 years
Momosaki 2015	Effect of dysphagia rehabilitation on oral intake in elderly patients with aspiration pneumonia	DPC		Nationwide		2010	2012	Intervention effect		Diseases of the respiratory system		aspiration pneumonia (ICD-10 codes, J69)	Older persons	excluded aged less than 65 years

Sasaki 2019	Quality Scope　医療の質への展望　「医療の質の評価・公表等推進事業」におけるDPCデータ活用による考察　誤嚥性肺炎の2指標について	DPC	(EF file)	Nationwide	68 hospitals	2016	2017	Quality of care		Diseases of the respiratory system		aspiration pneumonia		
Tsuneishi 2017	[Association between Number of Teeth and Medical Visit due to Aspiration Pneumonia in Older People Using the Receipt and Health Checkup Information Database]	NDB		Nationwide		2013	2013	Clinical epidemiology, course of diseases		Diseases of the respiratory system	Diseases of the digestive system	Number of Teeth, Aspiration Pneumonia	Older persons	aged 65 or older
Abe 2021	Trends in hospitalizations for asthma during the COVID-19 outbreak in Japan	MDV		Nationwide		2017	2020	COVID-19	Medical treatment status	Diseases of the respiratory system		asthma		children (aged <18 years) , adults (aged >18 years)
Nagase 2020	Prevalence, disease burden, and treatment reality of patients with severe, uncontrolled asthma in Japan	JMDC		Nationwide		2014	2015	Clinical epidemiology, course of diseases	Medical treatment status	Diseases of the respiratory system		asthma	Others	aged ≥17 years at the index date
Tomita 2020	Acid-suppressive medication as a possible risk factor for late-onset asthma	Others	data from 3 health insurance societies (MHI Co., Ltd)	Others	3 health insurance societies	2011	2015	Intervention effect		Diseases of the respiratory system	Others	asthma, Acid-suppressive medications	Others	40-64-year-old
Yoshida 2020	Assessment of asthma severity according to treatment steps in Japanese pediatric patients: a descriptive cross-sectional study using an administrative claims database	JMDC		Nationwide		2009	2015	Medical treatment status		Diseases of the respiratory system		asthma	Children	aged 2-15 years old
Sanada 2020	[Relationship between weather and childhood asthma: the "Health Weather" project using weather and medical data]	JMDC		Nationwide		2015	2016	Clinical epidemiology, course of diseases		Diseases of the respiratory system		asthma	Children	aged <10 years
Sato 2019	The Prevalence, Characteristics, and Patient Burden of Severe Asthma Determined by Using a Japan Health Care Claims Database	JMDC		Nationwide		2014	2016	Clinical epidemiology, course of diseases	Patient health service utilization	Diseases of the respiratory system		asthma	Others	>15-65 years of age
Atsuta 2018	Patients with Asthma Prescribed Once-Daily Fluticasone Furoate/Vilanterol or Twice-Daily Fluticasone Propionate/Salmeterol as Maintenance Treatment: Analysis from a Claims Database	JMDC		Nationwide		2014	2015	Medical treatment status	Patient health service utilization	Diseases of the respiratory system		Asthma	Others	aged ≥15 years
Yoshida 2018	Prenatal and early-life antibiotic use and risk of childhood asthma: A retrospective cohort study	JMDC		Nationwide		2005	2014	Intervention effect		Diseases of the respiratory system	Others	asthma, antibiotic use	Children	until age 6
Hamada 2015	Asthma Controller Medications for Children in Japan: Analysis of an Administrative Claims Database	JMDC		Nationwide		2008	2012	Medical treatment status		Diseases of the respiratory system		asthma	Children	aged 3 to 15 years
Nishimuta 2014	[The Factual Survey of Pediatric Asthma Treatment Using Electronic Receipt Data : Aiming for the Improvement of QOL of Both Patients and Their Parents]	JMDC		Nationwide		2005	2010	Clinical epidemiology, course of diseases	Medical treatment status	Diseases of the respiratory system		asthma	Children	pediatric asthma patients
Ishii 2020	Inhaled corticosteroid-containing regimens reduce hospitalizations and healthcare costs among elderly asthmatics: Real-world validation using the national health insurance claims database	NHI/LSEHS	NHI	Prefecture	Oita	2011	2018	Intervention effect	Health economics	Diseases of the respiratory system		asthma	Older persons	65 years of age or older
Morishima 2013	Physician adherence to asthma treatment guidelines in Japan: focus on inhaled corticosteroids	NHI/LSEHS	National Health Insurance and Long Life Medical Care System	Prefecture	Kyoto	2009	2009	Medical treatment status	Quality of care	Diseases of the respiratory system		Asthma	Others	age ≥15 years
Sato 2020	Burden of Asthma in Elderly Japanese Patients: Using Hospital-Based Administrative Claims Data	MDV		Nationwide		2014	2017	Clinical epidemiology, course of diseases	Health economics	Diseases of the respiratory system		asthma or status asthmaticus (ICD-10: codes J45 and J46)	Others	≥15 years of age
Takeuchi 2017	Risk of Acute Asthma Attacks Associated With Nonsteroidal Anti-inflammatory Drugs: A Self-Controlled Case Series	JMDC		Nationwide		2012	2013	Intervention effect		Diseases of the respiratory system		asthma		
Hasegawa 2017	Prognostic nomogram for inpatients with asthma exacerbation	DPC		Nationwide		2010	2013	Prediction model	Clinical epidemiology, course of diseases	Diseases of the respiratory system		asthma	Others	aged ≥18 years
Hirashima 2016	Effect of intravenous magnesium sulfate on mortality in patients with severe acute asthma	DPC		Nationwide		2010	2013	Intervention effect		Diseases of the respiratory system		severe acute asthma (ICD-10 code: J46)	Others	aged ≥15 years
Inoue 2019	A retrospective cohort study evaluating healthcare resource utilization in patients with asthma in Japan	JMDC		Nationwide		2009	2015	Medical treatment status	Health economics	Diseases of the respiratory system		asthma	Others	aged ≥16 years
Inoue 2021	Uncontrolled asthma: a retrospective cohort study in Japanese patients newly prescribed with medium-/high-dose ICS/LABA	JMDC		Nationwide		2009	2015	Medical treatment status	Intervention effect	Diseases of the respiratory system		uncontrolled asthma	Others	aged ≥12 years
Suzuki 2020	Clinical characteristics, treatment patterns, disease burden, and persistence/adherence in patients with asthma initiating inhaled triple therapy: real-world evidence from Japan	JMDC		Nationwide		2015	2017	Medical treatment status	Patient health service utilization	Diseases of the respiratory system		asthma	Others	≥15 years of age
To 2021	Real-world treatment and health care resource use among severe asthma patients in Japan	MDV		Nationwide		2014	2018	Clinical epidemiology, course of diseases	Health economics	Diseases of the respiratory system		asthma		
Tomita 2019	Obesity, but not metabolic syndrome, as a risk factor for late-onset asthma in Japanese women	Others	MHI Co., Ltd.	Others	three large health insurance societies	2011	2015	Clinical epidemiology, course of diseases		Diseases of the respiratory system	Endocrine, nutritional and metabolic diseases	asthma, Metabolic syndrome, Obesity	Others	aged 40-64 years
Okubo 2017	Impact of pediatric obesity on acute asthma exacerbation in Japan	DPC		Nationwide		2010	2015	Clinical epidemiology, course of diseases	Health economics	Diseases of the respiratory system	Endocrine, nutritional and metabolic diseases	acute asthma exacerbation, obesity	Children	aged 3- 8 years
Jo 2020	Inhaled corticosteroid withdrawal may improve outcomes in elderly patients with COPD exacerbation: a nationwide database study	DPC		Nationwide		2010	2016	Intervention effect		Diseases of the respiratory system		chronic obstructive pulmanary disease	Older persons	aged ≥65 years
Kitamura 2021	[The pulmonary rehabilitation utilization rate among older people with chronic obstructive pulmonary disease in Japan: A retrospective study using medical and long-term care insurance claims data]	NHI/LSEHS	medical and long-term care claims data	Municipality	Kashiwa city in Chiba	2012	2013	Medical treatment status		Diseases of the respiratory system		COPD (ICD10: J41-J44)	Older persons	aged ≥63 years
Goto 2019	Machine Learning-Based Prediction Models for 30-Day Readmission after Hospitalization for Chronic Obstructive Pulmonary Disease	DPC		Nationwide		2011	2016	Prediction model	Clinical epidemiology, course of diseases	Diseases of the respiratory system		COPD	Others	aged ≥40 years
Hasegawa 2014	Factors affecting mortality following emergency admission for chronic obstructive pulmonary disease	DPC		Nationwide		2010	2013	Clinical epidemiology, course of diseases		Diseases of the respiratory system		COPD (ICD-10 codes: J41, J42, J43, J44)	Others	aged ≥40 years
Jo 2019	Reduction in exacerbation of COPD in patients of advanced age using the Japanese Kampo medicine Dai-kenchu-to: a retrospective cohort study	DPC		Nationwide		2010	2016	Intervention effect		Diseases of the respiratory system	Diseases of the digestive system	COPD, gastrointestinal adverse effects	Older persons	aged ≥75 years
Matsui 2017	Outcomes after early and delayed rehabilitation for exacerbation of chronic obstructive pulmonary disease: a nationwide retrospective cohort study in Japan	DPC		Nationwide		2010	2013	Intervention effect		Diseases of the respiratory system		COPD		
Nakahara 2016	Mortality-Reducing Effect of Rehabilitation for COPD: Observational Propensity-Matched Cohort Study Using a Nationwide Database	DPC		Nationwide		2010	2011	Intervention effect		Diseases of the respiratory system		COPD	Others	aged ≥40 years
Murata 2010	[[Recent topics in COPD treatment] Current status of COPD patients as seen from medical fee statement (health insurance claims) data]*	JMDC		Nationwide		2005	2008	Medical treatment status		Diseases of the respiratory system		COPD	Others	aged ≥40 years

Yamauchi 2014	Paradoxical association between body mass index and in-hospital mortality in elderly patients with chronic obstructive pulmonary disease in Japan	DPC		Nationwide		2010	2013	Clinical epidemiology, course of diseases		Diseases of the respiratory system		chronic obstructive pulmonary disease	Older persons	over 65 years
Yamauchi 2016	Mortality associated with bone fractures in COPD patients	DPC		Nationwide		2010	2013	Clinical epidemiology, course of diseases		Diseases of the respiratory system	Injury, poisoning and certain other consequences of external causes	COPD, bone fracture	Others	aged 40 years and older
Hidaka 2021	Identification of risk factors for mortality and delayed oral dietary intake in patients with open drainage due to deep neck infections: A nationwide study using a Japanese inpatient database	DPC		Nationwide		2012	2017	Clinical epidemiology, course of diseases		Diseases of the respiratory system		deep neck infection		
Suzuki 2015	Factors associated with severe epiglottitis in adults: Analysis of a Japanese inpatient database	DPC		Nationwide		2011	2012	Clinical epidemiology, course of diseases		Diseases of the respiratory system		Epiglottitis (ICD-10 code: J05.1)	Others	aged 20 or older
Takeuchi 2013	The burden of epiglottitis among Japanese children before the Haemophilus influenzae type b vaccination era: an analysis using a nationwide administrative database	DPC		Nationwide		2007	2008	Clinical epidemiology, course of diseases		Diseases of the respiratory system		epiglottitis (J05.1)	Children	children ≤5 years old
Aso 2019	Systemic glucocorticoids plus cyclophosphamide for acute exacerbation of idiopathic pulmonary fibrosis: a retrospective nationwide study	DPC		Nationwide		2010	2014	Intervention effect		Diseases of the respiratory system		idiopathic pulmonary fibrosis	Others	patients aged ≥15 years
Oda 2018	Respiratory comorbidities and risk of mortality in hospitalized patients with idiopathic pulmonary fibrosis	DPC		Nationwide		2010	2013	Clinical epidemiology, course of diseases		Diseases of the respiratory system		idiopathic pulmonary fibrosis		
Urushiyama 2021	Preoperative use of pirfenidone and reduced risk of postoperative severe respiratory complications in patients with idiopathic pulmonary fibrosis: Propensity score-matched analysis using a nationwide database in Japan	DPC		Nationwide		2010	2018	Intervention effect		Diseases of the respiratory system		idiopathic pulmonary fibrosis		
Aso 2018	Effect of cyclosporine A on mortality after acute exacerbation of idiopathic pulmonary fibrosis	DPC		Nationwide		2010	2014	Intervention effect		Diseases of the respiratory system		idiopathic pulmonary fibrosis (ICD-10 codes: J84.1, J84.8, and J84.9)	Others	aged ≥15 years
Dronova 2021	Cost-effectiveness of Baloxavir Marboxil compared with Laninamivir for the treatment of influenza in patients at high risk for complications in Japan	Others	JammNet claims database (+clinical trial data and literature)	Nationwide		2016	2017	Health economics		Diseases of the respiratory system		influenza		
Shibata 2019	Influenza vaccination effectiveness for people aged under 65 years in Japan, 2013/2014 season: application of a doubly robust method to a large-scale, real-world dataset	JMDC		Nationwide		2013	2014	Intervention effect		Diseases of the respiratory system		influenza (ICD-10 codes J101, J110, J111, and J118)	Others	aged under 65 years
Miyashita 2021	Risk Factors for Pneumonia and Death in Adult Patients With Seasonal Influenza and Establishment of Prediction Scores: A Population-Based Study	NHI/LSEHS	SKDB	Prefecture	Shizuoka	2015	2018	Prediction model	Clinical epidemiology, course of diseases	Diseases of the respiratory system		influenza, pneumonia	Others	≥18 year of age
Skrzeczek 2021	Cost-effectiveness of baloxavir marboxil compared to laninamivir for the treatment of influenza in Japan	Others	JammNet claims database (sourced from the Phase III clinical trial)	Nationwide		2016	2017	Health economics		Diseases of the respiratory system		influenza	Others	12-64 year of age
Komeda 2020	Comparison of Hospitalization Incidence in Influenza Outpatients Treated With Baloxavir Marboxil or Neuraminidase Inhibitors: A Health Insurance Claims Database Study	JMDC		Nationwide		2018	2019	Intervention effect		Diseases of the respiratory system		Influenza	Others	aged <1 year were excluded
Komeda 2020	Comparison of Household Transmission of Influenza Virus From Index Patients Treated With Baloxavir Marboxil or Neuraminidase Inhibitors: A Health Insurance Claims Database Study	JMDC		Nationwide		2018	2019	Intervention effect		Diseases of the respiratory system		Influenza		
Sugawara 2019	Association of severe abnormal behavior and acetaminophen with/without neuraminidase inhibitors	NDB		Nationwide		2009	2017	Intervention effect		Diseases of the respiratory system		influenza	Children	5-19 year of age
Yokomichi 2019	Incidence of hospitalisation for severe complications of influenza virus infection in Japanese patients between 2012 and 2016: a cross-sectional study using routinely collected administrative data	JMDC		Nationwide		2012	2016	Clinical epidemiology, course of diseases		Diseases of the respiratory system	Diseases of the nervous system	influenza, influenza encephalitis/ encephalopathy	Others	0-74 year of age
Nakamura 2018	Severe abnormal behavior incidence after administration of neuraminidase inhibitors using the national database of medical claims	NDB	NDBEMC	Nationwide		2010	2014	Intervention effect		Diseases of the respiratory system		influenza	Children	5-19years
Ohkusa 2018	Comparative study of preciseness in the regional variation of influenza in Japan among the National Official Sentinel Surveillance of Infectious Diseases and the National Database of Electronic Medical Claims	NDB	Aggregated data: National Official Sentinel Surveillance of Infectious Diseases	Nationwide		2010	2014	Research methodology	Socioeconomic comparison	Diseases of the respiratory system		influenza		
Okubo 2018	Dose-response relationship between weight status and clinical outcomes in pediatric influenza-related respiratory infections	DPC		Nationwide		2010	2015	Clinical epidemiology, course of diseases		Diseases of the respiratory system		bronchitis/pneumonia and influenza	Children	aged under 18 years
Shibata 2018	Effectiveness of influenza vaccination for children in Japan: Four-year observational study using a large-scale claims database	JMDC	used application for an influenza vaccination subsidy	Nationwide		2010	2014	Intervention effect		Diseases of the respiratory system		influenza (J101, J110, J111 and J118)	Children	children aged 1–15 years
Sruamsiri 2017	Impact of patient characteristics and treatment procedures on hospitalization cost and length of stay in Japanese patients with influenza: A structural equation modelling approach	MDV		Nationwide		2014	2015	Health economics		Diseases of the respiratory system		influenza (J10.1, J11.1 and J11.8)		
Nakamura 2015	Evaluation of estimated number of influenza patients from national sentinel surveillance using the national database of electronic medical claims	NDB	Aggregated data: NOSSID, Prescription Surveillance	Nationwide		2012	2013	Clinical epidemiology, course of diseases	Research methodology	Diseases of the respiratory system		influenza		
Nakamura 2015	[Evaluation of the estimated number of influenza patients by prefecture in pharmacy surveillance using nationwide electronic medical health insurance claims]*	NDB	Aggregated data: Pharmacy Surveillance	Nationwide		2010	2013	Research methodology		Diseases of the respiratory system		influenza		
Nakano 2014	Spread of viral infection to family members from influenza patients treated with a neuraminidase inhibitor	JMDC		Nationwide		2010	2011	Intervention effect		Diseases of the respiratory system		influenza		
Sugawara 2012	Real-time prescription surveillance and its application to monitoring seasonal influenza activity in Japan	Others	electronic records related to prescription drugs	Nationwide		2009	2011	Clinical epidemiology, course of diseases		Diseases of the respiratory system		Influenza		
Takeuchi 2012	Clinical features of infants hospitalized for 2009 pandemic influenza A (H1N1) in Japan: analysis using a national hospital discharge database	DPC		Nationwide		2009	2009	Clinical epidemiology, course of diseases		Diseases of the respiratory system		InfluenzaA(H1N1)	Children	children 12 to 24 months of age
Shirado 2020	Impact of Body Mass Index on Activities of Daily Living in Patients with Idiopathic Interstitial Pneumonias	JMDC	JMDC DPC database	Nationwide		2014	2018	Clinical epidemiology, course of diseases		Diseases of the respiratory system		idiopathic interstitial pneumonia (ICD-10: code J84)	Others	over 20 years old
Sawabe 2020	Effectiveness of Early Versus Delayed Physical Rehabilitation on In-Hospital Mortality in Interstitial Pneumonia: A Retrospective Cohort Study	JMDC		Nationwide		2014	2018	Intervention effect		Diseases of the respiratory system		interstitial pneumonia (ICD-10 code J84)		
Momo 2018	Assessment of statin-induced interstitial pneumonia in patients treated for hyperlipidemia using a health insurance claims database in Japan	JMDC		Nationwide		2004	2016	Intervention effect		Diseases of the respiratory system	Endocrine, nutritional and metabolic diseases	interstitial pneumonia, hyperlipidemia		
Mouri 2019	Continuous Neuromuscular Blockade and Mortality in Subjects With Exacerbation of Idiopathic Interstitial Pneumonias	DPC		Nationwide		2010	2016	Intervention effect		Diseases of the respiratory system		idiopathic interstitial pneumonias	Others	aged ≥18 years

Mizuno 2021	Indications and postoperative outcomes of surgery for laryngotracheal stenosis: A descriptive study	MDV		Nationwide		2008	2016	Medical treatment status	Intervention effect	Diseases of the respiratory system		patients who underwent surgical treatments for laryngotracheal stenosis or defect		
Tashiro 2017	Comparison of Efficacy of Antimicrobial Agents Among Hospitalized Patients With Mycoplasma pneumoniae Pneumonia in Japan During Large Epidemics of Macrolide-Resistant M. pneumoniae Infections: A Nationwide Observational Study	DPC		Nationwide		2010	2013	Intervention effect		Diseases of the respiratory system		Mycoplasma pneumoniae infection	Others	aged ≥18 years
Tashiro 2017	Adjunctive corticosteroid therapy for inpatients with Mycoplasma pneumoniae pneumonia	DPC		Nationwide		2010	2013	Intervention effect		Diseases of the respiratory system		Mycoplasma pneumoniae pneumonia		
Shimizu 2020	Predictive Validity of Body Mass Index Cutoff Values Used in the Global Leadership Initiative on Malnutrition Criteria for Discriminating Severe and Moderate Malnutrition Based on In-Patients With Pneumonia in Asians	JMDC		Nationwide		2014	2018	Prediction model	Clinical epidemiology, course of diseases	Diseases of the respiratory system		pneumonia	Older persons	aged ≥65 years
Tashiro 2019	[Regional medical care: Current status and future forecast of medical expenses associated with pneumonia hospitalization in Niigata Prefecture: Towards the optimization of medical expenses]*	NHI/LSEHS		Prefecture	Niigata	2012	2016	Health economics	Health policy evaluation and utilization	Diseases of the respiratory system		pneumonia	Older persons	aged ≥75 years
Uematsu 2015	The Impact of Patient Profiles and Procedures on Hospitalization Costs through Length of Stay in Community-Acquired Pneumonia Patients Based on a Japanese Administrative Database	DPC	QIP	Nationwide		2012	2013	Health economics		Diseases of the respiratory system		Pneumonia	Others	age ≥15 years
Kawamoto 2011	[A comparison of the medial treatment of pneumonia in adults requiring hospitalization among 28 hospitals in the National Hospital Organization]	DPC	NHO 28 hospitals	Nationwide		2008	2008	Quality of care		Diseases of the respiratory system		Pneumonia	Others	Adults
Honda 2020	Nasogastric Tube Feeding Versus Total Parenteral Nutrition in Older Dysphagic Patients with Pneumonia: Retrospective Cohort Study	JMDC		Nationwide		2014	2017	Intervention effect		Diseases of the respiratory system		pneumonia (ICD codes: J10-18, J69)	Older persons	aged >65 years
Ichihara 2019	[Hospitalization for pneumococcal pneumonia among older adults in Fukuoka Prefecture using health insurance claims data]	NHI/LSEHS	Fukuoka Prefecture Wide-Area Association of Latter-Stage Elderly Healthcare	Prefecture	Fukuoka	2010	2017	Medical treatment status	Health economics	Diseases of the respiratory system		pneumococcal pneumonia	Older persons	aged 75 years or older
Matsuda 2019	[Big data analysis of medical and nursing care: An analysis of the length of stay in the hospital by route of admission and discharge for elderly people requiring nursing care who developed pneumonia]*	NHI/LSEHS	medical insurance claims and LTC insurance claims data	Others	A municipality in Western Japan	2011	2016	Socioeconomic comparison		Diseases of the respiratory system		pneumonia	Older persons	aged 75 years or older
Matsuda 2019	[Big data analysis of medical and nursing care: An analysis of the usage of medical and nursing care services of elderly pneumonia patients using consolidated data on health insurance claims in medical and nursing care]*	NHI/LSEHS	medical insurance claims and LTC insurance claims data	Others	A municipality in Western Japan	2012	2017	Clinical epidemiology, course of diseases	Health policy evaluation and utilization	Diseases of the respiratory system		pneumonia	Older persons	aged 65 years or older
Abe 2018	Donepezil is associated with decreased in-hospital mortality as a result of pneumonia among older patients with dementia: A retrospective cohort study	DPC		Nationwide		2010	2012	Clinical epidemiology, course of diseases	Intervention effect	Diseases of the respiratory system		pneumonia	Older persons	aged≥65 years
Kumamaru 2014	Association between hospital case volume and mortality in non-elderly pneumonia patients stratified by severity: a retrospective cohort study	DPC		Nationwide		2010	2010	Health policy evaluation and utilization		Diseases of the respiratory system	Certain infectious and parasitic diseases	pneumonia	Others	aged 18–64 years
Kobayashi 2021	Interrupted time-series analyses of routine vaccination program for elderly pneumonia patients in Japan; an ecological study using aggregated nationwide inpatient data	DPC	MSD Japan; the number of shipped PPSV23 syringes	Nationwide	exclude tohoku three prefectures	2011	2017	Clinical epidemiology, course of diseases	Health policy evaluation and utilization	Diseases of the respiratory system		pneumonia	Older persons	≥65 years of age
Nagano 2021	Hospitalization of mild cases of community-acquired pneumonia decreased more than severe ones during the COVID-19 epidemic	DPC	QIP	Nationwide		2018	2020	COVID-19	Clinical epidemiology, course of diseases	Diseases of the respiratory system	Certain infectious and parasitic diseases	pneumonia (during the COVID-19 pandemic)	Others	≥18 year of age
Yahaba 2021	Antibiotics for hospitalized children with community-acquired pneumonia in Japan: Analysis based on Japanese national database	NDB	Sampling data	Nationwide		2011	2014	Medical treatment status		Diseases of the respiratory system		Community-acquired pneumonia	Children	<15 year of age
Yamazaki 2021	Antibiotics prescriptions for pneumonia analyzed by claim information in Japan	NDB	Sampling data	Nationwide		2011	2014	Medical treatment status	Quality of care	Diseases of the respiratory system		community-acquired pneumonia	Others	aged ≥15 years
Igari 2020	Epidemiology and treatment outcome of pneumonia: Analysis based on Japan national database	NDB	Sampling data	Nationwide		2011	2014	Clinical epidemiology, course of diseases		Diseases of the respiratory system		pneumonia	Others	aged 15 years and older
Kimura 2020	Switching from intravenous to oral antibiotics in hospitalized patients with community-acquired pneumonia: A real-world analysis 2010-2018	MDV		Nationwide		2008	2018	Medical treatment status	Quality of care	Diseases of the respiratory system		community-acquired pneumonia	Others	≥20 years of age
Imamura 2020	[Survey of Blood Culture in Community-Acquired Pneumonia Patients Utilizing Health Insurance Claims Database]	Others	CISA database (13 university hospitals)	Nationwide		2014	2015	Medical treatment status	Quality of care	Diseases of the respiratory system		Community-Acquired Pneumonia	Others	≥15 years of age
Okubo 2018	Recent trends in practice patterns and impact of corticosteroid use on pediatric Mycoplasma pneumoniae-related respiratory infections	DPC		Nationwide		2010	2014	Intervention effect	Medical treatment status	Diseases of the respiratory system	Certain infectious and parasitic diseases	M. pneumoniae bronchitis and pneumonia (A49.3, J15.7, and J20.0)	Children	under 18 years
Uematsu 2017	Prediction of pneumonia hospitalization in adults using health checkup data	NHI/LSEHS	National Health Insurance and Long-Life Medical Care Insurance	Prefecture	kyoto	2010	2015	Prediction model	Clinical epidemiology, course of diseases	Diseases of the respiratory system	Certain infectious and parasitic diseases	pneumonia	Others	aged 40 to 74 years
Uematsu 2016	The economic burden of methicillin-resistant Staphylococcus aureus in community-onset pneumonia inpatients	DPC		Nationwide		2013	2014	Clinical epidemiology, course of diseases	Health economics	Diseases of the respiratory system	Certain infectious and parasitic diseases	pneumonia	Others	age 18 years or older
Uematsu 2014	Impact of guideline-concordant microbiological testing on outcomes of pneumonia	DPC		Nationwide		2010	2011	Intervention effect	Quality of care	Diseases of the respiratory system		pneumonia (ICD-10: J10– J18)	Others	aged 18 years or older
Uematsu 2014	Development of a risk-adjusted in-hospital mortality prediction model for community-acquired pneumonia: a retrospective analysis using a Japanese administrative database	DPC		Nationwide		2012	2013	Prediction model	Clinical epidemiology, course of diseases	Diseases of the respiratory system		pneumonia (ICD-10: J10–J18)	Others	aged >14 years
Amin 2020	Capturing the trends in hospital standardized mortality ratios for pneumonia: a retrospective observational study in Japan (2010 to 2018)	DPC	Medi-Target benchmarking project: AJHA	Nationwide		2010	2018	Clinical epidemiology, course of diseases		Diseases of the respiratory system	Certain infectious and parasitic diseases	pneumonia		
Kishimoto 2017	Sivelestat sodium and mortality in pneumonia patients requiring mechanical ventilation: propensity score analysis of a Japanese nationwide database	DPC		Nationwide		2012	2014	Intervention effect		Diseases of the respiratory system		pneumonia erquiring mechanical ventilation		
Ota 2019	Age distribution and seasonality in acute eosinophilic pneumonia: analysis using a national inpatient database	DPC		Nationwide		2010	2015	Clinical epidemiology, course of diseases		Diseases of the respiratory system		eonsinophilic pneumonia (J82)		
Tateda 2014	[Risk factors for the development of pneumonia: analysis of Japanese claims data]	JMDC		Nationwide		2005	2012	Clinical epidemiology, course of diseases		Diseases of the respiratory system		pneumonia		age categories
Sakamoto 2017	Guidelines-concordant empiric antimicrobial therapy and mortality in patients with severe community-acquired pneumonia requiring mechanical ventilation	DPC		Nationwide		2012	2014	Intervention effect	Quality of care	Diseases of the respiratory system		community-acquired pneumonia, antimicrobial therapy	Others	aged over 20 years

Jo 2017	Association between dementia and discharge status in patients hospitalized with pneumonia	DPC		Nationwide		2010	2014	Clinical epidemiology, course of diseases		Diseases of the respiratory system	Mental, Behavioral and Neurodevelopmental disorders	pneumonia, dimentia (F00–F03 or G30)	Older persons	aged ≥60 years
Hiyama 2018	The three peaks in age distribution of females with pneumothorax: a nationwide database study in Japan	DPC		Nationwide		2010	2016	Clinical epidemiology, course of diseases		Diseases of the respiratory system	Diseases of the genitourinary system	pneumothorax or Catamenial pneumothorax (ICD-10 code: J93, N948)		age categories
Imanaka 2019	An analysis of factors associated with compliance and dropout of sublingual immunotherapy on Japanese cedar pollinosis patients	JMDC		Nationwide		2014	2016	Patient health service utilization		Diseases of the respiratory system		cedar pollinosis		age group
Ono 2020	Relationship Between Severe Respiratory Depression and Codeine-Containing Antitussives in Children: A Nested Case-Control Study	JMDC		Nationwide		2012	2015	Intervention effect	Clinical epidemiology, course of diseases	Diseases of the respiratory system		respiratory depression	Children	children younger than 18 years
Kido 2017	Efficacy of early sivelestat administration on acute lung injury and acute respiratory distress syndrome	DPC		Nationwide		2012	2012	Intervention effect		Diseases of the respiratory system		acute lung injury and acute respiratory distress syndrome (ICD-10 code J80)		
Kido 2018	The relationship between high-dose corticosteroid treatment and mortality in acute respiratory distress syndrome: a retrospective and observational study using a nationwide administrative database in Japan	DPC		Nationwide		2012	2012	Intervention effect		Diseases of the respiratory system		acute respiratory distress syndrome (ICD-10 code J80)		
Takeshima 2019	Factors associated with severe postoperative acute respiratory failure requiring high dose corticosteroid therapy	DPC		Nationwide		2012	2013	Clinical epidemiology, course of diseases		Diseases of the respiratory system	Injury, poisoning and certain other consequences of external causes	postoperative acute respiratory failure	Others	aged ≥20 years
Kikuchi 2020	[The Prescription of Oral Antibiotics for Acute Respiratory Tract Infection in Pediatric Outpatients at Acute Diagnosis Procedure Combination Hospitals (2013-2018)]	MDV		Nationwide		2013	2018	Medical treatment status		Diseases of the respiratory system		acute respiratory tract infection	Children	aged 0 to 14 years
Yoshida 2018	Prescription of antibiotics to pre-school children from 2005 to 2014 in Japan: a retrospective claims database study	JMDC		Nationwide		2005	2014	Medical treatment status		Diseases of the respiratory system	Others	antibiotics, upper respiratory infections	Children	birth to 6 years of age
Tsuzuki 2020	Cost of inappropriate antimicrobial use for upper respiratory infection in Japan	JMDC		Nationwide		2013	2016	Health economics	Medical treatment status	Diseases of the respiratory system		upper respiratory infection	Others	under the age of 65 years
Matsuda 2018	[Big data analysis of medical and nursing care: An analysis of the current status of antimicrobial agent use for acute upper respiratory tract inflammation using data on health insurance claims]*	NHI/LSEHS	medical insurance claims (The National Health Insurance system and Late Elders' Health Insurance)	Others	A municipality in Western Japan	2015	2015	Medical treatment status		Diseases of the respiratory system		acute upper respiratory infection		all age groups
Koyama 2020	Antibiotic prescriptions for Japanese outpatients with acute respiratory tract infections (2013-2015): A retrospective Observational Study	JMDC		Nationwide		2013	2015	Medical treatment status		Diseases of the respiratory system		acute respiratory tract infections		age category
Muraki 2020	Impact of antimicrobial stewardship fee on prescribing for Japanese pediatric patients with upper respiratory infections	Others	IQVIA Claims	Nationwide		2017	2018	Health policy evaluation and utilization	Medical treatment status	Diseases of the respiratory system		Upper respiratory tract infection	Children	<15 year of age
Okubo 2020	National trends in appropriate antibiotics use among pediatric inpatients with uncomplicated lower respiratory tract infections in Japan	DPC		Nationwide		2010	2015	Medical treatment status		Diseases of the respiratory system	Certain infectious and parasitic diseases	acute lower respiratory tract infections	Children	3 month-15 year of age
Sugiyama 2020	[Prescription of Oral Antibacterial Drugs to Outpatients with Acute Respiratory Tract Infection and Acute Diarrhea : The Survey Based on Medical Data Using MDV analyzer]	MDV		Nationwide		2013	2019	Medical treatment status		Diseases of the respiratory system	Certain infectious and parasitic diseases	Acute respiratory tract infection, Acute diarrhea		
Hashimoto 2019	Antibiotic prescription among outpatients in a prefecture of Japan, 2012-2013: a retrospective claims database study	NHI/LSEHS	national or late elders' health insurance system	Prefecture	Kumamoto	2012	2013	Medical treatment status		Diseases of the respiratory system	Certain infectious and parasitic diseases	Acute respiratory tract infections, gastrointestinal infections		
Kimura 2019	Longitudinal trends of and factors associated with inappropriate antibiotic prescribing for non-bacterial acute respiratory tract infection in Japan: A retrospective claims database study, 2012-2017	JMDC		Nationwide		2012	2017	Medical treatment status		Diseases of the respiratory system	Certain infectious and parasitic diseases	non-bacterial acute respiratory tract infection		age groups
Teratani 2019	Pattern of antibiotic prescriptions for outpatients with acute respiratory tract infections in Japan, 2013-15: a retrospective observational study	JMDC		Nationwide		2013	2015	Medical treatment status		Diseases of the respiratory system		acute respiratory tract infections	Others	≤74 year of age
Uda 2019	Nationwide survey of indications for oral antimicrobial prescription for pediatric patients from 2013 to 2016 in Japan	NDB		Nationwide		2013	2016	Medical treatment status	Health policy evaluation and utilization	Diseases of the respiratory system	Certain infectious and parasitic diseases	respiratory infections	Children	≤15 year of age
Wada 2013	Practice patterns for lower respiratory tract infections in hospital patients with particular focus on bacteriological examinations and injection antibiotics use	JMDC		Nationwide		2004	2007	Medical treatment status	Quality of care	Diseases of the respiratory system		Lower respiratory tract infections	Others	aged 16 years or older
Suzuki 2015	Complication rates after functional endoscopic sinus surgery: analysis of 50,734 Japanese patients	DPC		Nationwide		2007	2013	Intervention effect		Diseases of the respiratory system		chronic sinusitis (ICD-10 code: J32x) or nasal polyps (J33x)	Others	aged ≥16 years
Koizumi 2019	Outcomes of endoscopic sinus surgery for sinusitis-induced intracranial abscess in patients undergoing neurosurgery	DPC		Nationwide		2010	2017	Intervention effect		Diseases of the respiratory system	Diseases of the nervous system	sinusitis-induced intracranial abscess		
Teratani 2019	Association between rapid antigen detection tests and antibiotics for acute pharyngitis in Japan: A retrospective observational study	JMDC		Nationwide		2013	2015	Medical treatment status	Quality of care	Diseases of the respiratory system		acute pharyngitis, acute tonsillitis	Others	≤74 year of age
Miyamoto 2018	Perioperative Steroid Use for Tonsillectomy and Its Association With Reoperation for Posttonsillectomy Hemorrhage: A Retrospective Cohort Study	MDV		Nationwide		2008	2014	Intervention effect		Diseases of the respiratory system	Others	tonsillectomy, posttonsillectomy hemorrhage		
11) Diseases of the digestive system (K00-K93)														
Sato 2019	Epidemiological analysis of achalasia in Japan using a large-scale claims database	JMDC		Nationwide		2005	2017	Clinical epidemiology, course of diseases	Medical treatment status	Diseases of the digestive system		achalasia		
Ishii 2019	Application of Large Electronic Medical Database for Detecting Undiagnosed Patients in the General Population	NHI/LSEHS	national medical insurance record database	Municipality	Ishinomaki City	2015	2017	Medical treatment status		Diseases of the digestive system		esophagogastric junction outflow obstruction (including achalasia)	Others	below 60 years old
Murata 2013	Association between hospital volume and outcomes of elderly and non-elderly patients with acute biliary diseases: A national administrative database analysis	DPC		Nationwide		2008	2008	Health policy evaluation and utilization		Diseases of the digestive system		acute biliary diseases	Others	50–74 years, aged 75 years or older
Tarasawa 2020	Recombinant Human Soluble Thrombomodulin Contributes to a Reduction In-Hospital Mortality of Acute Cholangitis with Disseminated Intravascular Coagulation: A Propensity Score Analyses of a Japanese Nationwide Database	DPC		Nationwide		2012	2018	Intervention effect		Diseases of the digestive system	Diseases of the blood and blood-forming organs and certain disorders involving the immune mechanism	acute cholangitis with disseminated intravascular coagulation		
Murata 2011	An observational study using a national administrative database to determine the impact of hospital volume on compliance with clinical practice guidelines	DPC		Nationwide		2008	2008	Health policy evaluation and utilization	Quality of care	Diseases of the digestive system		acute cholangitis	Others	aged ≥16 years
Murata 2010	Impact of hospital volume on clinical outcomes of endoscopic biliary drainage for acute cholangitis based on the Japanese administrative database associated with the diagnosis procedure combination system	DPC		Nationwide		2008	2008	Intervention effect	Health policy evaluation and utilization	Diseases of the digestive system		acute cholangitis		
Murata 2011	Evaluation of compliance with the Tokyo Guidelines for the management of acute cholangitis based on the Japanese administrative database associated with the Diagnosis Procedure Combination system	DPC		Nationwide		2008	2008	Quality of care		Diseases of the digestive system		acute cholangitis	Others	aged ≥16 years
Sekimoto 2010	[Practice Guidelines for Acute Cholecystitis and Acute Cholangitis: Administrative Data (DPC data) to Examine Trends in Patient Characteristics, Process of care, Patient Outcomes, and Medical Resource Utilization for Patients with Acute Cholecystitis]	DPC	QIP	Nationwide		2004	2009	Quality of care		Diseases of the digestive system		acute cholecystitis (K800 K801 K804 K810)		

Murata 2013	Multivariate analysis of factors influencing length of hospitalization and medical costs of cholecystectomy for acute cholecystitis in Japan: a national database analysis	DPC		Nationwide		2008	2008	Medical treatment status	Health economics	Diseases of the digestive system		acute cholecystitis		age categories
Murata 2012	The care processes for acute cholecystitis according to clinical practice guidelines based on the Japanese administrative database	DPC		Nationwide		2008	2008	Quality of care		Diseases of the digestive system		cholecystitis (K810)	Others	age ≥15 years
Murata 2012	[Preoperative antimicrobial therapy for patients with cholecystectomy for acute cholecystitis based on the administrative database associated with the Diagnosis Procedure Combination system]	DPC		Nationwide		2008	2008	Medical treatment status	Intervention effect	Diseases of the digestive system		acute cholecystitis		
Murata 2013	[An analysis of the current status of etiology and treatment in pediatric patients with acute pancreatitis from DPC data]*	DPC		Nationwide		2008	2008	Clinical epidemiology, course of diseases	Medical treatment status	Diseases of the digestive system		acute pancreatitis	Children	aged <15 years
Murata 2015	[Association between Hospitalization or Medical Costs and Severity of Acute Pancreatitis Based on the Diagnosis Procedure Combination Database]	DPC		Nationwide		2010	2012	Medical treatment status	Health economics	Diseases of the digestive system		acute pancreatitis		
Murata 2011	[The circumstance of antimicrobial therapy for severe acute pancreatitis in elderly patients based on the administrative database associated with the Diagnosis Procedure Combination system]	DPC		Nationwide		2008	2008	Medical treatment status		Diseases of the digestive system		acute pancreatitis	Older persons	aged 70 years and above
Ikeda Kurakawa	Effects of Prophylactic Antibiotics on Length of Stay and Total Costs for Pediatric Acute Pancreatitis: A Nationwide Database Study in Japan	DPC		Nationwide		2010	2017	Intervention effect		Diseases of the digestive system		aqute pancratitis (K85)	Children	<18 year of age
Nakaharai 2018	Early prophylactic antibiotics for severe acute pancreatitis: A population-based cohort study using a nationwide database in Japan	DPC		Nationwide		2010	2016	Intervention effect		Diseases of the digestive system		acute pancretitis	Others	≥18 years old
Endo 2018	Impact of continuous regional arterial infusion in the treatment of acute necrotizing pancreatitis: analysis of a national administrative database	DPC		Nationwide		2010	2015	Intervention effect		Diseases of the digestive system		acute necrotizing pancreatitis (K85)	Others	aged ≥16 years
Hamada 2016	No weekend effect on outcomes of severe acute pancreatitis in Japan: data from the diagnosis procedure combination database	DPC		Nationwide		2010	2013	Clinical epidemiology, course of diseases	Health policy evaluation and utilization	Diseases of the digestive system		severe acute pancreatitis (K85)	Others	aged ≥20 years
Murata 2011	A descriptive study evaluating the circumstances of medical treatment for acute pancreatitis before publication of the new JPN guidelines based on the Japanese administrative database associated with the Diagnosis Procedure Combination system	DPC		Nationwide		2008	2008	Medical treatment status	Quality of care	Diseases of the digestive system		acute pancreatitis		
Taguchi 2014	Body mass index influences the outcome of acute pancreatitis: An analysis based on the japanese administrative database	DPC		Nationwide		2010	2010	Clinical epidemiology, course of diseases		Diseases of the digestive system		acute pancreatis	Others	aged ≥20 years
Yasunaga 2013	Effect and cost of treatment for acute pancreatitis with or without gabexate mesylate: a propensity score analysis using a nationwide administrative database	DPC		Nationwide		2010	2010	Intervention effect	Health economics	Diseases of the digestive system		acute pancreatis	Others	aged ≥20 years
Iwasawa 2020	Influence of co-initiation of antiulcer drugs on persistence and adherence to low-dose aspirin: A retrospective cohort study using a Japanese claims database	JMDC		Nationwide		2005	2016	Patient health service utilization		Diseases of the digestive system	Others	low-dose aspirin, co-initiation of antiulcer drugs		
Iwasawa 2019	Adherence to guidelines for antiulcer drug prescription in patients receiving low-dose aspirin therapy in Japan	JMDC		Nationwide		2005	2016	Medical treatment status	Quality of care	Diseases of the digestive system	Others	antiulcer drug (low-dose aspirin therapy in Japan)	Others	≥18 year of age
Hamada 2015	Severe bleeding after percutaneous transhepatic drainage of the biliary system: effect of antithrombotic agents--analysis of 34 606 cases from a Japanese nationwide administrative database	DPC	DPC Research Group	Nationwide		2007	2012	Intervention effect		Diseases of the digestive system		biliary disease	Others	20 years and older
Takeuchi 2015	Descriptive epidemiology of children hospitalized for inflammatory bowel disease in Japan: Inpatient database analysis	DPC		Nationwide		2007	2010	Clinical epidemiology, course of diseases		Diseases of the digestive system		Inflammatory bowel disease	Children	≤18 years old
Hosomi 2018	An integrative approach using real-world data to identify alternative therapeutic uses of existing drugs	JMDC	FAERS: adverse events, NextBio: transcriptome data	Nationwide		2005	2016	Research methodology		Diseases of the digestive system		inflammatory bowel disease (Crohn's disease and ulcerative colitis)		
Wing 2018	Incidence of catheter-related complications among Japanese patients with central venous catheters as well as patients with short bowel syndrome	MDV		Nationwide		2008	2016	Intervention effect		Diseases of the digestive system		Short bowel syndrome		age group
Kobayashi 2020	Lack of Increased Risk of Lymphoma by Thiopurines or Biologics in Japanese Patients with Inflammatory Bowel Disease: A Large-Scale Administrative Database Analysis	MDV		Nationwide		2008	2018	Clinical epidemiology, course of diseases	Intervention effect	Diseases of the digestive system	Neoplasms	inflammatory bowel disease, lymphoma		
Sugihara 2013	Does mechanical bowel preparation improve quality of laparoscopic nephrectomy? Propensity score-matched analysis in Japanese series	DPC		Nationwide		2008	2010	Intervention effect		Diseases of the digestive system	Diseases of the genitourinary system	mechanical bowel preparation, laparoscopic nephrectomy		
Fujiogi 2018	Postoperative Small Bowel Obstruction Following Laparoscopic or Open Fundoplication in Children: A Retrospective Analysis Using a Nationwide Database	DPC		Nationwide		2010	2016	Intervention effect		Diseases of the digestive system	Injury, poisoning and certain other consequences of external causes	postoperative small bowel obstruction	Children	aged 0–18 years
Takeyama 2021	Association of diagnostic delay with medical cost for patients with Crohn's disease: A Japanese claims-based cohort study	JMDC		Nationwide		2005	2018	Medical treatment status	Health economics	Diseases of the digestive system		Chron's disease		
Yokoyama 2016	A Retrospective Claims Database Study on Drug Utilization in Japanese Patients with Crohn's Disease Treated with Adalimumab or Infliximab	JMDC		Nationwide		2012	2015	Intervention effect	Medical treatment status	Diseases of the digestive system		crohn's disease (K50)		
Murata 2020	Relationship Between the Number of Occlusal Supporting and Medical Cost: Analysis Using Large Claims Database from Employee Health Care Insurance in Japan	JMDC		Nationwide		2016	2017	Health economics		Diseases of the digestive system		gingivitis or periodontal	Others	aged over 20 years
Uene 2020	[Differences in Annual Use of Medical and dental Treatment and Cost among workers of Different Business Types: Data on Insured Persons Registered with the Japan Health Insurance Association Osaka Branch]	JHIA	Japan Health Insurance Association Osaka Branch	Prefecture	Osaka	2015	2016	Health economics	Patient health service utilization	Diseases of the digestive system		dental treatment	Others	aged between 35 and 74
Tsuneishi 2016	[Association between Number of Teeth and Medical and Dental Care Expenditure: Analysis Using the Receipt and Health Checkup Information Database in Japan]	NDB		Nationwide		2012	2013	Health economics		Diseases of the digestive system		periodontal disease	Others	40 years of age or older
Saito 2018	Type of dental visit and number of remaining teeth in Japanese elders	NHI/LSEHS		Prefecture	Mie	2014	2014	Intervention effect		Diseases of the digestive system		remaining teeth	Older persons	75 or 80 years
Murata 2018	[Big data analysis of medical and nursing care: An analysis of the current status of oral care for the elderly requiring long-term care]*	NHI/LSEHS	medical insurance claims and LTC insurance claims data	Others	A municip ality in Western Japan	2012	2016	Intervention effect	Medical treatment status	Diseases of the digestive system		Oral care	Older persons	Older People
Saito 2018	[The relationship between the number of teeth and medical expenses of elderly people: Based on the results of dental checkups and data on health insurance claims from the Mie Prefecture Advanced Elderly Medical Care Interdisciplinary Association]*	NHI/LSEHS	Dental checkup data	Prefecture	Mie	2014	2014	Health economics		Diseases of the digestive system		teeth number	Older persons	75 and 80 years old
Yoshiuchi 2011	[Survey of dental treatment expenditures based on medical expense data of dental status and general health of the elderly in Hokkaido]	NHI/LSEHS	NHI	Prefecture	Hokkaid o	2007	2007	Clinical epidemiology, course of diseases	Health economics	Diseases of the digestive system		dental status	Older persons	aged 70 years and above
Nishide 2017	Income-Related Inequalities in Access to Dental Care Services in Japan	NHI/LSEHS	NHI	Municipality	Chiba City	2014	2015	Patient health service utilization	Socioeconomic comparison	Diseases of the digestive system		dental care service		age categories
Tsuneishi 2019	[The presence of teeth type using the dental notation of periodontitis patients: a cross-sectional study using the receipt and health checkup information database in Japan]	NDB	Survey of Dental Disease (MHLW)	Nationwide		2016	2016	Clinical epidemiology, course of diseases	Research methodology	Diseases of the digestive system		Periodontitis	Others	aged ≥20 years
Miwa 2016	Medical cost, incidence rate, and treatment status of gastroesophageal reflux disease in Japan: analysis of claims data	JMDC		Nationwide		2005	2015	Clinical epidemiology, course of diseases	Health economics	Diseases of the digestive system		gastroesophageal reflux disease		all ages

Murata 2015	The changing patterns of dispensing branded and generic drugs for the treatment of gastroesophageal reflux disease between 2006 and 2011 in Japan: a retrospective cohort study	JMDC		Nationwide		2006	2011	Medical treatment status	Health policy evaluation and utilization	Diseases of the digestive system		gastroesophageal reflux disease		
Miyamoto 2020	The Effect of Carbazochrome Sodium Sulfonate in Patients with Colonic Diverticular Bleeding: Propensity Score Matching Analyses Using a Nationwide Inpatient Database	DPC		Nationwide		2010	2018	Intervention effect	Health economics	Diseases of the digestive system		patients with colonic diverticular bleeding (ICD-10: K57.3)	Others	excluded ≤17 years of age
Fujimoto 2021	Decline incidence in upper gastrointestinal bleeding in several recent years: data of the Japan claims database of 13 million accumulated patients	JMDC		Nationwide		2009	2014	Clinical epidemiology, course of diseases		Diseases of the digestive system		upper gastrointestinal bleeding	Others	20-74 years of age
Miyakuni 2020	Angiography versus colonoscopy in patients with severe lower gastrointestinal bleeding: a nation-wide observational study	DPC		Nationwide		2010	2017	Intervention effect		Diseases of the digestive system		severe lower gastrointestinal bleeding	Others	aged ≥16 years
Miyamoto 2020	Effect of tranexamic acid in patients with colonic diverticular bleeding: A nationwide inpatient database study	DPC		Nationwide		2010	2018	Intervention effect		Diseases of the digestive system		colonic diverticular bleeding	Others	aged ≥18 years
Moroi 2020	Efficacy of urgent colonoscopy for colonic diverticular bleeding: A propensity score-matched analysis using a nationwide database in Japan	DPC		Nationwide		2016	2019	Intervention effect		Diseases of the digestive system		colonic diverticular bleeding		age categories
Niikura 2015	Factors affecting in-hospital mortality in patients with lower gastrointestinal tract bleeding: a retrospective study using a national database in Japan	DPC		Nationwide		2010	2012	Clinical epidemiology, course of diseases		Diseases of the digestive system		gastrointestinal tract bleeding		
Murata 2011	Equivalent clinical outcomes of bleeding peptic ulcers in teaching and non-teaching hospitals: evidence for standardization of medical care in Japan	DPC		Nationwide		2008	2008	Quality of care	Health policy evaluation and utilization	Diseases of the digestive system		bleeding peptic ulcers		
Nagata 2018	Therapeutic endoscopy-related GI bleeding and thromboembolic events in patients using warfarin or direct oral anticoagulants: results from a large nationwide database analysis	DPC		Nationwide		2014	2015	Intervention effect		Diseases of the digestive system	Diseases of the circulatory system	GI bleeding and thromboembolic events	Others	aged ≥20 years
Nagata 2018	[High Risk Therapeutic Endoscopy: Related Gastrointestinal Bleeding and Thromboembolic Events in Patients Using Warfarin or Direct Oral Anticoagulants]	DPC		Nationwide		2014	2015	Intervention effect		Diseases of the digestive system	Diseases of the circulatory system	Gastrointestinal Bleeding and Thromboembolic events		
Kondo 2020	Follow-up Analysis after Colorectal Polypectomy on Claims Data	JMDC		Nationwide		2005	2017	Intervention effect		Diseases of the digestive system		Colorectal Polypectomy	Others	age ≥40 years, <70 years
Imai 2021	Healthcare utilization associated with adherence to antibiotics for abdominal surgeries in Japan: cross-sectional analysis of administrative database	DPC		Nationwide		2014	2016	Quality of care	Health economics	Diseases of the digestive system		abdominal surgeries	Others	aged 15 years and older
Otaka 2021	Associations between early parenteral nutrition and in-hospital outcomes in underweight patients with gastrointestinal surgery	DPC		Nationwide		2010	2018	Intervention effect		Diseases of the digestive system		patients with gastrointestinal surgery	Others	≥18 year of age
Tsuchiya 2019	Nationwide observational study of mortality from complicated intra-abdominal infections and the role of bacterial cultures	DPC		Nationwide		2014	2016	Intervention effect		Diseases of the digestive system		complicated intra-abdominal infection	Others	≥18 year of age
Abe 2021	Prevention of delayed bleeding with vonoprazan in upper gastrointestinal endoscopic treatment	DPC		Nationwide		2014	2019	Intervention effect		Diseases of the digestive system		delayed bleeding, upper gastrointestinal endoscopic treatment	Others	aged ≥20 years
Shimizu 2016	[Analysis of the complications that arose in cases of digestive tract hemostasis based on diagnosis procedure combination survey data]	DPC		Nationwide		2006	2008	Clinical epidemiology, course of diseases	Health economics	Diseases of the digestive system		digestive tract hemostasis		
Seta 2019	Status of use of protease inhibitors for the prevention and treatment of pancreatitis after endoscopic retrograde cholangiopancreatography: An epidemiologic analysis of the evidence-practice gap using a health insurance claims database	JMDC		Nationwide		2005	2015	Medical treatment status	Quality of care	Diseases of the digestive system		endoscopic retrograde cholangiopancreatography		
Sugisaki 2018	A case-control study of the risk of upper gastrointestinal mucosal injuries in patients prescribed concurrent NSAIDs and antithrombotic drugs based on data from the Japanese national claims database of 13 million accumulated patients	JMDC		Nationwide		2009	2014	Intervention effect		Diseases of the digestive system		upper gastrointestinal mucosal injuries	Others	aged 20–74 years
Takada 2013	[Study on Risk of Gastrointestinal Complications in Low-dose Aspirin Therapy Using the National Receipt Database]	NDB		Nationwide		2010	2010	Intervention effect		Diseases of the digestive system	Others	Gastrointestinal Complications, Low-dose Aspirin Therapy		
Matsuda 2018	[Big data analysis of medical and nursing care: Attribute analysis of gastrostomy patients using data on health insurance claims in medical and nursing care]*	NHI/LSEHS	medical insurance claims and LTC insurance claims data	Others	A municipality in Western Japan	2008	2016	Medical treatment status		Diseases of the digestive system		gastrostomy		
Sako 2014	Prevalence and in-hospital mortality of gastrostomy and jejunostomy in Japan: a retrospective study with a national administrative database	DPC		Nationwide		2007	2010	Medical treatment status	Intervention effect	Diseases of the digestive system		gastrostomy and jejunostomy		
Okada 2020	Outcomes of lactulose plus branched-chain amino acid infusion and lactulose alone for hepatic encephalopathy: A retrospective cohort study using a national inpatient database	DPC		Nationwide		2011	2017	Intervention effect		Diseases of the digestive system	Certain infectious and parasitic diseases	hepatic encephalopathy (ICD-10 codes, B18–19, I85, K70–77)	Others	aged ≥20 years
Tsuchiya 2018	Mortality and Morbidity After Hartmann's Procedure Versus Primary Anastomosis Without a Diverting Stoma for Colorectal Perforation: A Nationwide Observational Study	DPC		Nationwide		2010	2014	Intervention effect		Diseases of the digestive system		colorectal perforation	Others	age of 15 years or older
Odagiri 2015	Factors associated with perforation related to diagnostic balloon-assisted enteroscopy: analysis of a national inpatient database in Japan	DPC		Nationwide		2007	2013	Clinical epidemiology, course of diseases	Intervention effect	Diseases of the digestive system	Injury, poisoning and certain other consequences of external causes	perforation related to diagnostic balloon-assisted enteroscopy		
Odagiri 2015	Hospital volume and the occurrence of bleeding and perforation after colorectal endoscopic submucosal dissection: analysis of a national administrative database in Japan	DPC		Nationwide		2012	2013	Health policy evaluation and utilization		Diseases of the digestive system	Injury, poisoning and certain other consequences of external causes	bleeding and perforation after colorectal endoscopic submucosal dissection		
Yoshihara 2021	Association between prophylactic antibiotic use for transarterial chemoembolization and occurrence of liver abscess: a retrospective cohort study	DPC		Nationwide		2010	2017	Intervention effect		Diseases of the digestive system	Others	liver abscess, transarterial chemoembolization	Others	≥18 year of age
Yatsuhashi 2020	Prevalence of ascites and its treatment in inpatients with liver cirrhosis: A cohort study using a Japanese medical claims database	MDV		Nationwide		2008	2018	Clinical epidemiology, course of diseases	Medical treatment status	Diseases of the digestive system		liver cirrhosis (K70-74), ascites	Others	aged ≥20 years
Sato 2017	The ADOPT-LC score: a novel predictive index of in-hospital mortality of cirrhotic patients following surgical procedures, based on a national survey	DPC		Nationwide		2010	2012	Prediction model	Clinical epidemiology, course of diseases	Diseases of the digestive system		cirrhotic		
Yatsuhashi 2021	Real-world hospital mortality of liver cirrhosis inpatients in Japan: a large-scale cohort study using a medical claims database: Prognosis of liver cirrhosis	MDV		Nationwide		2011	2018	Clinical epidemiology, course of diseases	Intervention effect	Diseases of the digestive system	Certain infectious and parasitic diseases	liver cirrhosis	Others	aged ≥20 years
Hanatani 2014	A detection algorithm for drug-induced liver injury in medical information databases using the Japanese diagnostic scale and its comparison with the Council for International Organizations of Medical Sciences/the Roussel Uclaf Causality Assessment Method scale	Others	MDV, medical information databases from the Hamamatsu University Hospital	Nationwide	(Hamamatsu University Hospital)	2007	2012	Research methodology		Diseases of the digestive system		Drug-induced liver injury	Others	18 years old or older
Hasegawa 2021	High Healthcare Cost Burden for Liver Cirrhosis(LC) and Hepatocellular Carcinoma(HCC) Progression within Nonalcoholic Fatty Liver Disease(NAFLD) or Nonalcoholic Steatohepatitis(NASH) Patients in Japan: a Real-world Data Study Using a Claims Database	JMDC		Nationwide		2005	2019	Health economics		Diseases of the digestive system	Neoplasms	NAFLD, NASH, liver cirrhosis, hepatocellular carcinoma	Others	aged ≥18 years
Sato 2014	Acute liver disease in Japan: a nationwide analysis of the Japanese Diagnosis Procedure Combination database	DPC		Nationwide		2007	2010	Clinical epidemiology, course of diseases		Diseases of the digestive system		acute liver disease		

Terai 2021	Comorbidities and healthcare costs and resource use of patients with nonalcoholic fatty liver disease (NAFLD) and nonalcoholic steatohepatitis (NASH) in the Japan medical data vision database	MDV		Nationwide		2011	2018	Clinical epidemiology, course of diseases	Health economics	Diseases of the digestive system		nonalcoholic fatty liver disease and nonalcoholic steatohepatitis	Others	aged ≥18 years
Takiguchi 2020	Vasodilator Therapy and Mortality in Nonocclusive Mesenteric Ischemia: A Nationwide Observational Study	DPC		Nationwide		2010	2018	Intervention effect		Diseases of the digestive system		Nonocclusive Mesenteric Ischemia (K550)		
Yoshioka 2014	Impact of hospital volume on hospital mortality, length of stay and total costs after pancreaticoduodenectomy	DPC		Nationwide		2007	2010	Health policy evaluation and utilization		Diseases of the digestive system		Pancreaticoduodenectomy		
Hayashi 2020	Clinical Effect of the Acrylonitrile-Co-Methallyl Sulfonate Surface-Treated Membrane as a Cytokine Adsorption Therapy for Sepsis due to Acute Panperitonitis: A Retrospective Cohort Study	DPC		Nationwide		2014	2017	Intervention effect		Diseases of the digestive system	Certain infectious and parasitic diseases	sepsis due to panperitonitis	Others	≥18 year of age
Fujiogi 2019	Outcomes following laparoscopic versus open surgery for pediatric inguinal hernia repair: Analysis using a national inpatient database in Japan	DPC		Nationwide		2010	2016	Intervention effect		Diseases of the digestive system		primary inguinal hernia	Children	aged 7 days to 15 years
Nakashima 2019	Laparoscopic versus open repair for inguinal hernia in children: a retrospective cohort study	JMDC		Nationwide		2005	2017	Intervention effect		Diseases of the digestive system		primary inguinal hernia	Children	younger than 15 years old
Okumura 2018	Impact of Drain Insertion After Perforated Peptic Ulcer Repair in a Japanese Nationwide Database Analysis	DPC	QIP	Nationwide		2010	2016	Intervention effect		Diseases of the digestive system		perforated gastric/duodenal ulcers		
Hern 2020	Cost-Effectiveness Analysis of Vedolizumab Compared with Other Biologics in Anti-TNF-Naive Patients with Moderate-to-Severe Ulcerative Colitis in Japan	JMDC	A systematic literature review and network meta-analysis	Nationwide		2018	2018	Health economics		Diseases of the digestive system		Ulcerative Colitis		
Okayasu 2019	Use of corticosteroids for remission induction therapy in patients with new-onset ulcerative colitis in real-world settings	JMDC		Nationwide		2008	2014	Medical treatment status	Quality of care	Diseases of the digestive system		ulcerative colitis (ICD-10 code: K51)		
Moroi 2021	The clinical practice of ulcerative colitis in elderly patients: An investigation using a nationwide database in Japan	DPC		Nationwide		2012	2018	Medical treatment status	Clinical epidemiology, course of diseases	Diseases of the digestive system		Ulcerative colitis		
Kobayashi 2020	Impact of immunomodulator use on treatment persistence in patients with ulcerative colitis: A claims database analysis	JMDC		Nationwide		2010	2016	Intervention effect	Patient health service utilization	Diseases of the digestive system		ulcerative colitis (K51)	Others	aged ≥18 years
Matsuoka 2021	Trends in Corticosteroid Prescriptions for Ulcerative Colitis and Factors Associated with Long-Term Corticosteroid Use: Analysis Using Japanese Claims Data from 2006 to 2016	JMDC		Nationwide		2006	2016	Medical treatment status	Quality of care	Diseases of the digestive system		Ulcerative Colitis		
Miyazaki 2021	Real-World Prescription Pattern and Healthcare Cost Among Patients with Ulcerative Colitis in Japan: A Retrospective Claims Data Analysis	JMDC		Nationwide		2009	2018	Medical treatment status	Health economics	Diseases of the digestive system		Ulcerative Colitis		
Sato 2015	Variceal hemorrhage: Analysis of 9987 cases from a Japanese nationwide database	DPC		Nationwide		2007	2012	Clinical epidemiology, course of diseases		Diseases of the digestive system	Diseases of the circulatory system	variceal hemorrhage		
12) Diseases of the skin and subcutaneous tissue (L00-L99)														
Kawahara 2019	[Relationship between Treatment for Allergic Diseases and Medical Expenses: The Influence That Treatment Difference of Atopic Dermatitis of Dermatologist and Pediatrician Gives to Medical Expenses]	JMDC		Nationwide		2010	2011	Medical treatment status	Health economics	Diseases of the skin and subcutaneous tissue		atopic dermatitis	Children	aged <15 years
Komura 2018	Economic assessment of actual prescription of drugs for treatment of atopic dermatitis: Differences between dermatology and pediatrics in large-scale receipt data	JMDC		Nationwide		2010	2011	Medical treatment status	Health economics	Diseases of the skin and subcutaneous tissue		atopic dermatitis	Children	aged 0–14 years
Igarashi 2019	Health-care resource use and current treatment of adult atopic dermatitis patients in Japan: A retrospective claims database analysis	JMDC		Nationwide		2013	2016	Medical treatment status	Health economics	Diseases of the skin and subcutaneous tissue		atopic dermatitis	Others	15-60 year of age
Sugimoto 2018	Contact Dermatitis after Prescription of an Ophthalmic Ointment Containing Fradiomycin Sulfate: A Retrospective Database Study Using Japanese Health Insurance Claims Data	Others	MinaCare HDB	Nationwide		2013	2013	Intervention effect		Diseases of the skin and subcutaneous tissue		contact dermatitis		age group
Terui 2018	[Analysis of Real World Data on Epidemiological Characteristics of Hidradenitis Suppurativa in Japan]	JMDC		Nationwide		2015	2016	Clinical epidemiology, course of diseases	Medical treatment status	Diseases of the skin and subcutaneous tissue		hidradenitis suppurativa	Others	aged 15–64 years
Suwanai 2020	Dipeptidyl Peptidase-4 Inhibitor Reduces the Risk of Developing Hypertrophic Scars and Keloids following Median Sternotomy in Diabetic Patients: A Nationwide Retrospective Cohort Study Using the National Database of Health Insurance Claims of Japan	NDB		Nationwide		2013	2015	Intervention effect		Diseases of the skin and subcutaneous tissue		hypertrophic scars or keloids		
Kubota 2015	Epidemiology of psoriasis and palmoplantar pustulosis: a nationwide study using the Japanese national claims database	NDB		Nationwide		2010	2011	Clinical epidemiology, course of diseases		Diseases of the skin and subcutaneous tissue		psoriasis and palmoplantar pustulosis		age group
Miyazaki 2020	Treatment patterns and healthcare resource utilization in palmoplantar pustulosis patients in Japan: A claims database study	JMDC		Nationwide		2011	2017	Medical treatment status	Health economics	Diseases of the skin and subcutaneous tissue		Palmoplantar pustulosis (PPP)	Others	age ≥18 years
Nakagami 2020	Association between pressure injury status and hospital discharge to home: a retrospective observational cohort study using a national inpatient database	DPC		Nationwide		2014	2014	Clinical epidemiology, course of diseases	Health policy evaluation and utilization	Diseases of the skin and subcutaneous tissue		pressure injury	Older persons	aged 65 years or older
Sruamsiri 2018	Persistence rates and medical costs of biological therapies for psoriasis treatment in Japan: a real-world data study using a claims database	JMDC		Nationwide		2009	2016	Patient health service utilization	Health economics	Diseases of the skin and subcutaneous tissue		psoriasis (L40)		
Inui 2021	Treatment practices and costs among patients with psoriatic arthritis: A Japanese hospital claims database analysis	MDV		Nationwide		2009	2017	Medical treatment status	Health economics	Diseases of the skin and subcutaneous tissue		psoriatic arthritis (L40.50, L40.51, L40.52, L40.53, L40.54, and L40.59)		
Terui 2014	[A survey of the status of psoriasis conducted using information obtained from health insurance claims provided by health insurance societies]	JMDC		Nationwide		2011	2012	Clinical epidemiology, course of diseases	Medical treatment status	Diseases of the skin and subcutaneous tissue		psoriasi		
Makino 2015	[Identifying the Pattern of Physician Consultation for Skin Diseases in Kumamoto Prefecture, from the National Health Insurance Claims, during May of 2009]	NHI/LSEHS	NHI	Prefecture	Kumamoto	2009	2009	Patient health service utilization	Socioeconomic comparison	Diseases of the skin and subcutaneous tissue		Skin diseases		
Sato 2020	Real-world evidence of population differences in allopurinol-related severe cutaneous adverse reactions in East Asians: A population-based cohort study	JMDC		Nationwide	Taiwan, South Korea, Japan	2005	2016	Intervention effect	Clinical epidemiology, course of diseases	Diseases of the skin and subcutaneous tissue		alloprinol-related severe cutaneous adverse reactions		
Fukasawa 2021	Risk of Stevens-Johnson syndrome and toxic epidermal necrolysis associated with anticonvulsants in a Japanese population: Matched case-control and cohort studies	JMDC		Nationwide		2005	2017	Intervention effect		Diseases of the skin and subcutaneous tissue		anticonvulsant-induced Stevens-Johnson syndrome and toxic epidermal necrolysis		
Morita 2019	Association of Early Systemic Corticosteroid Therapy with Mortality in Patients with Stevens-Johnson Syndrome or Toxic Epidermal Necrolysis: A Retrospective Cohort Study Using a Nationwide Claims Database	DPC		Nationwide		2010	2014	Intervention effect		Diseases of the skin and subcutaneous tissue		Steven-Johnson syndrome, toxic epidermal necrolysis (ICD-10 codes L511–L512)	Others	aged ≥18 years
13) Diseases of the musculoskeletal system and connective tissue (M00-M99)														
Kawata 2018	Atopic dermatitis is a novel demographic risk factor for surgical site infection after anterior cruciate ligament reconstruction	DPC		Nationwide		2010	2015	Clinical epidemiology, course of diseases		Diseases of the musculoskeletal system and connective tissue	Injury, poisoning and certain other consequences of external causes	anterior cruciate ligament reconstruction, surgical site infection		age categories
Sakai 2019	Risk of opportunistic infections in patients with antineutrophil cytoplasmic antibody-associated vasculitis, using a Japanese health insurance database	MDV		Nationwide		2008	2017	Clinical epidemiology, course of diseases		Diseases of the musculoskeletal system and connective tissue		antineutrophil cytoplasmic antibody-associated vasculitis		

Uechi 2018	Effect of plasma exchange on in-hospital mortality in patients with pulmonary hemorrhage secondary to antineutrophil cytoplasmic antibody-associated vasculitis: A propensity-matched analysis using a nationwide administrative database	DPC		Nationwide		2009	2014	Intervention effect		Diseases of the musculoskeletal system and connective tissue	Others	Antineutrophil cytoplasmic antibody-associated vasculitis, secondary pulmonary hemorrhage		
Kumamaru 2019	Large hospital variation in the utilization of Post-procedural CT to detect pulmonary embolism/Deep Vein Thrombosis in Patients Undergoing Total Knee or Hip Replacement Surgery: Japanese Nationwide Diagnosis Procedure Combination Database Study	DPC		Nationwide		2012	2013	Medical treatment status	Health policy evaluation and utilization	Diseases of the musculoskeletal system and connective tissue	Diseases of the circulatory system	venous thromboembolism, total knee or hip replacement	Others	aged ≥40 years
Nagase 2011	Risk factors for pulmonary embolism and the effects of fondaparinux after total hip and knee arthroplasty: a retrospective observational study with use of a national database in Japan	DPC		Nationwide		2007	2008	Clinical epidemiology, course of diseases	Intervention effect	Diseases of the musculoskeletal system and connective tissue	Diseases of the circulatory system	total hip and knee arthroplasty, pulmonarry embolism		
Matsumoto 2016	Time trends and risk factors for perioperative complications in total ankle arthroplasty: retrospective analysis using a national database in Japan	DPC		Nationwide		2007	2013	Clinical epidemiology, course of diseases	Medical treatment status	Diseases of the musculoskeletal system and connective tissue	Injury, poisoning and certain other consequences of external causes	Total ankle arthroplasty, end-stage ankle arthritis		
Yamagami 2021	Unicompartmental knee arthroplasty is associated with lower proportions of surgical site infection compared with total knee arthroplasty: A retrospective nationwide database study	DPC		Nationwide		2010	2017	Clinical epidemiology, course of diseases	Intervention effect	Diseases of the musculoskeletal system and connective tissue	Injury, poisoning and certain other consequences of external causes	surgical site infection, Knee arthroplasty	Others	16-102 year of age
Morishita 2020	Comparison of Perioperative Complications Between Anterior Decompression With Fusion and Laminoplasty For Cervical Spondylotic Myelopathy: Propensity Score-matching Analysis Using Japanese Diagnosis Procedure Combination Database	DPC		Nationwide		2010	2016	Intervention effect	Health economics	Diseases of the musculoskeletal system and connective tissue		cervical spondylotic myelopathy		
Sumitani 2014	Perioperative factors affecting the occurrence of acute complex regional pain syndrome following limb bone fracture surgery: data from the Japanese Diagnosis Procedure Combination database	DPC		Nationwide		2007	2010	Clinical epidemiology, course of diseases		Diseases of the musculoskeletal system and connective tissue	Injury, poisoning and certain other consequences of external causes	Complex regional pain syndrome, fractures		
Ishikawa 2021	Estimation of treatment and prognostic factors of pneumocystis pneumonia in patients with connective tissue diseases	DPC		Nationwide		2014	2016	Clinical epidemiology, course of diseases		Diseases of the musculoskeletal system and connective tissue	Certain infectious and parasitic diseases	connective tissue disease- associated pneumocystis pneumonia		
Hasegawa 2015	Factors that predict in-hospital mortality in eosinophilic granulomatosis with polyangiitis	DPC		Nationwide		2010	2013	Clinical epidemiology, course of diseases		Diseases of the musculoskeletal system and connective tissue		Eosinophilic granulomatosis with polyangiitis (ICD-10 code, M301)		
Sugihara 2012	Impact of surgical intervention timing on the case fatality rate for Fournier's gangrene: an analysis of 379 cases	DPC		Nationwide		2007	2010	Intervention effect		Diseases of the musculoskeletal system and connective tissue		Fournier's gangrene (ICD-10 code, M72.6)		
Ohara 2020	Fracture risk increased by concurrent use of central nervous system agents in older people: Nationwide case-crossover study	NDB		Nationwide		2013	2014	Intervention effect		Diseases of the musculoskeletal system and connective tissue	Injury, poisoning and certain other consequences of external causes	fragility fracture	Older persons	aged ≥65 years
Ohara 2020	Central Nervous System Agent Classes and Fragility Fracture Risk among Elderly Japanese Individuals in a Nationwide Case-Crossover Design Study	NDB		Nationwide		2013	2014	Intervention effect		Diseases of the musculoskeletal system and connective tissue	Injury, poisoning and certain other consequences of external causes	fragility fracture	Older persons	aged ≥65 years
Iihara 2019	Fragility Fractures in Older People in Japan Based on the National Health Insurance Claims Database	NDB		Nationwide		2013	2014	Clinical epidemiology, course of diseases		Diseases of the musculoskeletal system and connective tissue	Injury, poisoning and certain other consequences of external causes	Fragility Fractures	Older persons	aged 65 years or older
Hakoda 2020	[Future trends for the number of gout patients in Japan]	JMDC	Comprehensive Survey of Living Conditions (MHLW)	Nationwide		2010	2014	Clinical epidemiology, course of diseases		Diseases of the musculoskeletal system and connective tissue		gout patients		
Koto 2021	Factors associated with achieving target serum uric acid level and occurrence of gouty arthritis: A retrospective observational study of Japanese health insurance claims data	JMDC		Nationwide		2015	2017	Clinical epidemiology, course of diseases	Medical treatment status	Diseases of the musculoskeletal system and connective tissue		gouty arthritis	Others	18-65 years old
Kawata 2021	Type of bone graft and primary diagnosis were associated with nosocomial surgical site infection after high tibial osteotomy: analysis of a national database	DPC		Nationwide		2010	2017	Clinical epidemiology, course of diseases		Diseases of the musculoskeletal system and connective tissue	Injury, poisoning and certain other consequences of external causes	surgical site infection after high tibial osteotomy		
Okubo 2020	The Impact of Changes in Clinical Guideline on Practice Patterns and Healthcare Utilizations for Kawasaki Disease in Japan	DPC		Nationwide		2010	2015	Medical treatment status	Health policy evaluation and utilization	Diseases of the musculoskeletal system and connective tissue		Kawasaki disease (ICD-10 code: M30.3)	Children	aged under 18 years
Okubo 2018	Association Between Dose of Glucocorticoids and Coronary Artery Lesions in Kawasaki Disease	DPC		Nationwide		2010	2015	Intervention effect		Diseases of the musculoskeletal system and connective tissue		Kawasaki Disease (M30.3)	Children	aged <18 years
Takeuchi 2018	Changes in Hemoglobin Concentrations Post-immunoglobulin Therapy in Patients with Kawasaki Disease: A Population-Based Study Using a Claims Database in Japan	MDV		Nationwide		2010	2016	Intervention effect		Diseases of the musculoskeletal system and connective tissue	Diseases of the blood and blood-forming organs and certain disorders involving the immune mechanism	Kawasaki Disease (M30.3), anemia	Children	aged ≤18 years
Kumamaru 2018	Radiologist involvement is associated with reduced use of MRI in the acute period of low back pain in a non-elderly population	Others	MinaCare	Nationwide		2013	2015	Medical treatment status	Health policy evaluation and utilization	Diseases of the musculoskeletal system and connective tissue		low-back pain	Others	aged 18-64 years
Ohya 2016	Does the microendoscopic technique reduce mortality and major complications in patients undergoing lumbar discectomy? A propensity score-matched analysis using a nationwide administrative database	DPC		Nationwide		2010	2013	Intervention effect		Diseases of the musculoskeletal system and connective tissue		lumbar discectomy		
Kawata 2018	Annual trends in arthroscopic meniscus surgery: Analysis of a national database in Japan	DPC		Nationwide		2007	2015	Medical treatment status		Diseases of the musculoskeletal system and connective tissue		meniscus surgery		
Chang 2013	Assessment of statin-associated muscle toxicity in Japan: a cohort study conducted using claims database and laboratory information	MDV		Nationwide		2004	2010	Intervention effect		Diseases of the musculoskeletal system and connective tissue		muscle toxicity	Others	aged 18 or above
Uehara 2014	Necrotising soft-tissue infections of the upper limb: risk factors for amputation and death	DPC		Nationwide		2007	2010	Clinical epidemiology, course of diseases		Diseases of the musculoskeletal system and connective tissue		Necrotising soft-tissue infections (M72.6)		
Suzuki 2021	Factors associated with mortality among patients with necrotizing soft tissue infections: An analysis of 4597 cases using the Diagnosis Procedure Combination Database	DPC		Nationwide		2014	2017	Clinical epidemiology, course of diseases		Diseases of the musculoskeletal system and connective tissue		necrotizing soft-tissue infection		
Ebata-Kogure 2020	Treatment and Healthcare Cost Among Patients with Hip or Knee Osteoarthritis: A Cross-sectional Study Using a Real-world Claims Database in Japan Between 2013 and 2019	MDV		Nationwide		2013	2019	Medical treatment status	Health economics	Diseases of the musculoskeletal system and connective tissue		hip or knee osteoarthritis	Others	aged ≥18 years
Ueda 2020	Long-Term Pain Management and Health Care Resource Use Among an Employed Population in Japan with Knee Osteoarthritis Combined with Low Back Pain	JMDC		Nationwide		2011	2012	Health economics	Medical treatment status	Diseases of the musculoskeletal system and connective tissue		knee osteoarthritis (ICD-10-CM code M17)	Others	more than 40 years of age
Ueda 2020	Longitudinal Assessment of Pain Management Among the Employed Japanese Population with Knee Osteoarthritis	JMDC		Nationwide		2011	2012	Clinical epidemiology, course of diseases	Medical treatment status	Diseases of the musculoskeletal system and connective tissue		knee osteoarthritis (ICD-10-CM code M17)	Others	aged over 40 years
Sakai 2019	International descriptive study for comparison of treatment patterns in patients with knee osteoarthritis between Korea and Japan using claims data	JMDC		Nationwide	Korea and Japan	2012	2013	Medical treatment status	Socioeconomic comparison	Diseases of the musculoskeletal system and connective tissue		knee osteoarthritis	Others	aged ≥50 years
Akazawa 2019	Patterns of drug treatment in patients with osteoarthritis and chronic low back pain in Japan: a retrospective database study	MDV		Nationwide		2013	2017	Medical treatment status	Clinical epidemiology, course of diseases	Diseases of the musculoskeletal system and connective tissue		osteoarthritis /chronic low back pain	Others	≥18 year of age
Kawata 2017	Annual trends in knee arthroplasty and tibial osteotomy: Analysis of a national database in Japan	DPC		Nationwide		2007	2015	Medical treatment status		Diseases of the musculoskeletal system and connective tissue		Osteoarthritis, osteonecrosis, rheumatoid arthritis		age groups
Kikuchi 2021	A Retrospective Database Study of Gastrointestinal Events and Medical Costs Associated with Nonsteroidal Anti-Inflammatory Drugs in Japanese Patients of Working Age with Osteoarthritis and Chronic Low Back Pain	JMDC		Nationwide		2009	2018	Intervention effect	Health economics	Diseases of the musculoskeletal system and connective tissue	Diseases of the digestive system	osteoarthritis, chronic low back pain, gastrointestinal events	Others	aged ≥18 years

Saka 2019	Coding and prescription rates of osteoporosis are low among distal radius fracture patients in Japan	JMDC		Nationwide		2014	2015	Medical treatment status	Quality of care	Diseases of the musculoskeletal system and connective tissue		osteoporosis (ICD10: M819), distal radius fracture (S525, S526)	Others	aged 50–75 years
Tanaka 2018	Real-world evidence of raloxifene versus alendronate in preventing non-vertebral fractures in Japanese women with osteoporosis: retrospective analysis of a hospital claims database	MDV		Nationwide		2008	2013	Intervention effect		Diseases of the musculoskeletal system and connective tissue		osteoporosis, non-vertebral fractures	Others	aged 50 years or older
Usui 2018	Persistence of and switches from teriparatide treatment among women and men with osteoporosis in the real world: a claims database analysis	JMDC		Nationwide		2005	2017	Medical treatment status	Patient health service utilization	Diseases of the musculoskeletal system and connective tissue		osteoporosis (ICD-10 codes: M80 or M81)		
Burge 2016	Real-world clinical and economic outcomes for daily teriparatide patients in Japan	MDV		Nationwide		2008	2013	Medical treatment status	Health economics	Diseases of the musculoskeletal system and connective tissue		osteoporosis		
Fujimori 2019	[Analysis of the persistence and compliance of switching medications for osteoporosis using E-claim database]	NHI/LSEHS	National Health Insurance and the late-stage medical care system	Prefecture	Hokkaido	2014	2016	Medical treatment status	Patient health service utilization	Diseases of the musculoskeletal system and connective tissue		osteoporosis	Others	aged 50 years or older
Fujimori 2019	[Analysis of the persistence and compliance of medications for osteoporosis using E-claim database]	NHI/LSEHS	National Health Insurance and the late-stage medical care system	Prefecture	Hokkaido	2014	2015	Patient health service utilization		Diseases of the musculoskeletal system and connective tissue		osteoporosis	Others	aged 50 years or older
Yamanaka 2019	[What is the current state of osteoporosis in terms of medical costs? Considering the current state of osteoporosis in communities from the National Health Insurance data system]*	NHI/LSEHS	long-term care insurance data	Municipality	Higashihiroshima city	2016	2016	Clinical epidemiology, course of diseases		Diseases of the musculoskeletal system and connective tissue		Osteoporosis		
Yokoyama 2020	Association between oral anticoagulants and osteoporosis: Real-world data mining using a multi-methodological approach	JMDC	FAERS	Nationwide		2005	2017	Intervention effect		Diseases of the musculoskeletal system and connective tissue	Others	osteoporosis and antimicrobial use		
Taguchi 2018	Treatment costs and cost drivers among osteoporotic fracture patients in Japan: a retrospective database analysis	MDV		Nationwide		2008	2017	Health economics		Diseases of the musculoskeletal system and connective tissue	Injury, poisoning and certain other consequences of external causes	osteoporotic fracture	Others	50 years and older
Morishita 2020	Risk factors related to perioperative systemic complications and mortality in elderly patients with osteoporotic vertebral fractures-analysis of a large national inpatient database	DPC		Nationwide		2012	2016	Clinical epidemiology, course of diseases		Diseases of the musculoskeletal system and connective tissue	Injury, poisoning and certain other consequences of external causes	osteoporotic vertebral fractures	Older persons	over 65 years old
Fujimori 2020	[Association of the medications for osteoporosis and the hip fracture using E-claim database]	NHI/LSEHS	The National Health Insurance system and Late Elders' Health Insurance	Prefecture	Hokkaido	2014	2018	Intervention effect		Diseases of the musculoskeletal system and connective tissue	Injury, poisoning and certain other consequences of external causes	osteoporosis, hip fracture	Older persons	60 years old and older
Kirigaya 2011	Management and treatment of osteoporosis in patients receiving long-term glucocorticoid treatment: current status of adherence to clinical guidelines and related factors	JMDC		Nationwide		2004	2007	Quality of care		Diseases of the musculoskeletal system and connective tissue	Endocrine, nutritional and metabolic diseases	Osteoporosis, glucocorticoid treatment	Others	aged ≥18 years
Shiragami 2014	[Cost effectiveness of using medical topical adhesive patches for peritendinitis patients]	Others	JammNet	Nationwide		2010	2012	Health economics		Diseases of the musculoskeletal system and connective tissue		peri tendinitis		
Morishita 2019	Perioperative complications of anterior decompression with fusion versus laminoplasty for the treatment of cervical ossification of the posterior longitudinal ligament: propensity score matching analysis using a nation-wide inpatient database	DPC		Nationwide		2010	2016	Intervention effect		Diseases of the musculoskeletal system and connective tissue	Injury, poisoning and certain other consequences of external causes	Perioperative complications, cervical ossification of the posterior longitudinal ligament		
Sruamsiri 2018	Persistence with Biological Disease-modifying Antirheumatic Drugs and Its Associated Resource Utilization and Costs	MDV		Nationwide		2009	2015	Patient health service utilization	Health economics	Diseases of the musculoskeletal system and connective tissue		rheumatoid arthritis	Others	aged ≥18 years
Sugiyama 2016	Treatment Patterns, Direct Cost of Biologics, and Direct Medical Costs for Rheumatoid Arthritis Patients: A Real-world Analysis of Nationwide Japanese Claims Data	JMDC		Nationwide		2005	2014	Medical treatment status	Health economics	Diseases of the musculoskeletal system and connective tissue		Rheumatoid arthritis	Others	aged 16 to <75 years
Nakajima 2021	Geographic variations in rheumatoid arthritis treatment in Japan: A nationwide retrospective study using the national database of health insurance claims and specific health checkups of Japan	NDB		Nationwide		2017	2018	Medical treatment status	Socioeconomic comparison	Diseases of the musculoskeletal system and connective tissue		rheumatoid arthritis	Others	16 years old or older
Lane 2020	Risk of hydroxychloroquine alone and in combination with azithromycin in the treatment of rheumatoid arthritis: a multinational, retrospective study	JMDC	Data comprised 14 sources of claims data or electronic medical records from Germany, Japan, the Netherlands, Spain, the UK, and the USA.	Nationwide		2000	2020	Intervention effect		Diseases of the musculoskeletal system and connective tissue		rheumatoid arthritis	Others	18 years or older
Nakajima 2020	Prevalence of patients with rheumatoid arthritis and age-stratified trends in clinical characteristics and treatment, based on the National Database of Health Insurance Claims and Specific Health Checkups of Japan	NDB		Nationwide		2017	2018	Clinical epidemiology, course of diseases	Medical treatment status	Diseases of the musculoskeletal system and connective tissue		rheumatoid arthritis	Others	aged ≥16 years
Yamairi 2020	Dose Adjustment of Methotrexate Administered Concomitantly with Golimumab for Rheumatoid Arthritis in Japanese Real-World Clinical Settings	JMDC		Nationwide		2005	2018	Intervention effect		Diseases of the musculoskeletal system and connective tissue		Rheumatoid arthritis (M05, M06, and M080)		
Kasai 2019	Higher risk of hospitalized infection, cardiovascular disease, and fracture in patients with rheumatoid arthritis determined using the Japanese health insurance database	JMDC		Nationwide		2005	2014	Clinical epidemiology, course of diseases		Diseases of the musculoskeletal system and connective tissue		Rheumatoid arthritis	Others	aged ≥18 years
Takabayashi 2019	Comparing the effectiveness of biological disease-modifying antirheumatic drugs using real-world data	Others	13 Japanese national university hospitals' claims data (Clinical Information Statistical Analysis (CISA))	Nationwide	13 Japanese national university hospitals	2005	2016	Intervention effect		Diseases of the musculoskeletal system and connective tissue		rheumatoid arthritis		
Mahlich 2017	Treatment patterns of rheumatoid arthritis in Japanese hospitals and predictors of the initiation of biologic agents	MDV		Nationwide		2012	2014	Medical treatment status		Diseases of the musculoskeletal system and connective tissue		rheumatoid arthritis		age distribution
Mahlich 2016	Persistence with biologic agents for the treatment of rheumatoid arthritis in Japan	MDV		Nationwide		2012	2014	Patient health service utilization		Diseases of the musculoskeletal system and connective tissue		rheumatoid arthritis		age distribution
Katada 2014	Prescription patterns and trends in anti-rheumatic drug use based on a large-scale claims database in Japan	JMDC		Nationwide		2005	2011	Medical treatment status	Quality of care	Diseases of the musculoskeletal system and connective tissue		rheumatoid arthritis		
Yamanaka 2014	Estimates of the prevalence of and current treatment practices for rheumatoid arthritis in Japan using reimbursement data from health insurance societies and the IORRA cohort (I)	JMDC		Nationwide		2005	2010	Clinical epidemiology, course of diseases	Medical treatment status	Diseases of the musculoskeletal system and connective tissue		rheumatoid arthritis	Others	aged ≥16 to <75 years

Sakai 2016	High prevalence of cardiovascular comorbidities in patients with rheumatoid arthritis from a population-based cross-sectional study of a Japanese health insurance database	JMDC		Nationwide		2010	2012	Clinical epidemiology, course of diseases		Diseases of the musculoskeletal system and connective tissue	Diseases of the circulatory system	rheumatoid arthritis (RA) and cardiovascular comorbidities		age group
Tanaka 2016	Risk for cardiovascular disease in Japanese patients with rheumatoid arthritis: a large-scale epidemiological study using a healthcare database	MDV		Nationwide		2011	2014	Clinical epidemiology, course of diseases		Diseases of the musculoskeletal system and connective tissue	Diseases of the circulatory system	rheumatoid arthritis, risk for cardiovascular disease	Others	aged 18 years or older
Taniguchi 2018	In-hospital mortality and morbidity of pediatric scoliosis surgery in Japan: Analysis using a national inpatient database	DPC		Nationwide		2010	2013	Clinical epidemiology, course of diseases	Intervention effect	Diseases of the musculoskeletal system and connective tissue		pediatric scoliosis	Children	less than 19 years
Oichi 2018	In-hospital complication rate following microendoscopic versus open lumbar laminectomy: a propensity score-matched analysis	DPC		Nationwide		2010	2013	Intervention effect		Diseases of the musculoskeletal system and connective tissue		Lumbar spinal stenosis	Others	patients aged ≥20 y
Kato 2016	Risk of infectious complications associated with blood transfusion in elective spinal surgery-a propensity score matched analysis	DPC		Nationwide		2007	2012	Intervention effect		Diseases of the musculoskeletal system and connective tissue		spinal stenosis, spondylolis- thesis	Others	aged ≥20 years
Kuboyama 2016	The Number of Patients and Therapeutic Profile of Spinal Stenosis Using Health Insurance Claims in Japan	NHI/LSEHS	National Health Insurance (NHI) and Late-stage Elderly Health Insurance (LEHI)	Prefecture	a prefecture in south-western Japan	2010	2011	Clinical epidemiology, course of diseases	Medical treatment status	Diseases of the musculoskeletal system and connective tissue		spinal stenosis		
Oichi 2019	[Can elective spine surgery be performed safely among nonagenarians? : Analysis of a national inpatient database in Japan]	DPC		Nationwide		2010	2013	Intervention effect		Diseases of the musculoskeletal system and connective tissue		spine surgery	Older persons	aged 65 years or older
Oichi 2019	Can Elective Spine Surgery Be Performed Safely Among Nonagenarians?: Analysis of a National Inpatient Database in Japan	DPC		Nationwide		2010	2013	Clinical epidemiology, course of diseases	Intervention effect	Diseases of the musculoskeletal system and connective tissue		surgical outcomes, underwent elective spine surgery	Older persons	≥65 years of age
Ohya 2015	Perioperative stroke in patients undergoing elective spinal surgery: a retrospective analysis using the Japanese diagnosis procedure combination database	DPC		Nationwide		2007	2012	Intervention effect		Diseases of the musculoskeletal system and connective tissue	Diseases of the circulatory system	perioperative stroke in patients undergoing elective spinal surgery	Others	aged 20 years or older
Masuda 2012	Factors affecting the occurrence of pulmonary embolism after spinal surgery: data from the national administrative database in Japan	DPC		Nationwide		2007	2008	Clinical epidemiology, course of diseases	Intervention effect	Diseases of the musculoskeletal system and connective tissue	Diseases of the circulatory system	pulmonary embolism after spinal surgery		
Chikuda 2012	Mortality and morbidity in dialysis-dependent patients undergoing spinal surgery: analysis of a national administrative database in Japan	DPC		Nationwide		2007	2008	Clinical epidemiology, course of diseases	Intervention effect	Diseases of the musculoskeletal system and connective tissue	Diseases of the genitourinary system	dialysis-dependent patients treated with spinal surgery		
Tomita 2021	Treatment patterns and health care resource utilization among Japanese patients with ankylosing spondylitis: A hospital claims database analysis	MDV		Nationwide		2009	2017	Medical treatment status	Health economics	Diseases of the musculoskeletal system and connective tissue		ankylosing spondylitis (ICD10 codes; M08.1, M45.0, M45.4, M45.9)		
Miyazaki 2020	Treatment patterns and medical cost of systemic lupus erythematosus patients in Japan: a retrospective claims database study	JMDC		Nationwide		2010	2017	Medical treatment status	Health economics	Diseases of the musculoskeletal system and connective tissue		systemic lupus erythematosus (M321)	Others	aged ≥18 years
Tanaka 2018	Disease severity and economic burden in Japanese patients with systemic lupus erythematosus: A retrospective, observational study	JMDC		Nationwide		2010	2012	Health economics		Diseases of the musculoskeletal system and connective tissue		systemic lupus erythematosus (M32)	Others	15 to 65 years of age
Sakai 2020	The risk of hospitalized infection in patients with systemic lupus erythematosus treated with hydroxychloroquine	MDV		Nationwide		2015	2017	Intervention effect		Diseases of the musculoskeletal system and connective tissue		systemic lupus erythematosus (M321 or M329)	Others	≥16 years of age
Akiyama 2013	Incidence and risk factors for mortality of vertebral osteomyelitis: a retrospective analysis using the Japanese diagnosis procedure combination database	DPC		Nationwide		2007	2010	Clinical epidemiology, course of diseases		Diseases of the musculoskeletal system and connective tissue		vertebral osteomyelitis		
14) Diseases of the genitourinary system (N00-N99)														
Ikuta 2021	Association of proton pump inhibitors and concomitant drugs with risk of acute kidney injury: a nested case-control study	JMDC		Nationwide		2005	2017	Intervention effect		Diseases of the genitourinary system		Acute kidney injury (AKI; N17X)		
Iwagami 2015	Current state of continuous renal replacement therapy for acute kidney injury in Japanese intensive care units in 2011: analysis of a national administrative database	DPC		Nationwide		2011	2011	Medical treatment status	Clinical epidemiology, course of diseases	Diseases of the genitourinary system		acute kidney injury in ICU	Others	aged ≥12 years
Miyamoto 2019	Association between intravenous contrast media exposure and non-recovery from dialysis-requiring septic acute kidney injury: a nationwide observational study	DPC		Nationwide		2011	2016	Intervention effect		Diseases of the genitourinary system		septic acute kidney injury	Others	aged ≥18 years
Takazono 2020	Factor analysis of acute kidney injury in patients administered liposomal amphotericin B in a real-world clinical setting in Japan	MDV		Nationwide		2008	2018	Intervention effect		Diseases of the genitourinary system	Others	broad-spectrum antifungal drug, acute kidney injury	Others	≥18 year of age
Iwagami 2015	[Comparison of characteristics and prognosis between septic and non-septic acute kidney injury in Japanese intensive care units]	DPC		Nationwide		2011	2011	Clinical epidemiology, course of diseases		Diseases of the genitourinary system	Certain infectious and parasitic diseases	septic and non-septic acute kidney injury		
Imai 2020	A cross-sectional exploratory survey on occurrence of triple-whammy prescription pattern in Japan	JMDC		Nationwide		2017	2017	Medical treatment status		Diseases of the genitourinary system		chronic kidney disease		
Nagai 2019	Higher medical costs for CKD patients with a rapid decline in eGFR: A cohort study from the Japanese general population	NHI/LSEHS	KDB	Nationwide		2012	2013	Health economics		Diseases of the genitourinary system		chronic kidney disease	Others	aged 40-74 years
Takada 2021	Stages of a transtheoretical model as predictors for the decline of estimated glomerular filtration rate: a retrospective cohort study	JHIA	Japan Health Insurance Association in Kyoto prefecture	Prefecture	Kyoto	2012	2016	Clinical epidemiology, course of diseases		Diseases of the genitourinary system		kidney disease (estimated glomerular filtration rate)	Others	between 35 and 75 years old
Fukuma 2020	Quality of Care in Chronic Kidney Disease and Incidence of End-stage Renal Disease in Older Patients: A Cohort Study	NDB		Nationwide		2010	2014	Quality of care	Intervention effect	Diseases of the genitourinary system		chronic kidney disease, end-stage renal disease	Older persons	≥65 years of age
Kikuchi 2018	Short-term prognosis of emergently hospitalized dialysis-independent chronic kidney disease patients: A nationwide retrospective cohort study in Japan	DPC		Nationwide		2013	2016	Clinical epidemiology, course of diseases		Diseases of the genitourinary system		chronic kidney disease	Others	20-69 years old
Hagiwara 2017	The Survey of the Compliance Situation to the Antihypertensive Therapy Guideline by Analyzing Japanese National Claims Data	NDB	Sampling data	Nationwide		2011	2011	Medical treatment status	Quality of care	Diseases of the genitourinary system		kidney disease		
Imai 2020	Nonsteroidal anti-inflammatory drugs use in patients with chronic kidney disease are often prescribed from different clinicians than those who diagnosed them	JMDC		Nationwide		2014	2017	Medical treatment status		Diseases of the genitourinary system		chronic kidney disease, NSAIDs	Others	aged ≥20 years
Takada 2019	Previously-initiated hemodialysis as prognostic factor for in-hospital mortality in pneumonia patients with stage 5 chronic kidney disease: Retrospective database study of Japanese hospitals	DPC		Nationwide		2012	2016	Clinical epidemiology, course of diseases		Diseases of the genitourinary system		chronic kidney disease	Others	aged ≥18 years
Takada 2020	Developing and validating a multivariable prediction model for in-hospital mortality of pneumonia with advanced chronic kidney disease patients: a retrospective analysis using a nationwide database in Japan	DPC		Nationwide		2012	2016	Prediction model	Clinical epidemiology, course of diseases	Diseases of the genitourinary system		chronic kidney disease	Others	aged from 18 to 94 years
Kimura 2020	Diagnosis Patterns of CKD and Anemia in the Japanese Population	Others	JMDC, MDV	Nationwide		2008	2017	Medical treatment status	Quality of care	Diseases of the genitourinary system	Diseases of the blood and blood-forming organs and certain disorders involving the immune mechanism	anemia, chronic kidney disease	Others	aged ≥18 years
Tohkin 2016	[Appropriate prescription pattern of medications at the special population]	NDB	Sampling data	Nationwide		2011	2011	Medical treatment status	Quality of care	Diseases of the genitourinary system	Diseases of the circulatory system	chronic kidney disease, cardiovascular disease		
Mandai 2020	Nationwide in-hospital mortality following major fractures among hemodialysis patients and the general population: An observational cohort study	DPC		Nationwide		2012	2014	Clinical epidemiology, course of diseases		Diseases of the genitourinary system	Injury, poisoning and certain other consequences of external causes	End-stage kidney disease, major fracture	Others	aged ≥18 years

Kohsaka 2020	Cardiovascular and Renal Outcomes Associated With Hyperkalemia in Chronic Kidney Disease: A Hospital-Based Cohort Study	MDV		Nationwide		2008	2018	Clinical epidemiology, course of diseases		Diseases of the genitourinary system	Endocrine, nutritional and metabolic diseases	CKD, hyperckalemia	Others	aged ≥18 years
Kusama 2021	Epidemiology of antibiotic treatment for uncomplicated cystitis in adults in Japan	JMDC		Nationwide		2013	2016	Medical treatment status		Diseases of the genitourinary system		acute cystitis	Others	aged ≥15 years
Sugihara 2012	Admissions related to interstitial cystitis in Japan: an estimation based on the Japanese diagnosis procedure combination database	DPC		Nationwide		2007	2009	Clinical epidemiology, course of diseases		Diseases of the genitourinary system		interstitial cystitis (N301)		
Onoue 2016	[Evaluation of regional differences in health care costs for patients undergoing hemodialysis within the Fukuoka Prefecture Wide-Area Association of Latter-Stage Elderly Healthcare]	NHI/LSEHS	Fukuoka Prefecture Wide-Area Association of Latter-Stage Elderly Healthcare data	Prefecture	Fukuoka	2013	2014	Health economics	Socioeconomic comparison	Diseases of the genitourinary system		hemodialysis	Older persons	Latter-Stage Elderly Healthcare data
Nakamoto 2019	[Medical expenses reduction effect by peritoneal dialysis promotion in elderly patients]	MDV		Nationwide		2015		Health economics	Health policy evaluation and utilization	Diseases of the genitourinary system		dialysis		
Yokoyama 2018	[Compliance and persistence of oral calcimimetics in maintenance hemodialysis patients: a pharmacoepidemiological study utilizing electronic health records]	MDV		Nationwide		2009	2015	Patient health service utilization		Diseases of the genitourinary system		maintenance hemodialysis patients		
Saruwatari 2012	[Regional variations in healthcare services for patients treated by hemodialysis in Fukuoka]	NHI/LSEHS	National Health Insurance Association of Fukuoka	Prefecture	Fukuoka	2009	2010	Clinical epidemiology, course of diseases	Socioeconomic comparison	Diseases of the genitourinary system		hemodialysis		
Kinoshita 2018	Hospital-Volume Effects on Perioperative Outcomes in Peritoneal Dialysis Catheter Implantation: Analysis of 2,505 Cases	DPC		Nationwide		2007	2012	Intervention effect	Health policy evaluation and utilization	Diseases of the genitourinary system		peritoneal dialysis catheter implantation		
Mandai 2018	Dialysis Case Volume Associated With In-Hospital Mortality in Maintenance Dialysis Patients	DPC		Nationwide		2012	2014	Clinical epidemiology, course of diseases	Health policy evaluation and utilization	Diseases of the genitourinary system		dialysis	Others	aged ≥20 years
Shinkawa 2019	Mortality and morbidity after pancreatoduodenectomy in patients undergoing hemodialysis: Analysis using a national inpatient database	DPC		Nationwide		2010	2015	Clinical epidemiology, course of diseases	Intervention effect	Diseases of the genitourinary system	Diseases of the digestive system	dialysis, pancreatoduodenectomy	Others	aged ≥20 years
Akiyama 2017	Evaluation of the treatment patterns and economic burden of dysmenorrhea in Japanese women, using a claims database	JMDC		Nationwide		2009	2014	Medical treatment status	Health economics	Diseases of the genitourinary system		primary or secondary dysmenorrhea	Others	aged 18–49 years
Akiyama 2018	Treatment patterns and healthcare resource utilization and costs in heavy menstrual bleeding: a Japanese claims database analysis	JMDC		Nationwide		2009	2014	Medical treatment status	Health economics	Diseases of the genitourinary system		heavy menstrual bleeding	Others	aged 18–49 years
Sugihara 2013	Longer operative time is associated with higher risk of severe complications after percutaneous nephrolithotomy: analysis of 1511 cases from a Japanese nationwide database	DPC		Nationwide		2007	2010	Prediction model	Intervention effect	Diseases of the genitourinary system		ercutaneous nephrolithotomy		
Sugihara 2012	In-hospital outcomes and cost assessment between bipolar versus monopolar transurethral resection of the prostate	DPC		Nationwide		2008	2009	Intervention effect	Health economics	Diseases of the genitourinary system	Others	hyperplasia of the prostate		
Sugihara 2011	Impact of hospital volume and laser use on postoperative complications and in-hospital mortality in cases of benign prostate hyperplasia	DPC		Nationwide		2006	2008	Intervention effect	Health policy evaluation and utilization	Diseases of the genitourinary system		Benign Prostate Hyperplasia		
Takayama 2012	Pharmacoeconomic evaluation of combination therapy with dutasteride and αI blocker for treatment of benign prostatic hyperplasia in Japan	MDV	CombAT Study data	Nationwide	15 hospitals	2008	2009	Health economics		Diseases of the genitourinary system		benign prostatic hyperplasia		
Shinkawa 2020	Risk factors of venous thromboembolism in patients with nephrotic syndrome: a retrospective cohort study	MDV		Nationwide		2008	2017	Clinical epidemiology, course of diseases		Diseases of the genitourinary system		nephrotic syndrome (N04.x)	Others	>18 years of age
Zaitsu 2018	Impact of a direct-to-consumer information campaign on prescription patterns for overactive bladder	JMDC		Nationwide		2010	2014	Medical treatment status	Health policy evaluation and utilization	Diseases of the genitourinary system		overactive bladder	Others	aged 20 years or older
Kato 2017	Persistence and adherence to overactive bladder medications in Japan: A large nationwide real-world analysis	Others	JMDC, Medi-Trend (pharmacy claims database provided by Kyowa Kikaku)	Nationwide		2012	2014	Patient health service utilization		Diseases of the genitourinary system		overactive bladder	Others	aged ≤75 years
Yoshida 2018	Anticholinergic burden in the Japanese elderly population: Use of antimuscarinic medications for overactive bladder patients	Others	Medi-Trend (Kyowa Kikaku)	Nationwide		2016	2016	Medical treatment status		Diseases of the genitourinary system		overactive bladder	Older persons	aged ≥65 years
Shigemi 2019	Laparoscopic Compared With Open Surgery for Severe Pelvic Inflammatory Disease and Tubo-Ovarian Abscess	DPC		Nationwide		2010	2016	Intervention effect		Diseases of the genitourinary system		pelvic inflammatory disease(N739 and N980)with or without tubi-ovarian abscess(N709)	Others	aged 18 years or older
Shigemi 2019	Therapeutic Impact of Initial Treatment for Chlamydia trachomatis Among Patients With Pelvic Inflammatory Disease: A Retrospective Cohort Study Using a National Inpatient Database in Japan	DPC		Nationwide		2010	2016	Intervention effect		Diseases of the genitourinary system		Pelvic inflammatory disease	Others	≥18 year of age
Obinata 2018	Tension-free vaginal mesh surgery versus laparoscopic sacrocolpopexy for pelvic organ prolapse: Analysis of perioperative outcomes using a Japanese national inpatient database	DPC		Nationwide		2014	2015	Intervention effect		Diseases of the genitourinary system		pelvic organ prolapse (N81.x)		
Sugihara 2011	Incidence and clinical features of priapism in Japan: 46 cases from the Japanese diagnosis procedure combination database 2006-2008	DPC		Nationwide		2006	2008	Clinical epidemiology, course of diseases		Diseases of the genitourinary system		priapism (N483)		
Shigemi 2020	Effect of Japanese herbal Kampo medicines on live birth rate in women with recurrent pregnancy loss	JMDC		Nationwide		2005	2018	Intervention effect		Diseases of the genitourinary system	Pregnancy, childbirth and the puerperium	recurrent pregnancy loss	Others	aged ≥18 years
Yamamoto 2015	Relationship between hospital volume and hemorrhagic complication after percutaneous renal biopsy: results from the Japanese diagnosis procedure combination database	DPC		Nationwide		2007	2010	Health policy evaluation and utilization		Diseases of the genitourinary system		percutaneous renal biopsy	Others	aged ≥15 years
Sugihara 2012	Renal haemorrhage risk after extracorporeal shockwave lithotripsy: results from the Japanese Diagnosis Procedure Combination Database	DPC		Nationwide		2006	2008	Clinical epidemiology, course of diseases		Diseases of the genitourinary system		renal haemorrhage	Others	excluded under the age of 15 years
Iwagami 2017	Impact of end-stage renal disease on hospital outcomes among patients admitted to intensive care units: A retrospective matched-pair cohort study	DPC		Nationwide		2011	2011	Clinical epidemiology, course of diseases		Diseases of the genitourinary system		end-stage renal disease	Others	aged ≥20 years
Katsuno 2021	Burden of Renal Events Associated with Nonsteroidal Anti-inflammatory Drugs in Patients with Osteoarthritis and Chronic Low Back Pain: A Retrospective Database Study	JMDC		Nationwide		2009	2018	Intervention effect	Health economics	Diseases of the genitourinary system	Diseases of the musculoskeletal system and connective tissue	renal disease, osteoarthritis and chronic low back pain	Others	≥18 years of age
Doi 2017	Associations of Polyethylenimine-Coated AN69ST Membrane in Continuous Renal Replacement Therapy with the Intensive Care Outcomes: Observations from a Claims Database from Japan	MDV		Nationwide		2014	2015	Intervention effect		Diseases of the genitourinary system		receiving continuous renal replacement therapy	Others	age at baseline ≥18 years
Miyamoto 2019	Temporal change in characteristics and outcomes of acute kidney injury on renal replacement therapy in intensive care units: analysis of a nationwide administrative database in Japan, 2007-2016	DPC		Nationwide		2007	2016	Clinical epidemiology, course of diseases	Intervention effect	Diseases of the genitourinary system		acute kidney injury on renal replacement therapy	Others	aged ≥18 years

Obata 2021	The clinical usage of liposomal amphotericin B in patients receiving renal replacement therapy in Japan: a nationwide observational study	MDV		Nationwide		2008	2018	Medical treatment status	Intervention effect	Diseases of the genitourinary system	Others	broad-spectrum antifungal drug (renal replacement therapy)	Others	≥18 year of age
Sugihara 2013	A nomogram predicting severe adverse events after ureteroscopic lithotripsy: 12 372 patients in a Japanese national series	DPC		Nationwide		2007	2010	Prediction model	Intervention effect	Diseases of the genitourinary system		ureteroscopic lithotripsy		
Adomi 2019	Factors associated with long-term urinary catheterisation and its impact on urinary tract infection among older people in the community: a population-based observational study in a city in Japan	NHI/LSEHS	Medical and long-term care insurance claims data	Municipality	Kashiwa City, Chiba Prefecture	2012	2013	Intervention effect	Medical treatment status	Diseases of the genitourinary system		urinary tract infection	Older persons	≥75 years of age
Imatoh 2018	Association between dipeptidyl peptidase-4 inhibitors and urinary tract infection in elderly patients: A retrospective cohort study	NHI/LSEHS	Wide・Area Association of Latter・Stage Elderly Healthcare	Prefecture	Fukuoka prefecture	2010	2016	Intervention effect		Diseases of the genitourinary system		urinary tract infections (UTI)	Older persons	aged 75 and over
Muramatsu 2018	Efficacy of Antimicrobial Catheters for Prevention of Catheter-Associated Urinary Tract Infection in Acute Cerebral Infarction	DPC		Nationwide		2012	2014	Intervention effect		Diseases of the genitourinary system	Diseases of the circulatory system	Catheter-associated urinary tract infection, cerebral infarction		
Isotani 2019	Endurological treatment trend of upper urinary urolithiasis in Japan from the Japanese Diagnosis Procedure Combination Database	DPC		Nationwide		2008	2012	Medical treatment status		Diseases of the genitourinary system		upper urinary urolithiasis		
15) Pregnancy, childbirth and the puerperium (O00-O99)														
Shigemi 2019	Safety of Laparoscopic Surgery for Benign Diseases during Pregnancy: A Nationwide Retrospective Cohort Study	DPC		Nationwide		2010	2016	Intervention effect		Pregnancy, childbirth and the puerperium		abdominal surgery during pregnancy	Others	aged 13 to 53 years
Maeda 2021	Cesarean delivery rates for overall and multiple pregnancies in Japan: A descriptive study using nationwide health insurance claims data	NDB		Nationwide		2014	2014	Medical treatment status	Socioeconomic comparison	Pregnancy, childbirth and the puerperium		Cesarean delivery		age group
Maeda 2018	Cesarean section rates and local resources for perinatal care in Japan: A nationwide ecological study using the national database of health insurance claims	NDB	Aggregated data	Nationwide		2013	2013	Medical treatment status	Socioeconomic comparison	Pregnancy, childbirth and the puerperium		Cesarean section		age group
Abe 2018	Association between mode of anaesthesia and severe maternal morbidity during admission for scheduled Caesarean delivery: a nationwide population-based study in Japan, 2010-2013	DPC		Nationwide		2010	2013	Intervention effect		Pregnancy, childbirth and the puerperium		severe maternal morbidity, caesarean delivery		
Ishikawa 2018	Antihypertensives prescribed for pregnant women in Japan: Prevalence and timing determined from a database of health insurance claims	JMDC		Nationwide		2005	2016	Medical treatment status		Pregnancy, childbirth and the puerperium	Diseases of the circulatory system	antihypertensives prescribed for pregnant women		
Naruse 2021	Placental abruption in each hypertensive disorders of pregnancy phenotype: a retrospective cohort study using a national inpatient database in Japan	DPC		Nationwide		2010	2018	Clinical epidemiology, course of diseases		Pregnancy, childbirth and the puerperium	Diseases of the circulatory system	Placental abruption, preeclampsia, hypertension	Others	aged 16–49 years
Michihata 2019	Safety and effectiveness of Japanese herbal Kampo medicines for treatment of hyperemesis gravidarum	JMDC		Nationwide		2005	2016	Intervention effect	Health economics	Pregnancy, childbirth and the puerperium	Others	pregnant women	Others	aged ≥19 years
Ueda 2020	[Responses to obstetric bleeding: Nationwide trend survey of obstetric critical bleeding using a database of information such as health insurance claims and specific health checkups]*	NDB		Nationwide		2010	2018	Clinical epidemiology, course of diseases	Medical treatment status	Pregnancy, childbirth and the puerperium		critical obstetrical hemorrhage		
Sato 2019	Nationwide survey of severe postpartum hemorrhage in Japan: an exploratory study using the national database of health insurance claims	NDB	Sampling data	Nationwide		2011	2014	Clinical epidemiology, course of diseases	Health policy evaluation and utilization	Pregnancy, childbirth and the puerperium		postpartum hemorrhage		
Isogai 2017	In-hospital management and outcomes in patients with peripartum cardiomyopathy: a descriptive study using a national inpatient database in Japan	DPC		Nationwide		2007	2014	Clinical epidemiology, course of diseases	Medical treatment status	Pregnancy, childbirth and the puerperium		Peripartum cardiomyopathy	Others	aged 16–55 years
Shigemi 2021	Association between rehabilitation during hospitalization and perinatal outcomes among pregnant women with threatened preterm birth	DPC		Nationwide		2010	2017	Intervention effect		Pregnancy, childbirth and the puerperium	Certain conditions originating in the perinatal period	threatened preterm birth	Others	aged 13-50 years
Hashimoto 2020	Ophthalmic Corticosteroids in Pregnant Women with Allergic Conjunctivitis and Adverse Neonatal Outcomes: Propensity Score Analyses	JMDC		Nationwide		2005	2018	Intervention effect		Pregnancy, childbirth and the puerperium	Certain conditions originating in the perinatal period	topical ophthalmic corticosteroids during pregnancy and adverse neonatal outcomes		
16) Certain conditions originating in the perinatal period (P00-P96)														
Shigeoka 2014	Supplier-induced demand for newborn treatment: evidence from Japan	Others	collecting data from Japanese insurance claims (introduction of the partial prospective payment system)	Nationwide		2004	2008	Health economics	Health policy evaluation and utilization	Certain conditions originating in the perinatal period		newborn treatment	Children	newborn
Maeda 2021	Trends in intensive neonatal care during the COVID-19 outbreak in Japan	MDV		Nationwide		2019	2020	Medical treatment status	COVID-19	Certain conditions originating in the perinatal period		intensive neonatal care	Children	neonate
Ogawa 2018	Maternal exposure to benzodiazepine and risk of preterm birth and low birth weight: A case-control study using a claims database in Japan	JMDC		Nationwide		2005	2014	Intervention effect		Certain conditions originating in the perinatal period	Mental, Behavioral and Neurodevelopmental disorders	benzodiazepine and risk of preterm birth and low birth weight		
17) Congenital malformations, deformations and chromosomal abnormalities (Q00-Q99)														
Ono 2015	Effect of Hospital Volume on Outcomes of Surgery for Cleft Lip and Palate	DPC		Nationwide		2010	2013	Health policy evaluation and utilization	Health economics	Congenital malformations, deformations and chromosomal abnormalities		Cleft lip and cleft palate	Children	children
Lin 2020	Diagnosis and medical care for congenital cytomegalovirus infection: An observational study using claims data in Japan, 2010 to 2017	JMDC		Nationwide		2010	2017	Medical treatment status	Quality of care	Congenital malformations, deformations and chromosomal abnormalities		congenital cytomegalovirus (ICD-10 code P35.1)	Children	newborn
Ishikawa 2021	Validity of congenital malformation diagnoses in healthcare claims from a university hospital in Japan	Others	inpatient and outpatient claims from a university hospital, medical records (as a gold standard)	Others	Tohoku University Hospital	2015	2015	Research methodology		Congenital malformations, deformations and chromosomal abnormalities		Congenital malformations (Q00–Q89)	Children	infants
Murase 2018	Nationwide Increase in Cryptorchidism After the Fukushima Nuclear Accident	DPC		Nationwide		2010	2015	Clinical epidemiology, course of diseases	Others	Congenital malformations, deformations and chromosomal abnormalities		cryptorchidism	Children	congenital diseases
Kimura 2020	Utilization and efficacy of palivizumab for children with Down syndrome	JMDC		Nationwide		2007	2015	Intervention effect	Medical treatment status	Congenital malformations, deformations and chromosomal abnormalities	Certain infectious and parasitic diseases	Down syndrome, respiratory syncytial virus	Children	≤24 months of age
Nomoto 2018	[Epidemiology Study of Risk Factors of Severe Respiratory Syncytial Virus Infectious Diseases in Children with Down Syndrome Using Medical Records]	MDV		Nationwide		2011	2015	Clinical epidemiology, course of diseases		Congenital malformations, deformations and chromosomal abnormalities	Certain infectious and parasitic diseases	Down's syndrome, Respiratory syncrytical virus	Children	aged 0-1 years

Ishimaru 2019	Impact of congenital heart disease on outcomes after primary repair of esophageal atresia: a retrospective observational study using a nationwide database in Japan	DPC		Nationwide		2010	2016	Clinical epidemiology, course of diseases		Congenital malformations, deformations and chromosomal abnormalities		esophageal atresia (ICD code: Q390, 391)	Children	neonates (≤ 2 days old)
Fujiogi 2019	Factors affecting successful atropine therapy for infantile hypertrophic pyloric stenosis: a retrospective analysis using a nationwide database in Japan	DPC		Nationwide		2010	2016	Prediction model	Intervention effect	Congenital malformations, deformations and chromosomal abnormalities		infantile hypertrophic pyloric stenosis (Q400)	Children	0–100 days old
Fujiogi 2020	Clinical features, practice patterns and outcomes of omphalocele: a descriptive study using a nationwide inpatient database in Japan	DPC		Nationwide		2010	2016	Clinical epidemiology, course of diseases	Medical treatment status	Congenital malformations, deformations and chromosomal abnormalities		Omphalocele (ICD-10 code: Q792)	Children	babies (≤2 days old)
Michihata 2016	Hospital volume and mortality due to preterm patent ductus arteriosus	DPC		Nationwide		2010	2013	Health policy evaluation and utilization		Congenital malformations, deformations and chromosomal abnormalities		Preterm patent ductus arteriosus (ICD-10 code: Q250)	Children	excluded: age >1 year at admission
Nagano 2017	Surgery versus percutaneous transcatheter embolization for pulmonary arteriovenous malformation: Analysis of a national inpatient database in Japan	DPC		Nationwide		2010	2015	Intervention effect		Congenital malformations, deformations and chromosomal abnormalities	Diseases of the circulatory system	pulmonary arteriovenous malformation (ICD-10 code: I28.0)		
Fujiogi 2019	Early Outcomes of Laparoscopic Versus Open Surgery for Urachal Remnant Resection in Children: A Retrospective Analysis Using a Nationwide Inpatient Database in Japan	DPC		Nationwide		2015	2017	Intervention effect		Congenital malformations, deformations and chromosomal abnormalities	Injury, poisoning and certain other consequences of external causes	urachal remnant, postoperative complications	Children	age: 0-18 years
18) Injury, poisoning and certain other consequences of external causes (S00-T98)														
Kondo 2019	Characteristics, treatments, and outcomes among patients with abdominal aortic injury in Japan: a nationwide cohort study	DPC		Nationwide		2010	2017	Clinical epidemiology, course of diseases	Medical treatment status	Injury, poisoning and certain other consequences of external causes		Abdominal aortic injury (ICD-10 code: S350)	Others	age ≥18 years
Otaka 2021	Association between parenteral nutrition in the early phase and outcomes in patients with abdominal trauma undergoing emergency laparotomy: A retrospective nationwide study	DPC		Nationwide		2010	2018	Intervention effect		Injury, poisoning and certain other consequences of external causes		abdominal trauma undergoing emergency laparotomy	Others	age of >18 years
Ohbe 2019	Extracorporeal membrane oxygenation improves outcomes of accidental hypothermia without vital signs: A nationwide observational study	DPC		Nationwide		2010	2017	Intervention effect		Injury, poisoning and certain other consequences of external causes		accidental hypothermia	Others	aged ≥19 years
Muramatsu 2020	Epidemiological Study of Hospital Admissions for Food-induced Anaphylaxis using the Japanese Diagnosis Procedure Combination Database	DPC		Nationwide		2014	2017	Clinical epidemiology, course of diseases		Injury, poisoning and certain other consequences of external causes		anaphylactic shock due to an adverse food reaction (ICD-10 code T78.0)		age group
Nakajima 2020	Epinephrine autoinjector prescription patterns for severe anaphylactic patients in Japan: A retrospective analysis of health insurance claims data	JMDC		Nationwide		2011	2016	Medical treatment status	Quality of care	Injury, poisoning and certain other consequences of external causes		anaphylaxis		
Okubo 2021	Effects of Glucocorticoids on Hospitalized Children With Anaphylaxis	DPC		Nationwide		2010	2015	Intervention effect	Medical treatment status	Injury, poisoning and certain other consequences of external causes	Diseases of the skin and subcutaneous tissue	anaphylaxis	Children	younger than 18 years
Yasunaga 2010	Short report: Clinical features of bowel anisakiasis in Japan	DPC		Nationwide		2007	2008	Clinical epidemiology, course of diseases		Injury, poisoning and certain other consequences of external causes		anisakiasis (ICD code, T810)		
Tsuchiya 2018	Tracheostomy and mortality in patients with severe burns: A nationwide observational study	DPC		Nationwide		2010	2014	Intervention effect		Injury, poisoning and certain other consequences of external causes		severe burns	Others	aged ≥18 years
Tagami 2017	Antithrombin use and 28-day in-hospital mortality among severe-burn patients: an observational nationwide study	DPC		Nationwide		2010	2013	Intervention effect		Injury, poisoning and certain other consequences of external causes		severe burns		
Tagami 2015	Validation of the prognostic burn index: a nationwide retrospective study	DPC	(relationships between PBI and mortality)	Nationwide		2010	2013	Research methodology		Injury, poisoning and certain other consequences of external causes		burn patients		
Endo 2019	Volume-outcome relationship on survival and cost benefits in severe burn injury: a retrospective analysis of a Japanese nationwide administrative database	DPC		Nationwide		2010	2015	Health economics	Health policy evaluation and utilization	Injury, poisoning and certain other consequences of external causes		burn injury		
Matsuo 2020	Age-dependent influence of premorbid underweight status on mortality in severe burn patients: An administrative database study	DPC		Nationwide		2014	2018	Clinical epidemiology, course of diseases		Injury, poisoning and certain other consequences of external causes		burn injuries	Others	18-84 years
Tagami 2016	Prophylactic Antibiotics May Improve Outcome in Patients With Severe Burns Requiring Mechanical Ventilation: Propensity Score Analysis of a Japanese Nationwide Database	DPC		Nationwide		2010	2013	Intervention effect		Injury, poisoning and certain other consequences of external causes		severe burn		
Nakajima 2019	Effect of high-dose vitamin C therapy on severe burn patients: a nationwide cohort study	DPC		Nationwide		2010	2016	Intervention effect		Injury, poisoning and certain other consequences of external causes		burn (ICD-10 codes: T20–T32)	Others	aged ≥15 years
Tagami 2019	Haptoglobin use and acute kidney injury requiring renal replacement therapy among patients with severe burn injury: a nationwide database study	DPC		Nationwide		2010	2013	Intervention effect		Injury, poisoning and certain other consequences of external causes		burn		
Nakajima 2020	Hyperbaric oxygen therapy and mortality from carbon monoxide poisoning: A nationwide observational study	DPC		Nationwide		2010	2017	Intervention effect		Injury, poisoning and certain other consequences of external causes		carbon monoxide poisoning (ICD-10 code: T58)	Others	adult patients
Nakajima 2019	Disaster-related carbon monoxide poisoning after the Great East Japan Earthquake, 2011: a nationwide observational study	DPC		Nationwide		2010	2017	Clinical epidemiology, course of diseases	Others	Injury, poisoning and certain other consequences of external causes		CO poisoning		
Chikuda 2014	Mortality and morbidity after high-dose methylprednisolone treatment in patients with acute cervical spinal cord injury: a propensity-matched analysis using a nationwide administrative database	DPC		Nationwide		2007	2009	Intervention effect		Injury, poisoning and certain other consequences of external causes		cervical spinal cord injury (SCI)(ICD-10 code: S141)		
Ohbe 2018	Cholinergic Crisis Caused by Cholinesterase Inhibitors: a Retrospective Nationwide Database Study	DPC		Nationwide		2010	2016	Intervention effect		Injury, poisoning and certain other consequences of external causes		cholinergic crisis (T44.0)		age categories
Toi 2018	Present epidemiology of chronic subdural hematoma in Japan: analysis of 63,358 cases recorded in a national administrative database	DPC		Nationwide		2010	2013	Clinical epidemiology, course of diseases		Injury, poisoning and certain other consequences of external causes		chronic subdural hematoma (S0650)		no restriction on patient age
Okumura 2017	Epidemiology of overdose episodes from the period prior to hospitalization for drug poisoning until discharge in Japan: An exploratory descriptive study using a nationwide claims database	NDB		Nationwide		2012	2013	Clinical epidemiology, course of diseases	Medical treatment status	Injury, poisoning and certain other consequences of external causes		overdose episodes (defined as an initial definitive diagnosis of drug poisoning (ICD10: T360-T509))		
Kanehara 2016	[Psychiatric intervention and repeated admission to emergency centers due to drug overdose]	DPC	DPC Study Group	Nationwide		2010	2013	Intervention effect		Injury, poisoning and certain other consequences of external causes	Mental, Behavioral and Neurodevelopmental disorders	psychiatric intervention, drug overdose	Others	aged ≥12 years
Okumura 2012	Characteristics, procedural differences, and costs of inpatients with drug poisoning in acute care hospitals in Japan	DPC		Nationwide		2008	2008	Medical treatment status	Health economics	Injury, poisoning and certain other consequences of external causes		drug poisoning	Others	aged 12 or above
Matsuda 2016	[An analysis of the utilization of medical care services for femoral neck fracture patients using the comprehensive medical care database]*	NHI/LSEHS	medical insurance claims and LTC insurance claims data	Municipality	A municipality in Fukuoka	2010	2013	Health economics	Health policy evaluation and utilization	Injury, poisoning and certain other consequences of external causes		femoral neck fracture		
Tsuda 2014	Effects of fondaparinux on pulmonary embolism following hemiarthroplasty for femoral neck fracture: a retrospective observational study using the Japanese Diagnosis Procedure Combination database	DPC		Nationwide		2007	2010	Intervention effect		Injury, poisoning and certain other consequences of external causes		femoral neck fractrue (S72.0)		
Hara 2020	[Assessment of early surgery for hip fracture from the diagnosis procedure combination database]	MDV		Nationwide		2017	2017	Intervention effect	Health economics	Injury, poisoning and certain other consequences of external causes		fracture of the femur	Older persons	aged ≥65 years

Nishioka 2020	Body mass index and recovery of activities of daily living in older patients with femoral fracture: An analysis of a national inpatient database in Japan	JMDC	Japan Medical Data Center (JMDC) Diagnosis Procedure Combination (DPC) database	Nationwide		2014	2017	Clinical epidemiology, course of diseases		Injury, poisoning and certain other consequences of external causes		acute femoral fracture	Older persons	aged ≥65 years
Tanaka 2016	Validation Study of Claims-based Definitions of Suspected Atypical Femoral Fractures Using Clinical Information	Others	claims databases, electronic medical record databases	Others	three hospital s	2010	2012	Research methodology		Injury, poisoning and certain other consequences of external causes		atypical femoral fractures		
Tanaka 2012	[An investigation of factors affecting in-hospital mortality of proximal femoral fracture using the DPC database]*	DPC	DPC Study Group	Nationwide		2007	2009	Clinical epidemiology, course of diseases		Injury, poisoning and certain other consequences of external causes		fracture of the neck of the femur, pertrochanteric fractur (S72.0 S72.1)	Older persons	excluded <60 years old
Hayashi 2019	Data regarding fracture incidence according to fracture site, month, and age group obtained from the large public health insurance claim database in Japan	NDB		Others	Kanto area	2013	2016	Clinical epidemiology, course of diseases	Research methodology	Injury, poisoning and certain other consequences of external causes		fracture		
Hayashi 2019	Variation in fracture risk by season and weather: A comprehensive analysis across age and fracture site using a National Database of Health Insurance Claims in Japan	NDB		Others	Kanto area	2013	2016	Clinical epidemiology, course of diseases		Injury, poisoning and certain other consequences of external causes		fracture		
Iihara 2016	Polypharmacy of medications and fall-related fractures in older people in Japan: a comparison between driving-prohibited and driving-cautioned medications	NDB	Sampling data	Nationwide		2011	2012	Clinical epidemiology, course of diseases	Medical treatment status	Injury, poisoning and certain other consequences of external causes		fall-related fractures	Older persons	aged ≥65 years
Miyake 2015	Characteristics of heat-illness patients in Japan : Analysis from receipt data for the past	NDB		Nationwide		2010	2014	Clinical epidemiology, course of		Injury, poisoning and certain other		heatstroke		
Jamal 2020	Multilevel analysis of hemodialysis-associated infection among end-stage renal disease patients: results of a retrospective cohort study utilizing the insurance claim data of Fukuoka Prefecture, Japan	NHI/LSEHS	Fukuoka Prefecture Association of Latter Stage Elderly Healthcare	Prefecture	Fukuoka	2015	2017	Clinical epidemiology, course of diseases	Socioeconomic comparison	Injury, poisoning and certain other consequences of external causes		hemodialysis-associated infection	Older persons	≥65 years of age
Yamazaki 2020	[The effect of rehabilitation to the patient with osteosynthesis of the proximal femoral fractures]	DPC		Nationwide		2013	2014	Intervention effect		Injury, poisoning and certain other consequences of external causes		hip fracture	Others	aged ≥15 years
Maki 2019	Impact of Number of Drug Types on Clinical Outcome in Patients with Acute Hip Fracture	JMDC		Nationwide		2014	2017	Intervention effect		Injury, poisoning and certain other consequences of external causes		acute hip fracture (ICD-10 code: S70)	Older persons	aged ≥65 years
Fukuda 2019	[Surgery for hip fracture within 36 hours of injury requires additional emergency care management Analysis of 2017 DPC data]*	MDV		Nationwide		2017	2017	Health economics		Injury, poisoning and certain other consequences of external causes		Hip fracture	Others	aged <65 years
Mori 2018	Estimated expenditures for hip fractures using merged healthcare insurance data for individuals aged >= 75 years and long-term care insurance claims data in Japan	NHI/LSEHS	healthcare insurance and long-term care insurance claims data	Municipality	City of Kashiwa	2012	2013	Health economics		Injury, poisoning and certain other consequences of external causes		hip fracture	Older persons	aged ≥75 years
Tomioka 2018	Equality of treatment for hip fracture irrespective of regional differences in socioeconomic status: Analysis of nationwide hospital claims data in Japan	DPC		Nationwide		2011	2014	Medical treatment status	Socioeconomic comparison	Injury, poisoning and certain other consequences of external causes		hip fracture	Others	aged ≥40 years
Matsuda 2020	[Big data analysis of medical and nursing care: An analysis of the usage of medical and nursing care services of elderly hip fracture patients using consolidated data on health insurance claims in medical and nursing care]*	NHI/LSEHS	medical insurance claims and LTC insurance claims data	Others	A municip ality in Western Japan	2012	2016	Clinical epidemiology, course of diseases	Health policy evaluation and utilization	Injury, poisoning and certain other consequences of external causes		hip fracture	Older persons	older patients
Nishi 2019	Comparison of regional with general anesthesia on mortality and perioperative length of stay in older patients after hip fracture surgery	NHI/LSEHS	Fukuoka Prefecture Regional Association for Late-Stage Healthcare for Older People	Prefecture	Fukuoka Prefectu re	2012	2016	Intervention effect		Injury, poisoning and certain other consequences of external causes		hip fracture surgery (S72.0-2)	Older persons	Older People
Nishi 2018	Association Between Financial Incentives for Regional Care Coordination and Health Care Resource Utilization Among Older Patients after Femoral Neck Fracture Surgery: A Retrospective Cohort Study Using a Claims Database	NHI/LSEHS	Fukuoka Prefecture Regional Association for Late-Stage Healthcare for Older People	Prefecture	Fukuoka	2010	2016	Health economics	Health policy evaluation and utilization	Injury, poisoning and certain other consequences of external causes		hip fracture	Older persons	75 years or older
Shoda 2012	Risk factors affecting inhospital mortality after hip fracture: retrospective analysis using the Japanese Diagnosis Procedure Combination Database	DPC		Nationwide		2007	2009	Clinical epidemiology, course of diseases	Intervention effect	Injury, poisoning and certain other consequences of external causes		single hip fracture	Others	aged ≥60 years
Ogawa 2021	Seasonal impact on surgical site infections in hip fracture surgery: Analysis of 330,803 cases using a nationwide inpatient database	DPC		Nationwide		2011	2016	Clinical epidemiology, course of diseases		Injury, poisoning and certain other consequences of external causes		hip fracture	Older persons	≥65 years of age
Mine 2020	Effectiveness of regional clinical pathways on postoperative length of stay for hip fracture patients: A retrospective observational study using the Japanese Diagnosis Procedure Combination database	DPC		Nationwide		2011	2013	Intervention effect	Health policy evaluation and utilization	Injury, poisoning and certain other consequences of external causes		hip fracture		
Tamaki 2019	Estimates of hip fracture incidence in Japan using the National Health Insurance Claim Database in 2012–2015	NDB	Aggregated data	Nationwide		2012	2015	Clinical epidemiology, course of diseases		Injury, poisoning and certain other consequences of external causes		hip fracture	Others	aged ≥40 years
Matsuda 2016	Relationship Between Depressive State and Treatment Characteristics of Acute Cervical Spinal Cord Injury in Japan	DPC		Nationwide		2010	2010	Clinical epidemiology, course of diseases		Injury, poisoning and certain other consequences of external causes	Mental, Behavioral and Neurodevelopmental disorders	unspecified injuries of cervical spinal cord (ICD-10 code; S14.1), depressive state		age group
Wada 2015	Effectiveness of surgical rib fixation on prolonged mechanical ventilation in patients with traumatic rib fractures: A propensity score-matched analysis	DPC		Nationwide		2010	2013	Intervention effect		Injury, poisoning and certain other consequences of external causes		injuries including rib fractures (S223, S224, or S225)	Others	excluded younger than 20 years
Aso 2020	Influence of the Fukushima Daiichi Nuclear Power Plant Accident on the Use of Computed Tomography in Children With Mild Head Injuries	JMDC		Nationwide		2008	2013	Medical treatment status	Others	Injury, poisoning and certain other consequences of external causes		mild head injuries, use of CT	Children	patients aged ≤15 years
Yamaoka 2020	Incidence and Age Distribution of Hospitalized Presumptive and Possible Abusive Head Trauma of Children Under 12 Months Old in Japan	DPC		Nationwide		2010	2013	Clinical epidemiology, course of diseases	Socioeconomic comparison	Injury, poisoning and certain other consequences of external causes	Others	Abusive head trauma	Children	under 36 months old
Maeda 2018	Safety of Tranexamic Acid During Pediatric Trauma: A Nationwide Database Study	DPC		Nationwide		2010	2014	Intervention effect		Injury, poisoning and certain other consequences of external causes		pediatric trauma	Children	less than or equal to 12 years old
Wada 2018	Development and validation of an ICD-10-based disability predictive index for patients admitted to hospitals with trauma	DPC		Nationwide		2010	2015	Prediction model	Research methodology	Injury, poisoning and certain other consequences of external causes		trauma (S00 to T14)	Others	aged ≥15 years
Wada 2017	Development and validation of a new ICD-10-based trauma mortality prediction scoring system using a Japanese national inpatient database	DPC		Nationwide		2007	2014	Prediction model	Clinical epidemiology, course of diseases	Injury, poisoning and certain other consequences of external causes		injuries		age group
Endo 2018	[ASSOCIATION BETWEEN SEVERE TRAUMA PATIENT VOLUME, SURVIVAL BENEFIT, AND TOTAL HEALTHCARE COSTS ; ANALYSIS OF JAPANESE NATIONAL ADMINISTRATIVE DATABASE]	DPC		Nationwide		2010	2015	Health policy evaluation and utilization	Health economics	Injury, poisoning and certain other consequences of external causes		Severe Trauma Patient	Others	aged ≥16 years
Aso 2017	Resuscitative endovascular balloon occlusion of the aorta or resuscitative thoracotomy with aortic clamping for noncompressible torso hemorrhage: A retrospective nationwide study	DPC		Nationwide		2010	2014	Intervention effect		Injury, poisoning and certain other consequences of external causes		trauma patients with uncontrolled hemorrhagic shock	Others	aged 15 years or older
Miyamoto 2020	Pregabalin and injury: A nested case-control and case-crossover study	JMDC		Nationwide		2014	2016	Intervention effect		Injury, poisoning and certain other consequences of external causes	Diseases of the nervous system	use of pregabalin and injury	Others	patients aged ≥20 years

Sumitani 2011	Prevalence of malignant hyperthermia and relationship with anesthetics in Japan: data from the diagnosis procedure combination database	DPC		Nationwide		2006	2008	Intervention effect		Injury, poisoning and certain other consequences of external causes		Malignant Hyperthermia (T883)		
Ono 2016	Impact of Body Mass Index on the Outcomes of Open Reduction for Mandibular Fractures	DPC		Nationwide		2010	2013	Clinical epidemiology, course of diseases	Intervention effect	Injury, poisoning and certain other consequences of external causes		mandibular fracture	Others	aged ≥18 years
Kishimoto 2018	Suspected periprosthetic joint infection after total knee arthroplasty under propofol versus sevoflurane anesthesia: a retrospective cohort study	DPC		Nationwide		2012	2015	Intervention effect		Injury, poisoning and certain other consequences of external causes		Periprosthetic joint infection	Others	exclude age <40
Suzuki 2014	Impact of systemic steroids on posttonsillectomy bleeding: analysis of 61 430 patients using a national inpatient database in Japan	DPC		Nationwide		2007	2013	Intervention effect		Injury, poisoning and certain other consequences of external causes	Others	postoperative bleeding after tonsillectomy	Others	50 years or older were excluded
Ohya 2017	Seasonal Variations in the Risk of Reoperation for Surgical Site Infection Following Elective Spinal Fusion Surgery	DPC		Nationwide		2010	2013	Clinical epidemiology, course of diseases		Injury, poisoning and certain other consequences of external causes		postoperative surgical site infection	Others	aged 20 years or older
Fukuda 2016	Patient-related risk factors for surgical site infection following eight types of gastrointestinal surgery	DPC	DPC, JANIS	Nationwide		2007	2011	Clinical epidemiology, course of diseases		Injury, poisoning and certain other consequences of external causes		Surgical site infections		
Kitazawa 2014	Perioperative patient safety indicators and hospital surgical volumes	DPC		Nationwide		2008	2010	Quality of care	Health policy evaluation and utilization	Injury, poisoning and certain other consequences of external causes		perioperative patient		
Sugihara 2014	Does mechanical bowel preparation ameliorate damage from rectal injury in radical prostatectomy? Analysis of 151 rectal injury cases	DPC		Nationwide		2007	2012	Intervention effect		Injury, poisoning and certain other consequences of external causes		rectal injury		
Sugihara 2012	Management trends, angioembolization performance and multiorgan injury indicators of renal trauma from Japanese administrative claims database	DPC		Nationwide		2006	2008	Clinical epidemiology, course of diseases	Medical treatment status	Injury, poisoning and certain other consequences of external causes		renal trauma (ICD-10 code: S37.0)		
Otaka 2020	Effectiveness of surgical fixation for rib fractures in relation to its timing: a retrospective Japanese nationwide study	DPC		Nationwide		2010	2018	Intervention effect		Injury, poisoning and certain other consequences of external causes		rib fractures (ICD-10 codes; S22.3–S22.5)		
Otaka 2020	Early Versus Late Rib Fixation in Patients With Traumatic Rib Fractures: A Nationwide Study	DPC		Nationwide		2010	2018	Intervention effect		Injury, poisoning and certain other consequences of external causes		rib fractures (ICD-10 codes; S22.3–S22.5)		
Ohya 2015	Risks of in-hospital death and complications after fusion surgery in patients with atlantoaxial subluxation: analysis of 1090 patients using the Japanese Diagnosis Procedure Combination database	DPC		Nationwide		2007	2012	Clinical epidemiology, course of diseases	Intervention effect	Injury, poisoning and certain other consequences of external causes	Diseases of the musculoskeletal system and connective tissue	Atlantoaxial Subluxation, rheumatoid arthritis	Others	aged 20 years or older
Wada 2018	Impact of hospital volume on mortality in patients with severe torso injury	DPC		Nationwide		2010	2014	Health policy evaluation and utilization		Injury, poisoning and certain other consequences of external causes		severe torso injury	Others	aged ≥15 years
Obikane 2020	Cumulative visits for care of minor injuries are associated with traumatic brain injury in young children	JMDC		Nationwide		2009	2012	Clinical epidemiology, course of diseases	Others	Injury, poisoning and certain other consequences of external causes		minor injuries, traumatic brain injuries	Children	aged ≤36 months
Yamaoka 2020	Impact of Number of Drugs on Rehabilitation Outcomes in Patients after Traumatic Brain Injury: A Retrospective Cohort Study	JMDC		Nationwide		2014	2017	Intervention effect		Injury, poisoning and certain other consequences of external causes		acute traumatic brain injury	Others	not included aged >90 years
Nishina 2020	[Investigation of Characteristics and Risk Factors for Death in Elderly People Who Suffered from Traumatic Brain Injury Occurred in Hospital]	DPC	DPC Study Group	Nationwide		2010	2017	Clinical epidemiology, course of diseases		Injury, poisoning and certain other consequences of external causes		traumatic brain injury	Older persons	65 years old and older
Ohbe 2020	Early enteral nutrition in patients with severe traumatic brain injury: a propensity score-matched analysis using a nationwide inpatient database in Japan	DPC		Nationwide		2014	2017	Intervention effect		Injury, poisoning and certain other consequences of external causes		traumatic brain injury	Others	aged ≥16 years
Tagami 2014	Thoracic aortic injury in Japan: Nationwide retrospective cohort study	DPC		Nationwide		2007	2013	Clinical epidemiology, course of diseases	Medical treatment status	Injury, poisoning and certain other consequences of external causes		traumatic thoracic aortic injury	Others	aged ≥18 years
Hamasaki 2020	Incidence of clinical vertebral fractures and hip fractures of the elderly (65 years or over) population-large-scale data analysis using claim database in Kure City, Hiroshima, Japan	NHI/LSEHS	National Health Insurance (NHI) or Senior Elderly Care System (SECS)	Municipality	Kure City	2015	2015	Clinical epidemiology, course of diseases		Injury, poisoning and certain other consequences of external causes		vertebral fractures	Older persons	over 65 years of age
Kobata 2020	Effectiveness of Early Rehabilitation for Vertebral Compression Fractures: A Retrospective Cohort Study	JMDC		Nationwide		2014	2018	Intervention effect		Injury, poisoning and certain other consequences of external causes		vertebral compression fracture	Older persons	65 years or older
Hamasaki 2020	[Incidence of clinical vertebral fractures and the rate for operation of the elderly in Kure City; large-scale claim database analysis]	NHI/LSEHS	The National Health Insurance system and Late Elders' Health Insurance	Municipality	Kure City	2015	2015	Clinical epidemiology, course of diseases	Medical treatment status	Injury, poisoning and certain other consequences of external causes		clinical vertebral fractures	Older persons	aged 65 and over
Toba 2017	[Develop a monitoring procedure for cases of bone fractures and intracranial bleeding due to falls based on integrating adverse event data extracted from medical fee information and other data]	DPC	medical records	Others	Medical Hospital, Tokyo Medical and Dental University	2012	2015	Research methodology		Injury, poisoning and certain other consequences of external causes		Fractures, Intracranial Hemorrhages	Others	
19) Others														
Murata 2014	Age-related differences in outcomes and etiologies of acute abdominal pain based on a national administrative database	DPC		Nationwide		2009	2011	Clinical epidemiology, course of diseases		Others		acute abdominal pain (R100)		age groups
Kobayashi 2012	[INVESTIGATION ON EVALUATION OF THE QUALITY OF MEDICAL CARE BASED ON CLINICAL INDICATORS BY THE UTILIZATION OF DIAGNOSIS PROCEDURE COMBINATION DATA]	DPC		Nationwide		2010	2010	Quality of care		Others		medical care at acute care hospitals		
Ueyama 2014	Application of a self-controlled case series study to a database study in children	MDV		Nationwide		2003	2011	Research methodology	Health policy evaluation and utilization	Others		diarrhea, bronchitis, and eczema related to palivizumab treatment	Children	aged between 0 and 5 years
Li 2021	Characterizing the incidence of adverse events of special interest for COVID-19 vaccines across eight countries: a multinational network cohort study	JMDC	13 databases from eight countries	Nationwide		2017	2019	Intervention effect	COVID-19	Others		adverse events of special interest for COVID-19 vaccines		age groups
Koizumi 2021	Effect of population inflow and outflow between rural and urban areas on regional antimicrobial use surveillance	NDB	Aggregated data	Nationwide	natinal, prefectural, second medical area	2015	2015	Medical treatment status	Socioeconomic comparison	Others		antimicrobial consumption		age category
Mita 2021	An alternative index for evaluating AMU and anti-methicillin-resistant Staphylococcus aureus agent use: A study based on the National Database of Health Insurance Claims and Specific Health Checkups data of Japan	NDB	Aggregated data	Nationwide		2016	2016	Medical treatment status	Research methodology	Others		antimicrobial consumption		
Hashimoto 2020	Indications and classes of outpatient antibiotic prescriptions in Japan: A descriptive study using the national database of electronic health insurance claims, 2012-2015	NDB		Nationwide		2012	2015	Medical treatment status		Others		outpatient antibiotic prescriptions		age group
Maeda 2020	Development of the predicted and standardized carbapenem usage metric: Analysis of the Japanese Diagnosis Procedure Combination payment system data	DPC		Nationwide		2017	2017	Prediction model	Research methodology	Others		antimicrobial consumption		

Ono 2020	The first national survey of antimicrobial use among dentists in Japan from 2015 to 2017 based on the national database of health insurance claims and specific health checkups of Japan	NDB	Aggregated data	Nationwide		2015	2017	Medical treatment status		Others	antimicrobial consumption		
Kinoshita 2019	Nationwide study of outpatient oral antimicrobial utilization patterns for children in Japan (2013-2016)	NDB		Nationwide		2013	2016	Medical treatment status	Health policy evaluation and utilization	Others	antimicrobial consumption	Children	<15 year of age
Tanaka 2019	Evaluation of the usefulness of antimicrobial use survey using claims data	DPC	claims data and electronic medical records	Others	National Centre for Global Health and Medicine	2016	2017	Research methodology		Others	antimicrobial use		
Yamasaki 2018	The first report of Japanese antimicrobial use measured by national database based on health insurance claims data (2011-2013): comparison with sales data, and trend analysis stratified by antimicrobial category and age group	NDB	Aggregated data: sales data previously reported	Nationwide		2011	2013	Research methodology	Medical treatment status	Others	antimicrobial use		age group
Gu 2011	[Outpatient antimicrobial drug use and antimicrobial resistance frequency in Suwa area, Nagano, Japan]	NHI/LSEHS	NHI	Prefecture	Suwa, Nagano prefecture	2009	2010	Medical treatment status		Others	Antimicrobial Drug Use		
Matsushita2011	[Study on Prescription Status and Clinical Utility of Injectable Antimicrobial Agents by Retrospective Analysis of Multicenter Medical Cost Accounting Data]	MDV		Nationwide		2006	2010	Medical treatment status	Intervention effect	Others	Antimicrobial Drug Use		
Goto 2019	[Investigation on the current status surrounding biologics and the impact of biosimilars on drug cost using real world data]	MDV		Nationwide		2012	2018	Medical treatment status	Health economics	Others	biosimilar		
Sekimoto 2010	[LINEAR REGRESSION MODELS PREDICT HOSPITAL-WIDE BLOOD PRODUCT USE]	DPC	QIP	Nationwide		2006	2008	Prediction model	Medical treatment status	Others	blood product use		
Yonekura 2019	Use of preoperative haemostasis and ABO blood typing tests in children: a retrospective observational study using a nationwide claims database in Japan	JMDC		Nationwide		2012	2018	Medical treatment status		Others	preoperative haemostasis and ABO blood typing tests	Children	aged 1–17 years
Yonekura 2018	Preoperative Blood Tests Conducted Before Low-Risk Surgery in Japan: A Retrospective Observational Study Using a Nationwide Insurance Claims Database	JMDC		Nationwide		2012	2016	Medical treatment status	Quality of care	Others	preoperative blood tests before low-risk surgery	Others	20–64 years of age
Tarasawa 2019	[A Study on factors related to blood culture performing rate preceding broad-spectrum antimicrobial agent therapy : Analyses of clinical indicators using the DPC data]	DPC	DPCRI	Nationwide		2016	2017	Medical treatment status		Others	Detection of bloodstream infections		
Matsuda 2018	[Big data analysis of medical and nursing care: An analysis of outpatient functions of chronic hospitals]*	NHI/LSEHS	medical insurance claims and LTC insurance claims data	Others	municipalities in Western Japan	2015	2015	Health policy evaluation and utilization		Others	Chronic disease	Older persons	Older People
Matsuda 2018	[Big data analysis of medical and nursing care: An analysis of inpatient functions of chronic hospitals]*	NHI/LSEHS	medical insurance claims (The National Health Insurance system and Late Elders' Health Insurance)	Others	municipalities in Western Japan	2015	2016	Health policy evaluation and utilization		Others	Chronic disease		
Matsuda 2018	[Big data analysis of medical and nursing care: An analysis of the structure of sickness of inpatients by classification of long-term care beds]*	NHI/LSEHS	medical insurance claims (The National Health Insurance system and Late Elders' Health Insurance)	Others	municipalities in Western Japan	2015	2015	Medical treatment status	Health policy evaluation and utilization	Others	chronic stage bed	Older persons	Older People
Imai 2020	Prescription of Colchicine with Other Dangerous Concomitant Medications: A Nation-Wide Survey Using the Japanese Claims Database	JMDC		Nationwide		2014	2017	Medical treatment status		Others	inappropriate colchicine prescriptions		
Lai 2018	Applying a common data model to Asian databases for multinational pharmacoepidemiologic studies: opportunities and challenges	JMDC		Nationwide	Japan, Taiwan, Hong Kong, South Korea, and US			Research methodology	Socioeconomic comparison	Others	multinational pharmacoepidemiologic studies		
Amano 2020	The Prevalence and Characteristics of Older Japanese Adults with Polypharmacy, Based on Regionally Representative Health Insurance Claims Data	NHI/LSEHS	National Health Insurance and Late-stage Elderly Health Insurance	Prefecture	Nagasaki	2016	2016	Medical treatment status	Patient health service utilization	Others	polypharmacy among elderly		
Ishizaki 2020	Drug prescription patterns and factors associated with polypharmacy in >1 million older adults in Tokyo	NHI/LSEHS	Tokyo Metropolitan Association of Medical Care for Senior Citizens	Prefecture	Tokyo	2014	2014	Medical treatment status	Patient health service utilization	Others	polypharmacy in older outpatients	Older persons	aged ≥75 years
Sato 2018	Potentially Inappropriate Medication Prescribing and Risk of Unplanned Hospitalization among the Elderly: A Self-Matched, Case-Crossover Study	MDV		Nationwide		2009	2015	Quality of care		Others	potentially inappropriate medication	Older persons	patients aged 65 years and above
Iihara 2014	[Survey of Usage of Medication with Driving with Prohibition or Caution by the National Health Insurance Claims Database in Japan]	NDB	Sampling data	Nationwide		2011	2011	Medical treatment status		Others	prohibitions or cautions on driving	Others	25 years and older
Akazawa 2010	Potentially inappropriate medication use in elderly Japanese patients	JMDC		Nationwide	(5 corporate health insurance societies)	2006	2007	Quality of care		Others	potentially inappropriate medications	Older persons	aged ≥65 years
Ogata 2019	Lower-dose prescriptions in the post-marketing situation and the influencing factors thereon	MDV		Nationwide		2015	2015	Medical treatment status	Health policy evaluation and utilization	Others	lower-dose prescription drugs		
Takahashi 2016	Social network analysis of duplicative prescriptions: One-month analysis of medical facilities in Japan	JMDC		Nationwide		2012	2012	Medical treatment status		Others	duplicative prescriptions		age group

Brauer 2020	Application of a Common Data Model (CDM) to rank the paediatric user and prescription prevalence of 15 different drug classes in South Korea, Hong Kong, Taiwan, Japan and Australia: an observational, descriptive study	JMDC		Nationwide	South Korea, Hong Kong, Taiwan, Japan and Australia	2009	2013	Medical treatment status	Socioeconomic comparison	Others		prescription prevalence of 15 different drug classes	Children	18 years or younger
Seki 2015	[Drug Utilization Study of Potential Drug-drug Interactions using the Sampling Dataset of the National Database of Health Insurance Claim Information]	NDB	Sampling data	Nationwide		2011	2012	Medical treatment status		Others		potential drug-drug interactions		
Maeda 2016	Investigation of the Existence of Supplier-Induced Demand in use of Gastrostomy Among Older Adults: A Retrospective Cohort Study	NHI/LSEHS	Late Elders' Health Insurance	Prefecture	Fukuoka	2010	2013	Health policy evaluation and utilization		Others		eating difficulties (F50: Eating disorders; J69: Pneumonitis due to solids and liquids; R13: Dysphagia; R630: Anorexia; and R633: Feeding difficulties)	Older persons	aged ≥75 years, or those aged 65 to 74 years with a specific disability
Kondo 2021	Efficacy of Prophylactic Antibiotics during Extracorporeal Membrane Oxygenation: A Nationwide Cohort Study	DPC		Nationwide		2010	2017	Intervention effect		Others		patients receiving ECMO treatment	Others	≥18 year of age
Aso 2016	In-hospital mortality and successful weaning from venoarterial extracorporeal membrane oxygenation: analysis of 5,263 patients using a national inpatient database in Japan	DPC		Nationwide		2010	2013	Clinical epidemiology, course of diseases		Others		receiving VA-ECMO (cardiogenic shoc, pulmonary embolism, hypothermia, poisoning, or trauma)	Others	aged ≥19 years
Muguruma 2020	Epidemiology and volume-outcome relationship of extracorporeal membrane oxygenation for respiratory failure in Japan: A retrospective observational study using a national administrative database	DPC		Nationwide		2010	2018	Intervention effect	Health policy evaluation and utilization	Others		underwent ECMO	Others	aged ≥18 years
Kondo 2020	Proton pump inhibitors versus histamine-2 receptor antagonists for stress ulcer prophylaxis during extracorporeal membrane oxygenation: a propensity score-matched analysis	DPC		Nationwide		2010	2017	Intervention effect		Others	Diseases of the digestive system	gastrointestinal bleeding (receiving ECMO)	Others	≥18 year of age
Matsuda 2013	Analysis of Transfer Distance of Emergency Stroke Cases in Kumamoto, Japan	DPC		Prefecture	Kumamoto	2010	2010	Health policy evaluation and utilization		Others		emergency cases by pathology base (neurological, cardiologic, pediatric and injury cases)		
Kuwahara 2010	[[Outline and problems of DPC system reform] Evaluation of emergency medical care using DPC data]*	DPC	hospitals participated in the study group	Nationwide		2009	2009	Medical treatment status	Health economics	Others		emergency medicine		
Sugitani 2016	Postmarketing Benefit-Risk Assessment for Erythropoiesis-Stimulating Agents Using a Health Care Database	MDV		Nationwide		2011	2014	Health policy evaluation and utilization		Others		erythropoietin agents	Others	≥15 years
Sekimoto 2010	[RISK-ADJUSTED ASSESSMENT OF FRESH FROZEN PLASMA AND ALBUMIN PRODUCT USE IN ACUTE-CARE HOSPITALS IN JAPAN: AN ANALYSIS USING ADMINISTRATIVE DATA]	DPC	QIP	Nationwide		2006	2008	Prediction model	Medical treatment status	Others		fresh frozen plasma (FFP) and albumin products	Others	adult
Uchida 2011	Impact of remifentanil introduction on practice patterns in general anesthesia	DPC		Nationwide		2006	2007	Medical treatment status	Health policy evaluation and utilization	Others		general anesthesia		
Takeda 2019	Utilization of arterial pulse waveform analysis during non-cardiac surgery in Japan: a retrospective observational study using a nationwide claims database	MDV		Nationwide		2014	2016	Medical treatment status		Others		non-cardiac surgery under general anesthesia with an arterial catheter	Others	aged 18 years or older
Takeda 2020	The association between arterial pulse waveform analysis device and in-hospital mortality in high-risk non-cardiac surgeries	MDV		Nationwide		2014	2016	Intervention effect		Others		patients undergoing high-risk surgery under general anaesthesia		
Ito 2019	Can income-based co-payment rates improve disparity? The case of the choice between brand-name and generic drugs	NHI/LSEHS	Medical Care System of Latter-stage Elderly	Prefecture	a large prefecture	2013	2014	Patient health service utilization	Health policy evaluation and utilization	Others		brand-name drug, generic drug	Older persons	≥75 years of age
Kinoshita 2019	Effect of tranexamic acid on mortality in patients with haemoptysis: a nationwide study	DPC		Nationwide		2010	2017	Intervention effect		Others		haemoptysis (ICD-10 code: R042)	Others	age ≥18 years
Miyawaki 2019	Effect of a medical subsidy on health service utilization among schoolchildren: A community-based natural experiment in Japan	NHI/LSEHS	NHI	Prefecture	Prefecture N's NHI organization	2012	2017	Health policy evaluation and utilization	Health economics	Others		health service expenditure	Children	aged 6–11 at baseline
Osawa 2020	Machine-learning-based prediction models for high-need high-cost patients using nationwide clinical and claims data	Others	MinaCare	Nationwide		2013	2016	Prediction model	Health economics	Others		High-need, high-cost patients	Others	aged ≥18 years
Tanke 2019	A challenge to all. A primer on inter-country differences of high-need, high-cost patients	Others	Japanese data came from two prefectures of insurance claims of those not currently employed	Prefecture	two prefectures (Canada, England, Germany, Japan, the Netherlands, Spain and the United States)	2014	2014	Health economics	Socioeconomic comparison	Others		high-need high-cost patients		
Imahori 2019	[Does income affect medical care and long-term care expenditures for the elderly? : Evidence from claims data of a municipality in Japan]	NHI/LSEHS	National Health Insurance and Later-Stage Elderly Healthcare System, long-term care data	Municipality	X city	2014	2015	Health economics	Socioeconomic comparison	Others		medical care expenditures	Others	aged ≥66 years
Akiyama 2018	Healthcare costs for the elderly in Japan: Analysis of medical care and long-term care claim records	NHI/LSEHS	MC (NHI) and LTC insurance data	Municipality	city A, Hokkaido	2007	2009	Health economics	Clinical epidemiology, course of diseases	Others		healthcare costs	Older persons	aged 65 years or older

Akiyama 2017	[Seasonal effects on healthcare costs: Using panel data from medical and long-term care claim records of the elderly in Hokkaido]	NHI/LSEHS	NHI and/or MC insurance systems for the elderly aged over 65 years old, LTC insurance data	Municipality	city A, Hokkaido	2007	2009	Health economics	Clinical epidemiology, course of diseases	Others	healthcare costs	Older persons	over 65 years old
Hiratsuka 2017	[Relationships between medical expenditures and the Specific Health Checkups scheme in Japan: A Study of outpatient medical expenditures and questionnaire responses concerning lifestyle that form part of the Specific Health Checkups scheme in Japan]	NHI/LSEHS	NHI	Municipality	Mishima City	2008	2012	Health economics		Others	the annual outpatients medical expenditures		
Fukushima 2016	Patient cost sharing and medical expenditures for the Elderly	JMDC		Nationwide		2005	2013	Health policy evaluation and utilization	Health economics	Others	medical spending	Older persons	elderly between 67 and 72 years old
Tamaki 2014	[Investigation of risk factors for increased medical expenses by matching data on health insurance claims: An analysis of questionnaires and each test item in specific health checkups]*	NHI/LSEHS	NHI	Municipality	Mishima city	2008	2013	Health economics		Others	medical expenditure		
Otsubo 2011	Evaluation of resource allocation and supply-demand balance in clinical practice with high-cost technologies	Others	claims data from 16 sampled hospitals (QIP), National Survey of Medical Care Institutions and National Patient Survey (MHLW)	Nationwide	16 sampled hospitals (QIP)	2001	2005	Health policy evaluation and utilization		Others	high-cost technologies (MRI, ESWL)		
Tomio 2010	[Association between the past health checkup results and health expenditures using the National Health Insurance claims data]	NHI/LSEHS	NHI	Municipality	a municipality in Kumamoto	2006	2007	Health economics		Others	medical expenditure	Others	aged 45–84 years
Mitsutake 2010	[Examination of specific health guidance recipients based on specific health checkup data and medical expense data]*	Others	a health insurance society and NHI (5 Municipalities)	Others	a health insurance society and NHI (5 Municip	2008	2008	Health economics	Others	Others	special health check-up		
Mori 2020	Association of long-term care needs, approaching death and age with medical and long-term care expenditures in the last year of life: An analysis of insurance claims data	NHI/LSEHS	insurance claims (medical and LTC insurance systems) and death certificates	Municipality	Soma City	2006	2009	Health economics		Others	medical and long-term care expenditures in the last year of life	Older persons	aged ≥65 years
Hamada 2019	Household Income Relationship With Health Services Utilization and Healthcare Expenditures in People Aged 75 Years or Older in Japan: A Population-Based Study Using Medical and Long-term Care Insurance Claims Data	NHI/LSEHS	medical and LTC claims	Municipality	suburban city in the Tokyo	2012	2013	Socioeconomic comparison	Patient health service utilization	Others	healthcare services utilization	Older persons	aged 75 years or older
Yasui 2016	[Analysis of the inpatient health care costs for the late elderly]	NHI/LSEHS	Fukuoka Prefecture WideArea Association of LatterStage Elderly Healthcare	Prefecture	Fukuoka	2010	2014	Health economics	Socioeconomic comparison	Others	inpatient health care costs	Older persons	aged 75 years and older
Anzai 2011	[A comparative analysis of medical expenses for elderly people with or without certification of long-term care need]*	NHI/LSEHS	National Health Insurance, Health Care System for the Late Elderly	Prefecture	Siga	2008	2008	Health economics		Others	medical expenses for the elderly	Older persons	aged 65 years and above
Hashimoto 2010	Micro data analysis of medical and long-term care utilization among the elderly in Japan	NHI/LSEHS	medical care and LTC by National Health Insurance	Prefecture	in a prefecture in the Kyushu district	2000	2004	Health economics		Others	cost of end-of-life care for the elderly	Older persons	aged 65 and over
Noguchi 2010	[[Empirical verification of medical and long-term care policies] Empirical verification based on National Health Insurance claims data of the impact of uneven distribution of medical resources on the range of medical consultations, the number of treatment days, and medical costs]*	NHI/LSEHS	WAM NET	Municipality	Nakatonnbetsu-chou	2003	2007	Health economics	Health policy evaluation and utilization	Others	health service utilization		
Tanihara 2012	A comparison of disease-specific medical expenditures in Japan using the principal diagnosis method and the proportional distribution method	JMDC		Nationwide		2006	2006	Health economics	Research methodology	Others	disease-specific medical expenditures		
Shin 2020	Economic impact of the first wave of the COVID-19 pandemic on acute care hospitals in Japan	DPC	QIP	Nationwide		2018	2020	COVID-19	Health economics	Others	hospital charges		
Mimura 2019	The Association Between Internet Searches and Moisturizer Prescription in Japan: Retrospective Observational Study	JMDC		Nationwide		2007	2017	Medical treatment status		Others	identified heparinoid prescription		
Koizumi 2018	In-Hospital Mortality for Hepatic Portal Venous Gas: Analysis of 1590 Patients Using a Japanese National Inpatient Database	DPC		Nationwide		2010	2015	Clinical epidemiology, course of diseases		Others	hepatic portal venous gas (ICD-10 code R93.2)	Others	less than 18 years of age were excluded
Teramoto 2019	Impact of a national medical fee schedule revision on the cessation of physician home visits among older patients in Tokyo: A retrospective study	NHI/LSEHS	Tokyo Metropolitan Association of Medical Care Services for Older Senior Citizens	Prefecture	Tokyo	2014	2014	Health policy evaluation and utilization		Others	physician home visits	Older persons	aged 75 years or older
Matsuda 2018	[Big data analysis of medical and nursing care: An analysis of user characteristics by type of home medical treatment]*	NHI/LSEHS	medical insurance claims and LTC insurance claims data	Others	A municipality in Western Japan	2015	2015	Medical treatment status		Others	in-home medical care		

Matsuda 2018	[Big data analysis of medical and nursing care: An analysis of the current status of home care management guidance by pharmacists]*	NHI/LSEHS	medical insurance claims and LTC insurance claims data	Others	A municipality in Western Japan	2016	2016	Medical treatment status		Others	home medication management and guidance by pharmacists	Older persons	Older People
Matsuda 2018	[Big data analysis of medical and nursing care: An analysis of characteristics of elderly people requiring home nursing care by frequency of home nursing care]*	NHI/LSEHS	medical insurance claims and LTC insurance claims data	Others	A municipality in Western Japan	2015	2015	Health policy evaluation and utilization		Others	home care service	Older persons	Older People
Matsuda 2018	[Big data analysis of medical and nursing care: An analysis of user characteristics by type of home nursing care]*	NHI/LSEHS	medical insurance claims and LTC insurance claims data	Others	A municipality in Western Japan	2015	2015	Health policy evaluation and utilization		Others	home nursing care		
Ishizaki 2018	[Home medical treatment by a medical institution outside Tokyo for home care patients who have an insurance card address in Tokyo]*	NHI/LSEHS		Prefecture	Tokyo	2014	2014	Medical treatment status		Others	in-home medical care	Older persons	aged ≥75 years
Naruse 2017	Measurement of special access to home visit nursing services among Japanese disabled elderly people: using GIS and claim data	NHI/LSEHS	medical and long-term care insurance claims data	Prefecture	A Prefecture	2010	2010	Health policy evaluation and utilization		Others	access to home visit nursing	Older persons	aged ≥75 years
Kato 2017	Effect of reducing cost sharing for outpatient care on children's inpatient services in Japan	DPC		Nationwide		2012	2013	Health policy evaluation and utilization	Medical treatment status	Others	overall hospital admissions	Children	children
Tarasawa 2020	[The characteristics of hospitalized patients and the proportion of hospitalization by ambulance from the viewpoint of presence or absence of home medical care before hospitalization : Cross-sectional analysis using large-scale DPC data for elderly people]	DPC		Nationwide		2016	2016	Clinical epidemiology, course of diseases	Medical treatment status	Others	hospitalized patients	Older persons	65 years old and older
Matsuda 2019	[An analysis of the current status of hospitalization from nursing homes and welfare facilities based on DPC data]*	DPC	DPC Study Group	Nationwide		2016	2016	Socioeconomic comparison		Others	Hospitalizations from nursing home	Older persons	Older People
Jeon 2018	Potentially avoidable hospitalizations, non-potentially avoidable hospitalizations and in-hospital deaths among residents of long-term care facilities	NHI/LSEHS	Long-term care and national health insurance claims data	Municipality	a suburban city of Chiba prefecture	2012	2013	Clinical epidemiology, course of diseases	Socioeconomic comparison	Others	potentially avoidable hospitalizations	Older persons	aged ≥75 years
Mitsutake 2018	[The associations between readmission within 30 days and the medical institute factors among older patients receiving home medical care]	NHI/LSEHS		Prefecture	Tokyo	2013	2014	Clinical epidemiology, course of diseases	Health policy evaluation and utilization	Others	readmission	Older persons	aged 75 years or older
Sasabuchi 2018	Effect of the 2016 Kumamoto earthquakes on preventable hospital admissions: a retrospective cohort study in Japan	NHI/LSEHS	National Health Insurance, Late Elders' Health Insurance	Prefecture	Kumamoto	2013	2016	Medical treatment status	Others	Others	preventable hospital admissions		
Yuda 2018	The medical assistance system and inpatient health care provision: Empirical evidence from short-term hospitalizations in Japan	Others	Survey of Medical Care Activities in Public Health Insurance, Fact-finding Survey on Medical Assistance	Nationwide		2000	2010	Medical treatment status	Socioeconomic comparison	Others	short-term inpatient health care provision		
Yano 2019	Establishing a hospital transfusion management system promotes appropriate clinical use of human albumin in Japan: a nationwide retrospective study	DPC		Nationwide		2012	2016	Medical treatment status	Health policy evaluation and utilization	Others	use of human albumin	Others	aged 15 years or older
Iwagami 2015	Choice of renal replacement therapy modality in intensive care units: data from a Japanese Nationwide Administrative Claim Database	DPC		Nationwide		2011	2011	Medical treatment status	Intervention effect	Others	adult patients admitted to ICUs	Others	aged 20 years or older
Ohnuma 2018	Predictors associated with unplanned hospital readmission of medical and surgical intensive care unit survivors within 30 days of discharge	DPC		Nationwide		2012	2014	Clinical epidemiology, course of diseases	Medical treatment status	Others	ICU survivors	Others	aged ≥18 years
Hayashida 2013	[Revenue and cost in Japanese ICU focused on the use of life-support system and length of stay in the diagnosis procedure combination/per-diem payment system]	DPC	hospitals participated in the research group	Nationwide		2010	2010	Health economics		Others	ICU		
Tarasawa 2021	[Evaluation of Unknown Registration and Utilization of SOFA Score : Analyses of ICU Emergency Inpatients Using Large-scale DPC Data]	DPC	DPC Research Institute	Nationwide		2018	2019	Research methodology		Others	SOFA Score	Others	aged ≥15 years
Sakai 2021	Decreased Administration of Life-Sustaining Treatment just before Death among Older Inpatients in Japan: A Time-Trend Analysis from 2012 through 2014 Based on a Nationally Representative Sample	NDB	Sampling data	Nationwide		2012	2014	Medical treatment status		Others	administration of life-sustaining treatments (LSTs) and intensive care unit (ICU) admissions just before death in older inpatients	Older persons	aged ≥65 years
Ohbe 2021	Intensive care unit occupancy in Japan, 2015-2018: a nationwide inpatient database study	DPC		Nationwide		2015	2018	Health economics	Health policy evaluation and utilization	Others	ICU bed occupancy		
Sasabuchi 2015	The Volume-Outcome Relationship in Critically Ill Patients in Relation to the ICU-to-Hospital Bed Ratio	DPC		Nationwide		2007	2012	Health policy evaluation and utilization		Others	ICU admitted patients	Others	aged ≥18 years
Hayashida 2013	[Revenue and cost in Japanese ICU focused on the use of life-support system and length of stay in the diagnosis procedure combination/per-diem payment system]	DPC		Nationwide		2010	2010	Health economics		Others	ICU		
Tomio 2017	Tuberculosis screening prior to anti-tumor necrosis factor therapy among patients with immune-mediated inflammatory diseases in Japan: a longitudinal study using a large-scale health insurance claims database	JMDC		Nationwide		2013	2014	Medical treatment status	Quality of care	Others	immune-mediated inflammatory diseases (M05–06 for rheumatoid arthritis, K50 for Crohn's disease, K51 for ulcerative colitis and L40 for psoriasis)	Others	aged 15–69 years
Yamana 2020	Outpatient Prescriptions of Kampo Formulations in Japan	JMDC		Nationwide		2017	2018	Medical treatment status		Others	patients with outpatient prescriptions of Kampo extract formulations		
Michihata 2018	[Japanese Herbal Kampo Medicine for Pediatric Inpatients in Japan : A Retrospective Nationwide Study]	DPC	DPCRI	Nationwide		2014	2015	Medical treatment status		Others	Herbal Kampo medicine	Children	aged <20 years
Katayama 2013	Prescription of kampo drugs in the Japanese health care insurance program	Others	health care claim records, which had been collected by MHLW	Nationwide		2009	2009	Medical treatment status		Others	Prescription of Kampo Drugs		

Hayashida 2016	The relationship between sedative drug utilization and outcomes in critically ill patients undergoing mechanical ventilation	DPC	QIP	Nationwide		2008	2010	Intervention effect	Medical treatment status	Others	undergoing mechanical ventilation	Others	aged ≥20 years
Sasaki 2016	Hospital Volume and Mortality in Mechanically Ventilated Children: Analysis of a National Inpatient Database in Japan	DPC		Nationwide		2010	2013	Health policy evaluation and utilization		Others	mechanical ventilation	Children	15 years old or younger
Yagi 2021	Outcomes After Intensive Rehabilitation for Mechanically Ventilated Patients: A Nationwide Retrospective Cohort Study	DPC		Nationwide		2010	2016	Intervention effect		Others	patients with mechanical ventilation in intensive care units	Others	20 years and older
Ibayashi 2021	Estimation of the number of patients with mitochondrial diseases: A descriptive study using a nationwide database in Japan	NDB		Nationwide		2018	2019	Clinical epidemiology, course of diseases		Others	mitochondrial disease (ICD10: D640, E744,E888, G318, G713, H472, H494, H498)		
Matsumoto 2021	Changes in the Penetration Rate of Biosimilar Infliximab Within Japan Using a Japanese Claims Database	MDV		Nationwide		2013	2019	Medical treatment status		Others	rheumatoid arthritis (M05, M06), Crohn's disease (K50), ulcerative colitis (K51), and psoriasis (L40) (ICD10)		
Yamana 2017	Validity of diagnoses, procedures, and laboratory data in Japanese administrative data	DPC	NHO, chart review results as reference standards	Others	four acute-care HMO hosapitals (Shizuoka, Kochi, Fukuoka, and Saga Prefectures)	2014	2015	Research methodology		Others	16 diseases	Others	aged ≥18 years
Yamana 2015	Categorized diagnoses and procedure records in an administrative database improved mortality prediction	DPC		Nationwide		2012	2013	Prediction model	Clinical epidemiology, course of diseases	Others	acute myocardial infarction, congestive heart failure, acute cerebrovascular disease, gastrointestinal hemorrhage, pneumonia, or septicemia	Others	adult patients (≥18 years)
Sano 2021	Large decrease in paediatric hospitalisations during the COVID-19 outbreak in Japan	MDV		Nationwide		2017	2020	COVID-19	Medical treatment status	Others	paediatric hospitalisations (food allergy, acute lower respiratory infections, Kawasaki disease, intestinal infectious diseases, febrile convulsions, asthma, appendicitis, inguinal hernia and trauma)	Children	aged 1–17 years
Mitsutake 2019	Patterns of Co-Occurrence of Chronic Disease Among Older Adults in Tokyo, Japan	NHI/LSEHS	Tokyo Extended Association of Medical Care System for the Latter-Stage Elderly People	Prefecture	Tokyo	2013	2014	Clinical epidemiology, course of diseases		Others	22 chronic diseases	Older persons	aged 75 or older
Naruse 2015	Diseases that precede disability among latter-stage elderly individuals in Japan	NHI/LSEHS	long-term care insurance system	Prefecture	Fukui	2011	2012	Clinical epidemiology, course of diseases	Health policy evaluation and utilization	Others	nine types of diseases as predisposing factors for disability (cancer, cerebrovascular disorder, arthropathy, fracture, pneumonia, dementia, psychiatric disorder, neurological disorder)	Older persons	aged 75 years or older
Moriwaki 2018	[Evaluation of healthcare safety indicators by the Diagnosis Procedure Combination data]	DPC		Nationwide		2014	2015	Quality of care		Others	6 healthcare safety indicators		
Yamana 2015	Procedure-based severity index for inpatients: development and validation using administrative database	DPC		Nationwide		2012	2013	Prediction model	Research methodology	Others	acute myocardial infarction, congestive heart failure, acute cerebrovascular disease, gastrointestinal hemorrhage, pneumonia, or septicemia	Others	≥18 years
Fujiu 2018	Evaluation of Regional Vulnerability to Disasters by People of Ishikawa, Japan: A Cross Sectional Study Using National Health Insurance Data	NHI/LSEHS	KDB (national health insurance data)	Municipality	Hakui City in Ishikawa Prefecture	2015	2015	Health policy evaluation and utilization		Others	disaster-vulnerable patients (specifically, hart disease, CVD, dialysis)	Others	at least 40 years old
Murata 2013	Association between ambulance distance to hospitals and mortality from acute diseases in Japan: national database analysis	DPC		Nationwide		2008	2008	Socioeconomic comparison	Clinical epidemiology, course of diseases	Others	acute diseases (acute myocardial infarction, brain infarction, subarachnoid hemorrhage, and pneumonia)		
Takeda 2016	Estimating the Ratio of Patients with a Certain Disease Between Hospitals for the Allocation of Patients to Clinical Trials Using Health Insurance Claims Data in Japan	Others	health insurance claims data, chart review	Others	Osaka University Hospital	2015	2015	Research methodology		Others	329 diseases	Others	<20 years of age (F90-F98)
Shimizu 2014	Assessment of Medical Information Databases to Estimate Patient Numbers	Others	JMDC, MDV, JMIRI (IMS-JPM)	Nationwide		2009	2012	Research methodology		Others	Seven diseases: benign prostatic hyperplasia, Parkinson's disease, herpes zoster, gout, asthma, epilepsy, and atopic dermatitis		
Kasamo 2021	Changes in prescribing trends and initial pharmacotherapy of children with nocturnal enuresis in Japan: a large-scale medical claims database analysis	JMDC		Nationwide		2005	2019	Medical treatment status		Others	Nocturnal enuresis(ICD-10:R32)	Children	children < 16 years
Nakatani 2021	Data resource profile of Shizuoka Kokuho Database (SKDB) using integrated health- and care-insurance claims and health checkups: the Shizuoka Study	NHI/LSEHS	Shizuoka Kokuho Database	Prefecture	Shizuoka Prefecture	2012	2018	Research methodology		Others	not specified		
Shin 2020	New outcome-specific comorbidity scores excelled in predicting in-hospital mortality and healthcare charges in administrative databases	DPC	QIP database	Nationwide		2016	2018	Prediction model	Clinical epidemiology, course of diseases	Others	comorbidities composing Charlson comorbidity index (CCI), Elixhauser comorbidity (EC), Gagne's combined comorbidity scores	Others	aged ≥18 years
Shinjo 2017	The degree of severity and trends in hospital standardized mortality ratios in Japan between 2008 and 2012: a retrospective observational study	DPC		Nationwide		2008	2012	Prediction model	Health policy evaluation and utilization	Others	Hospital standardized mortality ratio	Others	did not include patients less than 15 years old

Tanihara 2015	Assessment of text documentation accompanying uncoded diagnoses in computerized health insurance claims in Japan	NHI/LSEHS	National Health Insurance Organization of Kumamoto Prefecture	Prefecture	Kumamoto	2010	2010	Research methodology		Others	all disease categories		
Ooba 2013	Claims-based definition of death in Japanese claims database: validity and implications	JMDC		Nationwide		2005	2009	Research methodology		Others	Not specified	Others	aged 20 to 74
Sato 2012	Impact of teaching intensity and academic status on medical resource utilization by teaching hospitals in Japan	DPC	study group	Nationwide	40 national university academic teaching hospitals	2004	2004	Health economics		Others	Not specified		
Ohbe 2019	Physician-manned prehospital emergency care in tertiary emergency centers in Japan	DPC		Nationwide		2014	2015	Medical treatment status	Clinical epidemiology, course of diseases	Others	physician-manned prehospital emergency medical service		
Sato 2019	Enabling Patient Traceability Using Anonymized Personal Identifiers in Japanese Universal Health Insurance Claims Database	Others	national-level insurance claims data (NDB) and prefectural-level insurance claims data	Nationwide	municipality-administered health insurance programs in Mie Prefecture	2009	2014	Research methodology		Others	Not specified		
Kubo 2017	[The need and key points for patient matching in clinical studies using the National Database of Health Insurance Claims and Specific Health Checkups of Japan (NDB)]	NDB		Nationwide		2013	2014	Research methodology		Others	patient matching		
Noda 2017	[Improvements and verification of the patient matching (name matching) method in health insurance claims information and National DataBase (NDB) on specific medical checkups]*	NDB		Nationwide		2013	2014	Research methodology		Others	patient matching		
Nagamine 2016	[Consultation trends before and after the introduction of the subsidy system for child medical expenses in municipalities: From an analysis of health insurance claims from City A before and after the introduction of the system]*	NHI/LSEHS	NHI	Municipality	A city	2009	2013	Health policy evaluation and utilization		Others	use of medications and health care services	Children	school-age children
Matsuda 2015	A Pilot Study of Developing the Health Data Analysis System in the Japanese Occupational Setting	Others	claim data of one enterprise managed health insurance	Others	one enterprise managed health insurance	2011	2012	Health policy evaluation and utilization		Others	natural history of various diseases		
Tanihara 2011	A statistical analysis of 'rule-out' diagnoses in outpatient health insurance claims in Japan	Others	corporate health insurance societies	Others	corporate health insurance societies	2006	2006	Medical treatment status		Others	'rule-out' diagnoses		
Kawaguchi 2010	[Study about Evaluation of Hospital Efficiency and Function Used by DPC Data Set]	DPC		Nationwide		2005	2007	Research methodology	Health policy evaluation and utilization	Others	Not specified		
Fukuda 2019	[The Development of Dataset Tables for NDB Analyses]	NDB		Nationwide		2009	2016	Research methodology		Others	Not specified		
Fukuda 2019	[A comparison of correction methods for medical fee revisions in health expenditure analyses using claims data]	NDB		Nationwide		2009	2016	Research methodology		Others	Not specified		
Imai 2017	[Development and Validation of a Medicationbased Comorbidity Index for Use with an Administrative Database in Japan]	Others	NHO: National Hospital Organization	Nationwide		2011	2013	Prediction model	Clinical epidemiology, course of diseases	Others	comorbidity index	Others	aged ≥15 years
Bono 2017	[Approach Aiming at the Increase with the Discharge Drug Information Management Instruction Charges Calculation Method Review]	DPC	Federation of National Public Services Personnel Mutual Aid Associations	Nationwide		2015	2016	Medical treatment status		Others	Discharge drug information management instruction charges		
Ohtsu 2014	[The Impact of Short-term Health Insurance Card and Eligibility Certification Card on Health Care Access in Japan : Findings from National Health Insurance Receipt Data]	NHI/LSEHS	short-term health insurance card and eligibility certification card data	Municipality	X city	2008	2011	Health policy evaluation and utilization	Patient health service utilization	Others	Not specified	Others	Excludes children under high school age and seniors over 75
Tanihara 2012	Estimating medical expenditures spent on rule-out diagnoses in Japan	Others	corporate health insurance societies	Others	corporate health insurance societies	2006	2006	Health economics	Medical treatment status	Others	Not specified		
Nishi 2012	[Study of the indicators of affordable healthcare planning using electronic medical claim data]	NHI/LSEHS	Fukuoka National Health Insurance Organization	Prefecture	Fukuoka	2009	2010	Research methodology	Health policy evaluation and utilization	Others	affordable healthcare planning		

Itoh 2021	Comparison of progression of care-need levels among long-term care recipients with and without advanced care management in a rural municipality of Japan: A population-based observational study	NHI/LSEHS	medical care claim records from a rural municipal government and the Survey of Long-Term Care Benefit Expenditures	Others	A rural municipality in Japan	2012	2017	Intervention effect		Others	progression of careneed levels among long-term care recipients	Older persons	aged ≥65 years
Kim 2021	Comparison of care utilisation and medical institutional death among older adults by home care facility type: a retrospective cohort study in Fukuoka, Japan	NHI/LSEHS	medical claims database and an LTC insurance claims database (provided by the Fukuoka Prefecture Association of Latterstage Elderly Healthcare)	Prefecture	Fukuoka	2014	2017	Patient health service utilization		Others	care utilisation and the incidence of medical institutional death	Older persons	aged ≥75 years
Kono 2021	Five-year disease-related risk of mortality in ambulatory frail older Japanese	NHI/LSEHS	long-term care and health insurance claims data (accumulated electronically by local governments; SOHA study)	Municipality	Izumi, Misaki, and Izumiotsu (Osaka)	2012	2017	Clinical epidemiology, course of diseases		Others	Five-year disease-related risk of mortality in ambulatory frail older	Older persons	aged ≥65 years
Iwagami 2019	Association between recorded medical diagnoses and incidence of long-term care needs certification: a case control study using linked medical and long-term care data in two Japanese cities	NHI/LSEHS	medical and long-term care insurance claims	Municipality	two cities	2012	2016	Clinical epidemiology, course of diseases	Health policy evaluation and utilization	Others	all disease categories	Older persons	people aged ≥75 years
Mori 2019	The associations of multimorbidity with the sum of annual medical and long-term care expenditures in Japan	NHI/LSEHS	medical insurance claims data (of the Late-Stage Medical Care System for the Elderly) and LTC insurance claims data	Municipality	Kashiwa city	2012	2013	Health economics		Others	multimorbidity (Charlson Comorbidity Index scores)	Older persons	adults ≥75 years
Sakai 2019	Validation of claims data to identify death among aged persons utilizing enrollment data from health insurance unions	JMDC		Nationwide		2012	2015	Research methodology		Others	Inpatient and outpatient deaths	Older persons	aged 65–74 years
Matsuda 2019	[Big data analysis of medical and nursing care: A study on evaluating the quality of collaboration between home medical care and long-term care]*	NHI/LSEHS	medical insurance claims and LTC insurance claims data	Others	municipality in Western Japan	2012	2017	Quality of care	Health policy evaluation and utilization	Others	Not specified	Older persons	aged 75 years or older
Masuzawa 2019	[Analysis of a long-term health care organaization from structural analysis of medical expenditures of the elderly]	NHI/LSEHS	National Health Insurance and the late-stage medical care system	Municipality	Yokohama, Kawasaki, and Odawar	2010	2015	Health economics		Others	Not specified	Older persons	Older People
Takada 2018	Development and validation of a prediction model for functional decline in older medical inpatients	MDV		Nationwide		2014	2015	Prediction model	Clinical epidemiology, course of diseases	Others	risk of functional decline	Older persons	65 years old or older
Matsuda 2018	[Big data analysis of medical and nursing care: An analysis of the structure of injury and disease according to the level of nursing care required]*	NHI/LSEHS	medical insurance claims and LTC insurance claims data	Others	A municipality in Western Japan	2015	2015	Health policy evaluation and utilization		Others	common diseases	Older persons	Older People
Matsuda 2018	[Big data analysis of medical and nursing care: A trial study on the economic effects of long-term care prevention and comprehensive lifestyle projects]*	NHI/LSEHS	medical insurance claims and LTC insurance claims data	Others	A municipality in Western Japan	2008	2014	Health policy evaluation and utilization		Others	Not specified	Older persons	Older People
Matsuda 2018	[Big data analysis of medical and nursing care: Examination of the ideal "family pharmacy" from the perspective of the calculation of pharmaceutical management fees]*	NHI/LSEHS	Dispensing claims (The National Health Insurance system and Late Elders' Health Insurance)	Others	A municipality in Western Japan	2015	2015	Health policy evaluation and utilization		Others	Not specified		
Ishizaki 2017	Cumulative number of hospital bed days among older adults in the last year of life: A retrospective cohort study	NHI/LSEHS	medical insurance claims and LTC insurance claims data	Municipality	Soma City	2006	2009	Medical treatment status		Others	main cause of death	Older persons	aged ≥65years
Akiyama 2015	[About factors relating to the admission of the elderly with nursing care needs certification]	NHI/LSEHS	National Health Insurance and the late-stage medical care system (+LTC insurance)	Municipality	A city in Hokkaido prefecture	2007	2009	Health policy evaluation and utilization		Others	nursing care needs	Older persons	aged 65 years and above
Kimura 2010	Development of a database of health insurance claims: standardization of disease classifications and anonymous record linkage	JMDC		Nationwide		2003	2003	Research methodology		Others	Development of a database		
Matsuda 2010	[Development of a consolidated analysis system for health insurance/long-term care insurance claims and specific health checkup data]*	NHI/LSEHS	medical insurance claims and LTC insurance claims data	Municipality	A municipality in Fukuoka	2009	2009	Research methodology		Others	Not specified		
Hirota 2020	Association between clinic physician workforce and avoidable readmission: a retrospective database research	DPC		Nationwide		2014	2014	Health policy evaluation and utilization		Others	ambulatory care sensitive conditions	Older persons	≥65 years of age

Fujita 2016	Income Related Inequality of Health Care Access in Japan: A Retrospective Cohort Study	NHI/LSEHS	NHI	Municipality	Chiba City	2012	2014	Socioeconomic comparison	Patient health service utilization	Others		health care access	Others	0-74 years old
Suzuki 2014	[Regional diagnosis of municipalities within the prefecture using public data: Regional disparities in medical expenses: Regional diagnosis based on data on health insurance claims]*	NHI/LSEHS	NHI	Prefecture	Miyagi	2012	2012	Health economics	Socioeconomic comparison	Others		Not specified		
Kisa 2012	Medical Reimbursement Receipt Analysis to Determine the Relationship between Disease Type and Patients' Healthcare-seeking Behavior	NHI/LSEHS	NHI	Municipality	four towns in Hokkaido	2006	2010	Patient health service utilization		Others		disease type and healthcare-seeking behavior		
Miyata 2010	Assessment of hospital performance with a case-mix standardized mortality model using an existing administrative database in Japan	DPC		Nationwide		2006	2006	Prediction model	Health policy evaluation and utilization	Others		hospital performance		
Nojiri 2019	Comorbidity status in hospitalized elderly in Japan: Analysis from National Database of Health Insurance Claims and Specific Health Checkups	NDB		Nationwide		2015	2016	Clinical epidemiology, course of diseases		Others		comorbidity status of hospitalized elderly patients	Older persons	aged ≥60 years
Miyazaki 2012	A study on medical cost analysis and construction of an evaluation system for optimization of medical costs in Shimane Prefecture (2010)	NHI/LSEHS	NHI	Prefecture	Shimane	2001	2010	Health economics	Health policy evaluation and utilization	Others		Not specified	Others	under 75 years
Iwabuchi 2014	[Disease estimation and medical practice estimation using DPC data and regional population estimates: Reflection of a decline in biological functions and development of lifestyle-related diseases]*	DPC	Estimate of population	Prefecture	Yamagata	2012	2013	Health policy evaluation and utilization		Others		not specified		
Kuwabara 2011	Contribution of case-mix classification to profiling hospital characteristics and productivity	DPC		Nationwide		2006	2006	Health policy evaluation and utilization		Others		not specified		
Fushimi 2010	[HCSI initiative research paper: Factors associated with hospital selection of inpatients analyzed by patient survey data and DPC data]	DPC		Nationwide		2008	2008	Patient health service utilization	Health policy evaluation and utilization	Others		not specified		
Tanaka 2011	[[For more efficient management of the operating department: What is the index of efficiency?] Examination and multi-institutional comparison of the index of efficiency of operating room management]*	DPC		Nationwide		2006	2008	Research methodology		Others		Not specified		
Sato 2020	Retrieving and Analyzing Hospital Service Suspensions from Regional Healthcare Insurance Claims Data	Others	insurance claims dataset of 91 regional public insurance providers in the Mie prefectural area	Prefecture	Mie	2013	2017	Health policy evaluation and utilization		Others		Not specified		
Mitsutake 2020	[Examination of international statistical reports created by utilizing databases such as health insurance claims information]*	NDB	+NDB Open data	Nationwide		2009	2014	Medical treatment status	Health policy evaluation and utilization	Others		medical treatment information		
Kimura 2019	Validation and Recalibration of Charlson and Elixhauser Comorbidity Indices Based on Data From a Japanese Insurance Claims Database	JMDC		Nationwide		2011	2016	Prediction model	Research methodology	Others		Not specified	Others	aged ≥18 years
Doi 2018	[An Analysis of Factors Affecting Regional Differences in Home-care Utilization Levels Using Geographic Information Systems]	NHI/LSEHS	National Health Insurance and Later-Stage Elderly Healthcare System	Municipality	Funabashi city	2013	2014	Medical treatment status	Socioeconomic comparison	Others		Not specified	Older persons	aged ≥65 years
Tanihara 2017	[Comparison of the number of health insurance claims per population by major classification of injuries and diseases in the social medical treatment survey and society health insurance claims data]*	JMDC	Statistics of Medical Care Activities in Public Health Insurance (MHLW)	Nationwide		2014	2014	Research methodology		Others		Not specified		
Kimura 2015	[Same Examinations in Different Healthcare Providers in the Same Month of Referral, Analysis by Reimbursement Claim Database]	NDB	Sampling data	Nationwide		2011	2011	Medical treatment status		Others		same examinations in different healthcare provider		
Takenouchi 2013	[Combined Anonymization Considering Attackers in Data Holders]	JMDC		Nationwide		2005	2011	Research methodology		Others		Not specific (diabetes)		
Kobayashi 2012	[Calculation of clinical indices using DPC data: Focusing on AHRQ Patient Safety Indicators]*	DPC		Nationwide		2009	2009	Quality of care	Research methodology	Others		Patient Safety Indicator		
Ohbe 2021	Effect of Daikenchuto for Mechanically Ventilated Patients With Enteral Feeding Intolerance: A Propensity Score–Matched Analysis Using a Nationwide Administrative Inpatient Database	DPC		Nationwide		2010	2018	Intervention effect		Others		enteral feeding intolerance	Others	aged ≥18 years
Ohbe 2021	Small-bore feeding tube versus large-bore sump tube for early enteral nutrition in mechanically ventilated patients: A nationwide inpatient database study	DPC		Nationwide		2010	2018	Intervention effect		Others		early enteral nutrition in mechanically ventilated patients	Others	aged ≥18 years
Maeda 2019	A comparative analysis of treatment costs for home-based care and hospital-based care in enteral nutrition patients: A retrospective analysis of claims data	NHI/LSEHS	Tokyo Metropolitan Association of Medical Care for Senior Citizens	Prefecture	Tokyo	2013	2014	Health economics	Medical treatment status	Others		enteral nutrition patients	Older persons	aged 75 years or older
Tsugihashi 2021	Long-term prognosis of enteral feeding and parenteral nutrition in a population aged 75 years and older: a population-based cohort study	NHI/LSEHS	national health insurance claims in the Nara Prefecture	Prefecture	Nara	2014	2016	Clinical epidemiology, course of diseases		Others		Long - term prognosis of enteral feeding and parenteral nutrition	Older persons	aged ≥75 years
Kinoshita 2018	Prescription pattern and trend of oral contraceptives in Japan: A descriptive study based on pharmacy claims data (2006 - 2014)	Others	pharmacy claims datasets provided by the nationwide pharmacy chains	Nationwide		2006	2014	Medical treatment status		Others		oral contraceptives	Others	aged 11–59 years
Nakata 2017	Efficiency of inpatient orthopedic surgery in Japan: a medical claims database analysis	JMDC		Nationwide		2014	2014	Health policy evaluation and utilization		Others		inpatient orthopedic surgical case		
Watanabe 2018	Association between outpatient orthopedic surgery costs and healthcare facility characteristics	JMDC		Nationwide		2014	2014	Health economics	Health policy evaluation and utilization	Others		outpatient orthopedic surgery		
Shoda 2015	Prophylactic effect of fondaparinux and enoxaparin for preventing pulmonary embolism after total hip or knee arthroplasty: A retrospective observational study using the Japanese Diagnosis Procedure Combination database	DPC		Nationwide		2008	2010	Intervention effect		Others		after total hip or knee arthroplasty		
Chikuda 2013	Impact of age and comorbidity burden on mortality and major complications in older adults undergoing orthopaedic surgery: an analysis using the Japanese diagnosis procedure combination database	DPC		Nationwide		2007	2010	Clinical epidemiology, course of diseases	Intervention effect	Others		orthopaedic surgery	Others	patients aged 50 years or older
Kadono 2010	Statistics for orthopedic surgery 2006-2007: data from the Japanese Diagnosis Procedure Combination database	DPC		Nationwide		2006	2007	Medical treatment status		Others		orthopedic surgery		
Fuji 2017	Incidence of venous thromboembolism and bleeding events in patients with lower extremity orthopedic surgery: a retrospective analysis of a Japanese healthcare database	MDV		Nationwide		2008	2013	Intervention effect		Others	Diseases of the circulatory system	lower extremity orthopedic surgery, venous thromboembolism		

Urushihara 2018	[Socioeconomic impacts of policy on Rx-to-OTC switch to prescription of ethical drugs in Japan]	Others	National Prescription Audit (IMS Health)	Nationwide		2008	2014	Health policy evaluation and utilization	Health economics	Others	OTC switching		
Suzuki 2018	Trend in otolaryngological surgeries in an era of super-aging: Descriptive statistics using a Japanese inpatient database	DPC		Nationwide		2007	2014	Medical treatment status		Others	patients underwent otolaryngological surgeries		
Miyawaki 2017	Impact of medical subsidy disqualification on children's healthcare utilization: A difference-in-differences analysis from Japan	NHI/LSEHS	NHI	Municipality	a city within the Tokyo metropolitan	2012	2014	Health policy evaluation and utilization	Patient health service utilization	Others	outpatient service utilization	Children	school-age children
Nishioka 2020	Frequent outpatient attendance among people on the governmental welfare programme in Japan: assessing both patient and supplier characteristics	Others	medical assistance claim data, municipal public assistance databases	Municipality	two suburban municipalities, Osaka and Tokyo	2016	2016	Patient health service utilization	Socioeconomic comparison	Others	Frequent outpatient attendance		
Matsui 2018	Recent Trends in the Practice of Procedural Sedation Under Local Anesthesia for Catheter Ablation, Gastrointestinal Endoscopy, and Endoscopic Surgery in Japan: A Retrospective Database Study in Clinical Practice from 2012 to 2015	MDV		Nationwide		2012	2015	Medical treatment status	Clinical epidemiology, course of diseases	Others	catheter ablation, gastrointestinal endoscopic examination and surgery, dexmedetomidine	Others	aged ≥18 years
Yonekura 2018	Preoperative pulmonary function tests before low-risk surgery in Japan: a retrospective cohort study using a claims database	JMDC		Nationwide		2012	2016	Medical treatment status	Quality of care	Others	pulmonary function tests	Others	aged 20–64 years
Mitsutake 2020	Associations of Hospital Discharge Services With Potentially Avoidable Readmissions Within 30 Days Among Older Adults After Rehabilitation in Acute Care Hospitals in Tokyo, Japan	NHI/LSEHS	Tokyo Extended Association of Medical Care for Latter-Stage Older People	Prefecture	Tokyo	2013	2014	Intervention effect		Others	underwent rehabilitation and were discharged to home	Older persons	aged ≥75 years
Tsuchiya-Ito 2020	Association of household income with home-based rehabilitation and home help service utilization among long-term home care service users	NHI/LSEHS	LTC insurance claims and medical claims	Municipality	Kashiwa City	2012	2013	Socioeconomic comparison	Health policy evaluation and utilization	Others	home-based rehabilitation and home help services	Older persons	aged ≥65 years
Mitsutake 2018	[The outpatients of life rehabilitation by medical insurance service among older aged over 75 in Japan]	NHI/LSEHS		Prefecture	Tokyo	2014	2014	Health policy evaluation and utilization		Others	maintenance rehabilitation	Older persons	75 years or older
Matsuda 2018	[Big data analysis of medical and nursing care: An analysis of characteristics of outpatient rehabilitation and short-term intensive rehabilitation recipients]*	NHI/LSEHS	medical insurance claims and LTC insurance claims data	Others	municipalities in Western Japan	2011	2016	Clinical epidemiology, course of diseases	Health policy evaluation and utilization	Others	rehabilitation services	Older persons	Older People
Matsuda 2018	[Big data analysis of medical and nursing care: An analysis of user characteristics by type of home rehabilitation services]*	NHI/LSEHS	medical insurance claims and LTC insurance claims data	Others	municipalities in Western Japan	2015	2015	Health policy evaluation and utilization		Others	home rehabilitation	Older persons	Older People
Yoshikawa 2019	Effects of active smoking on postoperative outcomes in hospitalised patients undergoing elective surgery: a retrospective analysis of an administrative claims database in Japan	MDV		Nationwide		2018		Clinical epidemiology, course of diseases		Others	effects of smoking on prognosis after elective surgeries	Others	20 years of age or older
Mano 2015	Adherence to changing from brand-name to generic atorvastatin in newly treated patients: a retrospective cohort study using health insurance claims	JMDC		Nationwide		2011	2012	Patient health service utilization	Health policy evaluation and utilization	Others	statin therapy	Others	aged ≥18 years
Takizawa 2015	Price difference as a predictor of the selection between brand name and generic statins in Japan	JMDC		Nationwide		2008	2011	Patient health service utilization	Health policy evaluation and utilization	Others	statin users		
Gao 2021	Comparison of adherence, persistence, and clinical outcome of generic and brand-name statin users: A retrospective cohort study using the Japanese claims database	JMDC		Nationwide		2014	2016	Patient health service utilization	Health policy evaluation and utilization	Others	statin users	Others	aged ≥18 years
Ohbe 2021	Clinical Trajectories of Suicide Attempts and Self-harm in Patients Admitted to Acute-care Hospitals in Japan: A Nationwide Inpatient Database Study	DPC		Nationwide		2015	2017	Clinical epidemiology, course of diseases	Medical treatment status	Others	patients hospitalized for suicide attempts or selfharm	Others	aged ≥10 years
Miyawaki 2021	Changes in Surgeries and Therapeutic Procedures During the COVID-19 Outbreak: A Longitudinal Study of Acute Care Hospitals in Japan	MDV		Nationwide		2019	2020	Medical treatment status	COVID-19	Others	number of surgeries during the COVID-19 outbreak		
Koizumi 2019	Tranexamic acid and post-tonsillectomy hemorrhage: propensity score and instrumental variable analyses	DPC		Nationwide		2010	2016	Intervention effect		Others	patients who had undergone tonsillectomy	Others	excluded aged more than 61 years
Shiragami 2013	[An empirical study reviewing factors impacting coverage of the topical adhesive patch by health insurance]	JMDC		Nationwide		2008	2010	Medical treatment status	Health policy evaluation and utilization	Others	topical adhesive patch		
Mizuno 2019	Indications and outcomes of paediatric tracheotomy: a descriptive study using a Japanese claims database	JMDC		Nationwide		2005	2017	Medical treatment status	Intervention effect	Others	tracheotomy	Children	aged 0–15 years
Kitazawa 2017	Cost Analysis of Transplantation in Japan, Performed With the Use of the National Database	NDB		Nationwide		2009	2010	Health economics		Others	Transplantation		

Supplement file 5 List of frequently occurring diseases

1) Certain infectious and parasitic diseases	hepatitis B and C, sepsis/ septic shock
2) Neoplasms	cancer (general), colon cancer, liver cancer, lung cancer
3) Diseases of the blood and blood-forming organs and certain disorders involving the immune mechanism	disseminated intravascular coagulation (DIC)
4) Endocrine, nutritional and metabolic diseases	diabetes (with dental, hypertension, kidney outcomes etc.), obesity
5) Mental, Behavioral and Neurodevelopmental disorders	psychiatric disorders (general), psychotropic/ antidepressant/ antipsychotics, depressive disorders, schizophrenia, dementia
6) Diseases of the nervous system	dementia (Alzheimer disease), Parkinson's Disease
7) Diseases of the eye and adnexa	glaucoma
8) Diseases of the ear and mastoid process	otitis media
9) Diseases of the circulatory system	cardiovascular diseases (general), atrial fibrillation, cardiac arrest, heart failure, myocardial infarction, coronary syndrome/ intervention, hypertension, ischemic stroke, stroke cases
10) Diseases of the respiratory system	asthma, COPD, influenza, respiratory infections, pneumonia (including aspiration pneumonia)
11) Diseases of the digestive system	acute pancreatitis, dental, gastrointestinal bleeding
12) Diseases of the skin and subcutaneous tissue	atopic dermatitis, psoriasis
13) Diseases of the musculoskeletal system and connective tissue	osteoporosis, rheumatoid arthritis
14) Diseases of the genitourinary system	chronic kidney disease (CKD), dialysis
15) Pregnancy, childbirth and the puerperium	psychiatric disorders in pregnancy (psychotropics, antidepressant)
16) Certain conditions originating in the perinatal period	(diverse diseases)
17) Congenital malformations, deformations and chromosomal abnormalities	(diverse diseases)
18) Injury, poisoning and certain other consequences of external causes	fracture (particularly hip fracture)
19) Others (multidisease, not focused on specific diseases)	antimicrobial use, healthcare costs, hospitalizations/ ICU, multidisease, not specified

Supplement file 6 Research theme by diseases

		*medical treatme		*intervention effe		*clinical epidemi		*health economi		*health policy ev		*quality of care		*research metho		*patient health s		*socioeconomic		*prediction mode		*COVID-19		*others	
	Total	n	%	n	%	n	%	n	%	n	%	n	%	n	%	n	%	n	%	n	%	n	%	n	%
1) Certain	117	37	31.6	24	20.5	51	43.6	21	17.9	11	9.4	6	5.1	5	4.3	0	0.0	1	0.9	2	1.7	2	1.7	0	0.0
2) Neoplas	162	52	32.1	55	34.0	31	19.1	22	13.6	19	11.7	7	4.3	8	4.9	3	1.9	6	3.7	3	1.9	0	0.0	1	0.6
3) Disease	19	4	21.1	11	57.9	4	21.1	1	5.3	0	0.0	1	5.3	1	5.3	0	0.0	0	0.0	0	0.0	0	0.0	0	0.0
4) Endocri	171	45	26.3	44	25.7	60	35.1	24	14.0	7	4.1	9	5.3	8	4.7	21	12.3	6	3.5	1	0.6	1	0.6	0	0.0
5) Mental,	110	54	49.1	23	20.9	28	25.5	14	12.7	17	15.5	9	8.2	2	1.8	7	6.4	5	4.5	1	0.9	0	0.0	2	1.8
6) Disease	69	27	39.1	22	31.9	19	27.5	11	15.9	6	8.7	6	8.7	2	2.9	1	1.4	2	2.9	2	2.9	0	0.0	1	1.4
7) Disease	22	8	36.4	5	22.7	5	22.7	2	9.1	1	4.5	1	4.5	1	4.5	5	22.7	0	0.0	0	0.0	0	0.0	0	0.0
8) Disease	4	1	25.0	3	75.0	1	25.0	0	0.0	2	50.0	1	25.0	0	0.0	0	0.0	0	0.0	0	0.0	0	0.0	0	0.0
9) Disease	281	65	23.1	99	35.2	84	29.9	25	8.9	37	13.2	19	6.8	13	4.6	15	5.3	9	3.2	4	1.4	2	0.7	0	0.0
10) Diseas	135	35	25.9	47	34.8	46	34.1	14	10.4	8	5.9	11	8.1	3	2.2	4	3.0	2	1.5	6	4.4	3	2.2	0	0.0
11) Diseas	115	24	20.9	52	45.2	29	25.2	24	20.9	11	9.6	12	10.4	3	2.6	4	3.5	1	0.9	1	0.9	0	0.0	0	0.0
12) Diseas	17	8	47.1	6	35.3	5	29.4	6	35.3	1	5.9	0	0.0	0	0.0	2	11.8	1	5.9	0	0.0	0	0.0	0	0.0
13) Diseas	93	28	30.1	33	35.5	39	41.9	15	16.1	4	4.3	4	4.3	1	1.1	5	5.4	2	2.2	1	1.1	0	0.0	0	0.0
14) Diseas	75	16	21.3	32	42.7	29	38.7	10	13.3	6	8.0	5	6.7	0	0.0	2	2.7	2	2.7	3	4.0	0	0.0	0	0.0
15) Pregna	25	11	44.0	8	32.0	10	40.0	1	4.0	1	4.0	0	0.0	0	0.0	0	0.0	2	8.0	0	0.0	0	0.0	0	0.0
16) Certai	8	2	25.0	4	50.0	1	12.5	1	12.5	1	12.5	0	0.0	0	0.0	0	0.0	0	0.0	0	0.0	1	12.5	0	0.0
17) Conge	13	3	23.1	5	38.5	4	30.8	1	7.7	2	15.4	1	7.7	1	7.7	0	0.0	0	0.0	1	7.7	0	0.0	1	7.7
18) Injury,	110	15	13.6	48	43.6	47	42.7	10	9.1	10	9.1	2	1.8	5	4.5	0	0.0	3	2.7	2	1.8	0	0.0	3	2.7
19) Others	238	74	31.1	38	16.0	38	16.0	38	16.0	62	26.1	11	4.6	30	12.6	15	6.3	17	7.1	12	5.0	4	1.7	2	0.8

*up to two diseases/ theme could be choose
